# Mobile Phone and Web 2.0 Technologies for Weight Management: A Systematic Scoping Review

**DOI:** 10.2196/jmir.5129

**Published:** 2015-11-16

**Authors:** Marco Bardus, Jane R Smith, Laya Samaha, Charles Abraham

**Affiliations:** ^1^ Psychology Applied to Health research group Institute of Health Research University of Exeter Medical School Exeter United Kingdom; ^2^ Institute for Public Communication Faculty of Communication Sciences Università della Svizzera italiana Lugano Switzerland; ^3^ Institute of Communication and Health Faculty of Communication Sciences Università della Svizzera italiana Lugano Switzerland

**Keywords:** obesity, overweight, review, cellular phone, mobile apps, social media, mobile health, mHealth, mobile phone, Web 2.0

## Abstract

**Background:**

Widespread diffusion of mobile phone and Web 2.0 technologies make them potentially useful tools for promoting health and tackling public health issues, such as the increasing prevalence of overweight and obesity. Research in this domain is growing rapidly but, to date, no review has comprehensively and systematically documented how mobile and Web 2.0 technologies are being deployed and evaluated in relation to weight management.

**Objective:**

To provide an up-to-date, comprehensive map of the literature discussing the use of mobile phone and Web 2.0 apps for influencing behaviors related to weight management (ie, diet, physical activity [PA], weight control, etc).

**Methods:**

A systematic scoping review of the literature was conducted based on a published protocol (registered at PROSPERO: CRD42014010323). Using a comprehensive search strategy, we searched 16 multidisciplinary electronic databases for original research documents published in English between 2004 and 2014. We used duplicate study selection and data extraction. Using an inductively developed charting tool, selected articles were thematically categorized.

**Results:**

We identified 457 articles, mostly published between 2013 and 2014 in 157 different journals and 89 conference proceedings. Articles were categorized around two overarching themes, which described the use of technologies for either (1) promoting behavior change (309/457, 67.6%) or (2) measuring behavior (103/457, 22.5%). The remaining articles were overviews of apps and social media content (33/457, 7.2%) or covered a combination of these three themes (12/457, 2.6%). Within the two main overarching themes, we categorized articles as representing three phases of research development: (1) design and development, (2) feasibility studies, and (3) evaluations. Overall, articles mostly reported on evaluations of technologies for behavior change (211/457, 46.2%).

**Conclusions:**

There is an extensive body of research on mobile phone and Web 2.0 technologies for weight management. Research has reported on (1) the development, feasibility, and efficacy of persuasive mobile technologies used in interventions for behavior change (PA and diet) and (2) the design, feasibility, and accuracy of mobile phone apps for behavioral assessment. Further research has focused exclusively on analyses of the content and quality of available apps. Limited evidence exists on the use of social media for behavior change, but a segment of studies deal with content analyses of social media. Future research should analyze mobile phone and Web 2.0 technologies together by combining the evaluation of content and design aspects with usability, feasibility, and efficacy/effectiveness for behavior change, or accuracy/validity for behavior assessment, in order to understand which technological components and features are likely to result in effective interventions.

## Introduction

A recent consensus statement on the prevention and management of noncommunicable diseases stressed the need to focus on behavior change and to develop more user-centered, effective, and efficient preventive programs [1]. Overweight and obesity together are the fifth leading risk factor for global deaths, accounting for around 3.4 million deaths every year [2], making it a global public health priority [3-5]. Technology-based health services, delivered or enhanced through the Internet (ie, eHealth technologies) [6] and, in particular, mobile technologies, offer great potential to increase the reach of public health initiatives and to improve public health [7,8]. For example, behavioral and biomedical “big data” collected through ubiquitous mobile phones and their sensors [9] could be used to predict health trends and illnesses [10], hence optimizing the delivery of health care programs [11].

This potential is enhanced by increasing adoption rates for mobile and Internet technologies. In 2014, there were 6.5 billion mobile subscribers (93% of the entire world population) [12], with mobile phone penetration rates reaching over 70% of the population of many European and North American countries, such as Spain (83%), Canada (78%), the United Kingdom (75%), the United States (73%), and Italy (71%) [13]. Mobile phones allow users to access various Internet services, in particular social media profiles. In Europe, social media are accessed through mobile devices by 26% of the total population (66% of the total active social media population) [14]. Social media apps that “build on the ideological and technological foundations of Web 2.0, and that allow the creation and exchange of User Generated Content” [15] involve very large segments of the population. A recent Pew Research Center’s Internet & American Life Project report showed that 81% of online US adults are active social media users, with Facebook being the most popular social networking site, used by 58% of the population [16]. Similarly, in Europe there are 300 million active social media users (about 40% of the entire population), with Northern Europe having the highest rates for Facebook use (56%) [14].

The growth in mobile phone and social media usage supports widespread adoption and diffusion of mobile phone apps. At the end of 2014, there were 1.4 million apps available on Google Play, 1.2 million in the iTunes App Store, and 290,000 in the Amazon app stores [17]. In particular, the use of health and fitness apps has recently shown a rapid and steady growth. In December 2014, compared to the previous year, the time spent on apps in the health and fitness and sport categories increased by 89% and 74%, respectively [18]. Recent surveys show that 19% of mobile phone owners reported downloading an app to track or manage their health [19] which (1) helps set health-related goals (30%), (2) assists with health-related searches (28%), (3) enhances health-related motivation (27%), (4) identifies unhealthy habits (7%), or (5) supports adherence to medications (5%) [20]. The most popular health apps are used for tracking and monitoring physical activity (38%) and diet (31%), and for managing weight (12%) [19].

Following the trends in technology development, the eHealth research literature has increased considerably in the last decade. For example, a PubMed search for eHealth-related terms on June 4, 2015, resulted in 20,176 hits for “eHealth,” and 1166 hits for “eHealth interventions.” There were also 3148 hits for “eHealth review.” This trend is also reflected in the introduction of specific Medical Subject Headings (MeSH) topics, which are used by major electronic databases such as PubMed/Medline, Cochrane Library, and Web of Science. As of July 4, 2015, the MeSH major topic “Cell Phones” (introduced in 2003) produced 4481 hits (373.4 hits/year), whereas “Mobile Applications” (introduced in 2014) produced 357 hits in a year. For Web 2.0 technologies, the coverage is smaller but still indicative of a growing field; the general umbrella MeSH topic “Social Media” (introduced in 2012) produced 1369 hits (456.3 hits/year), whereas the specific term “Social Networking” (introduced in 2012) yielded 732 hits (146.4 hits/year), and “Blogging” (introduced in 2010) yielded only 401 hits (80.2 hits/year).

An increasing number of systematic reviews and meta-analyses on eHealth interventions are available. These evaluate their impact on general health promotion [21], specifically including smoking [22], weight management [23], and diet and physical activity (PA) [24], or they assess effects on health care program delivery [25] and treatments (eg, HIV [26]). Some scoping reviews have described the use of mobile and Web 2.0 technologies specifically for general health behavior change [27-31]. However, most focus on mobile technologies alone [28-31] and do not provide a comprehensive picture of the research involving both Web 2.0 and mobile phone technologies for weight management in particular. The depth and breadth of the potentially relevant literature in this domain prompts exploration of the field in the form of a scoping review [32]. Scoping reviews generally provide an overview or a map of the available literature, hence determining the *scope* of subsequent systematic reviews, which will have narrower or more focused research questions, detailed data extraction, and study quality assessment [33]. Scoping reviews allow researchers to synthesize the literature and to highlight potential gaps and parameters in the available literature.

Therefore, the aim of this scoping review is to provide a systematic, comprehensive, and updated overview of eHealth research into use of mobile phones and Web 2.0 technologies for weight management over the past decade. The general research questions that guided this scoping review were as follows: (1) What is the current state of research discussing the use of mobile phones in combination with Web 2.0 technologies for weight management?, (2) What type of research has investigated these technologies?, and (3) On which methodological and technological aspects has this research focused?

## Methods

### Overview

We conducted a systematic scoping review of the literature describing the role of mobile phone and Web 2.0 technologies for weight management. This review was based on a published protocol (registered at PROSPERO: CRD42014010323) [34]. In accordance with Arksey and O’Malley’s proposed framework for scoping reviews [32], we provide a qualitative, descriptive, comprehensive chart/map of the literature on the topic. The chart covers aspects related to design, implementation, and evaluation of mobile and social media technologies employed for promoting and assessing behaviors associated with weight management, in the broader context of obesity prevention initiatives.

### Information Sources

Articles were identified through a comprehensive search in the following 16 electronic databases, covering medicine and behavioral, social, and computer sciences, considering the multidisciplinary nature of the topic: PubMed/Medline, Embase, Global Health, the Cumulative Index to Nursing and Allied Health Literature (CINAHL), PsycINFO, the Cochrane Library (including the Database of Systematic Reviews, the Central Register of Controlled Trials, and the Database of Abstracts of Reviews of Effects), SPORTDiscus, PsycARTICLES, the Psychology & Behavioral Sciences Collection, the Education Resources Information Center (ERIC), Communication and Mass Media Complete, the Association for Computing Machinery (ACM) Digital Library, Institute of Electrical and Electronics Engineers (IEEE) Xplore, the Web of Science Core Collection (including Science Citation Index Expanded, Social Sciences Citation Index, Arts & Humanities Citation Index, Conference Proceedings Citation Index-Science, and Conference Proceedings Citation Index-Social Science & Humanities), and the “grey" literature sources WorldCat Dissertations (via Online Computer Library Center [OCLC] FirstSearch) and OpenGrey. Reference lists of the included studies and reviews were also screened for additional references.

### Search Strategy

Applying the PICOS (participants, interventions, comparators, outcomes, and study design) framework [35], a comprehensive search strategy included keywords and MeSH to describe the population (ie, any population, being obese/overweight, healthy, or interested in weight management), interventions/comparators (ie, mobile phones and social media), and outcomes (ie, weight, body mass index [BMI], diet, and physical activity). We included any study design. The strategy was developed by employing terms and MeSH used in related systematic reviews (eg, on weight management and diet [36], PA [37], mobile phones and mHealth [29,38], and social media [39,40]). Searches were restricted to publications available in English from January 1, 2004 to December 31, 2014 to ensure that relevant modern technologies were included. A sample of the search strategies used across databases and in Medline (Ovid) is provided in the Multimedia Appendix 1. Preliminary searches were conducted in June and July 2014; final searches were conducted in August 2014, and updated on February 27, 2015.

### Eligibility Criteria

We considered any type of primary research article or review describing the use of mobile phones or Web 2.0 technologies (ie, interventions) in relation to weight management and related behaviors (ie, outcomes), including any study design or type, and among any population group. Hence, any article was included that addressed the role of mobile devices and/or Web 2.0 technologies to measure, track, or encourage change in the behaviors that contribute to weight management (ie, PA and/or diet) for the prevention of overweight and obesity. We defined mobile devices as mobile phones, personal digital assistants (PDAs), and handheld and ultraportable computers such as tablets (eg, iPads) [25]. We defined Web 2.0 technologies as “Internet-based applications that allow the creation and exchange of User Generated Content and include social networking sites, collaborative projects, micro-blogging and blogging tools, content communities, virtual worlds” [15].

We excluded the following types of studies: general epidemiological studies on the use of the technologies (eg, effects of radiation from mobile phone use on brains and cells, or their association with cancer; penetration rates of mobile phones in households); studies where mobile phones were simply used as methods for data collection without any further reporting on, or testing of, the assessment methods, and research into mobile and Web 2.0 technologies for clinical management (eg, as decision support tools for health professionals); and studies where mobile phones were used for the self-management of chronic conditions (eg, diabetes, chronic obstructive pulmonary disease [COPD], and heart failure) where weight management was not the primary focus (eg, interventions using mobile phone apps to manage type 2 diabetes in obese patients where the main focus was on blood glucose control). We also excluded articles discussing the use of other technologies alone, such as video game consoles, virtual reality devices, computers, laptops, pagers, land phones, and wearable devices (eg, Fitbit, Nike+, and Jawbone UP), as well as traditional websites with no social media components specified.

### Study Selection, Categorization, and Data Extraction

Articles were selected in a two-step process, which involved two reviewers (MB and LS) who independently screened first the title and abstract, and then the full text of the retrieved articles applying the inclusion/exclusion criteria. One reviewer (MB) completed an initial categorization of the selected articles using an inductively developed “charting” tool (provided in Multimedia Appendix 2), which was improved upon with input from the other authors, and checked for consistency by another reviewer (JS). Data extracted included the following: author’s name, year of publication, country of origin, study objectives/purpose (as reported verbatim by the authors), targeted behaviors (eg, PA, diet, or weight loss/management), target population (ie, age group, health status/condition, and gender where explicitly indicated in the paper), type of technology (ie, mobile, Web 2.0, etc, with additional details about the type of mobile and Web 2.0 technology, operating system, and devices tested when reported), and type of study (eg, descriptive, qualitative, mixed methods, randomized controlled trial [RCT], or other quantitative studies). We also linked articles that presented data on, or analyzed data from, RCTs to their reported trial registry number (see Multimedia Appendix 3). As this is a scoping review, we did not assess the risk of bias in studies, heterogeneity, or publication bias.

Inter-rater reliability estimates were calculated (see below) and all disagreements were resolved through discussion until consensus was reached in all steps. For selection and data extraction, we evaluated inter-rater reliability using Gwet’s first-order agreement coefficient (AC1) statistic [41], a reliable alternative to Cohen’s kappa. Gwet's AC1 does not underestimate reliability when the number of instances is small or when there is an asymmetric distribution between agreements and disagreements, as is likely to occur when screening a large number of titles and abstracts [41,42].

### Analyses

A qualitative synthesis of the included studies was undertaken to map the literature as outlined in the research questions. Data were summarized using descriptive frequency tables for the inductively developed categories describing the purpose of the paper, its methodology, and the data reported. The literature was summarized according to the emerging research themes and technology used.

## Results

### Search Results and Study Selection

**Figure 1:**
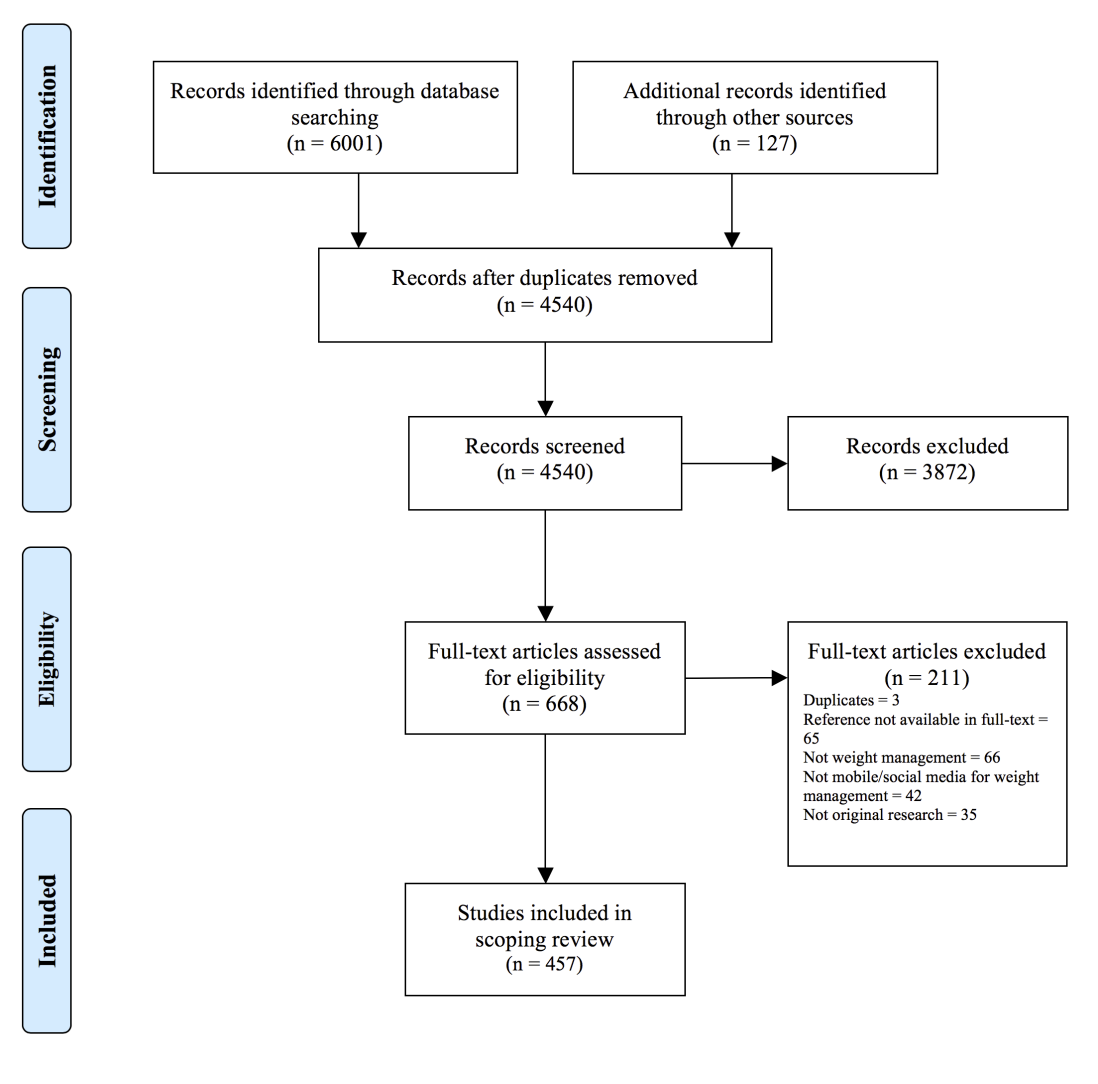
PRISMA flow diagram.

The search across the 16 electronic databases yielded a total of 6001 records; reference lists and other sources yielded an additional 127 references. After duplicate removal, one reviewer (MB) and a temporary research assistant screened the titles and abstracts of 4540 records, excluding 3872 entries (92% agreement; AC1 .91, 95% CI .89-.92). To ensure consistency in the application of inclusion/exclusion criteria, a third reviewer (JS) screened a randomly selected 5% sample of the references, achieving a 91% agreement with the previous judgments (AC1 .88, 95% CI .83-.94). The remaining 668 references were assessed in full text by the original reviewer (MB) and a second reviewer (LS), and a further 211 were excluded (90% agreement; AC1 .82, 95% CI .79-.85), leaving 457 articles included in this review (Preferred Reporting Items for Systematic Reviews and Meta-Analyses [PRISMA] diagram [43] in Figure 1). As in the first step, a third reviewer (JS) screened a 20% randomly selected sample of full-text articles, achieving 97% agreement and good reliability (AC1 .94, 95% CI .88 - .99). A table with the excluded references and the reasons for exclusion is provided in Multimedia Appendix 4.

### Article Characteristics

**Figure 2:**
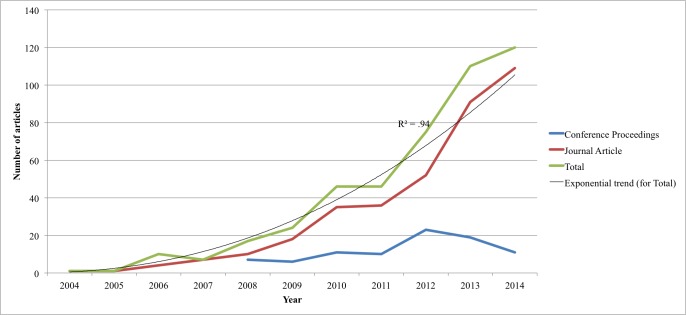
Distribution of articles included in scoping review (n=457).

The majority of the 457 included references (364/457, 79.6%) were published as journal articles in 157 different publications, covering a variety of disciplines including medical and health sciences, computing and informatics, education, and psychology. A relatively large number of publications came from high-ranking journals in the fields of medical informatics, health services research, and public health, such as the Journal of Medical Internet Research (36/364, 9.9%), BMC Public Health (21/364, 5.8%), the American Journal of Preventive Medicine (14/364, 3.8%), and the Journal of Telemedicine and Telecare (13/364, 3.6%). The remainder were published as conference proceedings (93/457, 20.4%) presented at 89 different conferences, mostly focusing on pervasive computing and design. More than half of the journal articles were published after 2013 (255/457, 55.8%, range 2004-2014), while the median year for publication in conference proceedings was 2012 (interquartile range [IQR] 3; 63/93, 68% published after 2012, range 2006-2014). Figure 2 shows an overall exponential trend (R^2^= .94) with the number of journal articles and conference proceedings growing considerably after 2008. Conference proceedings peaked in 2012 and declined progressively as publication in journal articles continued to increase.

Overall, the research was conducted in 39 countries, the majority of which were English-speaking (334/457, 73.1%), including the United States (219/457, 47.9%), Australia (54/457, 11.8%), the United Kingdom (30/457, 6.6%), Canada (17/457, 3.7%), New Zealand (8/457, 1.8%), and Ireland (6/457, 1.3%). Included research was also undertaken in other European countries (75/457, 16.4%), Southeast Asia (35/457, 7.7%), and the Middle East (11/457, 2.4%). Only 2 out of 457 (0.4%) publications originated from Latin and Central America—1 (0.2%) from Brazil and 1 (0.2%) from Mexico—and no articles originated from African countries.

### Categorization of Selected Studies

The first author (MB) developed the charting tool using an initial sample of 40 papers, and the judgements were independently validated by the second author (JS), first using a random sample of 10 papers (80% agreement; AC1 .79, 95% CI .28 - 1.00), then 113 out of 457 (24.7%) included papers, achieving 94% agreement and good reliability (AC1 .94, 95% CI .89 - .98). The second author checked the categorization and data extraction for 239 articles out of 457 (52.3%) for consistency.

### Overarching Research Themes

We categorized primary research and review evidence using three overarching themes. The majority of studies described the role of mobile and Web 2.0 technologies for the first theme, *promoting behavior change* (308/457, 67.4%; 263 primary research articles and 45 reviews), and the second theme, *measuring behavior* (103/457, 22.5%; 96 primary research articles and 7 reviews). The first group included articles that discussed the use of technologies to shape behavior patterns related to managing weight. Technology was construed as a delivery mode for interventions promoting behavior change (eg, through self-monitoring, providing feedback, reminding, and motivating). The second group included articles that specifically focused on the development and evaluation of technologies for assessing physical activity or dietary behaviors, without reporting data on their effects on behavior or weight-related outcomes. These studies focused on data describing the accuracy or validity of apps or systems for physical activity and dietary assessment (eg, activity recognition, energy expenditure estimation, activity classification, food classification and caloric intake estimation, and comparison between self-reported, paper-and-pencil, and objective measures of behavior using the technologies).

A third theme, *overviews of apps and social media content*, encompassed 33 articles out of 457 (7.2%), which presented primary research concerning content analyses of social media and reviews of mobile phone apps and their content. These articles did not focus on the impact of the technologies on behavior or behavioral assessment, but rather on the content characteristics of the media (eg, healthy living blogging communities [44] or Twitter conversations about weight loss [45]). The remaining articles (14/457, 3.1%) included aspects of two or more of the themes. Out of 457 articles, 9 (2.0%) referred to behavior change and behavioral assessment. Of these 9 articles, 6 (67%) were primary research articles describing the development of mobile-based methods for dietary assessment and intervention [46,47], mobile phone apps for exercise monitoring and analysis [48,49], and apps for food intake and calorie balance monitoring [50,51]. Of the 9 articles, 3 (33%) were reviews describing mobile technologies for assessing and promoting PA [52,53] and diet [54]. A further 3 (33%) reviews out of 9 articles included aspects that could be categorized under both the first and third theme: a narrative review and content analysis of a Dutch PA and diet blogging community (Valtaf.nl) [55], a systematic review on mobile phone apps for women’s health promotion [56], and a systematic review on mobile phone apps for food intake [57]. Hence, accounting for the overlaps between overarching themes (ie, the articles that covered both aspects are counted twice for each category), there were 468 categorizations in total. The majority of the articles covered *promoting behavior change* (318/468, 67.9%), of which 269 (84.6%) were primary research articles and 49 (15.4%) were reviews: 5 (1.6%) nonsystematic reviews, 5 (1.6%) general scoping reviews, 28 (8.8%) systematic reviews with qualitative syntheses, and 11 (3.5%) meta-analyses. The theme *measuring behavior* included 112 articles out of 468 total categorizations (23.9%), of which 101 (90.2%) were primary research articles and 11 (9.8%) were reviews: 2 (1.8%) nonsystematic reviews and 9 (8.0%) qualitative syntheses. The theme *overviews of social media and mobile phone apps* included 36 articles (36/468, 7.7%).

### Research Themes

Within two of the main overarching themes—*behavior change* and *measuring behavior*—three research themes emerged from the data. These represent the progressive stages in research: (1) *design and development*, (2) *feasibility*, and (3) *evaluations*. *Design and development* included articles describing systems design [58] or the development of apps and platforms aimed at influencing or assessing behavior without reporting data on their effects, their usability, acceptability, or feasibility. *Feasibility* represented articles describing the results of pilot/feasibility studies focusing on process and procedural outcomes (eg, acceptability, participation, utilization, retention and recruitment, adherence, or compliance), rather than on the effects on behavior or on the accuracy/validity of behavioral assessment. Finally, *evaluations* included studies presenting the effects of technology-based interventions on behavior or weight-related outcomes, or technology-based methods for assessing behavior. Within the evaluations discussing behavior change, we created a distinctive subcategory—*process/outcome evaluations or causal-comparative studies*—which included primary research articles examining sociocognitive or technological factors associated with outcomes in the context of existing interventions, without directly reporting on the effects of the technology on behavior. We created subcategories to account for articles covering a combination of two or three of the research themes described above, which constitute the overlap between the themes. For example, we identified the concept of *usability* as indicating the overlap between *design and development* and *feasibility*, and used it to categorize articles that described the development of a system and measured outcomes such as ease of use, learnability, task efficiency, memorability, satisfaction, and usefulness [59]. The term usability was used with similar connotations in articles from different research fields to describe the elements associated with feasibility and acceptability of technologies in interventions and pilot studies.

Among the total of 366 primary research articles covering the two overarching research themes, *design and development* was discussed in 139 articles (38.0%): 87 (23.8%) covered *promoting behavior change*, 48 (13.1%) covered *measuring behavior*, and 4 (1.1%) covered both overarching themes. *Feasibility* was discussed in 191 primary research articles out of 366 (52.2%): 154 (42.1%) covered *promoting behavior change*, 35 (9.6%) covered *measuring behavior*, and 2 (0.5%) covered both overarching themes. *Evaluation* was reported in 247 primary articles out of 366 (67.5%): 167 (45.6%)—including 20 process evaluation papers—were related to *behavior change*, 75 (20.5%) were related to *measuring behavior*, and 3 (0.8%) covered both overarching themes.

Among the 58 reviews covering the two overarching research themes, 11 (19%) were narrative reviews: 5 (9%) nonsystematic reviews and 5 (9%) systematic scoping reviews on the use of technologies for general health promotion, and 1 (2%) on the use of technologies for dietary assessment [54]. All of these discussed the uses of technologies in general, recognizing their potential for behavior change or behavioral assessment without specifically focusing on design, development, or evaluations. Only 1 review out of 58 (2%) also covered aspects of *feasibility* in conjunction with the evaluation of accuracy and validity of technologies for dietary assessment [60]. No review explicitly reported on the feasibility of technologies for behavior change. The remaining 47 out of 58 reviews (81%) reported data on *evaluation* of technologies for behavior change, 9 (16%) for behavioral assessment, and 2 (3%) for both behavior change and assessment.

**Figure 3:**
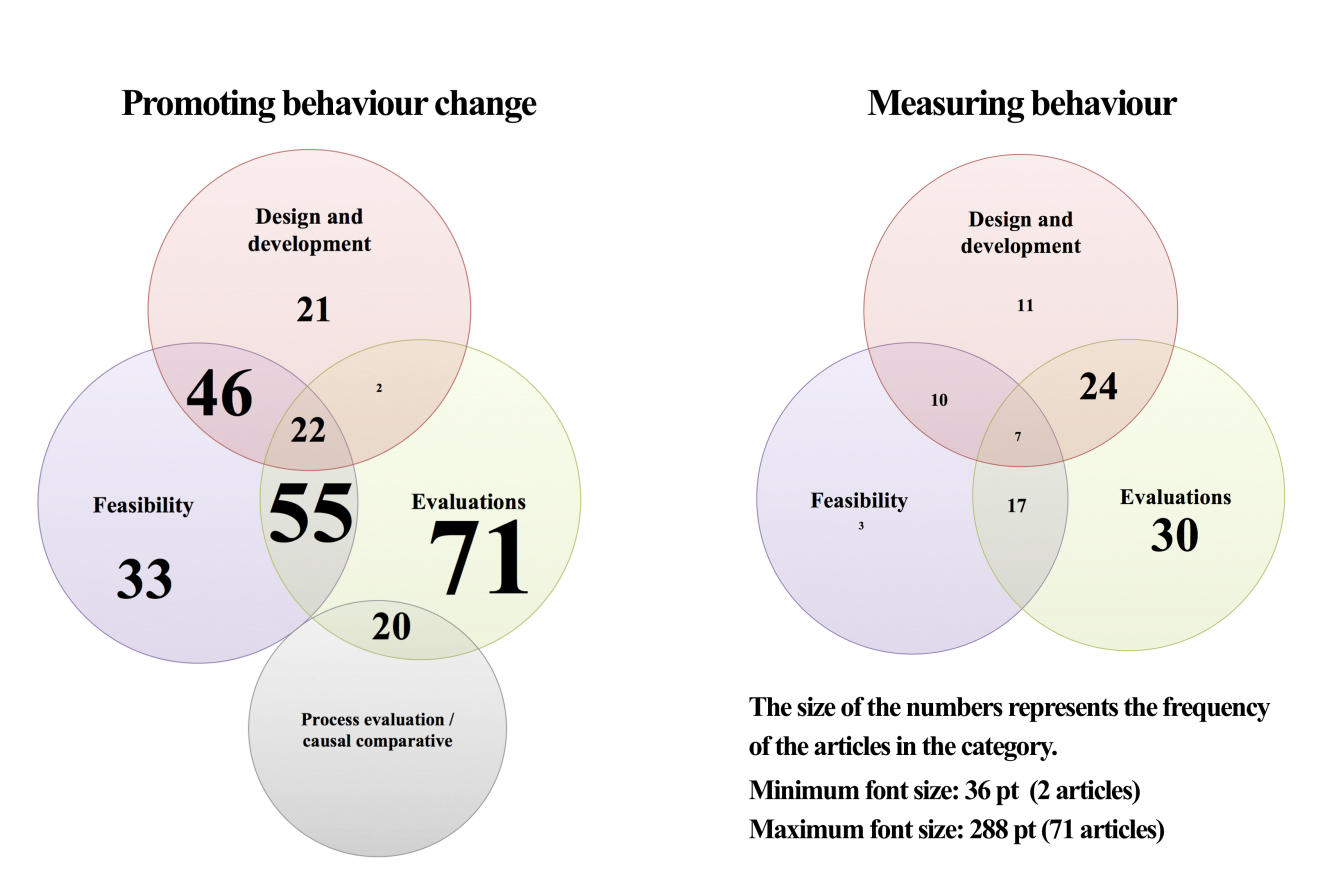
Venn diagrams representing the main research themes for primary research articles (n=366).

A visual summary of the research themes for primary research studies is presented in the Venn diagrams in Figure 3. Table 1 describes the distribution of primary research studies according to research themes and technology type. Articles that appear in more than one research theme are counted twice. The characteristics of the individual articles grouped by research theme and technology type are reported in Multimedia Appendix 3. Examples are provided in the following paragraphs.

**Table 1:** Distribution of primary research articles (n=366) according to research theme and technology used.

Research themes		Technology			Total (n=366), n (%)
		Mobile, n (%)	Web 2.0, n (%)	Mobile and Web 2.0, n (%)	
**Promoting behavior change**		192 (52.5)	31 (8.5)	41 (11.2)	264 (72.1)
	Design	11 (3.0)	1 (0.3)	8 (2.2)	20 (5.5)
	Feasibility	26 (7.1)	4 (1.1)	3 (0.8)	33 (9.0)
	Evaluations	54 (14.8)	6 (1.6)	9 (2.5)	69 (18.9)
	Process evaluations	7 (1.9)	8 (2.2)	5 (1.4)	20 (5.5)
	Design and evaluations	1 (0.3)	0 (0)	0 (0)	1 (0.3)
	Design and feasibility	31 (8.5)	4 (1.1)	9 (2.5)	44 (12.0)
	Feasibility and evaluations	46 (12.6)	5 (1.4)	4 (1.1)	55 (15.0)
	Design, feasibility, and evaluations	16 (4.4)	3 (0.8)	3 (0.8)	22 (6.0)
**Measuring behavior**		94 (25.7)	0 (0)	2 (0.5)	96 (26.2)
	Design	10 (2.7)	0 (0)	0 (0)	10 (2.7)
	Feasibility	3 (0.8)	0 (0)	0 (0)	3 (0.8)
	Evaluations	28 (7.7)	0 (0)	0 (0)	28 (7.7)
	Design and evaluations	22 (6.0)	0 (0)	1 (0.3)	23 (6.3)
	Design and feasibility	7 (1.9)	0 (0)	1 (0.3)	8 (2.2)
	Feasibility and evaluations	17 (4.6)	0 (0)	0 (0)	17 (4.6)
	Design, feasibility, and evaluations	7 (1.9)	0 (0)	0 (0)	7 (1.9)
**Promoting behavior change and measuring behavior**		6 (1.6)	0 (0)	0 (0)	6 (1.6)
	Design	1 (0.3)	0 (0)	0 (0)	1 (0.3)
	Evaluations	2 (0.5)	0 (0)	0 (0)	2 (0.5)
	Design and feasibility	1 (0.3)	0 (0)	0 (0)	1 (0.3)
	Design, feasibility, and evaluations	2 (0.5)	0 (0)	0 (0)	2 (0.5)
Total		292 (79.8)	31 (8.5)	43 (11.7)	366 (100)

### Research on Technologies for Promoting Behavior Change

The majority of primary research studies dealing with behavior change (138/270, 51.1%) were published in the last 2 years. Most of these focused on mobile technologies (198/270, 73.3%). Research was comprised mostly of *evaluations* (127/198, 64.1%), followed by *feasibility* (121/198, 61.1%), and *design and development* studies (63/198, 31.8%). Examples of studies describing *design and development* and *usability* included testing of research-based apps (eg, UbiFit Garden [61,62] or bActive [63]) or commercial apps (eg, MyFitnessPal [64] or Lose It! [65]) for weight management and PA. Other articles presented details on the design; in addition, they evaluated the effects of technologies on PA behavior using uncontrolled before-and-after experiments (eg, Motivate [66,67] and BeWell [68,69]) or randomized controlled trials (eg, Fit Up [49] or Houston [70]). Several reported on the development and usability of apps for dietary interventions (eg, EatWell [71], Kalico [72], and My Meal Mate [73]). Others focused only on text messaging to support weight loss among a variety of populations and settings (eg, overweight and obese adults [74], female university staff [75], and children [76]). A total of 31 articles (31/270, 11.5%) reported exclusively on the use of Web 2.0 technologies, and mostly focused on *evaluations* (22/31, 71%) and *feasibility* (16/31, 52%). *Design and development* was reported in 8 articles (8/31, 26%). Examples include studies that reported on the development of research-developed social networking communities promoting weight loss (eg, Total Wellbeing Diet (TWD) online program [77] or Social Families (SOFA) project [78,79]), or Facebook-based weight management interventions among young adult cancer survivors [80] and adult employees with metabolic syndrome [81]. A total of 41 studies out of 270 (15.2%) combined both mobile and Web 2.0 technologies, and almost equally covered all research stages—20 (7.4%) design and development, 19 (7.0%) feasibility, and 21 (7.8%) evaluations. Examples are the ManUp study, which aimed to promote healthy eating and PA among adults using a combination of social media and mobile phone apps [82], the Pounds Off Digitally study [83], and the follow-up, Mobile Pounds Off Digitally study [84].

In terms of reviews on behavior change, 33 studies out of 49 (67%) were published in the last 2 years. A total of 4 out of 5 (80%) general narrative reviews discussed the role of mobile technologies for weight management [85-88], and 4 out of 5 (80%) scoping reviews discussed the use of mobile technologies for general health behavior change, including studies on weight management [27-29,31]. A total of 15 out of 28 (54%) qualitative syntheses on behavior change interventions also specifically focused on mobile technologies. Social media was covered in 1 narrative review on blogs to record PA and diet [55], 1 scoping review on Web 2.0 technologies used among patients and caregivers [30], 1 qualitative synthesis on weight management [40], and 5 meta-analyses on interventions promoting general health behavior change [89], weight management [90], PA promotion [37], or PA and diet [91]. A total of 14 reviews out of 49 (29%) reported on various eHealth technologies, including mobile and Web 2.0, without considering these separately: 1 (2%) nonsystematic review, 12 (25%) qualitative syntheses, and 1 (2%) meta-analysis.

### Research on Technologies for Measuring Behavior

More than half of the primary research studies dealing with behavioral assessment (55/102, 53.9%) were published since 2012. Almost all of these (100/102, 98.0%) focused on mobile technologies employed for dietary or PA assessment. Of the 102 studies, 50 (49.0%) dealt with *design and development*, 34 (33.3%) dealt with *feasibility*, and 70 (68.6%) dealt with *evaluations* of accuracy and validity of mobile technologies employed for dietary or PA assessment. Examples of studies focusing on testing accuracy in PA tracking developed systems using both old (eg, Nokia N97 [92]) and more modern devices (eg, iPhone [93], iPod Touch [94], or Android phones [95,96]). Dietary assessment technologies include those that utilize mobile phone cameras to capture images of food and keep food diaries (eg, DietCam [97]), or native mobile phone apps that allow users to manually input information about the food consumed ([98,99]). A few articles reported on the development and evaluation of the usability of systems that encompassed both PA and dietary assessment (eg, SapoFit [100,101]).

A total of 7 out of the 11 (64%) reviews focusing on technologies for behavior assessment were published since 2013 and all reported on mobile technologies. Of these 11 reviews, 9 (82%) focused on dietary assessment and 2 (18%) focused on PA assessment [52,53]. A total of 2 out of the 11 (18%) reviews were nonsystematic reviews that described various methods for technology-based food assessment, including mobile-based digital photography [102,103]. A total of 4 qualitative syntheses out of 11 (36%) reported on *evaluations* of the accuracy and validity of mobile phone apps for dietary assessment [57,104-106], and 1 (9%) reported on *feasibility* and *evaluations* of these mobile technologies [60].

### Overview of Apps and Social Media Content

The literature on mobile phone apps and Web 2.0 content is recent; almost all studies (19/21, 90%) were published in the last 2 years—7 (33%) studies in 2013 and 12 (57%) studies in 2014—and the earliest study was published in 2011 [107]. In general, most of these reviews focused on mobile phone apps for PA/fitness [108,109], and also analyzed the content of online social networks for PA promotion [110] and dietary and fitness apps [111-114]. Some examined mobile phone apps for dietary control [115-117] or weight loss [107,118]. Only 1 (5%) review and case study out of 21 reported on the use of a website with social media apps for promoting PA [119]. A total of 8 studies out of 21 (38%) investigated whether apps included constructs derived from behavioral theories [117,120] or evidence-based strategies and expert recommendations for behavior change [107,113]. A total of 5 out of 21 (24%) explicitly investigated the presence of behavioral change techniques (BCTs) in apps [108,109]. A total of 7 out of 21 studies (33%) dealt with the evaluation of *usability* principles (eg, heuristic evaluation) in apps [56,116,121] or websites with social media components [110].

Content analyses of Web 2.0 apps were published in the last 7 years, with the oldest study dating back to 2007 [55] and half of the studies (8/15, 53%) published in 2014. Of the 15 overviews of social media, 7 (47%) focused on the analysis of blog content related to weight management. For example, some studies analyzed how members of food blogging communities interact and what information they share [44,122], or how users seek emotional support when dieting [123]. Other research focused on the analysis of how social support is provided in PA-oriented online communities [124] or commercial weight-loss programs such as Weight Watchers [125]. Other studies focused on Twitter as a venue for discussion about childhood obesity [126] and about weight loss among adults [45].

## Discussion

### Principal Findings

There is an extensive body of knowledge on the use of mobile and Web 2.0 technologies for weight management. In this review we included 457 articles published in a wide range of journals and conference proceedings worldwide. The eHealth field is multidisciplinary, encompassing medical informatics, public health, computing and informatics, and health communication. The research originated mainly in the Anglo-Saxon world with a considerable number of studies from Europe, Asia, and the Middle East, showing that eHealth research is conducted on a global scale using English as the *lingua franca* for research dissemination. However, no African studies were identified and only few originated from developing areas of the world (eg, Latin America and Asia Pacific). Considering that in the first quarter of 2015, 334.4 million mobile phones were shipped worldwide (+16% compared to the previous year) [127] and that mobile phone markets have grown considerably in the past 2 years in Latin America, Africa, and Asia Pacific emerging countries [128], we expected more research and anticipate future growth in these regions. The lack of evidence from developing countries may be due to language barriers or a lack of research funds.

Half of the identified articles were published in the last 2 years. This suggests that the body of evidence is expanding rapidly, posing a challenge for reviewers who wish to synthesize this evidence. Increasing numbers of studies means that overall evidence-based conclusions may change rapidly over a short time.

### Research Themes

We categorized research into two main overarching themes: *promoting behavior change* and *measuring behavior*. Emergence of these two main themes suggests that the discipline has largely split into two distinct research streams. The review literature has specialized and focused either on the evaluation of effects on behavior or on the accuracy and validity of instruments assessing PA or dietary behaviors. Only two systematic reviews [52,53] discussed both aspects and reported on mobile technologies for PA behavior change and assessment. This is an important limitation of current research because the effects on behavior cannot be ascertained if the measures are not accurate or valid. Future research could aim to encompass both of these aspects.

We further categorized the articles according to three themes that define the different phases of research development: *design and development*, *feasibility*, and *evaluation*. Through this classification we gave equal attention and consideration to research that belongs to the area of systems design [129,130], which is often neglected in reviews that focus on the efficacy or effectiveness of interventions. To the best of our knowledge, no other reviews available on the topic have described the evidence from systems design articles. Future studies could seek to integrate the evidence from various disciplines. This scoping review also provides a map of *feasibility* studies testing the use of mobile and Web 2.0 technologies for weight management. A relatively large number of studies reported on the effectiveness of these technologies on behavior and weight-related outcomes. At the same time, a large number of studies reported on the accuracy and validity of mobile technologies for PA and dietary assessment. It was not the aim of this review to provide evidence on the effectiveness of these technologies, but we can conclude that there is a database of reviews and primary studies that report effects needing further synthesis.

### Use of Technology for Behavior Change and Assessment

Research in the domain of behavior change and assessment has focused almost exclusively on mobile devices, suggesting that future health promotion and care is mobile [131,132]. Mobile phones have evolved from just being used for sending and receiving text messages, to more advanced, interactive portable computers linked to the Internet. Information can only be exchanged through the Internet, via wireless networks, or via mobile data packages. Even mobile phone apps designed to promote behavior change use the same architectures and technological infrastructures as Internet programs, so that it is almost impossible to separate the two delivery modes completely.

Many studies described the *design and development* of mobile phone apps for behavior change and behavioral assessment, and many also evaluated them. The majority of such papers reported on evaluations of intervention effectiveness and on the validity and accuracy of technologies for behavior change. Most notably, all research on *measuring behavior* reported on use of mobile apps and mobile-based methods for dietary and PA assessment. A relatively recent subsection of the literature has focused specifically on the evaluation of mobile phone apps. This appears to be an expanding area, now including RCTs and quasi-experimental designs, thereby responding to calls for further studies [27,29].

Relatively little attention has been paid to the application and testing of social media technologies for behavior change or behavioral assessment. Primary research articles dealt exclusively with the role of Web 2.0 technologies for behavior change, but we also found many systematic reviews and meta-analyses on the topic. Compared to the only scoping review on social media for patients and caregivers [30], which included 371 studies, the number of studies we identified was low. This inconsistency can be explained by the different focus of our review (weight management) compared to the more generalized scope of the other review (general health promotion). However, it might also be due to a different definition of social media. In fact, Hamm and colleagues encompassed studies that used chat rooms and discussion forums, which represent an older type of social media available before the advent of Web 2.0 [133]. Future research should clarify social media definitions, thereby specifying the technologies under investigation. Our review shows that research into social media use for weight management has mostly focused on analyzing the content generated by users rather than on the effects of the use of the media on behavior. *User-generated content* is one of the core, basic features of Web 2.0, which appeared in 1999 [134] but became popular only after the 2004 Web 2.0 Conference [133]. Only a few years later, the first articles were published investigating how users portray themselves on social media, or investigating how they seek emotional and social support or discuss their health (eg, weight loss). These accounts are important to get a better picture on the usage of the medium, but more in-depth analyses of the effects are also required to establish whether and how social media-based interventions work.

### Strengths and Limitations

Unlike other similar scoping reviews, we included a large number of studies, including both review and primary research evidence. Except for Hamm and colleagues’ scoping review on social media [30], systematic reviews rarely use other reviews as sources of information for primary research studies. Our approach has generated a systematic, comprehensive, and detailed map of the available evidence on mobile and Web 2.0 technologies used in the domain of weight management. Another strength is the development of a data-driven categorization tool that could be used by other researchers or by journal editors or reviewers wishing to optimize the classification of the literature available in their journals.

A limitation of this study, which is common to systematic reviews in general, is the exclusion of articles not published in English. Unsurprisingly, the majority of articles identified originated in English-speaking countries. Another limitation is related to the inclusion of materials that were available in full text. Even though we searched a broad variety of databases and sources of “grey” literature (ie, conference proceedings, theses, and dissertations), we had to exclude entries that did not have, or were not freely available in, full text. This included meeting abstracts, conference abstracts, and theses and dissertations. During title and abstract screening we identified 32 theses and dissertations that were relevant to the topic, but had to be excluded (in most cases under embargo or not accessible through interlibrary loans) as it was not possible to complete the categorization process. However, we considered these as sources of information about potentially relevant studies. A third limitation is the subjectivity intrinsic to the inductive analytic approach we adopted to categorize the literature, which is common in qualitative research. However, we tried to reduce bias by testing the reliability of the charting tool within our team of reviewers.

### Conclusions

This scoping review provides a descriptive map of the literature on mobile and Web 2.0 technologies for weight management. We described and categorized 457 papers that discussed the design and development, feasibility, and evaluation of these eHealth technologies for promoting behavior change and also for measuring behavior. Even though the quality of this evidence needs to be evaluated using appropriate analytical strategies, there is an extensive evidence base that assesses the impact of technologies on behavior and weight-related outcomes, in particular by mobile phones and mobile apps. Some research focused exclusively on the analysis of the content of mobile phone apps. Limited evidence exists on social media for behavior change, but a segment of studies focused on the analysis of social media content to understand behaviors related to weight management from a broader, holistic perspective. Future research should analyze mobile phone and Web 2.0 technologies by combining the evaluation of content with design aspects, usability, feasibility, efficacy/effectiveness for behavior change, and accuracy/validity for behavior assessment. This way we could better understand how technologies influence behavior and how they can be more effectively and efficiently used in eHealth interventions.

## Appendix

Multimedia Appendix 1 Search strategies.

Multimedia Appendix 2 Charting tool.

Multimedia Appendix 3 Characteristics of included studies.

Multimedia Appendix 4 Excluded studies.

## Abbreviations

AC1: first-order agreement coefficient
BCT: behavioral change technique
BMI: body mass index
CINAHL: Cumulative Index to Nursing and Allied Health Literature
COPD: chronic obstructive pulmonary disease
ERIC: Education Resources Information Center
IEEE: Institute of Electrical and Electronics Engineers
IQR: interquartile range
MeSH: Medical Subject Headings
NIHR: National Institute for Health Research
OCLC: Online Computer Library Center
PA: physical activity
PDA: personal digital assistant
PenCLAHRC: Collaboration for Leadership in Applied Health Research and Care of the South West Peninsula
PICOS: participants, interventions, comparators, outcomes, and study design
PRISMA: Preferred Reporting Items for Systematic Reviews and Meta-Analyses
RCT: randomized controlled trial
SNSF: Swiss National Science Foundation
SOFA: Social Families
TWD: Total Wellbeing Diet
