# Supplementary material for: Mobile Phone and Web 2.0 Technologies for Weight Management: A Systematic Scoping Review
Source: J Med Internet Res. 2015 Nov 16;17(11):e259. doi: 10.2196/jmir.5129 (PMC4704945; doi:10.2196/jmir.5129)
Supplement: Multimedia Appendix 1 [file jmir_v17i11e259_app1.pdf]

Mobile Phone and Web 2.0 Technologies for Weight Management: A Systematic Scoping Review

Multimedia Appendix 1: Search strategies

Search components for basic search strings

S1: Weight management

MeSH1.1: [Obesity] or [Obesity, Morbid] or [Obesity, Abdominal] only this term
MeSH1.2: [Overweight] explode all trees

MeSH1.3: [Body Mass Index] only this term

MeSH1.4: [Body Weight] only this term

MeSH1.5: [Body Weight Changes] only this term

MeSH1.6: [Weight Loss] only this term

MeSH1.7: [Weight Gain] only this term

MeSH1.8: [Skinfold thickness] only this term

MeSH1.9: [Waist-hip ratio] only this term

MeSH1.10: [Abdominal fat] explode all trees

MeSH1.11: [Weight reduction programs] only this term

Keywords1: obes$ or (overweight$ or "over weight$" or overeat$ or "over eat$" or overfeed$ or "over feed$") or ("body mass index" or BMI) or (body near (weight or mass)) or ("body weight" adj1 change$) or (weight adj1 (chang$ or los$ or gain$ or maint$ or decreas$ or reduc$ or watch$ or control$ or manag$)) or ((skinfold and thickness$) or (("waist-hip" or "waist-to-hip") and ratio$) or (abdominal and fat$))

S1 = or /MeSH1.1-1.11 or Keywords1

S2: Diet

MeSH2.1: [Diet] expand all trees
MeSH2.2: [Diet Therapy] expand all trees
MeSH Subheading: Dietetics/education (ed), methods (mt)
MeSH2.3: [Diet Records] only this term
MeSH2.4: [Dietary Fats] expand all trees
MeSH2.5: [Fruit] explode all trees
MeSH2.6: [Vegetables] explode all trees
Keywords2: (diet or diets or dieting or dietary) or (diet$ adj2 (modific$ or therapy or intervention$ or strateg$ or program$ or management or scheme$)) or (nutrition adj2 (modific$ or therapy or intervention$ or strateg$ or program$ or management or scheme$)) or ("low calorie$" or hypocaloric or "hypo caloric" or "calorie control$") or (health$ adj1 eating) or (fasting or "modified fast$") or (fruit or vegetable$) or (high fat$ or low fat$ or fatty food$).

S2 = or /MeSH2.1-2.6 or Keywords2

S3: Physical Activity

MeSH3.1: [Exercise] explode all trees
MeSH3.2: [Physical Activity] only this term
MeSH3.3: [Physical Fitness] only this term
MeSH3.4: [Physical Exertion] only this term
MeSH3.5: ["Physical Education and Training"] explode all trees
MeSH3.6: [Sports**]** explode all trees
MeSH3.7: [Exercise Therapy] explode all trees
Keywords3: (exercis$ adj5 (train$ or physical$ or activ$)) or (exercise and (therapy or therapies or activity or activities or class$ or program$ or group$ or session$ or scheme$)) or ((exercise$ adj3 aerobic$) or aerobics) or (physical$ adj5 (fit$ or train$ or activ$ or endur$ or exertion$)) or (physical$ adj (activit$ or fit$ or exertion$)) or ("physical activity" or "physical fitness" or "physical education" or "physical therapy" or "physical inactivity") or (sedentary behavio?r) or sport$ or (walking or jogging or running or cycling) or ((lifestyle or life-style) adj3 (active or activit$ or physical$)).
S3 = or /MeSH3.1-3.6 or Keywords3

S4: Smartphones & mHealth

MeSH4.1: [Cellular Phone] expand all trees
MeSH4.2: [Computers, Handheld] only this term
MeSH4.3: [Mobile applications] only this term
MeSH4.3: [MP3-Player] only this term
Keywords4.1: (smartphone$ or smart-phone$ or "smart-phone$" or "smart phone$") or ((smart or cell$ or mobile) adj phone$) or ("cellular phone$" or "cellphone$" or "mobile phone$" or "mobile telephone$") or ("smartphone$ app?" or "mobile app?") or ((app? or application?) adj2 (smartphone$ or smart-phone$ or "smart-phone$" or "smart phone$" or "mobile$" or phone$ or "cellular phone$" or "cellphone$" or "cell phone$")) or
Keywords4.2: (Android adj5 (smartphone$ or smart-phone$ or "smart-phone$" or "smart phone$" or "mobile$" or phone$ or "cellular phone$" or "cellphone$" or "cell phone$" or "mobile phone$" or "mobile telephone$" or app? or application?)) or ("Android Operating System$" or "Android OS" or "Google Android" or (Android adj3 Google)) or (iOS adj5 (smartphone$ or smart-phone$ or "smart-phone$" or "smart phone$" or "mobile$" or phone$ or "cellular phone$" or "cellphone$" or "cell phone$" or "mobile phone$" or "mobile telephone$" or app? or application?)) or ("Apple iPhone OS" or "Apple iOS" or "iPhone operative system$" or (iOS adj3 phone$)) or (("Windows mobile") adj5 (smartphone$ or smart-phone$ or "smart-phone$" or "smart phone$" or "mobile$" or phone$ or "cellular phone$" or "cellphone$" or "cell phone$" or "mobile phone$" or "mobile telephone$" or app? or application?)) or ((Nokia or Samsung or BlackBerry) adj5 (smartphone$ or smart-phone$ or "smart-phone$" or "smart phone$" or "mobile$" or phone$ or "cellular phone$" or "cellphone$" or "cell phone$" or "mobile phone$" or "mobile telephone$" or app? or application?)) or ("Android Market$" or "Apple App Store$" or "App Store$" or "iTunes" or "BlackBerry App World" or "BlackBerry World" or "Ovi Store" or "Nokia Ovi Store" or "Google Play" or "Windows Phone Apps+Games") or (iPhone$ or iPod$ or "iPod Touch" or iPad$ or "i-Phone$" or "i-Pod$" or "i-Pad$") or (iPhone$ or iPod$ or "iPod Touch" or iPad$)
Keywords4.3: (mhealth or "m-health" or mHealth or "m-Health")
MeSH4.4: [Telemedicine] only this term
Keywords4.4: (telemed$ or tele-med$ or telehealth$ or tele-health$ or "mobile health")
Keywords4.5: (ehealth or "e-health" or eHealth or "e-Health)

S4 = (or/MeSH4.1-3 or Keywords4.1-2) or Keywords4.3 or (Keywords4.3 and (MeSH4.4 or Keywords4.4 or Keywords4.5)

S5: Social Media

MeSH5.1: [Social Media] explode all trees
MeSH5.2: [Blogging] explode all trees
Keywords5: ("web 2.0" or "web2.0" or "social media" or "social software" or "social web" or "social media app$" or "social media technolog$" or "social media platform$")

("social networking sit$" or "social networking websit$" or "social networking communit$" or "digital social network$" or "online social network$")

(Twitter or tweet$)

Facebook or (blog$ or vlog$ or blogging or vlogging) or "RSS feed$" or podcast$.

S5 = or/ MeSH5.1-2 or Keywords5

Final search strategy: (S1 or S2 or S3) and (S4 or S5)

Basic keyword search string

(obese or obesity or overweight or "body mass index" or "body mass indices" or "body mass indexes" or BMI or "waist-hip ratio" or "waist-to-hip ratio" or "weight gain" or "weight loss" or sports or "physical activity" or "exercise" or "walking" or "diet") AND (smartphone OR smart-phone OR "smart phone" OR "cellular phone" OR cellphone OR "mobile phone" OR "mobile telephone" OR "smartphone app*" OR "mobile app*" OR handheld OR hand-held OR PDA OR Android OR iOS OR BlackBerry OR Nokia OR Symbian OR iPhone OR iPod OR iPad OR mp3 OR "web 2.0" OR "web2.0" OR "social media" OR "social software" OR "social web" OR "social networking" OR Twitter OR tweet OR Facebook OR blog OR "RSS feed" OR podcast)

Example of Search Strategy

Medline (OvidSP)

MeSH = Medical subject heading (Medline medical index term); exp = exploded MeSH (Ovid); the dollar sign ($) or the asterisk (*) stand for any character(s); the question mark (?) substitutes one or no characters; tw = text word, including title, abstract and keyword; pt = publication type; adjX = X adjacent terms.

#	Searches
1	Obesity/ or Obesity, Abdominal/ or Obesity, Morbid/
2	exp Overweight/
3	Body Mass Index/

4	Body Weight/
5	Body Weight Changes/
6	Weight Loss/
7	Weight Gain/
8	Skinfold Thickness/
9	Waist-hip ratio/
10	exp Abdominal fat/
11	Weight Reduction Programs/
12	1 or 2 or 3 or 4 or 5 or 6 or 7 or 8 or 9 or 10 or 11
13	obes$.tw.
14	(overweight$ or "over weight$" or overeat$ or "over eat$" or overfeed$ or "over feed$").tw.
15	("body mass index" or BMI).tw.
16	(body adj (weight or mass)).tw.
17	(weight adj1 (chang$ or los$ or gain$ or maint$ or decreas$ or reduc$ or watch$ or control$ or manag$)).tw.
18	(skinfold adj1 thickness$).tw.
19	(("waist-hip" or "waist-to-hip") and ratio$).tw.
20	(abdominal and fat$).tw.
21	13 or 14 or 15 or 16 or 17 or 18 or 19 or 20
22	12 or 21
23	exp Diet/
24	exp Diet Therapy/
25	Dietetics/ed, mt
26	Diet Records/
27	exp Dietary Fats/
28	exp Fruit/
29	exp Vegetables/
30	23 or 24 or 25 or 26 or 27 or 28 or 29
31	(diet or diets or dieting or dietary).tw.
32	(diet$ adj2 (modific$ or therapy or intervention$ or strateg$ or program$ or management or scheme$)).tw.
33	(nutrition adj2 (modific$ or therapy or intervention$ or strateg$ or program$ or management or scheme$)).tw.
34	("low calorie$" or hypocaloric or "hypo caloric" or "calorie control$").tw.
35	(health$ adj1 eating).tw.
36	(fasting or "modified fast$").tw.
37	(fruit or vegetable$).tw.
38	("high fat$" or "low fat$" or "fatty food$").tw.
39	31 or 32 or 33 or 34 or 35 or 36 or 37 or 38
40	30 or 39
41	exp Exercise/
42	Physical Activity/
43	Physical Fitness/
44	Physical Exertion/
45	exp "Physical Education and Training"/
46	exp Sports/
47	exp Exercise Therapy/
48	41 or 42 or 43 or 44 or 45 or 46 or 47
49	(exercis$ adj5 (train$ or physical$ or activ$)).tw.
50	(exercise and (therapy or therapies or activity or activities or class$ or program$ or group$ or session$ or scheme$)).tw.
51	((exercise$ adj3 aerobic$) or aerobics).tw.
52	(physical$ adj5 (fit$ or train$ or activ$ or endur$ or exertion$)).tw.
53	(physical$ adj (activit$ or fit$ or exertion$)).tw.
54	physical education.tw.
55	("physical activity" or "physical fitness" or "physical education").tw.
56	("physical activity" or "physical fitness" or "physical education" or "physical therapy" or "physical inactivity").tw.
57	(fitness adj3 (class$ or regime$ or program$)).tw.
58	sedentary behavio?r.tw.
59	sport$.tw.
60	(walking or jogging or running or cycling).tw.
61	((lifestyle or life-style) adj3 (active or activit$ or physical$)).tw.
62	49 or 50 or 51 or 52 or 53 or 54 or 55 or 56 or 57 or 58 or 59 or 60 or 61
63	48 or 62
64	exp Cellular Phone/
65	Computers, Handheld/
66	Mobile applications/
67	MP3-Player/
68	64 or 65 or 66 or 67
69	(smartphone$ or smart-phone$ or "smart-phone$" or "smart phone$").tw.
70	((smart or cell$ or mobile) adj phone$).tw.
71	("cellular phone$" or "cellphone$" or "mobile phone$" or "mobile telephone$").tw.
72	("smartphone$ app?" or "mobile app?" or "smartphone$ application?" or "mobile application? ").tw.
73	((app? or application?) adj2 (smartphone$ or smart-phone$ or "smart-phone$" or "smart phone$" or "mobile$" or phone$ or "cellular phone$" or "cellphone$" or "cell phone$" or "mobile phone$" or "mobile telephone$")).tw.

74	(Android adj5 (smartphone$ or smart-phone$ or "smart-phone$" or "smart phone$" or "mobile$" or phone$ or "cellular phone$" or "cellphone$" or "cell phone$" or "mobile phone$" or "mobile telephone$" or app? or application?)).tw.
75	("Android Operating System$" or "Android OS" or "Google Android" or (Android adj3 Google)).tw.
76	(iOS adj5 (smartphone$ or smart-phone$ or "smart-phone$" or "smart phone$" or "mobile$" or phone$ or "cellular phone$" or "cellphone$" or "cell phone$" or "mobile phone$" or "mobile telephone$" or app? or application?)).tw.
77	("Apple iPhone OS" or "Apple iOS" or "iPhone operative system*" or (iOS adj3 phone$)).tw.
78	("Windows mobile" adj5 (smartphone$ or smart-phone$ or "smart-phone$" or "smart phone$" or "mobile$" or phone$ or "cellular phone$" or "cellphone$" or "cell phone$" or "mobile phone$" or "mobile telephone$" or app? or application?)).tw.
79	((Nokia or Samsung or BlackBerry) adj5 (smartphone$ or smart-phone$ or "smart-phone$" or "smart phone$" or "mobile$" or phone$ or "cellular phone$" or "cellphone$" or "cell phone$" or "mobile phone$" or "mobile telephone$" or app? or application?)).tw.
80	("Android Market$" or "Apple App Store$" or "App Store$" or "iTunes" or "BlackBerry App World" or "BlackBerry World" or "Ovi Store" or "Nokia Ovi Store" or "Google Play" or "Windows Phone Apps+Games").tw.
81	(iPhone$ or iPod$ or "iPod Touch" or iPad$ or "i-Phone$" or "i-Pod$" or "i-Pad$").tw.
82	69 or 70 or 71 or 72 or 73 or 74 or 75 or 76 or 77 or 78 or 79 or 80 or 81
83	(mhealth or "m-health" or mHealth or "m-Health").tw.
84	exp Telemedicine/
85	(telemedic$ or tele-medic$ or telehealth$ or tele-health$ or "mobile health$").tw.
86	(ehealth or "e-health" or eHealth or "e-Health").tw.
87	82 and (84 or 85 or 86)
88	87 or 83 or 82
89	Social Media/
90	Blogging/
91	89 or 90
92	("web 2.0" or "web2.0" or "social media" or "social software" or "social web" or "social media app*" or "social media technolog*" or "social media platform*").tw.
93	("social networking sit*" or "social networking websit*" or "social networking communit*" or "digital social network*" or "online social network*").tw.
94	(Twitter or tweet*).tw.
95	Facebook.tw.
96	(blog* or vlog* or blogging or vlogging).tw.
97	RSS feed*.tw.
98	podcast*.tw.
99	92 or 93 or 94 or 95 or 96 or 97 or 98
100	91 or 99
101	22 or 40 or 63
102	88 or 100
103	101 and 102
104	limit 103 to (english language and english)
105	limit 104 to (yr="2004 - 2014")
