# Supplementary material for: Mobile Phone and Web 2.0 Technologies for Weight Management: A Systematic Scoping Review
Source: J Med Internet Res. 2015 Nov 16;17(11):e259. doi: 10.2196/jmir.5129 (PMC4704945; doi:10.2196/jmir.5129)
Supplement: Multimedia Appendix 2 [file jmir_v17i11e259_app2.pdf]

Mobile Phone and Web 2.0 Technologies for Weight Management: A Systematic Scoping Review
Multimedia Appendix 2 – Codebook and categorisation tool

Procedure

General categorisation of the literature (i.e., "type of study")

Please adhere to the following procedure to categorise the literature:
1) Read the title, abstract and full-text of the paper where necessary to identify the: 1) Purpose/Objective of the paper, 2) Measures, outcomes, and other methods of assessment (e.g. qualitative interviews) used 3) Results data actually reported in the paper.

2) Decide the focus of the paper: Does it report data on the analysis of mobile devices and Web 2.0 tools primarily for A) **promoting behaviour change/influencing behaviour** or for B) **measuring behaviour (i.e., physical activity or diet)**, or C) other (see below)? In some cases, the articles may report data that pertain to both these aspects. In such cases, the article must categorised as both A and B.

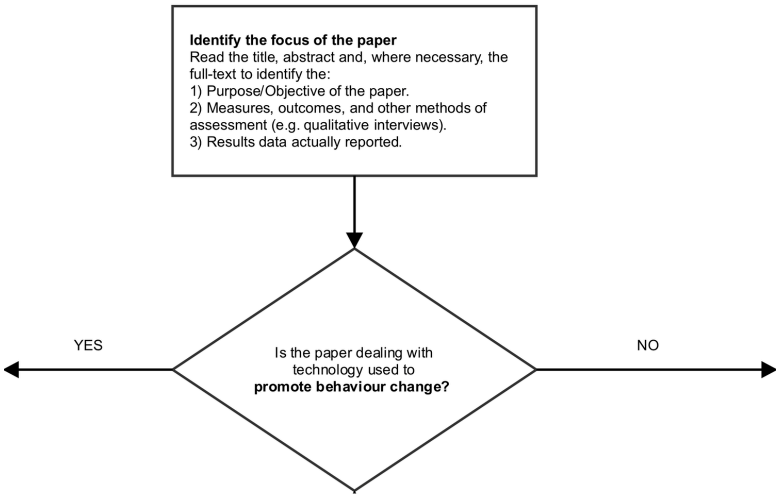

3) Categorise the paper into one of the following categories by attributing the relevant code. In EndNote, append the code in the Custom 4 or Custom 5 fields; in Excel, create a new column next to the reference and add the code there.

Notes
A) "Promoting behaviour change/Influencing behaviour". This category includes articles that discuss the use of mobile and web 2.0 technologies with the aim to achieve a modification or a change in the weight-related behaviours such as weight control, physical activity or diet. Technology in this sense is used as delivery mode for an intervention that promotes behaviour change, e.g., through self-monitoring, providing feedback, reminding about the behaviour or motivating it. These articles report data and results that reflect effects on behaviour, weight-related outcomes or socio-cognitive predictors of behaviour and of weight-related outcomes.

B) "Measures of behaviour": This category includes articles that specifically focus on the development, test and evaluation of technologies for assessing physical activity or dietary behaviours, without reporting data on their effects on actual behaviour. The reported outcome data include elements associated with the accuracy or validity of apps or systems for physical activity and dietary assessment (e.g., activity recognition, energy expenditure estimation, activity classification, food classification and estimation, comparison between self-reported, paper and pencil or objective measures of behaviour using the technologies).

The information contained in the article's abstract, background or introduction sections should help define the scope of the paper and might clearly indicate whether the article pertains to the category "Promoting behaviour change" or "Measuring behaviour". However, if this does not occur, the sections Methodology and Results may bring more clarity and help identify the core focus of the paper. If the paper clearly reports both aspects, it has to be labelled as an overlap, including the labels of the categories indicated below.

The categorisation process is summarised in a **flowchart**, which is represented in the following pages as screenshots. The full, high-quality version of the flowchart is available from the following link: http://www.gliffy.com/go/publish/7672495.

PROCEDURE **1**
GENERAL CATEGORISATION OF THE LITERATURE (I.E., "TYPE OF STUDY") 1
1) BEHAVIOUR CHANGE (REVIEWS) **3**
1.1 GENERAL REVIEW 4
1.2 REVIEW OF EVALUATIONS OR OF EXISTING REVIEWS 4
1.3 REVIEW ABOUT FEASIBILITY AND EFFICACY/ EFFECTIVENESS OF INTERVENTIONS 4
2) BEHAVIOUR CHANGE (PRIMARY RESEARCH ARTICLES) **5**
2.1 SYSTEM DESIGN STUDY 6
2.2 FEASIBILITY STUDY 6
2.3 EFFICACY/EFFECTIVENESS EVALUATION STUDY 7
2.4 PROCESS/OUTCOME EVALUATIONS, OR CAUSAL-COMPARATIVE STUDIES 7
2.5 SYSTEM DESIGN AND USABILITY 8
2.6 SYSTEM DESIGN AND EFFICACY/EFFECTIVENESS 8
2.7 SYSTEM DESIGN USABILITY, FEASIBILITY AND EFFICACY/EFFECTIVENESS 8
2.8 FEASIBILITY AND EFFICACY EVALUATION STUDY 9
3) MEASURES OF BEHAVIOUR (REVIEWS) **10**
3.1 GENERAL REVIEW 11
3.2 REVIEW ABOUT ACCURACY/VALIDITY 11
3.3 REVIEW ABOUT ACCURACY/VALIDITY AND FEASIBILITY 11
4) MEASURES OF BEHAVIOUR (PRIMARY RESEARCH ARTICLES) **12**
4.1 SYSTEM DESIGN 13
4.2 FEASIBILITY 13
4.3 ACCURACY/VALIDITY 13
4.4 SYSTEM DESIGN AND USABILITY 14
4.5 SYSTEM DESIGN AND ACCURACY/VALIDITY STUDY 14
4.6 SYSTEM DESIGN AND USABILITY, AND FEASIBILITY AND ACCURACY/VALIDITY STUDY 14
4.7 FEASIBILITY AND ACCURACY STUDY 14
5) OVERVIEWS OF APPS AND THEIR CONTENT **15**
5.1 REVIEWS OF APP CONTENT 15
6) DESCRIPTIONS AND ANALYSES OF SOCIAL MEDIA **15**
6.1 REVIEWS OF SOCIAL MEDIA CONTENT 15

1) Behaviour change (Reviews)

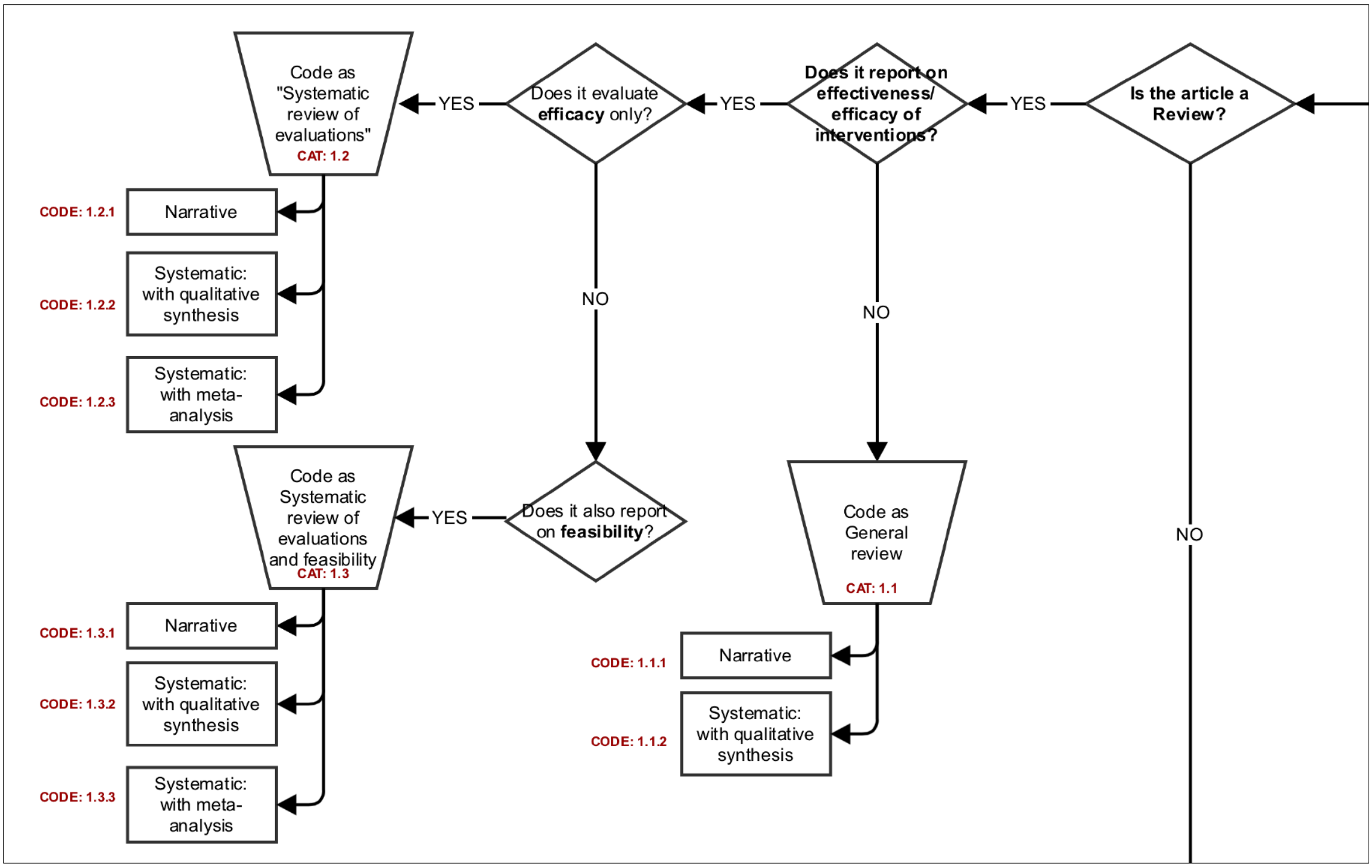

Label	Definition	Examples of study purpose
**1.1 General review**<br><br>**1.1.1 Narrative**<br>**1.1.2 Systematic**	The paper is a general **review** about eHealth/mHealth/Web 2.0 technologies for behaviour change, **which does not report any synthesis of evaluation results**. The units of analysis of the review are studies that used technologies for used to influence behaviour.<br><br>*Note: A narrative review does not contain information on how the reviewed interventions/paper have been retrieved, included and reviewed. A systematic review includes a methodology section that explicitly defines the sources of information chosen, the search strategy, the inclusion/exclusion criteria, etc.*<br><br>If the review is systematic and reports a synthesis of evaluation of results, categorise as: **1.2** or as **1.3**.	"The purpose of this review is to discuss studies in which digital technology has been used for behavioural weight control, report on advances in consumer technology that are widely adopted but insufficiently tested, and explore potential future directions for both."<br><br>"The study aims to determine how health behavior theories are applied to mobile interventions. **This is a review of the theoretical basis and interactivity** of mobile health behavior interventions. […] In contrast to meta-analytic reviews of effect size, the purpose of this review is to assess how theory has been employed in the development of mobile health behavior interventions […]."
**1.2 Review of evaluations or of existing reviews**<br><br>**1.2.1 Narrative**<br>**1.2.2 Qualitative synthesis**<br>**1.2.3 Meta-analysis**	The article is a **systematic review** (or a review of reviews) about the efficacy or effectiveness of eHealth/mHealth/Web 2.0 for behaviour change.<br><br>*Note: A systematic review includes a methodology section that explicitly defines the sources of information chosen, the search strategy, the inclusion/exclusion criteria, etc. A meta-analysis is a systematic review that comprises statistical methods for summarising and combining results from different studies.*<br><br>If the review is systematic but does not report a synthesis of evaluation of results, categorise as: **1.1**.	"The purpose of the present study was to perform a systematic review and meta-analysis of RCTs reporting the use of mobile electronic devices in weight loss efforts among overweight and obese adult population."<br><br>"This article will focus on recent research looking at the effectiveness of text messaging as a health intervention eliciting a positive behavioural response among adolescents and young adults in the management of chronic disease and unhealthy behaviours."
**1.3 Review about feasibility AND efficacy/ effectiveness of interventions**<br><br>**1.3.1 Narrative**<br>**1.3.2 Qualitative synthesis**<br>**1.3.3 Meta-analysis**	The paper presents **a review** focusing on the evaluation of **feasibility** and **efficacy** elements of interventions. To be included in this category, the review needs to have reviewed outcomes relevant to feasibility (e.g. usability, acceptability) as well as outcomes.<br><br>*Note: **Feasibility studies** are in general pieces of research done before a main study in order to answer the question "Can this study be done?". They are used to estimate important parameters that are needed to design the main study such as: standard deviation of the outcome measure, which is needed in some cases to estimate sample size; willingness of participants to be randomised; willingness of clinicians to recruit participants; recruitment and assessment procedures, etc. [1]*	"The present study analyses the feasibility and efficacy of text-messaging-delivered interventions in clinical outcomes and healthy behaviour modifications."

2) Behaviour change (Primary research articles)

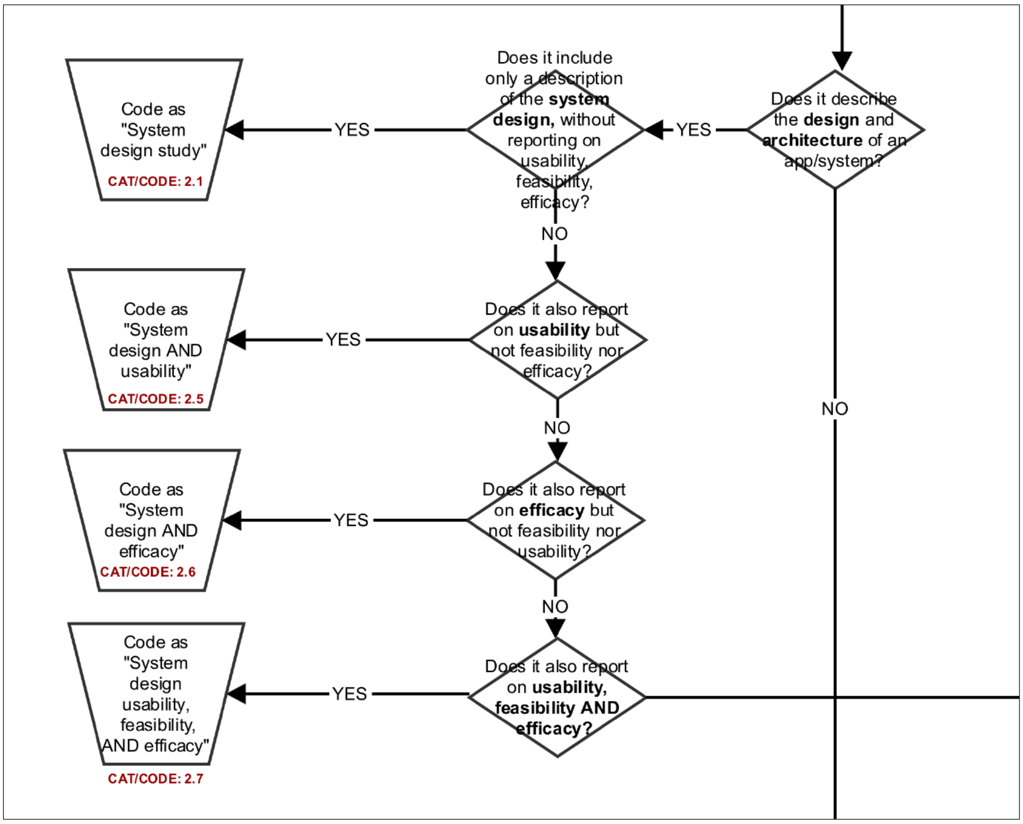

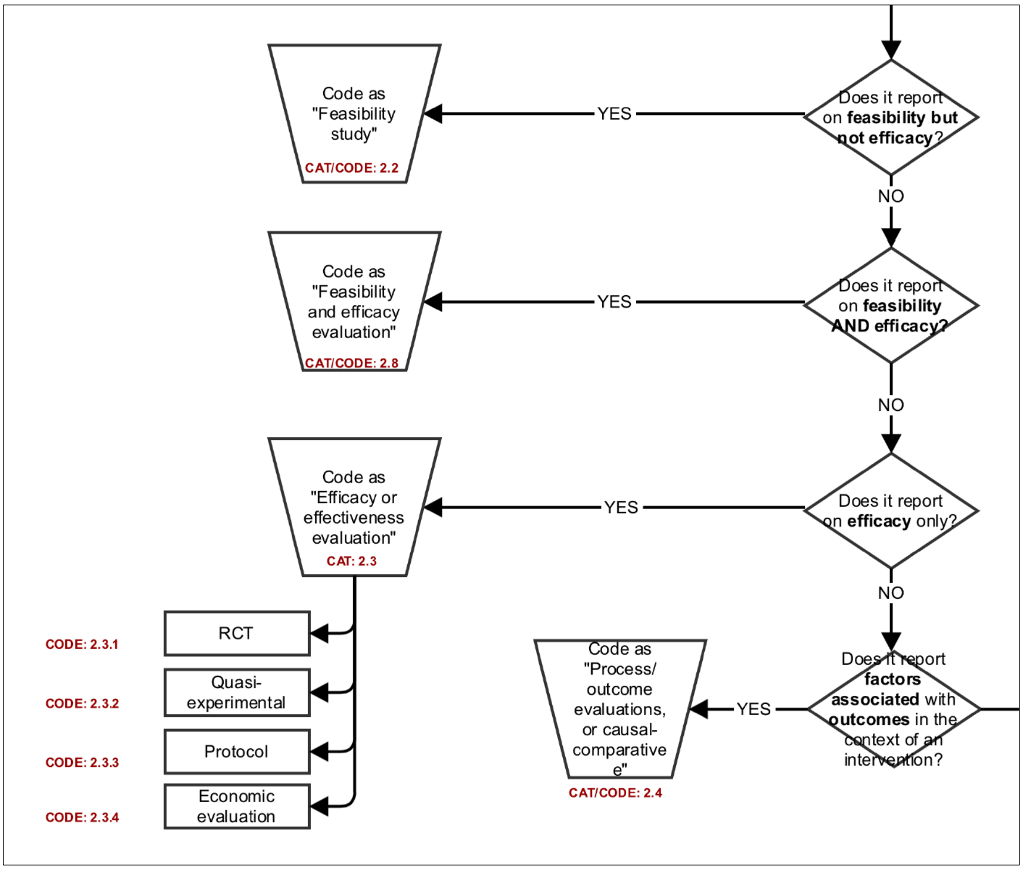

Label	Definition	Examples of study purpose
**2.1 System design study**	The paper **describes the design** and architecture of a system/app for influencing behaviour, ***without* reporting any kind of evaluation** or results.<br><br>*Note: System design is the activity of proceeding from an identified set of requirements for a system to a design that meets those requirements. A distinction is sometimes drawn between high-level or architectural design, which is concerned with the main components of the system and their roles and interrelationships, and detailed design, which is concerned with the internal structure and operation of individual components.[2]*<br><br>If the paper reports also results in terms of usability and feasibility, code as: **2.5**.<br><br>If it also reports on efficacy/effectiveness, code as: **2.6**.<br><br>If it also reports on feasibility usability AND efficacy/effectiveness, code as: **2.7**.	"This article explains the conceptual design process for a mobile application that seeks to persuade people to make behavioural changes. Current applications in this market are good but are not oriented to persuasion. The article introduces the philosophy and principles behind the application and describes an early conceptual prototype design."
**2.2 Feasibility study**	The article describes the results of an actual, existing **pilot/feasibility study**. It might include formative research and development of an existing app/intervention. It focuses on outcomes such as: acceptability, participation, utilisation, retention and recruitment rates, adherence or compliance to processes and procedures. It **does not report an evaluation of effects** on behaviour and/or weight-related outcomes.<br><br>*Note: **Feasibility studies** are in general pieces of research done before a main study in order to answer the question "Can this study be done?". They are used to estimate important parameters that are needed to design the main study such as: standard deviation of the outcome measure, which is needed in some cases to estimate sample size; willingness of participants to be randomised; willingness of clinicians to recruit participants; recruitment and assessment procedures, etc. [1]*<br><br>*Note: Pilot studies are a smaller version of the main study used to test whether the components of the main study can all work together. They are generally focused on the processes of the main study, for example to ensure that recruitment, randomisation, treatment, and follow-up assessments all run smoothly. They resemble the main study in many respects, including an assessment of the primary outcome.[1]*<br><br>*Note: Some studies might refer to usability as feasibility, depending on the discipline (i.e., computer sciences, health sciences or behavioural sciences). Usability is "the extent to which a product can be used to achieve specified goals with effectiveness, efficiency, and satisfaction in a specified context of use. The word "usability" also refers to methods for improving ease-of-use, learnability, task-efficiency, memorability, ease-of-use, satisfaction, and usefulness".*[3] *(see **2.5** below for definitions and examples of this type of papers).*<br><br>If the paper reports on system design AND reports on usability measures, categorise as: **2.5**.<br><br>If it also reports on efficacy/effectiveness it needs to be categorised as: **2.8**.<br><br>If it reports on feasibility, usability AND efficacy/effectiveness, code as: **2.7**.	"The purpose of the current study is to report the **feasibility** of a Twitter-based core-strengthening exercise challenge by reporting the number and characteristics of participants, the frequency of participation, the extent to which participants spread the challenge to others, and the extent to which participants experience social support from online friends relative to in-person friends or family."<br><br>"The objective of the present study was to test the **feasibility and acceptability** of a computerized system to send tailored messages to the mobile phones of obese adolescents enrolled in the Michigan Pediatric Outpatient Weight Evaluation and Reduction (MPOWER) programme."

Label	Definition	Examples of study purpose
**2.3 Efficacy/effectiveness evaluation study**<br><br>**2.3.1) RCT**<br>**2.3.2) Quasi-experimental**<br>**2.3.3) Protocol**<br>**2.3.4) Economic evaluation**	The article **reports on the evaluation** of an intervention using a randomised controlled clinical trial (RCT) or a quasi-experimental design (e.g. non-randomised trials, before-after studies). The article mainly focuses on describing the **effects of the intervention on the study outcomes**, such as behaviour (i.e., PA or diet) or weight-related outcomes.<br><br>*Note: The study design might not be clearly mentioned in the abstract or title, but will likely be explained in the methods section. Efficacy studies investigate the benefits and harms of an intervention under highly controlled conditions. Effectiveness studies (also known as pragmatic studies) examine interventions under circumstances that more closely approach real-world practice, with more heterogeneous patient populations, less-standardized treatment protocols, and delivery in routine clinical settings. [4]*<br><br>If the paper presents a **study protocol** and rationale for a prospective intervention, code as "protocol" (2.3.3). A protocol does not include any efficacy results, but may include initial information about participant recruitment and baseline sample characteristics.<br><br>If the paper presents an **economic evaluation** code as "economic evaluation" (2.3.4). Economic evaluation studies report on the costs or **cost-effectiveness** of one or more interventions, and it may also include separate results on effectiveness.<br>*Note: Cost-effectiveness analysis (CEA) is a form of economic analysis that compares the relative costs and outcomes (effects) of two or more courses of action. [...] Typically the CEA is expressed in terms of a ratio where the denominator is a gain in health from a measure (years of life, premature births averted, sight-years gained) and the numerator is the cost associated with the health gain. The most commonly used outcome measure is quality-adjusted life years (QALY).*<br><br>If it also reports on the development of a system or system design, code as: **2.6**.<br><br>If it also reports also on feasibility outcomes, categorise as **2.8**.<br><br>If it reports on feasibility, usability AND efficacy/effectiveness, code as: **2.7**.<br><br>If the article describes process and outcome evaluation measures or reports on secondary data analyses of other factors associated with an intervention (e.g., social cognitive determinants of behavior), without reporting effects on behavioural or weight-related outcomes, code as: **2.4** (see below).	"This paper reports the final 24-month outcomes of a randomized controlled trial evaluating the effect of additional therapeutic contact (ATC) as an adjunct to a community-based weight-management program for overweight and obese 13–16-year-olds."<br><br>"The present study evaluated whether an intervention using the SMS by cellular phone and Internet would affect the levels of blood pressure, body weight, waist circumference, and serum lipids of the patients with obese hypertension."<br><br>"The aim of this study was to make a cost-utility analysis of a dietary behavior modification treatment, compared to usual care, among lactating overweight and obese women."<br><br>"The aim of this study is to assess the cost-effectiveness of e-learning devices as a method of promoting weight loss via dietary change."
**2.4 Process/outcome evaluations, or causal-comparative studies**	The article reports a study where the main focus is on examining socio-cognitive, technological, or other **factors associated with outcomes** in the context of existing interventions, or effectiveness studies. It might include **secondary data analyses** which may use data from existing RCTs/effectiveness studies, but do not have effectiveness as their main focus.<br><br>If the article describes process and outcome evaluation measures associated with effects on behaviour or weight-related outcomes. In this case it should be coded as "efficacy study" **2.3**.	"The objectives of this study were to (1) characterize how pregnant women engaged with features of an online intervention to prevent excessive gestational weight gain, (2) identify demographic and weight status subgroups of women within the sample, and (3) examine differences in use of intervention features across the demographic and weight status subgroups."

Label	Definition	Examples of study purpose
**2.5 System design AND usability**	The paper **<u>describes the design</u>** and architecture of a system/app that is aimed at influencing behaviour, AND reports on **<u>usability of the system</u>** (i.e., technical feasibility).<br><br>*Note: Usability is the extent to which a product can be used to achieve specified goals with effectiveness, efficiency, and satisfaction in a specified context of use. The word "usability" also refers to methods for improving ease-of-use, learnability, task-efficiency, memorability, ease-of-use, satisfaction, and usefulness.*[3] *In this case, usability can be interpreted as "technical feasibility". Feasibility studies are in general pieces of research done before a main study in order to answer the question "Can this study be done?". They are used to estimate important parameters that are needed to design the main study.*[1]<br><br>*Usability and feasibility measures might not be clear from the abstract of the paper, but they should be mentioned in the methodology and results section.*<br><br>If it reports on efficacy/effectiveness but does <u>not</u> include feasibility measures, code as: **2.6**.<br><br>If it reports on feasibility, usability AND efficacy/effectiveness, code as: **2.7**.	"In this paper, we aim to provide a complete physical activity system with continual motivation through our exergaming model, developed into an application called World of Workout. This game provides a large selection of exercises using sensors widely available in smartphones, allowing physical activity recording on a wide selection of devices. We also provide several channels of feedback for both long and short-term drive, which encourages users to exercise regularly."<br><br>"The aim of the present study was to evaluate the usability and feasibility of a mobile phone program in recording of symptoms during a physical exercise trial."
**2.6 System design AND efficacy/effectiveness**	The paper **<u>describes the design</u>** and architecture of a system/app for behaviour change and report results on **<u>effects</u>** on behaviour and/or weight-related outcomes.<br><br>If the paper does <u>not</u> present an efficacy evaluation, it needs to categorised as: **2.1**.<br><br>If the paper presents results on usability and feasibility, but <u>not</u> efficacy/effectiveness, categorise as: **2.5**.<br><br>If it reports on feasibility, usability AND efficacy/effectiveness, code as: **2.7**.	"Our current investigation examines the long-term effectiveness of using a stylized representation of behavior on a personal, mobile display to encourage regular and varied physical activity. We conducted a three-month field experiment of our system."<br><br>"This multidisciplinary paper reports on a large-scale field trial, designed and implemented by a group of social scientists, computer scientists and statisticians, of a new smartphone-based app for the promotion of walking in everyday life."
**2.7 System design usability, feasibility AND efficacy/effectiveness**	The paper **<u>describes the system design</u>** and reports on **<u>feasibility</u>**, **<u>usability</u>** AND **<u>efficacy</u>** in terms of behaviour and/or weight-related outcomes.<br><br>*Note: The study should encompass all three elements to be included in this category; the methodology and results sections should include the measures about feasibility, usability and efficacy.*<br><br>If the paper does <u>not</u> present an efficacy evaluation, or results on feasibility, code as: **2.1**.<br><br>If the paper presents results in terms of usability and feasibility, but not efficacy/effectiveness, categorise as: **2.5**.	"We describe Xxxxxx, a prototype mobile phone application **for encouraging activity** by sharing step count with friends. We also present four **design requirements** for technologies that encourage physical activity that we derived from a three-week long in situ **pilot study**"<br><br>"We embarked on a **live clinical trial of a behavior** based mobile application designed to assist users on meal replacement diet programs to judge its impact and value. We conducted a **user study** aimed at evaluating the **effectiveness** of the WMM prototype for the meal replacement program participants and its **impact on user engagement** with the diet and weight loss."

Label	Definition	Examples of study purpose
**2.8 Feasibility AND efficacy evaluation study**	The paper **describes a pilot/feasibility study**, and reports results about its **efficacy** in terms of behaviour change or weight-related outcomes.<br><br>*Note: **Feasibility studies** are in general pieces of research done before a main study in order to answer the question "Can this study be done?". They are used to estimate important parameters that are needed to design the main study such as: standard deviation of the outcome measure, which is needed in some cases to estimate sample size; willingness of participants to be randomised; willingness of clinicians to recruit participants; recruitment and assessment procedures, etc. [1]*<br><br>*Note: Pilot studies are a smaller version of the main study used to test whether the components of the main study can all work together. They are generally focused on the processes of the main study, for example to ensure that recruitment, randomisation, treatment, and follow-up assessments all run smoothly. They resemble the main study in many respects, including an assessment of the primary outcome.[1]*	"The major goal of this pilot study was to evaluate the feasibility, acceptability, and preliminary efficacy of theoretically based behavioural interventions delivered by smartphone technology."<br><br>"The aim of this pilot study was to collect acceptability and feasibility outcomes of a self-monitoring weight management intervention delivered by a smartphone app, compared to a website and paper diary."

3) Measures of behaviour (Reviews)

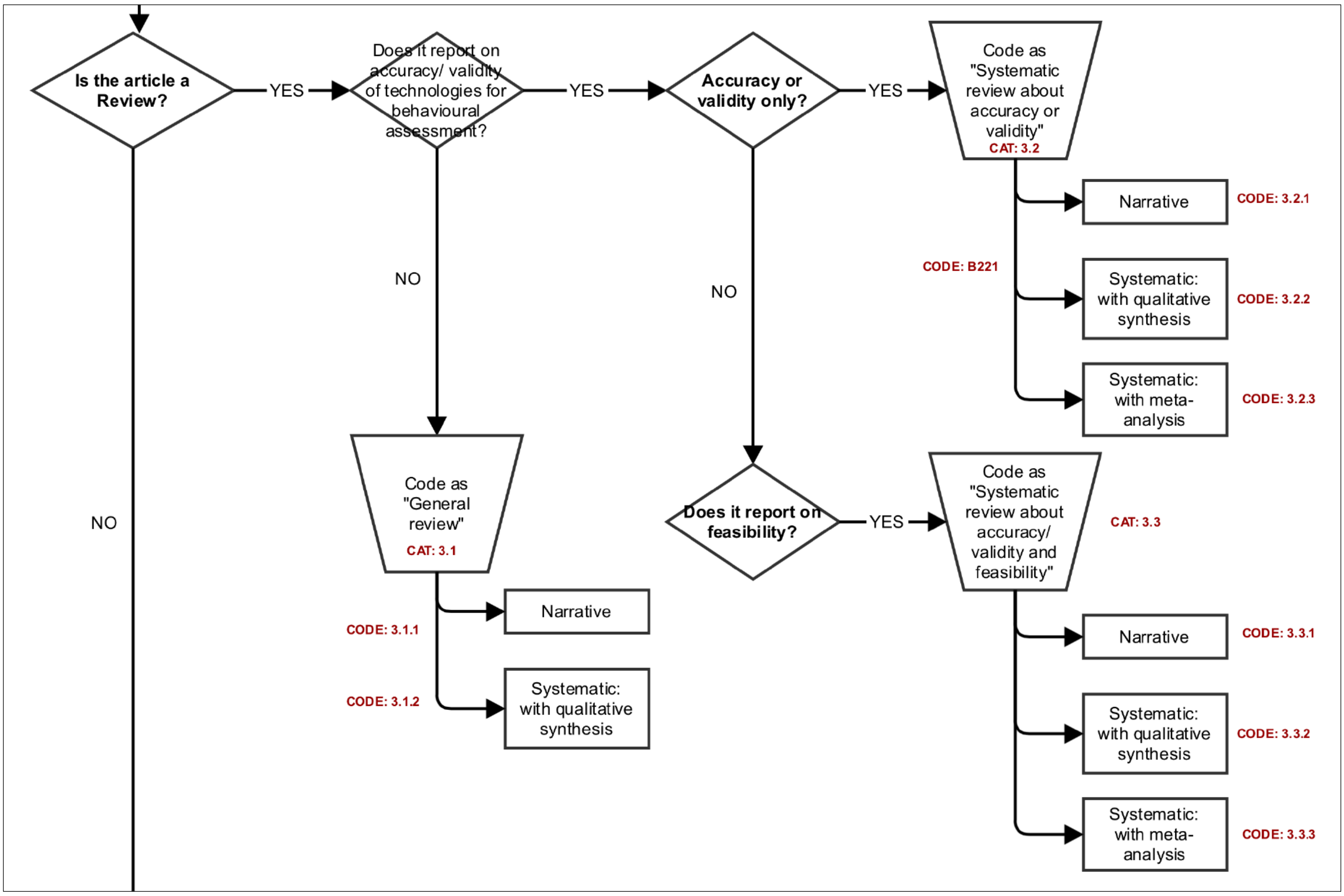

Label	Definition	Examples of study purpose
**3.1 General review**<br><br>**3.1.1 Narrative**<br>**3.1.2 Systematic**	The paper is a **review** about mHealth for measuring behaviour in general, without discussing accuracy/validity of the measurement tools.<br><br>*Note: A narrative review does not contain information on how the reviewed interventions/paper have been retrieved, included and reviewed.*<br>*A systematic review includes a methodology section that explicitly defines the sources of information chosen, the search strategy, the inclusion/exclusion criteria, etc.*	"The objective of this paper was to summarize existing approaches to self-management of food intake recording and to analyse the functionalities and input methods."
**3.2 Review about accuracy/validity**<br><br>**3.2.1 Narrative**<br>**3.2.2 Systematic**<br>**3.2.3 Meta-analysis**	The paper is a **review** of the evidence about **accuracy**/**validity** of technology-based behavioural assessment techniques and methods.<br><br>*Note: A narrative review does not contain information on how the reviewed interventions/paper have been retrieved, included and reviewed.*<br>*A systematic review includes a methodology section that explicitly defines the sources of information chosen, the search strategy, the inclusion/exclusion criteria, etc.*	"The purpose of this literature review was to describe and evaluate the applications of ICT in dietary intake assessment. The aim was to explore a broad range of studies that assessed the use of new as well as renewed technologies to obtain dietary data."
**3.3 Review about accuracy/validity AND feasibility**<br><br>**3.3.1 Narrative**<br>**3.3.2 Systematic**<br>**3.3.3 Meta-analysis**	The paper presents **a systematic review** focusing on both and **accuracy/validity** and on the **feasibility** of using mobile-based interventions.	"This study systematically reviewed evidence on smartphones and their **viability** for **measuring and influencing physical activity**."

4) Measures of behaviour (Primary research articles)

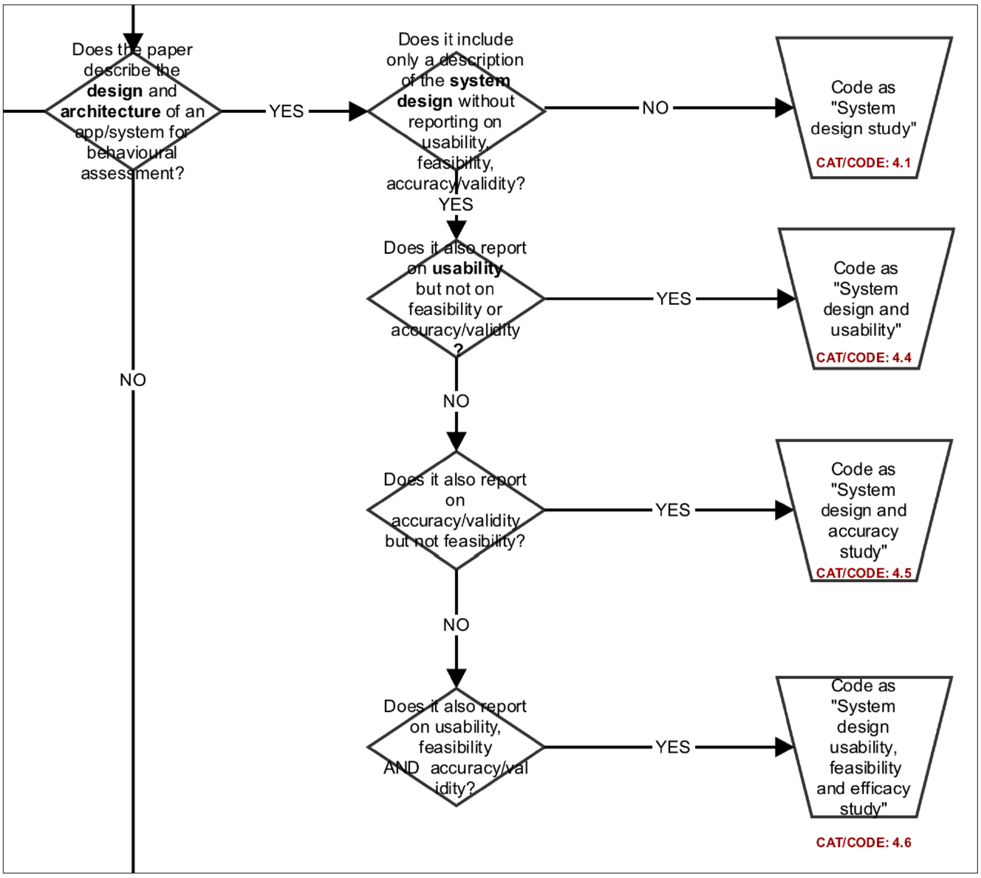

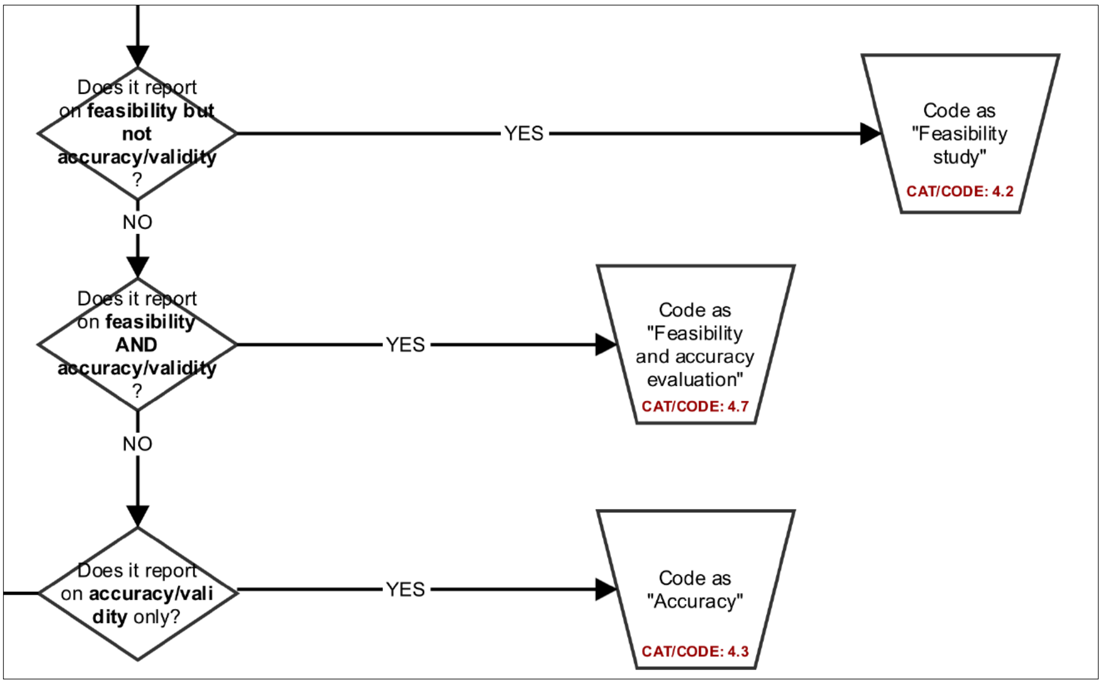

Label	Definition	Examples of study purpose
**4.1 System design**	The paper **describes the design** and architecture of a system/app for measuring behaviour, ***without* reporting any kind of evaluation** or results.<br><br>*Note: System design is the activity of proceeding from an identified set of requirements for a system to a design that meets those requirements. A distinction is sometimes drawn between high-level or architectural design, which is concerned with the main components of the system and their roles and interrelationships, and detailed design, which is concerned with the internal structure and operation of individual components.*<br><br>If the paper also reports on usability measures it needs to be categorised as: **4.4.**<br><br>If the paper also reports on accuracy/validity it needs to be categorised as: **4.5.**<br><br>If the paper also reports on usability AND accuracy/validity, code as **4.6**.	"This paper describes the motivation for, and overarching design of, an open-source hardware and software system to enable population-scale, longitudinal measurement of physical activity and sedentary behavior using common mobile phones."
**4.2 Feasibility**	The article reports on **feasibility of using technologies for assessing behaviour.** It might include formative research and development of an existing app/intervention. It focuses on outcomes such as: acceptability, utilisation, retention and recruitment rates, adherence or compliance to processes and procedures. It **does not report an evaluation of accuracy/validity** of the instrument.<br><br>If it also reports on accuracy/validity, code as: **4.7**.<br><br>If it also reports on usability AND accuracy/validity, code as **4.6**.<br><br>If the paper reports on system design AND usability only, categorise as: **4.4**.	"The aim of the present study was to evaluate the usability and feasibility of a mobile phone program in recording of symptoms during a physical exercise trial."
**4.3 Accuracy/Validity**	The article reports the results of a study focusing on testing the **accuracy, validity and/or reliability** of an app/system for measuring behaviour. The results of the study must contain measures of accuracy. It may encompass methods and comparisons of tools used for estimating energy expenditure, dietary assessment or physical activity.<br>*Note: Accuracy, precision and validity/reliability: In the fields of science, engineering, industry, and statistics, the accuracy of a measurement system is the degree of closeness of measurements of a quantity to that quantity's actual (true) value*[5]. *The precision of a measurement system, related to reproducibility and repeatability, is the degree to which repeated measurements under unchanged conditions show the same results.* [5,6]<br><br>*In psychometrics the term accuracy is interchangeably used with validity and constant error. Precision is a synonym for reliability and variable error. The validity of a measurement instrument or psychological test is established through experiment or correlation with behavior. Reliability is established with a variety of statistical techniques, classically through an internal consistency test like Cronbach's alpha to ensure sets of related questions have related responses, and then comparison of those related question between reference and target population [6]*<br><br>If the paper also presents a system design, it needs to be categorised as: **4.5**.<br><br>If it also reports on feasibility, code as: **4.7**.<br><br>If it also reports on usability AND feasibility, code as **4.6** [B1234]	"In this paper, we present a practical solution for estimating an individual's energy expenditure using mobile phones in real-life ambulatory settings."<br><br>"The objective of this study is to investigate two novel approaches to provide the missing information, enabling food volume estimation from a single image."<br><br>"The purpose of this study was to compare energy expenditure measured using DLW with self-reported EI using a PDA or written food record (written record) in weight-stable individuals over 7 days. The hypothesis was that the PDA would be as accurate as the written record in assessing EI in weight-stable volunteers when compared to TEE."

**4.4 System design AND usability**	The paper **<u>describes the design</u>** and architecture of a system/app for measuring behaviour, and reports results about formal **<u>usability testing</u>**.<br>*Note: Usability is the extent to which a product can be used to achieve specified goals with effectiveness, efficiency, and satisfaction in a specified context of use. The word "usability" also refers to methods for improving ease-of-use, learnability, task-efficiency, memorability, ease-of-use, satisfaction, and usefulness.*[3]<br><br>If the paper also reports on accuracy/validity, it needs to be categorised as: **4.5**.<br><br>If it also reports on feasibility, code as: **4.7**.<br><br>If it also reports on feasibility AND accuracy/validity, code as **4.6**.	"Mobile telephones with an integrated camera can provide a unique mechanism for collecting dietary information that reduces burden on record-keepers. Objectives for this study were to test whether participant's proficiency with the mobile telephone food record improved after training and repeated use and to measure changes in perceptions regarding use of the mobile telephone food record after training and repeated use."
**4.5 System design AND accuracy/validity study**	The paper **<u>describes the design</u>** and architecture of a system/app focusing on behavioural assessment, and reporting results about its **<u>accuracy/validity</u>**.<br><br>If the paper also reports on feasibility, code as: **4.7**.<br><br>If it also reports on feasibility AND accuracy/validity, code as **4.6**.<br><br>If the paper reports on system design AND usability only, categorise as: **4.4**.	"In this paper, we design a system to measure the patient's activity by estimating his walking habits. The system, ready to be integrated in a mobile health application, consists of an inertial sensor with a tri-axial orthogonal accelerometer attached to patient's foot, while the sensor is connected to a smart phone for data processing."
**4.6 System design AND usability, AND feasibility AND accuracy/validity study**	The paper **<u>describes the design</u>** and architecture of a system/app focusing on behavioural assessment, and reports results about its **<u>usability/feasibility</u>** and **<u>accuracy/validity</u>**.<br><br>*Note: The study should encompass all three elements to be included in this category; the methodology and results sections should include the elements to determine whether the study analysed and reported measures of usability/feasibility and accuracy/validity.*	"To present the design and pilot test results of a continuous multi-sensor monitoring system of real-world physiological conditions and daily life (activities, travel, exercise, and food consumption), culminating in a Web-based graphical decision-support interface."
**4.7 Feasibility AND accuracy study**	The paper **<u>describes a pilot/feasibility study</u>** testing the feasibility of an app/system for measuring behaviour, and reports results about its **<u>accuracy/validity</u>**.<br><br>If it also reports on usability (including feasibility and accuracy/validity), code as **4.6**.<br><br>If the paper reports on system design AND usability, categorise as: **4.4**.<br><br>If the paper also presents a system design, categorise as: **4.5**.	"In this paper, the authors describe a method of accurately detecting human activity using a smartphone accelerometer paired with a dedicated chest sensor. The design, implementation, testing and validation of a custom mobility classifier are also presented. An initial trial of N=6 healthy individuals (5M, 1F) was conducted to validate our algorithms, and test the feasibility of using smartphones to aid activity recognition. The chest sensor was not available for this pre-trial."

5) Overviews of apps and their content

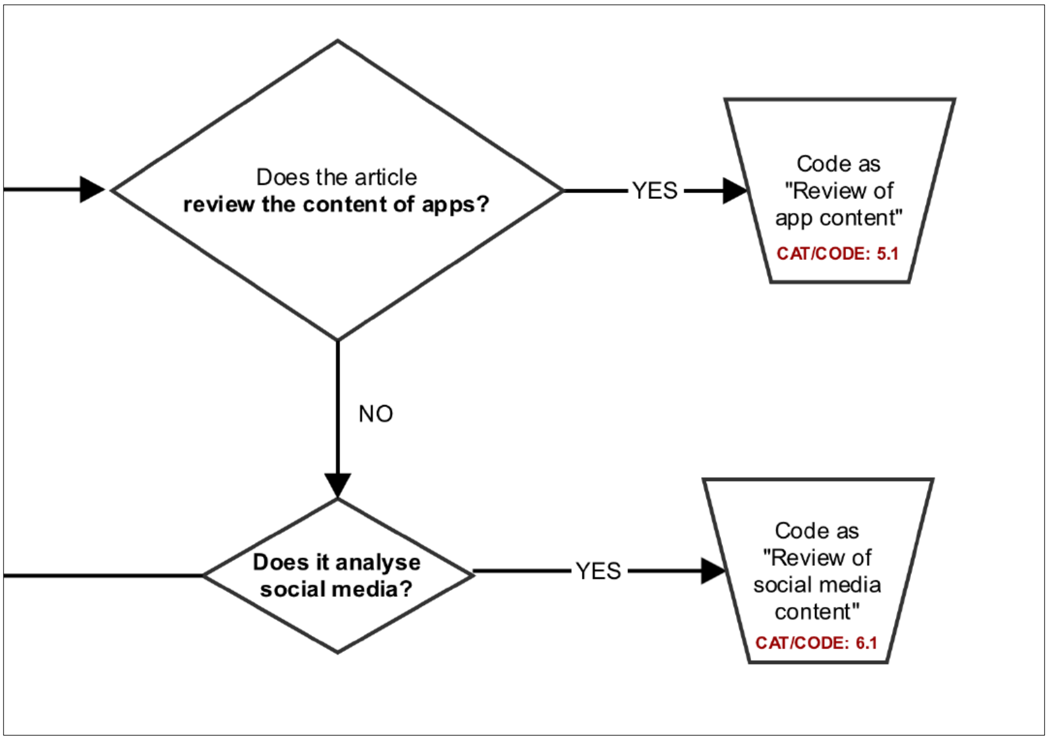

Label	Definition	Examples of study purpose
**5.1 Reviews of app content**	The paper reports a **review of the content of smartphone apps**. The unit of analysis of the review is the smartphone app and its content.<br><br>*Note: The study can be based on a systematic selection of the unit of analysis.*	"The goal of the study was to evaluate diet/nutrition and anthropometric tracking apps based on incorporation of features consistent with theories of behavior change."

6) Descriptions and analyses of social media

Label	Definition	Examples of study purpose
**6.1 Reviews of social media content**	Descriptive analyses of social media, including content analyses of user-generated content (e.g., blogs, forum posts, tweets, comments) about PA, diet or obesity-related social media communities. The unit of analysis is the blog entry, the content posted.	"We describe a topic modelling framework for discovering health topics in Twitter, a social media website. This is an exploratory approach with the goal of understanding what health topics are commonly discussed in social media. This paper describes in detail a statistical topic model created for this purpose, the Ailment Topic Aspect Model (ATAM), as well as our system for filtering general Twitter data based on health keywords and supervised classification. We show how ATAM and other topic models can automatically infer health topics in 144 million Twitter messages from 2011 to 2013."

References

1. Arain M, Campbell MJ, Cooper CL, Lancaster GA. What is a pilot or feasibility study? A review of current practice and editorial policy. BMC Med Res Methodol 2010 Jul 16;10(1):67. PMID: 20637084
2. Daintith J, Wright E. A dictionary of computing. Oxford University Press, Inc.; 2008.
3. Gould JD, Lewis C. Designing for usability: key principles and what designers think. Commun ACM 1985;28(3):300–311.
4. Singal AG, Higgins PDR, Waljee AK. A Primer on Effectiveness and Efficacy Trials. Clin Transl Gastroenterol 2014 Jan 2;5(1):e45.
5. BIPM I, IFCC I, IUPAC I, ISO O. The international vocabulary of metrology—basic and general concepts and associated terms (VIM), 3rd edn. JCGM 200: 2012. JCGM Jt Comm Guid Metrol 2008;
6. Accuracy and precision [Internet]. Wikipedia Free Encycl. 2015 [cited 2015 Mar 26]. Available from: http://en.wikipedia.org/w/index.php?title=Accuracy_and_precision&oldid=652988666. Accessed: 2015-06-21. (Archived by WebCite® at http://www.webcitation.org/6ZSMlHh7s)
