# Supplementary material for: Mobile Phone and Web 2.0 Technologies for Weight Management: A Systematic Scoping Review
Source: J Med Internet Res. 2015 Nov 16;17(11):e259. doi: 10.2196/jmir.5129 (PMC4704945; doi:10.2196/jmir.5129)
Supplement: Multimedia Appendix 3 [file jmir_v17i11e259_app3.pdf]

Mobile Phone and Web 2.0 Technologies for Weight Management: A Systematic Scoping Review

Multimedia Appendix 3: Characteristics of Selected Studies

Mobile Phone and Web 2.0 Technologies for Weight Management: A Systematic Scoping Review........................1
1a) Technologies for behavior change (primary research articles)........................2
1.1) Design and development (21 studies)........................2
1.2) Feasibility (33 studies)........................5
1.3) Evaluations (71 studies)........................10
1.4) Process evaluation/Causal-comparative (20 studies)........................22
1.5) Design and Feasibility (46 studies)........................25
1.6) Design and Evaluations (2 studies)........................34
1.7) Feasibility and Evaluations (55 studies)........................35
1.8) Design, Feasibility and Evaluations (22 studies)........................44
1b) Technologies for behavior change (reviews)........................49
1.1) Design and development (10 studies)........................49
1.2) Evaluations (39 studies)........................52
2a) Technologies for measuring behavior (primary research articles)........................60
2.1) Design and development (11 studies)........................60
2.2) Feasibility (3 studies)........................62
2.3) Evaluations (30 studies)........................63
2.4) Design and Feasibility (10 studies)........................68
2.5) Design and Evaluations (24 studies)........................70
2.6) Feasibility and Evaluations (17 studies)........................75
2.7) Design, Feasibility, and Evaluations (7 studies)........................78
2b) Technologies for measuring behavior (reviews)........................80
2.1) Design and development (3 studies)........................80
2.2) Feasibility (1 study)........................80
2.3) Evaluations (8 studies)........................80
3) Overviews of apps and social media content........................82
Reference list of included studies (in alphabetical order)........................89

Notes for all tables:

- **Objectives of the paper:** These are verbatim transcriptions of the content provided by the authors in the paper.
- **Target population:** Condition (obese, overweight, healthy, etc.), gender (female/male only, mostly female/male) and age group (children, adolescents, adults, etc.) are deducted from the title, abstract and full-text of the paper. If the authors included more detailed information regarding the target group, this was included (e.g., patients, employees, cancer survivors, etc.). If not explicitly mentioned, the characteristics of the recruited population were used to define the target group. "General pop." (population) means that condition, age and gender were not specified.
- **Methodology/Study design:** "Descriptive" papers included descriptions and proof of concepts, without conducting any qualitative or quantitative study. "Qualitative" papers included results of interviews, focus groups, with qualitative syntheses of the data. "Quantitative" papers reported quantitative syntheses and evaluations of studies, including non-experimental, quasi-experimental and experimental studies. "Mixed" include studies that included both qualitative and quantitative syntheses of the data. In brackets we highlighted links to RCTs, and we included, if available, the name of the trial and its associated registry number.
- **Number of studies and %** are relative to each research theme category and type of technology used.

1a) Technologies for behavior change (primary research articles)

Study ID	Author (Year), Country	Objectives of the paper	Technologies used	Relevant Outcomes	Target population	Methodology/Study design
1.1) Design and development (21 studies)						
Mobile technologies (12 studies, 57%)<br>***Study design: Descriptive (12/12, 100%)***						
99	Damkliang (2014), Thailand	This paper proposes the design and a prototype for nutrition recommendation and energy expenditure application.	Mobile features: App<br>App name: not provided<br>Operative system: iOS	PA and diet	General pop.	Descriptive
139	Freyne (2010), Australia	Here we present a tool designed to maximise the support offered to users through mobile technology.	Mobile features: App<br>App name: *Weight Management Mentor*<br>Operative system: iOS	Weight-related and behavioural (PA, diet)	General pop.	Descriptive
150	Gay (2012), Australia	Our team has developed a mobile health and fitness app that has been available via Android market since February 2011. The objective of this paper is to share our experience with rolling out a mobile health and fitness app.	Mobile features: App<br>App name: *myFitnessCompanion*<br>Operative system: Android	PA	General pop.	Descriptive
188	Hui-Huang (2012), Taiwan	We have developed a health management application (app) run on iPhone. It uses the QR-Code to input calorie and nutrition information of food, snacks and drinks.	Mobile features: App (using camera)<br>App name: not provided<br>Operative system: iOS	Diet	General pop.	Descriptive
208	Keung (2013), USA	In this paper, we will discuss the integration of location technologies like GPS with fitness related technologies, when considering the parameters of youth-targeted mobile app development. Our paper will also describe development goals, the structure of the game, and initial testing.	Mobile features: App (using accelerometer and GPS)<br>App name: *BunnyBolt*<br>Operative system: Android	PA	Children and adolescents	Descriptive
244	Lim (2012), USA	We present a motivational exercise gaming system whose goal is to increase the activity of elders with complex chronic conditions.	Mobile features: App (using accelerometer)<br>App name: *HealthOS*, *DailyAlert*<br>Operative system: Python	PA	Patients and caregivers	Descriptive
264	Marcus (2011), USA	This article explains the conceptual design process for a mobile application that seeks to persuade people to make health behaviour changes. The article introduces the philosophy and principles behind the application and describes an early conceptual prototype design.	Mobile features: App<br>App name: *Health Machine*<br>Operative system: iOS	PA and diet	General pop.	Descriptive

Study ID	Author (Year), Country	Objectives of the paper	Technologies used	Relevant Outcomes	Target population	Methodology/Study design
265	Marins (2011), Brazil	This paper presents SmartRabbit and how the location-based games concepts were used in the development of this game. SmartRabbit is a mobile running game where players compete using a smartphone with GPS.	Mobile features: App (using GPS)<br>App name: *SmartRabbit*<br>Operative system: Android	PA	General pop.	Descriptive
275	McMahon (2013), USA	This article describes features of a new application, Ready-Steady, highlighting approaches used in its design and development, and implications for clinical practice.	Mobile features: App (using accelerometer)<br>App name: *ReadySteady*<br>Operative system: iOS	PA	Patients and caregivers	Descriptive
** 277<br><br>[Also cited in Section 2a]	Mendi (2013), Turkey	In this paper, we introduce a real-time food intake monitoring system for mobile devices.	Mobile features: App (using accelerometer and Bluetooth)<br>App name: not provided<br>Operative system: Android	Diet	General pop.	Descriptive
440	Wylie (2009), UK	In this paper we present a novel general health monitoring software for mobile phones. This software is aimed at helping users monitor, record, as well as improve their fitness levels.	Mobile features: App (using GPS and Bluetooth)<br>App name: *Heart Angel*<br>Operative system: Symbian	PA	Patients and caregivers	Descriptive
448	Young-Seob (2014), South Korea	This paper presents a semi-automated obesity-care application for helping people manage their own lifestyles.	Mobile features: App<br>App name: *Lifestyle Manager*<br>Operative system: Android	Weight-related and behavioural (PA, diet)	General pop.	Descriptive
Web 2.0 technologies (1 study, 5%)<br>***Study design: Descriptive (1/1, 100%)***						
49	Bonacina (2009), Italy	We designed and implemented a dynamic web application for controlling eating and physical activity behaviours based on the cooperation between two communities: the Patient community, i.e. people who decided to follow a healthy lifestyle to lose weight and the Nutritionist community, i.e. professionals who guide patients to reach a healthy lifestyle.	Web 2.0 features: Custom social networking site	PA and diet	Patients and caregivers	Descriptive
Mobile and Web 2.0 technologies (8 studies, 38%)<br>***Study design: Descriptive (8/8, 100%, of which 1 linked to subsequent RCT)***						
28	Ayubi (2012), USA	This paper describes the motivation for and overarching design of the app and its functional and non-functional features.	Mobile features: App (using accelerometer)<br>App name: *PersonA, SocioPedometer*<br>Operative system: Android<br><br>Web 2.0 features: Facebook app	PA	General pop.	Descriptive

Study ID	Author (Year), Country	Objectives of the paper	Technologies used	Relevant Outcomes	Target population	Methodology/Study design
84	Chuah (2011), USA	In this paper, we design a location-based mobile application for encouraging school children or college students to participate in outdoor physical activities in a social friendly manner. Our main goal in developing this application is to combat the problem of obesity among children, adolescent, and college students.	Mobile features: App<br>App name: *FitnessTour*<br>Operative system: Android<br><br>Web 2.0 features: Facebook app	PA	Children and college students	Descriptive
109	Ding (2012), USA	This paper describes our preliminary work on developing a physical activity monitoring and sharing platform (PAMS) especially suited for capturing PA that are part of the lifestyle in wheelchair users and motivating them to be physically active via web-based or mobile social networking applications.	Mobile features: App (using accelerometer)<br>App name: *Physical Activity Monitoring and Sharing (PAMS)*<br>Operative system: Android<br><br>Web 2.0 features: Facebook app	PA	General pop.	Descriptive
110	Ding (2013), Australia	This paper is focused on describing the mobile applications in the delivery of intervention components, and user interface developed to trial among middle-aged males in regional Queensland town of Gladstone, Australia.	Mobile features: App and SMS<br>App name: *ManUp*<br>Operative system: iOS<br><br>Web 2.0 features: "Social media group challenges"	Weight-related and behavioural (PA, diet)	Middle-aged adults (males only)	Descriptive (related to RCT)<br><br>Trial name: *ManUp* (ACTRN12611000081910)
243	Liew (2012), Malaysia	This study will propose a framework that is suitable for children. The objectives of this study are: 1) To explore various persuasive technology applications; 2) To study the strengths and weaknesses of various approaches used in persuasive technology; 3) To propose a framework for childhood obesity intervention using persuasive web-mobile technology.	Mobile features: App<br>App name: not provided<br>Operative system: Not specified<br><br>Web 2.0 features: Custom social networking site	PA and diet	Children	Descriptive
290	Mulas (2013), Italy	This paper presents a persuasive web application for sport and health, designed to motivate people in their exercising activity. For the first time ever, our application makes available on a web browser some features previously available only through a mobile application.	Mobile features: App<br>App name: *Everywhere Run!*, *Everywhere Race!*<br>Operative system: Android<br><br>Web 2.0 features: Facebook app	PA	General pop.	Descriptive

Study ID	Author (Year), Country	Objectives of the paper	Technologies used	Relevant Outcomes	Target population	Methodology/Study design
363	Silva (2011), Portugal	This paper presents a mobile health application for a dietary evaluation and the implementation of challenges, alerts, and constantly motivates the user to use the system and keep the diet plan. The main goal of this application is to offer a motivation tool for weight reduction and increase physical activity.	Mobile features: App (using accelerometer and camera) and SMS<br>App name: *SapoFitness*<br>Operative system: Android, iOS<br><br>Web 2.0 features: Facebook group	PA and diet	General pop.	Descriptive
385	Stroulia (2013), Canada	Grounding our framework the Theory of Planned Behavior and the Intention-Behavior Gap Theory, we explicitly designed all the functions in our applications to influence behavior. In this paper we discuss the framework and the first two applications we have developed with it.	Mobile features: App<br>App name: *EASI, Physitivity*<br>Operative system: Java<br><br>Web 2.0 features: Sharing on Facebook	PA and diet	Overweight or obese adults, with Type 2 Diabetes	Descriptive
1.2) Feasibility (33 studies)						
Mobile technologies (26 studies, 79%)<br>***Study design: Qualitative (8/26, 31%, of which 1 linked to subsequent RCT); Mixed (10/26, 38%); Quantitative (8/26, 31%, of which 3 linked to subsequent RCT)***						
59	Buis (2013), USA	The purpose of this investigation was to use the RE-AIM framework to document the efficacy of the programme by focusing on perceptions of satisfaction, usage, and behavior change among individuals who used txt4health in pilot studies in Southeast Michigan and Greater Cincinnati.	Mobile features: SMS	Diet	Adults, at risk of Type 2 Diabetes (mostly females)	Quantitative<br><br>Trial name: *txt4health*
61	Burke (2009), USA	The SMART weight loss trial examined the impact of replacing the standard paper record used for self-monitoring with a personal digital assistant (PDA). This paper describes the design, methods, intervention, and baseline sample characteristics of the trial.	Mobile features: PDA App<br>App name: *DietMatePro, CalculFit*<br>Operative system: Palm<br>Tested on: PalmOne Tungsten E2	Weight-related and behavioural (diet)	Obese adults (mostly females)	Quantitative (related to RCT)<br><br>Trial name: *SMART* (NCT00277771)
97	Cushing (2011), USA	To examine the effectiveness of a personal electronic device (PED) in order to improve adherence to self-monitoring of dietary intake and physical activity.	Mobile features: App<br>App name: *Livestrong*<br>Operative system: iOS<br>Tested on: iPod	Weight-related and behavioural (PA, diet)	Overweight or obese adolescents (females only)	Mixed
106	Dennison (2013), UK	Explore young adults' perspectives on apps related to health behavior change. It sought their experiences and views of features that might support health behavior change and issues that contribute to interest in and willingness to use such apps.	Mobile features: App<br>App name: not provided<br>Operative system: Android, Blackberry, iOS	PA and diet	University students (mostly females)	Qualitative

Study ID	Author (Year), Country	Objectives of the paper	Technologies used	Relevant Outcomes	Target population	Methodology/Study design
123	Ehlers (2014), USA	The purpose of this study was to describe which theory-based behavioural and technological features middle-aged women prefer to be included in a mobile application designed to help them adopt and maintain regular physical activity (PA).	Mobile features: App<br>App name: not provided<br>Operative system: Not specified	PA	Middle-aged adult women	Quantitative
142	Fukuoka (2011), USA	The purposes of this analysis were to explore applicability of potential components of a mobile phone-based healthy lifestyle program and to understand motivators and barriers to continued engagement in a mobile phone healthy lifestyle program.	Mobile features: App and SMS<br>App name: not provided<br>Operative system: Not specified	PA and diet	Overweight, sedentary adults (mostly females)	Qualitative
145	Fukuoka (2012), USA	Explore the acceptability of components of a mobile phone/pedometer-based physical activity program and to understand motivators and barriers to increase physical activity in a diverse sample of sedentary women	Mobile features: App and SMS<br>App name: not provided<br>Operative system: Java<br>Tested on: Motorola RAZR V3xx	PA	Sedentary adults (females only)	Qualitative
151	Gerber (2009), USA	We investigated the feasibility of mobile phone text messaging to enable ongoing communication with African-American women participating in a weight management program.	Mobile features: SMS	Weight-related and behavioural (PA, diet)	Obese adults (females only)	Quantitative
156	Gorton (2011), New Zealand	The present study aimed to explore the potential for weight-loss interventions to be delivered via mobile phone. The present study aimed to understand the acceptability and desired features of a weight-loss intervention delivered via mobile phone.	Mobile features: SMS	PA and diet	Adolescents and adults, (mostly females)	Mixed
174	Hearn (2013), Australia	Study aimed at determining the opinions of women and perinatal health care providers about what online information is needed, in what form and how best this could be presented to promote healthy lifestyles during the perinatal period.	Mobile features: App<br>App name: *Ngala HYHB*<br>Operative system: Not specified	PA and diet	Perinatal women	Mixed<br><br>Trial name: *Healthy You, Healthy Baby*
173	Hearn (2014), Australia	This paper presents the findings of a project designed to develop an online resource to promote healthy lifestyles during the perinatal period. The outcome was the development of the Healthy You, Healthy Baby website and smartphone app.	Mobile features: App<br>App name: *Ngala HYHB*<br>Operative system: Not specified	PA and diet	Perinatal women and healthcare providers	Qualitative<br><br>Trial name: *Healthy You, Healthy Baby*

Study ID	Author (Year), Country	Objectives of the paper	Technologies used	Relevant Outcomes	Target population	Methodology/Study design
179	Helander (2014), Finland	The aim of the study was to assess the factors related to sustained use of a free mobile app that promotes healthy eating through photographic dietary self-monitoring and peer feedback.	Mobile features: App (using camera)<br>App name: *The Eatery*<br>Operative system: iOS<br>Tested on: iPhone	Diet	Healthy adults	Mixed
183	Hingle (2013), USA	To develop and test messages and a mobile phone delivery protocol designed to influence the nutrition and physical activity knowledge, attitudes, and behavior of adolescents.	Mobile features: SMS	PA and diet	Children and adolescents (mostly females)	Mixed
218	Kim (2013), South Korea	We developed and pilot-tested a personalized text messaging service program based on data from electronic health records (EHRs) and lifestyle questionnaires for weight control.	Mobile features: SMS	Weight-related and behavioural (PA, diet)	Overweight or obese adults (mostly males)	Quantitative
229	Kornman (2010), Australia	To examine adolescent and facilitator participation in the first 10 months of an obesity management intervention including electronic contact (e-contact) via e-mail and short message service (SMS) communication.	Mobile features: SMS	Weight-related and behavioural (PA, diet)	Overweight or obese adolescents (gender not specified)	Quantitative (related to RCT)<br><br>Trial name: *Loozit*®<br>(ACTRN126060001755 72)
241	Leijdekkers (2013), Australia	This paper reflects on 7 years of experience in mobile health and fitness app development. It analyses the uptake of a health and fitness app by the healthcare industry and end-users dealing with chronic disease management. The use of the app is analysed from an end-user perspective.	Mobile features: App<br>App name: *myFitnessCompanion*<br>Operative system: Android	Weight-related and behavioural (PA, diet)	Adults and healthcare providers	Mixed
250	Lister (2013), USA	Explore the utility of iPads in facilitating diet-related behavior change.	Mobile features: App<br>App name: not provided<br>Operative system: iOS<br>Tested on: iPad	Diet	Adults (mostly females)	Qualitative
278	Mendis (2014), Australia	Explore: 1) Feasibility of recruiting 17–25 year old aboriginals in Dubbo, NSW to obtain baseline data on the rate of overweight and obesity, and collect socio- demographic, nutrition, exercise and mobile phone usage data; 2) Views of participants, with a waist measurement of 94 cm or more, about being overweight or obese, and using text messages and incentives to promote healthy lifestyles.	Mobile features: SMS	Weight-related and behavioural (PA, diet)	Ethnic minority adolescent and obese adult patients, at risk of CVD and T2D, (males only)	Mixed

Study ID	Author (Year), Country	Objectives of the paper	Technologies used	Relevant Outcomes	Target population	Methodology/Study design
284	Mojica (2014), USA	This study assessed access/use of various media (cell phones, computers, gaming systems, Internet) among adolescent Latino girls and examined the relationship between PA and media access/use.	Mobile features: SMS	PA	Latino minority children and adolescents (females only)	Quantitative
329	Rabin (2011), USA	We conducted formative research aimed at developing theoretically and empirically based smartphone apps that incorporate user preferences.	Mobile features: App and SMS<br>App name: *iTreadmill*, *iFitness Hero*, *Exercise Tracker*<br>Operative system: iOS<br>Tested on: iPhone	Diet	Adults	Mixed
355	Sharifi (2013), USA	Examine parental acceptability and preferences for text messaging to support paediatric obesity-related behavior change.	Mobile features: SMS	Weight-related and behavioural (PA, diet)	Parents of overweight and obese children (mostly females)	Qualitative (related to RCT)<br><br>Trial name: *Healthy Habits*, *Happy Homes* (NCT01565161)
365	Silveira (2013), Italy	We developed an IT-based system for active and healthy aging aiming at improving elderly's balance and strength. The objective is to run a pilot study to investigate: (i) the feasibility of assisting the autonomous, physical training of independently living elderly with the system, (ii) the adherence of the participants to the training plans, and (iii) the effectiveness of the motivation instruments built into the system.	Mobile features: App<br>App name: *Active Lifestyle*<br>Operative system: iOS<br>Tested on: iPad	PA	Older adults (mostly females)	Mixed
393	Taveras (2012), USA	The goal of the proposed study was to develop and test an intervention delivered to racial/ethnic minority and low socioeconomic status parents of 2–5 year old children in their homes, designed to promote adoption of household routines and parenting strategies that are preventive of overweight and obesity. In this paper we describe the development and baseline assessments of the intervention.	Mobile features: SMS	Weight-related and behavioural (PA, diet)	Racial/ethnic minority and low socioeconomic status children and adults	Quantitative (related to RCT)<br><br>Trial name: *Healthy Habits*, *Happy Homes* (NCT01565161)
436	Woolford (2010), USA	The objective of the present study was to test the feasibility and acceptability of a computerized system to send tailored messages to the mobile phones of obese adolescents enrolled in the Michigan Pediatric Outpatient Weight Evaluation and Reduction (MPOWER) programme.	Mobile features: SMS	Weight-related and behavioural (PA, diet)	Overweight or obese children and adolescents (mostly females)	Mixed<br><br>Trial name: *MPOWER*

Study ID	Author (Year), Country	Objectives of the paper	Technologies used	Relevant Outcomes	Target population	Methodology/Study design
435	Woolford (2011), USA	This study explored participants' perspectives about message content. A library of messages was developed focused on topics central to weight management	Mobile features: SMS	Weight-related and behavioural (PA, diet)	Obese children and adolescents (mostly females)	Qualitative
438	Woolford (2012), USA	This study uses a variation of the Photo-voice method to explore the types of images that obese adolescents believe would be helpful in supporting their weight loss efforts.	Mobile features: SMS	Weight-related and behavioural (PA, diet)	Overweight or obese adolescents (mostly females)	Qualitative
Web 2.0 technologies (4 studies, 12%) ***Study design: Qualitative (1/4, 25%); Mixed (1/4, 25% of which 1 linked to subsequent RCT); Quantitative (2/4, 50%, of which 1 linked to subsequent RCT)***						
157	Graham (2014), USA	This article describes the development and use of e-Moms of Rochester (e-Moms Roc), an electronic intervention (e-intervention), to address this health issue in a socioeconomically diverse sample of pregnant women.	Web 2.0 features: Blog	Weight-related and behavioural (PA, diet)	Pregnant women	Mixed (related to RCT)<br><br>Trial name: *e-Moms Roc* (NCT01331564)
310	Pagoto (2014), USA	The purpose of the current study is to report the feasibility of a Twitter-based core-strengthening exercise challenge by reporting the number and characteristics of participants, the frequency of participation, the extent to which participants spread the challenge to others, and the extent to which participants experience social support from online friends relative to in-person friends or family.	Web 2.0 features: Twitter #PlankADay challenge	Weight-related and behavioural (PA)	Adults (mostly females)	Quantitative
437	Woolford (2013), USA	To explore patients' and parents' perspectives about developing a Facebook group as a component of a paediatric weight management program.	Web 2.0 features: Facebook group	Weight-related and behavioural (PA, diet)	Overweight or obese patients, adolescents and adults (mostly females)	Qualitative
409	Turner-McGrievy (2013), USA	This study explored differences in psychosocial and physiological variables in response to being presented with information on weight loss through either reading text on a website or listening to the same information via podcast.	Web 2.0 features: Podcast	Weight-related and behavioural (PA, diet)	Adolescents and adults (mostly females)	Quantitative (related to RCT)
Mobile and Web 2.0 technologies (3 studies, 9%) ***Study design: Qualitative (1/3, 33%, of which 1 linked to subsequent RCT); Mixed (2/3, 67%, of which 1 linked to subsequent RCT)***						

Study ID	Author (Year), Country	Objectives of the paper	Technologies used	Relevant Outcomes	Target population	Methodology/Study design
122	Edwards (2013), UK	This paper presents a field study, eHealth, which used technology probes to investigate the reactions of adolescents to different sets of exercise-related technologies. The aim was to prompt them to generate ideas about the types of artefacts (both hardware and software) that they and their peer group would be motivated to use.	Mobile features: PDA App<br>App name: not provided<br>Operative system: Not specified<br><br>Web 2.0 features: Potential use of social networking components, blogs	PA	Children and adolescents	Mixed
279	Merchant (2014), USA	The objective of this study was to describe participant exposure to a Facebook page designed to deliver content to overweight/obese college students in a weight loss randomized controlled trial and examine participant engagement with behavior change campaigns for weight loss delivered via Facebook.	Mobile features: App and SMS<br>App name: *Goal Getter*<br>Operative system: iOS<br><br>Web 2.0 features: Facebook page, blogs	Weight-related and behavioural (PA, diet)	Overweight or obese college students (mostly females)	Mixed (related to RCT)<br><br>Trial name: *SMART* (NCT01200459)
414	Vandelanotte (2013), Australia	Examine opinions and perceptions regarding the use of Internet and mobile phones to improve physical activity and nutrition behaviours for middle-aged men.	Mobile features: App and SMS<br>App name: *ManUp*<br>Operative system: Not specified<br><br>Web 2.0 features: Potential use of social networking components	Weight-related and behavioural (PA, diet)	Adults (males only)	Qualitative (related to RCT)<br><br>Trial name: *ManUp* (ACTRN12611000081910)
1.3) Evaluations (71 studies)						
Mobile technologies (56 studies, 79%)<br>***Study design: Quantitative (56/56, 100%, of which 46 linked to subsequent RCT and 18 RCT study protocols)***						
1	Acharya (2011), USA	The study aimed to describe the differences in dietary changes at 6 months between participants randomly assigned to use a paper record or PDA for self-monitoring in a clinical trial of weight-loss treatment.	Mobile features: PDA App<br>App name: *DietMatePro*, *CalculFit*<br>Operative system: Palm<br>Tested on: PalmOne Tungsten E2	Weight-related and behavioural (diet)	Overweight or obese adults (mostly females)	Quantitative (related to RCT)<br><br>Trial name: *SMART* (NCT00277771)
35	Batch (2014), USA	To describe the design and rationale of the NHLBI-sponsored Cell Phone Intervention for You (CITY) study, which is a single centre, randomised three-arm trial	Mobile features: App<br>App name: not provided<br>Operative system: Android	Weight-related and behavioural (PA, diet)	Overweight or obese adults	Study protocol for RCT<br><br>Trial name: *Cell Phone Intervention for You (CITY)* (NCT01092364)

Study ID	Author (Year), Country	Objectives of the paper	Technologies used	Relevant Outcomes	Target population	Methodology/Study design
** 37<br><br>[Also cited in Section 2a]	Beasley (2008), USA	The purpose of this 4-week randomized, controlled trial was to evaluate the effectiveness of DietMatePro. Two primary aims were to 1) compare concordance of reported energy and nutrient intakes obtained from DietMatePro and paper food diaries with 24-hr recall; and 2) compare adherence to a strict dietary regimen among users randomized to use DietMatePro versus a paper-based food record.	Mobile features: PDA App<br>App name: *DietMatePro*<br>Operative system: Palm<br>Tested on: PalmZire 21	Diet	Overweight or obese adults (mostly females)	Quantitative (related to RCT)
71	Castelnuovo (2010), Italy	This paper describes the design of the TECNOB study, a two-arm randomized controlled trial (related to RCT). The aims of this study are to evaluate the effectiveness of the TEC- NOB program in a sample of obese people with type 2 diabetes seeking treatment for weight reduction and to find out what behavioural and psychological variables are predictive of treatment success.	Mobile features: App and SMS<br>App name: *METADIETA*<br>Operative system: Not specified	Weight-related	Obese adult patients with T2D	Study protocol for RCT<br><br>Trial name: *TECNOB* (ISRCTN07265661)
70	Castelnuovo (2011), Italy	To examine ad interim effectiveness of a 12-month multidisciplinary telecare intervention for weight loss provided to obese patients with Type 2 Diabetes.	Mobile features: App and SMS<br>App name: *METADIETA*<br>Operative system: Not specified	Weight-related and behavioural (diet)	Obese adult patients with T2D	Quantitative (related to RCT)<br><br>Trial name: *TECNOB* (ISRCTN07265661)
81	Cheung (2012), China	The purpose of the study was to compare the effectiveness of different strategies applied to a pedometer-based intervention designed to promote the physical activity level of working adults.	Mobile features: SMS	Weight-related and behavioural (PA)	Employees (mostly males)	Quantitative (related to RCT)
88	Collins (2014), Australia	The aim was to evaluate the 12-month impact of key nutrition program messages on dietary intake and food behaviours.	Mobile features: SMS	Weight-related and behavioural (diet)	Children and adolescents (females only)	Quantitative (related to RCT)<br><br>Trial name: *NEAT Girls* (ACTRN12610000330044)

Study ID	Author (Year), Country	Objectives of the paper	Technologies used	Relevant Outcomes	Target population	Methodology/Study design
90	Conroy (2011), USA	The purposes of the current study were to: 1) examine the frequency of PA self-monitoring behavior during 6 months with three self-monitoring methods paper record (PR), PDA, or PDA with daily tailored feedback message (PDA + FB); and 2) determine the associations of PA self- monitoring with adherence to PA goals, PA levels, and weight loss at 6 months in a behavioural weight loss trial.	Mobile features: PDA App<br>App name: *DietMatePro*, *CalculFit*<br>Operative system: Palm<br>Tested on: PalmOne Tungsten E2	Weight-related and behavioural (PA)	Obese adults (mostly females)	Quantitative (related to RCT)<br><br>Trial name: *SMART* (NCT00277771)
104	de Niet (2012), Netherlands	This study analyses whether self-monitoring of lifestyle behaviours through a short message service maintenance treatment (SMSMT) via mobile phones with personalized feedback positively affects weight, lifestyle behaviours and psychological well being in obese children.	Mobile features: SMS	Weight-related and behavioural (diet)	Overweight or obese children and adolescents (mostly females)	Quantitative (related to RCT)<br><br>Trial name: *NTT-CO* (ISRCTN33476574)
108	Dewar (2013), Australia	To evaluate the 24-month impact of a school-based obesity prevention program among adolescent girls living in low-income communities.	Mobile features: SMS	Weight-related and behavioural (PA, diet)	Children and adolescents (females only)	Quantitative (related to RCT)<br><br>Trial name: *NEAT Girls* (ACTRN12610000330044)
116	Duncan (2011), USA	The PDA+: A Personal Digital Assistant for Obesity Treatment randomized controlled trial (related to RCT) was designed to test whether a PDA-based behavioural intervention enhances the effectiveness of the existing group weight loss treatment program at VA Medical Centers.	Mobile features: PDA App<br>App name: not provided<br>Operative system: Not specified	Weight-related and behavioural (PA, diet)	Obese adult patients with chronic pain	Study protocol for RCT<br><br>Trial name: *MOVE2!; PDA+* (NCT00371462)
129	Fjeldsoe (2014), Australia	This trial aims to evaluate the feasibility, acceptability and efficacy of a text message-delivered extended contact intervention to enhance or maintain change in physical activity, dietary behaviour and weight loss among participants who have completed a six month Government-funded, population-based telephone coaching lifestyle program.	Mobile features: SMS	Weight-related and behavioural (PA, diet)	Adults	Study protocol for RCT<br><br>Trial name: *Get Healthy, Stay Healthy (GHSH)* (ACTRN12613000949785)
144	Fukuoka (2011), USA	The purpose of this paper is to describe the study design and protocol of a randomised controlled clinical trial that examines the efficacy of a 3-month mobile phone and pedometer based physical activity intervention and compares two different 6-month maintenance interventions.	Mobile features: App<br>App name: not provided<br>Operative system: Not specified	PA	Sedentary adults (females only)	Study protocol for RCT<br><br>Trial name: *mPED* (NCT01280812)

Study ID	Author (Year), Country	Objectives of the paper	Technologies used	Relevant Outcomes	Target population	Methodology/Study design
153	Glynn (2013), Ireland	The aim of this study is to evaluate the effectiveness of a smartphone application as an intervention to promote physical activity in primary care.	Mobile features: App (using accelerometer)<br>App name: *Accupedo*<br>Operative system: Android	Weight-related and behavioural (PA)	Adolescents and adult patients	Study protocol for RCT<br><br>Trial name: *SMART Move* (ISRCTN99944116)
154	Glynn (2014), Ireland	To evaluate the effectiveness of a smartphone application (app) to increase physical activity in primary care.	Mobile features: App (using accelerometer)<br>App name: *Accupedo*<br>Operative system: Android	Weight-related and behavioural (PA)	Adult patients (mostly females)	Quantitative (related to RCT)<br><br>Trial name: *SMART Move* (ISRCTN99944116)
166	Hagberg (2014), Sweden	The aim of this study was to make a cost-utility analysis of a dietary behavior modification treatment, compared to usual care, among lactating overweight and obese women.	Mobile features: SMS	Weight-related	Postpartum overweight or obese women	Quantitative (related to RCT)<br><br>Trial name: *LEVA* (NCT01343238)
189	Hurling (2007), UK	The aim was to evaluate the impact of a physical activity program based on the Internet and mobile phone technology provided to individuals for 9 weeks.	Mobile features: SMS	PA	Overweight or obese adults (mostly females)	Quantitative (related to RCT)<br><br>Trial name: *GetActive*
197	Joo (2010), South Korea	We evaluated two 12-week long community-based obesity control programmes in Korea. One was a visiting-type programme (V-type) (n = 515) administered by a public health centre and the other was a remote-type programme (R-type) (n = 410) utilizing an Internet website and mobile phones with a short message service.	Mobile features: SMS	Weight-related	Obese adults (mostly females)	Quantitative (cost evaluation)<br><br>Trial name: *Prodiet.co.kr*
206	Kerr (2012), Australia	This randomised controlled trial aims to evaluate the effectiveness of using a mobile device to assess dietary intake, provide tailored dietary feedback and text messages to motivate changes in fruit, vegetable and junk food consumption among young adults.	Mobile features: App (using camera) and SMS<br>App name: not provided<br>Operative system: Not specified	Weight-related and behavioural (diet)	Adults	Study protocol for RCT<br><br>Trial name: *CHAT* (ACTRN126120002508 31)
224	Knight (2014), Canada	The purpose of this study was to test the effectiveness of an mHealth study with tailored physical activity prescription targeting changes in various intensities of physical activity (e.g., exercise, sedentary behavior, or both) for improving physiological and behavioural markers of lifestyle-related disease risk.	Mobile features: App<br>App name: not provided<br>Operative system: BlackBerry<br>Tested on: BlackBerry Curve 8530	Weight-related and behavioural (PA)	Older adults (mostly females)	Quantitative (related to RCT)

Study ID	Author (Year), Country	Objectives of the paper	Technologies used	Relevant Outcomes	Target population	Methodology/Study design
233	Laing (2014), USA	To evaluate the effect of introducing primary care patients to a free smartphone app for weight loss.	Mobile features: App<br>App name: *MyFitnessPal*<br>Operative system: iOS, Android<br>Tested on: iPad	Weight-related and behavioural (diet)	Overweight or obese adult patients (mostly females)	Quantitative (related to RCT)<br><br>Trial name: *mFit* (NCT01650337)
247	Lin (2014), China	The impact of a text messaging-assisted lifestyle weight loss intervention on weight change among overweight adults in Beijing was examined.	Mobile features: SMS	Weight-related and behavioural (PA, diet)	Overweight or obese adults (mostly females)	Quantitative (related to RCT)<br><br>Trial no.: NCT01491906
251	Lombard (2010), Australia	To develop and evaluate the effectiveness of a community behavioural intervention to prevent weight gain and improve health related behaviours in women with young children.	Mobile features: SMS	Weight-related and behavioural (PA, diet)	Overweight or obese adults (females only)	Quantitative (related to RCT)<br><br>Trial name: *HeLP-her* (ACTRN12608000110381)
254	Lubans (2010), Australia	The purpose of this paper is to report the rationale, study design and baseline findings of a school-based obesity prevention program for low-active adolescent girls from disadvantaged secondary schools.	Mobile features: SMS	Weight-related and behavioural (PA, diet)	Children and adolescents (females only)	Study protocol for RCT<br><br>Trial name: *NEAT Girls* (ACTRN12610000330044)
** 273<br><br>[Also cited in Section 2a]	Mattila (2010), Finland	In the present study, we used data from an earlier study, and examined the validity of self-observed weight measures as well as behavioural patterns in those successful and unsuccessful in weight loss.	Mobile features: App<br>App name: *Wellness Diary*<br>Operative system: Symbian	Weight-related and behavioural (PA, diet)	Adults (mostly males)	Quantitative
282	Miners (2012), UK	The aim of this study is to assess the cost-effectiveness of e-learning devices as a method of promoting weight loss via dietary change.	Mobile features: App<br>App name: not provided<br>Operative system: Not specified	Diet	Obese	Quantitative
286	Monteiro (2011), Australia	This study aims to confirm if a low-cost, accessible playgroup based intervention program can improve the dietary and physical activity behaviours of mothers with young children.	Mobile features: SMS	Weight-related and behavioural (PA, diet)	Mothers of young children	Study protocol for RCT<br><br>Trial name: *PANS* (ACTRN12609000735257)
296	Newton (2009), New Zealand	To assess whether pedometers and text messaging increase physical activity in adolescents with Type 1 Diabetes.	Mobile features: SMS	Weight-related and behavioural (PA)	Child and adolescent obese patients with T1D (mostly females)	Quantitative (related to RCT)<br><br>Trial name: *RAPT* (ACTRN012605000339651)

Study ID	Author (Year), Country	Objectives of the paper	Technologies used	Relevant Outcomes	Target population	Methodology/Study design
300	Nguyen (2012), Australia	To assess the outcomes of the Loozit adolescent weight management intervention and to evaluate the effect of additional therapeutic contact 12 months into the program.	Mobile features: SMS	Weight-related and behavioural (PA, diet)	Overweight or obese adolescents (mostly females)	Quantitative (related to RCT)<br><br>Trial name: *Loozit®* (ACTRN12606000175572)
299	Nguyen (2013), Australia	This paper reports the final 24-month outcomes of a randomized controlled trial evaluating the effect of additional therapeutic contact (ATC) as an adjunct to a community-based weight-management program for overweight and obese 13–16-year-olds.	Mobile features: SMS	Weight-related and behavioural (PA, diet)	Overweight or obese adolescents (mostly females)	Quantitative (related to RCT)<br><br>Trial name: *Loozit®* (ACTRN12606000175572)
305	Norman (2013), USA	In this study, we used data from Patrick et al.'s (2009) Mobile Diet Intervention through Electronic Technology (mDIET) study to examine if fruit and vegetable intake and eating behaviours mediated the intervention effect on weight loss.	Mobile features: SMS	Weight-related and behavioural (diet)	Overweight or obese adults (mostly females)	Quantitative (related to RCT)<br><br>Trial name: *mDIET* (NCT00415870)
307	O'Malley (2014), Ireland	The primary aim of this parallel non-inferiority trial is to assess the effectiveness of an experimental smartphone application in reducing obesity at 12 months, compared to the Temple Street W82GO Healthy Lifestyles intervention.	Mobile features: App<br>App name: not provided<br>Operative system: Not specified	Weight-related and behavioural (PA)	Obese children and adolescents	Study protocol for RCT<br><br>Trial name: *W82GO* (NCT01804855)
313	Park (2009), South Korea	The present study evaluated whether an intervention using the SMS by cellular phone and Internet would affect the levels of blood pressure, body weight, waist circumference, and serum lipids of the patients with obese hypertension.	Mobile features: SMS	Weight-related	Obese adult patients with hypertension	Quantitative
312	Park (2012), South Korea	The present study evaluated whether an intervention using a short message service (SMS) by personal cellular phone and Internet would reduce cardiovascular risk factors in post-menopausal women with abdominal obesity over 12 weeks. The goal of intervention was to reduce waist circumference, body weight, and blood pressure levels.	Mobile features: SMS	Weight-related	Obese, post menopausal women	Quantitative

Study ID	Author (Year), Country	Objectives of the paper	Technologies used	Relevant Outcomes	Target population	Methodology/Study design
315	Patrick (2013), USA	We conducted a randomized controlled trial to evaluate the effectiveness of an intervention targeting this population that was offered to participants recruited through clinical sites but primarily delivered through combinations of three modalities: the web, group sessions for adolescents and parents, and short message service (SMS).	Mobile features: SMS	Weight-related and behavioural (PA)	Parents and children, at risk of T2D	Quantitative (related to RCT)<br><br>Trial name: *PACEi-DP* (NCT00412165)
319	Pellegrini (2014), USA	The Opt-IN study is a theory-guided trial using the Multiphase Optimization Strategy (MOST) to develop an optimized, scalable version of a technology-supported weight loss intervention. Objective Opt-IN aims to identify which of 5 treatment components or component levels contribute most meaningfully and cost-efficiently to the improvement of weight loss over a 6 month period.	Mobile features: App and SMS<br>App name: not provided<br>Operative system: Not specified	Weight-related and behavioural (PA, diet)	Overweight or obese adults	Study protocol for RCT<br><br>Trial name: *Opt-IN*
320	Perez-Cruzado (2013), Spain	The aim of the study will be to improve physical activity and physical condition after multimodal intervention and to analyse the promotion of adherence to physical activity through a multimodal intervention and an app intervention (mHealth).	Mobile features: App<br>App name: not provided<br>Operative system: Not specified	PA	Adults with intellectual disabilities	Study protocol for RCT<br><br>Trial name: *APPCOID* (NCT01915381)
325	Prestwich (2009), UK	The central aim of the research is to test whether the effects of implementation intentions on exercise can be strengthened by combining them with text message reminders.	Mobile features: SMS	PA	Overweight or obese adults (mostly females)	Quantitative (related to RCT)
326	Prestwich (2010), UK	To test the efficacy in promoting brisk walking of two theory-based interventions that incorporate implementation intentions and text message reminders directed at one's walking-related plans or goals.	Mobile features: SMS	Weight-related and behavioural (PA)	University students (mostly females)	Quantitative (related to RCT)
333	Recio-Rodriguez (2014), Spain	The aims of this study are to develop and validate a smartphone application, and to evaluate the effect of adding this tool to a standardized intervention designed to improve adherence to the Mediterranean diet and to physical activity. An evaluation is also made of the effect of modifying habits upon vascular structure and function, and therefore on arterial aging.	Mobile features: App<br>App name: not provided<br>Operative system: Not specified	Weight-related and behavioural (PA, diet)	Adults	Study protocol for RCT<br><br>Trial name: *EVIDENT* (NCT02016014)

Study ID	Author (Year), Country	Objectives of the paper	Technologies used	Relevant Outcomes	Target population	Methodology/Study design
336	Robbins (2013), USA	The main aim of the 5-year “Girls on the Move” group randomized trial is to evaluate the efficacy of a comprehensive school-based intervention in increasing girls’ minutes of moderate to vigorous physical activity and improving cardiovascular fitness, body mass index, and % body fat immediately post-intervention (after 17 weeks) and at 9-month post-intervention follow-up (9 months after end of intervention).	Mobile features: App<br>App name: not provided<br>Operative system: iOS<br>Tested on: iPad	Weight-related and behavioural (PA)	Children and adolescents (females only)	Study protocol for RCT<br><br>Trial name: *Girls on the Move* (NCT01503333)
341	Rompotis (2014), Australia	The primary objective of the present study was to assess the impact of messages emphasising habit processes and delivered by e-mail or SMS on factors associated with fruit and vegetable consumption.	Mobile features: SMS	Weight-related and behavioural (diet)	University students (mostly females)	Quantitative (related to RCT)
342	Rossi (2010), Italy	The aim of this multicentre, longitudinal, single-arm, pre-post comparison was to test a telemedicine system able to promote body weight reduction, nutritional education, and consumption of fresh local produce.	Mobile features: App (using camera) and SMS<br>App name: *DAI*<br>Operative system: Not specified	Weight-related and behavioural (PA, diet)	Overweight or obese adults (mostly females)	Quantitative
362	Shrewsbury (2009), Australia	This study aims to evaluate the effect of additional therapeutic contact as an adjunct to the Loozit® group program – a community-based, lifestyle intervention for overweight and lower grade obesity in adolescents. The additional therapeutic contact is provided via telephone coaching and either mobile phone SMS or electronic mail, or both.	Mobile features: SMS	Weight-related and behavioural (PA, diet)	Overweight or obese adolescents	Study protocol for RCT<br><br>Trial name: *Loozit®* (ACTRN126060001755 72)
372	Smith (2014), Australia	The aim of this paper is to report the rationale and study protocol for the 'Active Teen Leaders Avoiding Screen-time' (ATLAS) obesity prevention intervention for adolescent boys living in low-income communities.	Mobile features: App<br>App name: *Atlas*<br>Operative system: Android, iOS	Weight-related and behavioural (PA, diet)	Children and adolescents (males only)	Study protocol for RCT<br><br>Trial name: *ATLAS* (ACTRN126120009788 64)
375	Sniehotta (2012), UK	To investigate the suitability of N-of-1 randomized controlled trials (RCTs) as a means of testing the effectiveness of behavior change techniques based on self-regulation theory (goal setting and self-monitoring) for promoting walking in healthy adult volunteers.	Mobile features: SMS	PA	Normal weight and overweight university students and staff (mostly females)	Quantitative (related to RCT)

Study ID	Author (Year), Country	Objectives of the paper	Technologies used	Relevant Outcomes	Target population	Methodology/Study design
376	Soureti (2011), UK	The primary aim of the present study was to assess the effects of a fully automated planning tool and mobile text reminders on participants' reduced intake of high-fat foods and food portion sizes. A secondary aim of this study was to assess changes in other health-related behaviours not directly targeted by the intervention.	Mobile features: SMS	PA and diet	Overweight or obese adults	Quantitative (related to RCT)<br><br>Trial no.: ISRCTN61819220
381	Spring (2010), USA	The study aim is to determine which combination of two behaviour change goals - one dietary, one activity - yields greatest overall healthy lifestyle change.	Mobile features: PDA App<br>App name: *Nutribase*<br>Operative system: Not specified	PA and diet	Sedentary adults	Study protocol for RCT<br><br>Trial name: *Make Better Choices* (NCT00113672)
380	Spring (2012), USA	To test which combination of diet and activity advice maximizes healthy change, we randomized 204 adults with elevated saturated fat and low fruit and vegetable intake, high sedentary leisure time, and low physical activity to 1 of 4 treatments: increase fruit/vegetable intake and physical activity, decrease fat and sedentary leisure, decrease fat and increase physical activity, and increase fruit/vegetable intake and decrease sedentary leisure.	Mobile features: PDA App<br>App name: *Nutribase*<br>Operative system: Not specified	PA and diet	Sedentary adults (mostly females)	Quantitative (related to RCT)<br><br>Trial name: *Make Better Choices* (NCT00113672)
386	Struempler (2014), USA	To increase fruit and vegetable consumption of youth in Body Quest: Food of the Warrior, a childhood obesity prevention program.	Mobile features: App<br>App name: *Body Quest: Food of the Warrior*<br>Operative system: iOS<br>Tested on: iPad	Diet	Third grade students	Quantitative<br><br>Trial name: *Body Quest: Food of the Warrior*
387	Stuckey (2011), Canada	The Diabetes and Technology for Increased Activity (DaTA) study was designed to test the effectiveness of a lifestyle intervention driven by self-monitoring of blood glucose, blood pressure, physical activity, and weight to positively impact CVD risk factors in a medically underserviced rural population with a high incidence of metabolic syndrome.	Mobile features: App<br>App name: not provided<br>Operative system: BlackBerry<br>Tested on: BlackBerry Curve 8300	Weight-related and behavioural (PA)	Obese patients with metabolic syndrome (mostly females)	Quantitative<br><br>Trial name: *DaTA*
390	Suggs (2013), Switzerland	The purpose of this study was to test the effects of adding weekly SMS communication to weekly email communication on physical activity of employees of academic workplaces in the UK.	Mobile features: SMS	Weight-related and behavioural (PA)	University and college employees (mostly females)	Quantitative (related to RCT)

Study ID	Author (Year), Country	Objectives of the paper	Technologies used	Relevant Outcomes	Target population	Methodology/Study design
402	Turk (2013), USA	This study examined the effect of participant feedback frequency on weight loss and determined if this effect was mediated by adherence to self-monitoring in a behavioural weight-loss trial conducted in the USA. This study is a secondary analysis of the 6-month data from the Self-Monitoring and Recording using Technology (SMART).	Mobile features: PDA App<br>App name: *DietMatePro*, *CalculFit*<br>Operative system: Palm<br>Tested on: PalmOne Tungsten E2	Weight-related	Obese adults (mostly females)	Quantitative (related to RCT)<br><br>Trial name: *SMART* (NCT00277771)
425	Wedderkopp (2012), Denmark	It is the purpose of this paper to describe the organization of the CHAMPS- study DK, the main research questions, and the main methods of data collection. The main aim of this study is to describe differences in development of health and motor performance over time in relation to type of school and other background variables.	Mobile features: SMS	Weight-related and behavioural (PA)	Children	Study protocol for Quantitative study<br><br>Trial name: *The CHAMPS-study DK*
430	Wharton (2014), USA	The authors assessed the use of a popular smartphone app for dietary self-monitoring and weight loss by comparing it with traditional diet counselling and entry methods.	Mobile features: App<br>App name: *Lose it!*<br>Operative system: iOS<br>Tested on: iPhone	Weight-related and behavioural (diet)	Adults (mostly females)	Quantitative (related to RCT)
446	Yon (2007), USA	The purpose of this study was to investigate whether the use of a PDA for dietary self-monitoring would improve self-monitoring frequency and subsequent weight loss at the end of a 24-week behavioural weight loss program.	Mobile features: PDA App and SMS<br>App name: *Calorie King's Diet Diary*<br>Operative system: Palm<br>Tested on: PalmZire 21	Weight-related	Overweight or obese adults (mostly females)	Quantitative
Web 2.0 technologies (6 studies, 9%)<br>***Study design: Quantitative (RCTs) (6/6, 100%, of which 4 study protocols)***						
64	Caperchione (2014), Canada	The aim of this study is to investigate the difference in physical activity behaviour between individuals using a traditional Web 1.0 website with those using a novel Web 2.0 website.	Web 2.0 features: Custom social networking site	Weight-related and behavioural (PA)	Adults	Study protocol for RCT<br><br>Trial name: *WALK 2.0* (ACTRN12611000253909)
77	Chee (2014), Malaysia	This study aimed to ascertain the effects of a Facebook-based physical activity intervention on improvements in step counts and metabolic syndrome.	Web 2.0 features: Facebook group	Weight-related and behavioural (PA)	Employees with metabolic syndrome (mostly females)	Quantitative (related to RCT)

Study ID	Author (Year), Country	Objectives of the paper	Technologies used	Relevant Outcomes	Target population	Methodology/Study design
159	Greene (2013), USA	Using a randomized trial, we assessed the impact of a health-oriented OSN with accelerometer and scales on participant's physical activity, weight, and clinical indicators.	Web 2.0 features: Custom social networking site	Weight-related and behavioural (PA)	Adults (mostly females)	Quantitative (related to RCT)
205	Kernot (2013), Australia	This study aims to determine the effectiveness of the Mums Step it Up Program	Web 2.0 features: Facebook app	Weight-related and behavioural (PA)	Post-partum women	Study protocol for RCT<br><br>Trial name: *Mums Step it Up* (ACTRN12613000069752)
227	Kolt (2013), Australia	The Walk 2.0 project is a 3-arm randomised controlled trial investigating the effects of "next generation" web-based applications on engagement, retention, and subsequent physical activity behaviour change.	Web 2.0 features: Custom social networking site	Weight-related	Adults	Study protocol for RCT<br><br>Trial name: *WALK 2.0* (ACTRN12611000157976)
323	Po'e (2013), USA	This paper describes the design, methodology, and proposed evaluation of the GROW Trial.	Web 2.0 features: Custom social networking site, blog, Facebook app	Weight-related and behavioural (PA, diet)	Children and adolescents	Study protocol for RCT<br><br>Trial name: *GROW*
Mobile and Web 2.0 technologies (9 studies, 13%)<br>***Study design: Quantitative (related to RCT) (9/9, 100%, of which 6 study protocols)***						
117	Duncan (2012), Australia	The aim of the ManUp Study is to examine the effectiveness of an IT-based intervention to increase the physical activity and nutrition behaviour and literacy in middle-aged males (Aged 35–54 years).	Mobile features: App and SMS<br>App name: *ManUp*<br>Operative system: Not specified<br><br>Web 2.0 features: Social media group challenges	Weight-related and behavioural (PA, diet)	Middle-aged males	Study protocol for RCT<br><br>Trial name: *ManUp* (ACTRN12611000081910)
175	Hebden (2013), Australia	This paper reports the protocol for a RCT aimed at (1) testing the efficacy of the TXT2BFiT program by comparing changes in body weight and selected dietary, physical activity and sedentary behaviours among young adults aged 18 to 35 years with changes in a control group, and (2) evaluating program reach, costs, implementation and participant engagement to inform the potential future translation of the program into the broader community.	Mobile features: App and SMS<br>App name: not provided<br>Operative system: Not specified<br><br>Web 2.0 features: Blogs, discussion forums	Weight-related and behavioural (PA, diet)	Overweight or obese adults	Study protocol for RCT<br><br>Trial name: *TXT2BFiT* (ACTRN12612000924853)

Study ID	Author (Year), Country	Objectives of the paper	Technologies used	Relevant Outcomes	Target population	Methodology/Study design
213	Kharrazi (2011), USA	The purpose of this study is to review and propose a conceptual framework that increases physical activity by implementing a behavioural intervention using patient health records in a sample target population of college freshmen.	Mobile features: App<br>App name: *ActiPed*<br>Operative system: Not specified<br><br>Web 2.0 features: Facebook app	Weight-related and behavioural (PA)	Adults	Study protocol for RCT
266	Marshall (2013), Australia	This RCT will evaluate the efficacy, feasibility and acceptability, cost-effectiveness, mediators and moderators of the program.	Mobile features: SMS<br><br>Web 2.0 features: Facebook group	PA	Adult mothers	Study protocol for RCT<br><br>Trial name: *MobileMums* (ACTRN12611000481976)
314	Patrick (2014), USA	To describe the theoretical rationale, intervention design, and clinical trial of a two-year weight control intervention for young adults deployed via social and mobile media.	Mobile features: App and SMS<br>App name: *Goal Getter*<br>Operative system: iOS<br>Tested on: iPhone<br><br>Web 2.0 features: Facebook page, blogs	Weight-related and behavioural (PA, diet)	Overweight or obese adults (mostly females)	Study protocol for RCT<br><br>Trial name: *SMART* (NCT01200459)
318	Pellegrini (2012), USA	To test the feasibility and efficacy of this mobile technology-supported strategy, we have developed the E-Networks Guiding Adherence to Goals in Exercise and Diet (ENGAGED) study, a randomized controlled trial (related to RCT) that uses a theory-guided, technology-supported weight loss program.	Mobile features: App<br>App name: *ENGAGED*; *Calorie King*<br>Operative system: Android<br>Tested on: Motorola Droid<br><br>Web 2.0 features: Custom social networking site	Weight-related and behavioural (PA, diet)	Obese adults	Study protocol for RCT<br><br>Trial name: *ENGAGED* (NCT01051713)
404	Turner-McGrievy (2009), USA	A 12-week RCT was conducted. The hypothesis of this study is that a weight-loss podcast designed with health behavior theories will produce greater weight loss than a currently available podcast that is not theory based.	Mobile features: MP3 Player<br><br>Web 2.0 features: Podcast	Weight-related and behavioural (PA, diet)	Overweight or obese adults (mostly females)	Quantitative (related to RCT)<br><br>Trial name: *Pounds off Digitally* (NCT00771095)
408	Turner-McGrievy (2011), USA	The objective of our study was to examine whether a combination of podcasting, mobile support communication, and mobile diet monitoring can assist people in weight loss.	Mobile features: App<br>App name: *FatSecret Calorie Counter*<br>Operative system: Android, Blackberry, iOS<br><br>Web 2.0 features: Podcast, Twitter	Weight-related and behavioural (PA, diet)	Overweight or obese adults (mostly females)	Quantitative (related to RCT)<br><br>Trial name: *Mobile Pounds off Digitally* (NCT01139255)

Study ID	Author (Year), Country	Objectives of the paper	Technologies used	Relevant Outcomes	Target population	Methodology/Study design
407	Turner-McGrievy (2014), USA	The purpose of this paper is to examine the use of mobile devices and traditional desktop computer access methods in two mobile weight loss interventions. The goal of this study is to examine how participants accessed study components and to examine the effects of type of device (mobile vs. non- mobile) with engagement and weight loss.	Mobile features: App<br>App name: *FatSecret Calorie Counter*<br>Operative system: Android, Blackberry, iOS<br><br>Web 2.0 features: Podcast	Weight-related	Overweight or obese adults (mostly females)	Quantitative (related to RCT)<br><br>Trial names: *Mobile Pounds off Digitally (Mobile POD); Pounds off Digitally (POD)*
1.4) Process evaluation/Causal-comparative (20 studies)						
Mobile technologies (6 studies, 30%)<br>***Study design: Quantitative (6/6, 100%, of which 5 linked to subsequent RCT)***						
102	David (2014), USA	Using day-to-day measurements obtained over 84 days, we examined the relationship between state self-efficacy and PA. Postmenopausal women (n = 71) participated in a 12-week PA intervention administered via cell phone and monitored their daily PA using a pedometer.	Mobile features: App (using microphone) and SMS<br>App name: not provided<br>Operative system: Not specified	PA	Post-menopausal women	Quantitative (related to RCT)
131	Fjeldsoe (2013), Australia	To explore whether improvements in physical activity following the MobileMums intervention were mediated by changes in Social Cognitive Theory (SCT) constructs targeted in the intervention (barrier self efficacy, goal setting skills, outcome expectancy, social support, and perceived environ- mental opportunity for exercise). This paper also examined if the mediating constructs differed between initial (baseline to 6 weeks) and overall (baseline to 13 weeks) changes in physical activity.	Mobile features: SMS	PA	Sedentary mothers	Quantitative (related to RCT)<br><br>Trial name: *MobileMums* (ACTRN126110004819 761656)
185	Hofmann (2014), Germany	The literature on dieting has sparked several debates over how restrained eaters differ from unrestrained eaters in their self-regulation of healthy and unhealthy food desires and what distinguishes successful from unsuccessful dieters.	Mobile features: App<br>App name: not provided<br>Operative system: BlackBerry	Weight-related	Restrained and unrestrained eaters (mostly females)	Quantitative
352	Sevick (2010), USA	This study examined modifiable and non-modifiable factors associated with technology-based self-monitoring.	Mobile features: PDA App<br>App name: *BalanceLog*<br>Operative system: Palm<br>Tested on: PalmOne Tungsten E2	Diet	Overweight or obese adults, with T2D (mostly females)	Quantitative (related to RCT)<br><br>Trial name: *ENHANCE*

Study ID	Author (Year), Country	Objectives of the paper	Technologies used	Relevant Outcomes	Target population	Methodology/Study design
361	Shay (2009), USA	The purpose of this article is to report the findings on food/exercise diary preference in relation to adherence patterns, self-efficacy and change in body composition.	Mobile features: PDA App<br>App name: *Calorie King's Diet Diary*<br>Operative system: Palm<br>Tested on: PalmOne Tungsten E2	Weight-related and behavioural (PA, diet)	Overweight or obese soldiers<br>(mostly males)	Quantitative (related to RCT)<br><br>Trial name: *ShipShape*
422	Wang (2012), USA	This study examined the mediating role of adherence to self-monitoring of diet and physical activity on weight loss in a behavioural weight-loss trial testing the use of personal digital assistants (PDA) for self-monitoring.	Mobile features: PDA App<br>App name: *DietMatePro*, *CalculFit*<br>Operative system: Palm<br>Tested on: PalmOne Tungsten E2	Weight-related and behavioural (diet)	Obese adults (mostly females)	Quantitative (related to RCT)<br><br>Trial name: *SMART* (NCT002777711570)
Web 2.0 technologies (8 studies, 40%)<br>***Study design: Mixed (1/8, 13%, linked to subsequent RCT); Quantitative (7/8, 87%, of which 2 linked to subsequent RCT)***						
73	Cavallo (2013), USA	The primary objective of the current study was to examine the relationship between social support and physical activity within the theory of planned behavior (TPB) theoretical framework.	Web 2.0 features: Facebook group	PA	Adults (females only)	Quantitative (related to RCT)<br><br>Trial name: *INSHAPE* (NCT014217581618)
75	Cavallo (2014), USA	This study aims to examine Facebook engagement among participants in the online social networking arm of a randomized controlled physical activity promotion trial (n=67).	Web 2.0 features: Facebook group	PA	Adults (females only)	Mixed (related to RCT)<br><br>Trial name: *INSHAPE* (NCT014217581618)
82	Chomutare (2014), Norway	The goal of this study is to determine online interaction behaviours that predict weight loss performance. The problem is modelled as a binomial classification task for predicting whether a patient would lose significant weight, based on analysis of two obesity online communities, Obesity(50+) and Obesity(Surgery).	Web 2.0 features: Custom social networking site	Weight-related	Obese adult patients	Quantitative
162	Groenewegen (2012), Netherlands	This paper discusses the impact of online social networks as means to motivate people to become more physically active.	Web 2.0 features: Custom social networking site	Behavioural (PA)	Adults (mostly males)	Quantitative
167	Hales (2014), USA	The purpose of this study was to examine whether different types of posts differentially affect participant engagement and if engagement with social media enhances weight loss. Data are a sub-analysis from a randomized weight loss study with a 4-month follow- up support period via private Facebook groups and monthly meetings.	Web 2.0 features: Facebook group	Weight-related	Overweight and obese adults (mostly females)	Quantitative (related to RCT)<br><br>Trial name: *New DIETs*

Study ID	Author (Year), Country	Objectives of the paper	Technologies used	Relevant Outcomes	Target population	Methodology/Study design
190	Hwang (2014), USA	To test the hypothesis that the frequency of using social media tools (structural support) is directly related to perceptions of Encouragement, Information and Shared Experiences support (functional support).	Online support group and Mobile app (*SparkPeople*)	Functional and structural support, encouragement, information	General pop.	Quantitative
260	Ma (2010), USA	This paper provides an empirical analysis of a health OSN, which allows its users to record their foods and exercises, to track their diet progress towards weight-change goals, and to socialize and group with each other for community support.	Web 2.0 features: Custom social networking site	Weight-related	Adults	Quantitative
389	Sugano (2011), Japan	In this paper, we verify the behavior of Social Networking Service (SNS) users who focus on diet and clarify points for utilizing such a SNS more effectively.	Web 2.0 features: Yahoo Diet Diary, a commercial social networking site	Weight-related and behavioural (diet)	Adults	Quantitative
Mobile and Web 2.0 technologies (7 studies, 30%) ***Study design: Mixed (1/7, 14%, linked to subsequent RCT); Quantitative (6/7, 86%, of which 5 linked to subsequent RCT)***						
30	Ba (2013), USA	This research analyses data collected from a digital health community to examine what mechanisms can help motivate people.	Mobile features: App<br>App name: *DailyBurn.com; FoodScanner; Push-up Wars*<br><br>Web 2.0 features: Custom social networking site	PA	Adults (mostly males)	Quantitative
105	Demment (2014), USA	The objectives of this study were to (1) characterize how pregnant women engaged with features of an online intervention to prevent excessive gestational weight gain, (2) identify demographic and weight status subgroups of women within the sample, and (3) examine differences in use of intervention features across the demographic and weight status subgroups.	Mobile features: SMS<br><br>Web 2.0 features: Blogs	Weight-related	Overweight adults (females only)	Quantitative (related to RCT)<br><br>Trial name: *e-Moms Roc* (NCT013315641968)

Study ID	Author (Year), Country	Objectives of the paper	Technologies used	Relevant Outcomes	Target population	Methodology/Study design
226	Ko (2014), USA	This study investigated whether constructs of social cognitive theory and information processing theories (IPTs) mediate the effect of a podcast intervention on weight loss among overweight individuals. Data are from Pounds off Digitally, a study testing the efficacy of two weight loss podcast interventions (control podcast and theory-based podcast).	Mobile features: MP3 Player<br><br>Web 2.0 features: Podcast	Weight-related	Overweight or obese adults (mostly females)	Quantitative (related to RCT)<br><br>Trial name: *Pounds off Digitally (POD)* (NCT007710951724)
406	Turner-McGrievy (2013), USA	This study investigated what types of social support were utilized, predictors of Twitter engagement, and the relationship between Twitter engagement and weight loss. There were three primary aims of the statistical analyses as part of this paper. The first aim was to examine Twitter engagement. The second aim was to examine the relationship between Twitter engagement and weight loss. The final aim was to examine the types of social support provided on Twitter by participants.	Mobile features: MP3 Player<br><br>Web 2.0 features: Podcast	Weight-related and behavioural (PA, diet)	Overweight or obese adults (mostly females)	Mixed (related to RCT)<br><br>Trial name: *Mobile Pounds off Digitally (Mobile POD)* (NCT011392551569)
403	Turner-McGrievy (2013), USA	The purpose of this study was to assess the relationship between diet (mobile app, website, or paper journal) and PA (mobile app vs. no mobile app) self-monitoring and dietary and PA behaviours. This study is a post hoc analysis of a 6-month randomized weight loss trial among 96 overweight men and women.	Mobile features: MP3 Player<br><br>Web 2.0 features: Podcast	Weight-related and behavioural (PA, diet)	Overweight or obese adults (mostly females)	Quantitative (related to RCT)<br><br>Trial name: *Mobile Pounds off Digitally (Mobile POD)* (NCT011392551569)
405	Turner-McGrievy (2014), USA	The present study compared baseline characteristics of participants enrolling in two different 6-month behavioural weight loss studies: the mobile Pounds Off Digitally (mPOD) study and the New Dietary Interventions to Enhance the Treatments for weight loss (New DIETs) study.	Mobile features: MP3 Player<br><br>Web 2.0 features: Podcast	PA and diet	Overweight or obese adults (mostly females)	Quantitative (related to RCT)<br><br>Trial names: *Mobile Pounds off Digitally (Mobile POD); New DIETs*
1.5) Design and Feasibility (46 studies)						
Mobile technologies (33 studies, 72%)<br>***Study design: Qualitative (4/33, 12%, of which 1 linked to subsequent RCT); Mixed (20/33, 61%, of which 5 linked to subsequent RCT); Quantitative (9/33, 27%)***						

Study ID	Author (Year), Country	Objectives of the paper	Technologies used	Relevant Outcomes	Target population	Methodology/Study design
7	Ahtinen (2013), Finland	This paper reports the findings of a constructive design research study exploring mobile wellness applications in two different contexts: Finland and India. The study arrived at four design principles for motivating users to engage in physical exercise	Mobile features: App and SMS<br>App name: *Wellness Diary, Magical Gadget*<br>Operative system: Symbian	PA	Adults	Qualitative
11	Almomani (2014), Jordan	In this paper we describe a new persuasive technology designed to inspire kids to apply healthy eating habits, as well as to encourage them to be physically active.	Mobile features: App<br>App name: *Mobile Approach to Challenge Obesity (MACO)*<br>Operative system: Not specified	PA and diet	Children and adolescents	Quantitative
13	Anderson (2007), UK	This paper explores the potential for use of an unaugmented commodity technology—the mobile phone—as a health promotion tool. We describe a prototype application that tracks the daily exercise activities of people, using an Artificial Neural Network (ANN) to analyse GSM cell signal strength and visibility to estimate a user's movement.	Mobile features: App (using accelerometer and GPS)<br>App name: *Shakra*<br>Operative system: Windows Mobile	PA	Adults (mostly females)	Mixed
17	Anwar (2013), USA	We elicit the requirements of a system to address the research gaps. Third, we design and develop a dietary app for android platform that implements many of the elicited requirements.	Mobile features: App<br>App name: *Kalico*<br>Operative system: Android	Diet	General pop.	Mixed
23	Arsand (2008), Norway	This article focuses on secondary prevention for people with Type 2 diabetes. The system presented may also hold potential for primary prevention, i.e., to be used by healthy people who want to reduce their chances for developing health problems.	Mobile features: App (using accelerometer and Bluetooth)<br>App name: not provided<br>Operative system: Windows Mobile	PA	Overweight or obese adult patients with T2D	Mixed
24	Arteaga (2012), USA	In this paper we discuss design guidelines for game-based applications that motivate teenagers to do physical activity. We describe an application developed based on these guidelines and results from a user study we ran using this application.	Mobile features: App<br>App name: not provided<br>Operative system: iOS	PA	Adolescents (males only)	Mixed

Study ID	Author (Year), Country	Objectives of the paper	Technologies used	Relevant Outcomes	Target population	Methodology/Study design
34	Bartley (2013), USA	In this paper, we aim to provide a complete physical activity system with continual motivation through our exergaming model, developed into an application. This game provides a large selection of exercises using sensors widely available in smartphones, allowing physical activity recording on a wide selection of devices. We also provide several channels of feedback for both long and short-term drive, which encourages users to exercise regularly.	Mobile features: App (using accelerometer and GPS)<br>App name: *World of Workout*<br>Operative system: Android	PA	University students	Mixed
63	Buttussi (2010), Italy	We created a context- aware, user-adaptive fitness game for mobile phones, which trains and motivates users to jog outdoors at the correct intensity.	Mobile features: App (using GPS)<br>App name: *Monster & Gold*<br>Operative system: Not specified	PA	Adults (mostly males)	Mixed
65	Carter (2013), UK	This paper describes the MMM app, discusses its development and outlines plans for further investigation.	Mobile features: App (using camera)<br>App name: *My Meal Mate*<br>Operative system: Android	Diet	University students (mostly females)	Qualitative (related to RCT)<br>Trial name: *My Meal Mate* (NCT01744535)
69	Casey (2014), Ireland	To explore patients' views and experiences of using smartphones to promote physical activity in primary care.	Mobile features: App (using accelerometer)<br>App name: *Accupedo*<br>Operative system: Android	Weight-related and behavioural (PA)	Adolescent and adult patients<br>(mostly females)	Qualitative (related to RCT)<br>Trial name: *SMART Move* (ISRCTN9994116)
93	Consolvo (2008), USA	We have developed a system, UbiFit Garden, which uses these technologies and a personal, mobile display to encourage physical activity.	Mobile features: App<br>App name: *UbiFit Garden*<br>Operative system: Not specified	PA	Adults	Mixed
140	Fujiki (2008), USA	This article describes research that aims to encourage physical activity through a novel pervasive gaming paradigm.	Mobile features: App<br>App name: *NEAT-o-Games*<br>Operative system: Palm<br>Tested on: Palm Treo 700w/wx	Weight-related and behavioural (PA)	University students and staff	Mixed
149	Gasser (2006), Switzerland	We performed a field study that compares a mobile application on a Smartphone with a functionally equivalent web application. In our study, we explored its persuasive power in a healthy lifestyle coaching application.	Mobile features: App<br>App name: *eDIM*<br>Operative system: Not specified	PA and diet	Adolescents and adults	Mixed (related to RCT)

Study ID	Author (Year), Country	Objectives of the paper	Technologies used	Relevant Outcomes	Target population	Methodology/Study design
** 158<br><br>[Also cited in Section 2a]	Greeff (2013), New Zealand	In this research we developed a smartphone application, which assists users with learning and monitoring exercises. A key feature of the application is a novel algorithm for analysing accelerometer data and automatically counting repetitive exercises.	Mobile features: App (using accelerometer)<br>App name: *My Personal Trainer*<br>Operative system: iOS<br>Tested on: iPhone	PA	Adolescents and adults	Quantitative
177	Hebden (2012), Australia	This paper describes the process of developing four apps aimed at modifying key lifestyle behaviours associated with weight gain during young adulthood, including physical activity, and consumption of take-out foods (fast food), fruit and vegetables, and sugar-sweetened drinks.	Mobile features: App<br>App name: *ePass*, *eVIP*, *eTIYP*, *eSIYP*<br>Operative system: Python	PA and diet	Overweight or obese adults	Quantitative
193	Järvinen (2008), Finland	HyperFit is an Internet service for personal management of nutrition and exercise. It provides tools for promoting healthy diet and physical activity. The principle of the service is to mimic the process of personal nutrition counselling.	Mobile features: App (using camera)<br>App name: *HyperFit*<br>Operative system: Symbian	PA and diet	Patients and caregivers	Mixed
211	Khan (2012), South Korea	In this paper, we present the preliminary results of our study where an accelerometer enabled smart phone was used to recognize a person's daily physical activities using activity acceleration signals. This knowledge was then used to suggest people minor changes in their daily routine, through text-based interventions, that might impact their daily energy expenditure positively.	Mobile features: App (using accelerometer)<br>App name: not provided<br>Operative system: Android<br>Tested on: Samsung Galaxy S2	PA	Adults	Quantitative
222	Kirwan (2013), Australia	This article describes the development and formative evaluation of a smartphone app associated with a physical activity promotion website.	Mobile features: App<br>App name: *iStepLog*<br>Operative system: iOS	PA	Adults	Mixed
231	Kurdi (2012), Saudi Arabia	The paper describes the complete process beginning from the software requirements gathering in section II, system design and architecture in section III, implementation in section IV and ending with testing and evaluating the fully running application in section V. The summary and conclusions of the papers are presented in section VI.	Mobile features: App<br>App name: *myPDA*<br>Operative system: iOS	Diet	General pop.	Quantitative

Study ID	Author (Year), Country	Objectives of the paper	Technologies used	Relevant Outcomes	Target population	Methodology/Study design
234	Lane (2014), China	In this article, we present the next generation of the BeWell smartphone wellbeing app, which monitors user behavior along three health dimensions, namely sleep, physical activity, and social interaction. We present the evaluation of the system and results from a user study.	Mobile features: App (using accelerometer and microphone)<br>App name: *BeWell+*<br>Operative system: Android	PA	University students and staff<br>(mostly males)	Mixed (related to RCT)
245	Limam (2014), Qatar	We explored ways in which mobile technologies can be adapted to meet environmental and cultural norms and thereby support individuals in their effort to lose weight. In this paper, we examine the case of the Middle East through the example of Qatar. In this paper, we briefly describe our application, which can be used by any user who can read English or Arabic.	Mobile features: App and SMS<br>App name: not provided<br>Operative system: Android	Weight-related and behavioural (PA, diet)	Adults (females only)	Quantitative
249	Lin (2011), Netherlands	This paper presents the design, implementation and evaluation of a context-aware recommendation system that promotes the adoption of a healthy and active lifestyle.	Mobile features: App (using GPS) and SMS<br>App name: *Motivate*<br>Operative system: Android	PA	Adults (mostly males)	Quantitative
246	Lin (2012), USA	In this paper, we present the next generation of the BeWell smartphone health app, which continuously monitors user behavior along three distinct health dimensions, namely sleep, physical activity, and social interaction. BeWell promotes improved behavioural patterns via feedback rendered as an ambient display on the smartphone's wallpaper.	Mobile features: App (using accelerometer and microphone)<br>App name: *BeWell+*<br>Operative system: Android	PA	University students and staff<br>(mostly males)	Mixed (related to RCT)
256	Lubans (2014), Australia	The primary objective of this paper is to describe the development and implementation of a smartphone app designed to support the delivery of the Active Teen Leaders Avoiding Screen- time (ATLAS) obesity prevention program (21). A secondary objective is to explore participants' perceptions of the program in general.	Mobile features: App<br>App name: *Atlas*<br>Operative system: Android, iOS	PA	Low-income children and adolescents at risk of obesity<br>(males only)	Mixed (related to RCT)<br>Trial name: *ATLAS*<br>(ACTRN12612000978864)

Study ID	Author (Year), Country	Objectives of the paper	Technologies used	Relevant Outcomes	Target population	Methodology/Study design
263	Mansar (2012), Qatar	This paper introduces a context-appropriate mobile application for sustainable weight loss. In this paper, we discuss contributions in the literature for technology-based weight loss support. We design a mobile application that leverages three strategies from proven behaviour change theories (increasing awareness of the aims of dieting, fostering motivation and self-efficacy, and impacting dieters' attitudes).	Mobile features: App and SMS<br>App name: not provided<br>Operative system: Android	Diet	General pop. (females only)	Mixed
272	Mattila (2008), Finland	We developed the Wellness Diary (WD) concept for personal wellness management based on the philosophy of CBT. The objective of this paper is to describe the WD concept and to present two separate studies conducted with the WD. In the first study, the WD implementation for weight management was studied, and in the second study, an implementation for general wellness management was used. Our main focus in this paper will be on the usage, usability, and acceptance of the concept and its implementations.	Mobile features: App and SMS<br>App name: *Wellness Diary*<br>Operative system: Symbian<br>Tested on: Nokia S60	Weight-related and behavioural (PA, diet)	Overweight or obese adults	Mixed
271	Mattila (2010), Finland	In this paper, we present concepts for improving Wellness Diary. We first present findings from earlier studies and analyse their implications to future WD design. We then present design factors derived from psychological theories and propose improvements to the WD concept.	Mobile features: App and SMS<br>App name: *Wellness Diary*<br>Operative system: Symbian<br>Tested on: Nokia S60	PA and diet	General pop.	Qualitative

Study ID	Author (Year), Country	Objectives of the paper	Technologies used	Relevant Outcomes	Target population	Methodology/Study design
288	Mukhtar (2013), Pakistan	The purpose of the current research is to find how we can effectively use mobile phone technology to raise awareness about sedentary behavior in users and how we can persuade users to do physical activities. For this purpose, we developed a mobile application. We describe different mobile application prototypes and experiments that were conducted on adult users for preventing sedentary behavior in order to promote physical activities among adults. Our final prototype that is able to motivate the users to take corrective actions, after detecting prolonged sedentary behavior, uses an adaptive model based on each user's progress.	Mobile features: App (using accelerometer and GPS)<br>App name: *Sedentaware*<br>Operative system: Android<br>Tested on: Samsung GT-I9100	PA	University students and staff<br>(mostly males)	Quantitative
** 401<br><br>[Also cited in Section 2a]	Tsai (2006), USA	In this paper, we describe the Patient-Cantered Assessment and Counseling Mobile Energy Balance (PmEB) cell phone application that allows users to self-monitor caloric balance in real time. We developed and applied a four-phase iterative research and development methodology. We conducted a usability study and a preliminary feasibility study.	Mobile features: App<br>App name: *PmEB*<br>Operative system: Java<br>Tested on: Nokia 6600, Motorola V300, Motorola RAZR V3xx	PA and diet	Overweight or obese adults (mostly females)	Mixed
413	van der Weegen (2014), Netherlands	In this study the usability of the tool was evaluated by technology experts and end users (people with chronic obstructive pulmonary disease or type 2 diabetes, with ages from 40–70 years), to improve the user interfaces and content of the tool.	Mobile features: App (using accelerometer)<br>App name: *It's LiFe!*<br>Operative system: Android<br>Tested on: Samsung Galaxy Ace	PA	Overweight or obese adult patients with COPD or T2D and experts	Mixed
418	Verwey (2012), Netherlands	To examine user requirements and to evaluate the usability of the secured website, in order to increase the probability of effective use by nurses.	Mobile features: App (using accelerometer)<br>App name: *It's LiFe!*<br>Operative system: Android<br>Tested on: Samsung Galaxy Ace	PA	Patients and caregivers (females only)	Mixed
447	Youm (2014), South Korea	The purpose of this study was to provide motivation for monitoring and improving harmful lifestyle habits that influence the health of individuals, with the help of a mobile application that analyses lifestyles. In addition, to evaluate the developed mobile application, the study investigated the changes in health and lifestyle awareness before and after using the application.	Mobile features: App<br>App name: not provided<br>Operative system: Android	PA and diet	Adults (mostly females)	Quantitative

Study ID	Author (Year), Country	Objectives of the paper	Technologies used	Relevant Outcomes	Target population	Methodology/Study design
451	Yuzhong (2011), Netherlands	This paper presents the practices of a research aiming at the design of a context-aware recommendation system that promotes the adoption of a healthy and active lifestyle. A Smartphone application that provides personalized and contextualized advice on physical activities was developed. The goal of our endeavour is to find out whether this recommendation system can generate motivating advice at an appropriate time and location by taking environment, weather, user location and agenda into account.	Mobile features: App (using GPS) and SMS<br>App name: *Motivate*<br>Operative system: Android	PA	Adults (mostly males)	Mixed
Web 2.0 technologies (4 studies, 9%)<br>***Study design: Mixed (2/4, 50%, of which 1 linked to subsequent RCT); Quantitative (2/4, 50%, of which 2 linked to subsequent RCT)***						
32	Baghaei (2011), New Zealand	This article reports the results of a live user study aimed at investigating how user profile representation and system-assigned tasks influence users engagement with the system and change their attitude toward a healthy lifestyle.	Web 2.0 features: Custom social networking site	PA	Adolescents and adults	Quantitative (related to RCT)
137	Freyne (2010), Australia	This paper presents results of a three-week live user evaluation of an online social networking system designed for families who wish to achieve and maintain a healthy lifestyle.	Web 2.0 features: Custom social networking site	PA	Adults	Quantitative (related to RCT)
201	Kamal (2014), Canada	This paper presents an online social network for health behaviour change that was designed from a theoretical foundation. In this paper, we present the design strategies in an online social network for health behaviour change that will promote socialization.	Web 2.0 features: Custom social networking site	Diet	Adults (mostly females)	Mixed (related to RCT)
248	Lin (2012), Taiwan	To understand how different motivation factors affect user behavior through social media, a healthcare compliance website with built-in behavior analyses was constructed to conduct experiments. This study proposes a social networking site leveraging gamification as an information-based intervention to improve the compliance with chronic disease prevention prescriptions.	Web 2.0 features: Custom social networking site	PA and diet	Patients and caregivers	Mixed
Mobile and Web 2.0 technologies (9 studies, 20%)<br>***Study design: Qualitative (1/9, 11%); Mixed (6/9, 67%, of which 1 linked to subsequent RCT); Quantitative (2/9, 22%)***						

Study ID	Author (Year), Country	Objectives of the paper	Technologies used	Relevant Outcomes	Target population	Methodology/Study design
46	Bielik (2012), Slovakia	We have created a solution, which encourages a healthier lifestyle and motivates to participate in regular physical activity. We conducted several experiments evaluating the individual parts of the system as well as the whole concept in cooperation with doctors and real users, which yielded encouraging results.	Mobile features: App (using accelerometer and GPS)<br>App name: *Move2Play*<br>Operative system: Windows Mobile<br><br>Web 2.0 features: Custom social networking site	PA	Children and adolescents	Qualitative
95	Cornejo (2012), Mexico	Building upon our prior work and using the results from 6 participatory design sessions, we present the design of two ubiquitous exergames.	Mobile features: App (using camera)<br>App name: *GuessMyCaption, TakeAPhoto*<br>Operative system: Not specified<br><br>Web 2.0 features: Custom social networking site	PA	Older adults (females only)	Mixed
160	Grimes (2008), USA	We developed EatWell, a system that allows people to use their cell phones to create voice memories describing how they have tried to eat healthfully in their neighbourhoods (e.g., at local restaurants) and listen to the memories that others have created. In this paper, we describe the results of our field trial of EatWell, specifically characterizing how our participants were able to craft stories that were both emotive and culturally-relevant, the challenges that arose in creating these memories and finally how sharing these stories facilitated a sense of community empowerment.	Mobile features: App (using microphone)<br>App name: *EatWell*<br>Operative system: Not specified<br><br>Web 2.0 features: Online support groups	Diet	Adults (mostly females)	Mixed
184	Hingle (2013), USA	The purpose of this study was to test the feasibility and acceptability of a popular social media software application (Twitter) to capture young adults' dietary behavior and reasons for eating. A secondary aim was to visualize data from Twitter using a novel analytic tool designed to help identify relationships among dietary behaviours, reasons for eating, and contextual factors.	Mobile features: App<br>App name: not provided<br>Operative system: Not specified<br><br>Web 2.0 features: Twitter	Diet	Adults, gender not specified)	Quantitative
194	Jen (2010), Taiwan	The aim of the study was to explore the factors that affect college students' intention to adopt the program for managing their weight.	Mobile features: SMS<br><br>Web 2.0 features: Custom social networking site	PA and diet	College students (mostly males)	Quantitative<br><br>Trial name: *We Care for You Virtual Community (WCU VC)*

Study ID	Author (Year), Country	Objectives of the paper	Technologies used	Relevant Outcomes	Target population	Methodology/Study design
289	Mulas (2013), Italy	This paper presents a persuasive web application for sport and health, designed to motivate people in their exercising activity. The innovative aspect of our application is the possibility to use on a web browser some features previously available only through a mobile application.	Mobile features: App<br>App name: *Everywhere Run!*, *Everywhere Race!*<br>Operative system: Android<br><br>Web 2.0 features: Facebook app	PA	Adults (mostly females)	Mixed
292	Munson (2010), USA	In this paper, we report on a case study of deploying the Three Good Things positive psychology exercise as a Facebook application.	Mobile features: App<br>App name: *Three Good Things*<br>Operative system: iOS<br><br>Web 2.0 features: Facebook app	PA	Adults (mostly females)	Mixed (related to RCT)
291	Munson (2012), USA	The purpose of this study was to investigate particular strategies and their implementations to encourage physical activity and to compare the outcomes (goals, goal achievement, and activities journaled) between participants who used GoalPost and GoalLine.	Mobile features: App<br>App name: *GoalPost*, *GoalLine*<br>Operative system: iOS<br><br>Web 2.0 features: Facebook app	PA	Adults (males only)	Mixed
457	Zwinderman (2012), Netherlands	The goal of the current study was to examine whether persuasive technology, in the form of a physically active smartphone game, can help people engage more in moderate-intensity physical activity.	Mobile features: App<br>App name: *Phone Row*<br>Operative system: Android<br><br>Web 2.0 features: Facebook app	PA	University students	Mixed
1.6) Design and Evaluations (2 studies)						
Mobile technologies (2 studies, 100%)<br>***Study design: Mixed (2/2, 100%, linked to subsequent RCT)***						
169	Harries (2013), UK	This multidisciplinary paper reports on a large-scale field trial, designed and implemented by a group of social scientists, computer scientists and statisticians, of a new smartphone-based app for the promotion of walking in everyday life.	Mobile features: App (using accelerometer) and SMS<br>App name: *bActive*<br>Operative system: Android<br>Tested on: HTC Desire-S	PA	Adults (males only)	Mixed (related to RCT)
** 410<br><br>[Also cited in Section 2a]	Valentin (2013), USA	In the following paper, we compare two methods for engaging individuals in exercise based on passive versus active-encouragement. The passive method utilizes a wearable device that records exercise activities throughout the day whereas the active-encouragement approach utilizes a smartphone device to send encouraging reminders to the user during the day.	Mobile features: App<br>App name: *Fit Up*<br>Operative system: Android<br>Tested on: LG Optimus	PA	Adolescents and adults (mostly males)	Quantitative

Study ID	Author (Year), Country	Objectives of the paper	Technologies used	Relevant Outcomes	Target population	Methodology/Study design
1.7) Feasibility and Evaluations (55 studies)						
Mobile technologies (46 studies, 84%)<br>***Study design: Mixed (3/46, 7%, of which 1 linked to subsequent RCT); Quantitative (43/46, 93%, of which 32 linked to subsequent RCT)***						
2	Adams (2013), USA	To test an adaptive intervention for PA based on Operant and Behavior Economic principles and a percentile- based algorithm. The adaptive intervention was hypothesized to result in greater increases in steps per day than the static intervention.	Mobile features: SMS	Weight-related and behavioural (PA)	Overweight adults (mostly females)	Quantitative (related to RCT)<br><br>Trial name: *An Adaptive Physical Activity Intervention for Overweight Adults* (NCT01793064)
9	Allen (2013), USA	The major goal of this pilot study was to evaluate the feasibility, acceptability, and preliminary efficacy of theoretically based behavioural interventions delivered by smartphone technology.	Mobile features: App<br>App name: *Lose It!*<br>Operative system: iOS, Android<br>Tested on: iPhones; Android	Weight-related and behavioural (PA, diet)	Obese adults (mostly females)	Quantitative (related to RCT)<br><br>Trial name: *SLIM*
18	Appel (2014), USA	The objectives of this study are to determine the feasibility and acceptability of a smart phone application (app) or handwritten diary to self-monitor diet, sugar-drink intake, and physical activity in a mainly minority adolescent population and to estimate the pilot effectiveness of self-monitoring in helping adolescents improve their nutritional intake and physical activity levels.	Mobile features: App<br>App name: *Lose It!*<br>Operative system: iOS, Android<br>Tested on: iPhones; Android	Weight-related and behavioural (PA, diet)	Adolescents and adults (mostly females)	Quantitative<br><br>Trial name: *Starting the Conversation*
26	Atienza (2008), USA	The purpose of this pilot study was to evaluate the efficacy of a hand-held computer (i.e., personal digital assistant [PDA]) for increasing vegetable and whole-grain intake over 8 weeks in mid-life and older adults, using a randomized study design.	Mobile features: PDA App<br>App name: not provided<br>Operative system: Windows Mobile<br>Tested on: Dell Axim X5	PA and diet	Older adults (mostly females)	Quantitative (related to RCT)
36	Bauer (2010), Germany	This paper first illustrates the general potential of the short message service (SMS) for symptom and behavior monitoring and the provision of tailored feedback. Second, an SMS-based maintenance treatment (SMSMT) is introduced aimed at enhancing the treatment of childhood overweight.	Mobile features: SMS	Weight-related	Overweight or obese children and adolescents (mostly females)	Quantitative

Study ID	Author (Year), Country	Objectives of the paper	Technologies used	Relevant Outcomes	Target population	Methodology/Study design
39	Bech-Larsen (2013), Denmark	The paper presents the design and results of a goal setting and feedback intervention aimed at increasing consumption of fruits and vegetables. The purpose of the current study was to assess an application of a short message services (SMS) diary and feedback procedure to enhance a nutrition programme for Danish school children.	Mobile features: SMS	Diet	Adolescents	Quantitative (related to RCT)
50	Bond (2014), USA	We tested a smartphone-based intervention to monitor and decrease sedentary time in overweight/obese individuals, and compared 3 approaches to prompting physical activity (PA) breaks and delivering feedback on SED.	Mobile features: App (using accelerometer)<br>App name: *B-Mobile*<br>Operative system: Android<br>Tested on: Samsung Exhibit 4G SGH-T759	PA	Overweight or obese adults (mostly females)	Quantitative<br><br>Trial name: *Reducing Sedentary Time in Obese Adults* (NCT01688804)
56	Brown (2014), USA	To evaluate the acceptance and effectiveness of repetitive nutrition-related text messages on college students' nutrition knowledge and fruit and vegetable consumption.	Mobile features: SMS	Diet	College students	Quantitative (related to RCT)<br><br>Trial name: *Mobile My Plate*
60	Burke (2011), USA	Our study aimed to investigate whether using a personal digital assistant (PDA) with dietary and exercise software, with and without a feedback message, compared to using a paper diary/record (PR), results in greater weight loss and improved self-monitoring adherence.	Mobile features: PDA App<br>App name: *DietMatePro, CalculFit*<br>Operative system: Palm<br>Tested on: PalmOne Tungsten E2	Weight-related and behavioural (diet)	Obese adults (mostly females)	Quantitative (related to RCT)<br><br>Trial name: *SMART* (NCT00277771)
62	Burke (2012), USA	To determine if self-monitoring diet using a PDA only or the PDA with daily tailored feedback (PDA+ feedback), was superior to using a paper diary on weight loss and maintenance.	Mobile features: PDA App<br>App name: *DietMatePro, CalculFit*<br>Operative system: Palm<br>Tested on: PalmOne Tungsten E2	Weight-related	Obese adults (mostly females)	Quantitative (related to RCT)<br><br>Trial name: *SMART* (NCT00277771)
66	Carter (2013), UK	The aim of this pilot study was to collect acceptability and feasibility outcomes of a self-monitoring weight management intervention delivered by a smartphone app, compared to a website and paper diary.	Mobile features: App (using camera)<br>App name: *My Meal Mate*<br>Operative system: Android	Weight-related and behavioural (diet)	Overweight or obese adults (mostly females)	Quantitative (related to RCT)<br><br>Trial name: *My Meal Mate* (NCT01744535)
80	Cheung (2008), China	The purpose of the present study was to examine the effectiveness of a six-week intervention that aimed to promote teachers' physical activity level during working hours.	Mobile features: SMS	Weight-related and behavioural (PA)	School teachers	Quantitative (related to RCT)

Study ID	Author (Year), Country	Objectives of the paper	Technologies used	Relevant Outcomes	Target population	Methodology/Study design
101	David (2012), USA	We conducted a feasibility study of a 12-week walking intervention administered through an Interactive Voice Response (IVR) system and mobile phones.	Mobile features: App (using microphone) and SMS<br>App name: not provided<br>Operative system: Not specified	Weight-related and behavioural (PA)	Overweight or obese older adults (females only)	Quantitative (related to RCT)
103	de Niet, (2012), Netherlands	The effect of an SMS approach aimed at improving treatment results and reducing dropout rates in a paediatric lifestyle intervention is explored.	Mobile features: SMS	Weight-related and behavioural (diet)	Overweight or obese children and adolescents (mostly females)	Quantitative (related to RCT)<br><br>Trial name: *NTT-CO* (ISRCTN33476574)
115	Donaldson (2014), UK	The present study aimed to contribute to the knowledge gap of whether a personalised text messaging initiative could be effective and acceptable to patients in promoting positive weight outcomes (weight maintenance or further weight loss) and improved attendance at LEAP (Lifestyle, Eating and Activity Programme) WMG follow- up appointments.	Mobile features: App and SMS<br>App name: *Lifestyle, Eating and Activity Programme (LEAP)*<br>Operative system: BlackBerry<br>Tested on: BlackBerry 8700v Enterprise	Weight-related and behavioural (PA, diet)	Overweight or obese older adults (mostly females)	Quantitative<br><br>Trial name: *LEAP*
125	Faghanipour (2013), Iran	The aim of the present study was to assess the effectiveness of text messaging as an intervention to help Iranian overweight and obese women manage their weight.	Mobile features: SMS	Weight-related and behavioural (diet)	Overweight or obese university staff (females only)	Quantitative<br><br>Trial no.: IRCT201204029360N1
130	Fjeldsoe (2010), Australia	To evaluate the efficacy and feasibility of a theory-based physical activity (PA) intervention delivered to postnatal women primarily via mobile telephone short message service (SMS).	Mobile features: SMS	PA	Post-partum women	Quantitative (related to RCT)<br><br>Trial name: *MobileMums* (ACTRN12611000481976)

Study ID	Author (Year), Country	Objectives of the paper	Technologies used	Relevant Outcomes	Target population	Methodology/Study design
138	Freyne (2012), Australia	We embarked on a live clinical trial of a behavior based mobile application designed to assist users on meal replacement diet programs to judge its impact and value. We conducted a user study aimed at evaluating the effectiveness of the WMM prototype for the meal replacement program participants and its impact on user engagement with the diet and weight loss. The overall aim of the study is to investigate the effectiveness of an automated mobile phone based diet and lifestyle, prototype support tool, Weight Management Mentor, to increase consumer retention and weight loss on meal replacement programs (MRP).	Mobile features: App<br>App name: *Weight Management Mentor*<br>Operative system: iOS	Weight-related	Overweight or obese adults (females only)	Quantitative (related to RCT)<br><br>Trial name: *Celebrity Slim* (ACTRN12611000693921)
146	Fukuoka (2010), USA	This uncontrolled pilot study assessed changes in pedometer-measured step counts and self-reported physical activity during a 3-week mobile phone-based intervention. We also explored whether age, BMI, and psychosocial factors were associated with changes in step counts.	Mobile features: App and SMS<br>App name: not provided<br>Operative system: Java<br>Tested on: Motorola RAZR V3xx	Weight-related and behavioural (PA)	Sedentary adults (females only)	Quantitative
152	Glanz (2006), USA	We developed and tested a real-time diet-monitoring and feedback system using hand-held computers. The goals were to increase diet self-monitoring, reduce the burden of monitoring food intake, and increase adherence to dietary goals within a clinical trial.	Mobile features: PDA App<br>App name: *Puma Satellite Forms, Pendragon Forms*<br>Operative system: Palm<br>Tested on: Palm Pilot Vx	Diet	Overweight or obese older adults (females only)	Mixed
165	Haapala (2009), Finland	To investigate the short- and long-term effectiveness and the predictors of weight loss in a mobile phone weight-loss programme among healthy overweight adults.	Mobile features: SMS	Weight-related	Healthy and overweight adults (mostly females)	Quantitative (related to RCT)<br><br>Trial name: *Weight Balance*
186	Huang (2014), USA	In a randomized clinical trial, we evaluated whether a cancer survivor-tailored WMI would improve weight management to a greater extent as compared to a generic WMI among children who had survived acute lymphoblastic leukaemia.	Mobile features: SMS	Weight-related and behavioural (PA, diet)	Child and adolescent overweight or obese survivors of Acute lymphoblastic leukaemia (mostly females)	Quantitative (related to RCT)<br><br>Trial name: *PACE CALL/Fit4Life* (NCT01253720)

Study ID	Author (Year), Country	Objectives of the paper	Technologies used	Relevant Outcomes	Target population	Methodology/Study design
196	Johnston (2013), USA	The primary purpose of this study was first to examine the efficacy of the ì program that incorporates multiple access points compared with the self-directed approach that most Americans employ. The secondary purpose of this study was to examine the role of the modes of accessing the program within the treatment group.	Mobile features: App<br>App name: *Weight Watchers*<br>Operative system: Not specified	Weight-related	Overweight or obese adults (mostly females)	Quantitative (related to RCT)
198	Joo (2007), South Korea	We have conducted a community-based anti-obesity programme, using the mobile phone short message service (SMS) for behaviour modification.	Mobile features: SMS	Weight-related and behavioural (PA, diet)	Overweight or obese adults (mostly females)	Quantitative
217	Kim (2013), USA	To examine if a 6-week program of motivational text messaging increases physical activity (step count) among older African Americans in an urban setting.	Mobile features: SMS	PA	Overweight or obese older adults (mostly females)	Quantitative (related to RCT)<br><br>Trial name: *Text-Messaging to Motivate Walking in Older African Americans* (NCT01697475)
219	King (2008), USA	The purpose of this first-generation study was to evaluate the efficacy of a hand-held computer (i.e., personal digital assistant [PDA]) for increasing moderate intensity or more vigorous (MOD+) physical activity levels over 8 weeks in mid-life and older adults relative to a standard information control arm.	Mobile features: PDA App<br>App name: not provided<br>Operative system: Windows Mobile<br>Tested on: Dell Axim X5	PA	Older adults (mostly males)	Quantitative (related to RCT)<br><br>Trial name: CHART-PA
225	Knight (2014), Canada	The purpose of this next phase was 2-fold: (1) determine if improvements made through the 12-week intervention phase were maintained long-term by measuring a clinical marker of cardio metabolic health risk (functional aerobic capacity) at 6 months post-intervention; and (2) dis- cuss with participants their experience in a behavioural health intervention to elicit themes describing the participant experience in a program aimed to modify lifestyle using activity prescription and mHealth.	Mobile features: App<br>App name: not provided<br>Operative system: Not specified	PA	Older adults (mostly females)	Quantitative (related to RCT)

Study ID	Author (Year), Country	Objectives of the paper	Technologies used	Relevant Outcomes	Target population	Methodology/Study design
238	Lee (2011), South Korea	The aim of this study was to determine the effectiveness of a 12-week self-management intervention program, as compared to a structured exercise intervention, for obesity control among middle-aged women in Korea.	Mobile features: SMS	Weight-related and behavioural (PA, diet)	Obese adults (females only)	Quantitative
255	Lubans (2012), Australia	To evaluate the impact of a 12-month multicomponent school-based obesity prevention program, Nutrition and Enjoyable Activity for Teen Girls among adolescent girls.	Mobile features: SMS	Weight-related and behavioural (PA, diet)	Children and adolescents (females only)	Quantitative (related to RCT)<br><br>Trial name: *NEAT Girls* (ACTRN12610000330044)
297	Newton (2014), USA	The intent of the study was to determine the feasibility and efficacy of a 12-week physical activity promotion program targeting children, which was delivered to parents through mobile phones.	Mobile features: SMS	Weight-related and behavioural (PA)	Sedentary children (mostly females)	Quantitative (related to RCT)<br><br>Trial name: *P-Mobile* (NCT01551108)
301	Ni Mhurchu (2014), New Zealand	We developed an mHealth weight management programme using proven face-to-face behaviour change techniques and incorporating target population input. Our aim was to evaluate the feasibility, acceptability and potential effectiveness of this programme for ethnically diverse adults with a view to informing a larger trial.	Mobile features: SMS	Weight-related	Overweight or obese adults (mostly females)	Quantitative<br><br>Trial name: *Horizon Study* (ACTRN12612000850875)
304	Nollen (2014), USA	To test a 12-week mobile technology intervention for use and estimate effect sizes for a fully powered trial.	Mobile features: PDA App<br>App name: not provided<br>Operative system: Windows Mobile<br>Tested on: Asus MyPal A626	Weight-related and behavioural (diet)	Overweight or obese children and adolescents (females only)	Quantitative (related to RCT)
316	Patrick (2009), USA	This paper describes the development and evaluation of a text message–based intervention designed to help individuals lose or maintain weight over 4 months.	Mobile features: SMS	Weight-related	Overweight or obese adults (mostly females)	Quantitative (related to RCT)<br><br>Trial name: *mDIET* (NCT00415870)

Study ID	Author (Year), Country	Objectives of the paper	Technologies used	Relevant Outcomes	Target population	Methodology/Study design
332	Read (2014), Canada	This is the first study to use cell phone technology combined with self-monitored and a stage-matched physical activity intervention with metabolic syndrome patients in a rural community. The purpose of this pilot project is to test the feasibility and effectiveness of the intervention and decide whether a larger randomized trial is warranted.	Mobile features: App<br>App name: not provided<br>Operative system: BlackBerry<br>Tested on: BlackBerry Curve 8300	Weight-related and behavioural (PA)	Obese adult patients with metabolic syndrome<br>(mostly females)	Quantitative<br><br>Trial name: *DaTA*
337	Robinson (2013), UK	Here, the development and feasibility testing of an 'attentive eating' smart-phone application is reported. Our main aims were to test whether overweight and obese participants find an attentive eating smartphone application acceptable to use and use it on a regular basis.	Mobile features: App (using camera)<br>App name: not provided<br>Operative system: iOS<br>Tested on: iPhone	Weight-related	Overweight or obese adults (mostly females)	Mixed
351	Schwerdtfeger (2012), Germany	We examined whether a text message-based intervention, targeted at increasing daily levels of physical activity, would be more effective than a standard psycho-educational intervention and a control condition.	Mobile features: SMS	Weight-related and behavioural (PA)	University students and staff<br>(mostly females)	Quantitative (related to RCT)
353	Shapiro (2008), USA	To examine acceptability, attrition, adherence, and preliminary efficacy of mobile phone short message service (SMS; text messaging) for monitoring healthful behaviours in children.	Mobile features: SMS	PA and diet	Children and adults (mostly females)	Quantitative (related to RCT)
354	Shapiro (2012), USA	This randomized controlled trial evaluated a daily text-messaging weight loss intervention.	Mobile features: SMS	Weight-related and behavioural (PA, diet)	Overweight or obese adults (mostly females)	Quantitative (related to RCT)<br><br>Trial name: *Text4Diet*
358	Shaw (2013), USA	We conducted an exploratory randomized controlled trial using mixed methods, with data collected at baseline, and 1 month and 3 months post baseline. A primary focus of this exploratory study was to estimate effect sizes for text message-based interventions.	Mobile features: SMS	Weight-related	Overweight or obese adults (mostly females)	Mixed (related to RCT)
366	Silveira (2013), Italy	The objectives were to (1) investigate which IT-mediated motivation strategies increase adherence to physical exercise training plans in older people, (2) assess the impact of app on physical activity behavior change, and (3) demonstrate the effectiveness of the app training to improve gait speed.	Mobile features: App<br>App name: *Active Lifestyle*<br>Operative system: iOS<br>Tested on: iPad	PA	Healthy older adults (mostly females)	Quantitative (related to RCT)

Study ID	Author (Year), Country	Objectives of the paper	Technologies used	Relevant Outcomes	Target population	Methodology/Study design
369	Sirriyeh (2010), UK	The present study attempts to develop and pilot the feasibility and efficacy of a novel intervention using affective messages as a strategy to increase physical activity (PA) levels in adolescents.	Mobile features: SMS	PA	Adolescents and adults (mostly females)	Quantitative (related to RCT)
373	Smith (2014), Australia	The goal of this study was to evaluate the impact of the Active Teen Leaders Avoiding Screen-time (ATLAS) intervention for adolescent boys, an obesity prevention intervention using smartphone technology.	Mobile features: App<br>App name: *Atlas*<br>Operative system: Android, iOS	Weight-related and behavioural (PA, diet)	Children and adolescents (males only)	Quantitative (related to RCT)<br><br>Trial name: ATLAS (ACTRN12612000978864)
379	Spring (2013), USA	We conducted a 2-arm, 12-month study (October 1, 2007, through September 31, 2010). Seventy adults (body mass index < 25 and < 40 [calculated as weight in kilograms divided by height in meters squared]) were randomly assigned either to standard-of-care group treatment alone (standard group) or to the standard and connective mobile technology system (+mobile group)	Mobile features: PDA App<br>App name: not provided<br>Operative system: Not specified	Weight-related and behavioural (PA, diet)	Obese adult patients with chronic pain (mostly males)	Quantitative (related to RCT)<br><br>Trial name: MOVE2!; PDA+ (NCT00371462)
382	Steinberg (2013), USA	This pilot study evaluated the feasibility of a text messaging intervention for weight loss among predominantly black women.	Mobile features: SMS	Weight-related and behavioural (PA)	Overweight or obese adults of ethnic minority<br>(females only)	Quantitative (related to RCT)<br><br>Trial name: *Shape Plan* (NCT00939081)
396	Thomas (2013), USA	The objective of this study was to evaluate smartphones as a method of delivering key components of established and empirically validated behavioural weight loss treatment, with an emphasis on adherence to self-monitoring.	Mobile features: App<br>App name: *Health-E-Call, DailyBurn*<br>Operative system: iOS<br>Tested on: iPhone 3GS	Weight-related	Overweight or obese older adults (mostly females)	Quantitative
397	Thorsteinsen (2014), Norway	The main objective of this pilot study was to test the effectiveness of an online, interactive physical activity intervention that also incorporated gaming components.	Mobile features: SMS	PA	Adults (mostly females)	Quantitative (related to RCT)<br><br>Trial name: *Lifestyle Tool*

Study ID	Author (Year), Country	Objectives of the paper	Technologies used	Relevant Outcomes	Target population	Methodology/Study design
Web 2.0 technologies (5 studies, 11%)<br>Study design: Mixed (1/5, 20%, linked to subsequent RCT); Quantitative (4/5, 80%, all linked to subsequent RCT)						
55	Brindal (2012), Australia	To determine whether supportive features and personalization in a 12-week web-based lifestyle intervention with no in-person professional contact affect retention and weight loss.	Web 2.0 features: Custom social networking site	Weight-related and behavioural (PA, diet)	Overweight or obese adults (mostly females)	Quantitative (related to RCT)
74	Cavallo (2012), USA	The purpose of the present study was to test the efficacy and feasibility of a 12-week physical activity social support intervention partly administered through Facebook.	Web 2.0 features: Facebook group	PA	University students (females only)	Quantitative (related to RCT)<br><br>Trial name: *INSHAPE* (NCT01421758)
411	Valle (2013), USA	This study evaluated the feasibility and preliminary efficacy of a 12-week, Facebook-based intervention aimed at increasing moderate-to-vigorous intensity PA compared with a Facebook-based self-help comparison condition.	Web 2.0 features: Facebook group	Weight-related and behavioural (PA)	Adult cancer survivors (mostly females)	Quantitative (related to RCT)<br><br>Trial name: *FITNET* (NCT01349153)
434	Womble (2004), USA	To assess, in a 1-year randomized controlled trial, the efficacy of a commercial Internet weight loss program in improving weight, cardiovascular health, and quality of life.	Web 2.0 features: eDiets, commercial social networking site	Weight-related	Overweight or obese adults (females only)	Quantitative (related to RCT)
444	Yen (2013), USA	This study was conducted to assess the impact of a nutrition education intervention on food groups and omega-3 (n-3) fatty acid consumption in middle-aged women.	Web 2.0 features: Blog	Diet	Middle-aged women	Mixed (related to RCT)
Mobile and Web 2.0 technologies (4 studies, 7%)<br>Study design: Quantitative (4/4, 100%, all linked to subsequent RCT)						
118	Duncan (2014), Australia	This study examines the effectiveness of a 9-month IT-based intervention (ManUp) to improve the physical activity, dietary behaviours, and health literacy in middle-aged males compared to a print-based intervention.	Mobile features: App and SMS<br>App name: *ManUp*<br>Operative system: iOS<br><br>Web 2.0 features: "Social media group challenges"	Weight-related and behavioural (PA, diet)	Adults (males only)	Quantitative (related to RCT)<br><br>Trial name: *ManUp* (ACTRN12611000081910)
176	Hebden (2014), Australia	This pilot study aimed to measure the effect of a 12-week mobile health (mHealth) intervention on body weight, body mass index and specific lifestyle behaviours addressed by the programme.	Mobile features: App and SMS<br>App name: *ePass*, *eVIP*, *eTIYP*, *eSIYP*<br>Operative system: Python<br><br>Web 2.0 features: Blogs, discussion forums	Weight-related and behavioural (PA)	Overweight or obese adults (mostly females)	Quantitative (related to RCT)

Study ID	Author (Year), Country	Objectives of the paper	Technologies used	Relevant Outcomes	Target population	Methodology/Study design
182	Herring (2014), USA	To examine the feasibility, acceptability, and initial efficacy of a technology-based weight loss intervention for urban, low-income mothers.	Mobile features: SMS<br><br>Web 2.0 features: Facebook group	Weight-related and behavioural (PA, diet)	Overweight or obese adults (females only)	Quantitative (related to RCT)<br><br>Trial name: *Healthy4Baby*
295	Napolitano (2013), USA	This study examined the feasibility, acceptability, and initial efficacy of a technology-based 8-week weight loss intervention among college students.	Mobile features: SMS<br><br>Web 2.0 features: Facebook group	Weight-related and behavioural (PA)	Overweight or obese college students (mostly females)	Quantitative (related to RCT)
1.8) Design, Feasibility and Evaluations (22 studies)						
Mobile technologies (16 studies, 72%)<br>***Study design: Mixed (11/16, 69%, of which 3 linked to subsequent RCT); Quantitative (5/16, 31%, of which 1 linked to subsequent RCT)***						
54	Brindal (2013), Australia	The objective of the present study was to develop and evaluate a weight-loss intervention delivered by a smartphone app that supported individuals embarking on a diet and that was evidenced-based.	Mobile features: App<br>App name: *Weight Management Mentor*<br>Operative system: iOS<br>Tested on: iPhone	Weight-related	Overweight or obese adults (females only)	Quantitative (related to RCT)<br><br>Trial name: *Celebrity Slim* (ACTRN12611000693921)
91	Consolvo (2006), USA	We describe Houston, a prototype mobile phone application for encouraging activity by sharing step count with friends. We also present four design requirements for technologies that encourage physical activity that we derived from a three-week long in situ pilot study	Mobile features: App<br>App name: *Houston*<br>Operative system: Python<br>Tested on: Nokia 6600	PA	University students and staff<br>(females only)	Mixed
92	Consolvo (2008), USA	Our current investigation examines the long-term effectiveness of using a stylized representation of behavior on a personal, mobile display to encourage regular and varied physical activity. We conducted a three-month field experiment of our system.	Mobile features: App<br>App name: *UbiFit Garden*<br>Operative system: Not specified	PA	Adults (mostly females)	Mixed (related to RCT)
220	King (2013), USA	This first-generation feasibility study aimed to apply a behavioural science-informed user experience design (BSUED) process in developing smartphone applications to increase regular physical activity and decrease sedentary behavior (e.g., prolonged sitting) in adults who to date have received less attention in this field (i.e., midlife and older adults new to smartphone technology).	Mobile features: App<br>App name: not provided<br>Operative system: Android<br>Tested on: Android phone	PA	Sedentary adults (mostly females)	Mixed (related to RCT)

Study ID	Author (Year), Country	Objectives of the paper	Technologies used	Relevant Outcomes	Target population	Methodology/Study design
221	Kirwan (2012), Australia	To measure the potential of a newly developed smartphone application to improve health behaviours in existing members of a website-delivered physical activity program (10,000 Steps, Australia). The aims of the study were to (1) examine the effect of the smartphone application on self-monitoring and self-reported physical activity levels, (2) measure the perceived usefulness and usability of the application, and (3) examine the relationship between the perceived usefulness and usability of the application and its actual use.	Mobile features: App (using accelerometer)<br>App name: *iStepLog*<br>Operative system: iOS	PA	Adults	Quantitative
240	Lee (2010), South Korea	We developed a mobile phone-based weight control system that tracks an obese patient's daily nutrition intake, and also has games that users can play to learn about weight control. The present study evaluated the effectiveness of the mobile phone application with respect to acquiring dietary information, weight control and user satisfaction.	Mobile features: App<br>App name: *SmartDiet*<br>Operative system: Java	Weight-related	Obese adults	Quantitative
287	Morak (2008), Austria	The aim of the present pilot study was to assess the technical feasibility, usability and acceptance of a web-based therapy management system with mobile phone access to support obese patients.	Mobile features: SMS<br>Operative system: Symbian<br>Tested on: Nokia 6020	Weight-related and behavioural (PA)	Obese patients (mostly females)	Quantitative
293	Mutsuddi (2012), USA	In this paper we report the findings from a study conducted for a longer term (3 months) with a larger sample size (n=28) to discover if daily mobile phone text messaging is indeed an effective delivery channel for encouraging physical activity, even once the novelty effect of the technology wears off. The goal of our text messaging system was to encourage opportunistic physical activity, which is, incorporating activities into everyday life to increase the number of steps taken (e.g. walking to work instead of driving, taking the stairs instead of the elevator, walking to take a break from studies). In this paper, we focus on the overall impact of text messaging.	Mobile features: SMS<br>Operative system: Perl, PHP	PA	University students (mostly females)	Mixed

Study ID	Author (Year), Country	Objectives of the paper	Technologies used	Relevant Outcomes	Target population	Methodology/Study design
303	Nollen (2013), USA	This study developed a personal digital assistant (PDA) program to promote increased intake of fruits and vegetables (FV) in predominately low-income, ethnic minority girls. This study used a three-phase community- engaged process, including (i) engagement of a Student Advisory Board (SAB) to determine comfort with PDAs; (ii) early testing of Prototype I and rapid re-design by the SAB and (iii) feasibility testing of Prototype II in a new sample of girls.	Mobile features: PDA App<br>App name: *Growing Up Strong*<br>Operative system: Windows Mobile<br>Tested on: Asus MyPal A626	Diet	Low-income children and adolescents (females only)	Mixed
360	Shaw (2013), USA	To develop theoretically driven weight loss sustaining text messages and pilot an mHealth SMS text messaging intervention to promote sustaining recent weight loss in order to understand optimal frequency and timing of message delivery, and for feasibility and usability testing.	Mobile features: SMS	Weight-related and behavioural (PA, diet)	Overweight or obese adults (mostly females)	Mixed
377	Spillers (2014), USA	In this paper we discuss to what extent gamification and social elements improve user motivation and lead to short-term positive behavior change. We examine the efficacy of social features in three different m-health running applications with varying levels of social and gamification functionality, each supporting the core task of tracking a user's running activity.	Mobile features: App<br>App name: *Digifit, Endomondo, Runno*<br>Operative system: iOS<br>Tested on: iPhone	PA	Adult runners (mostly males)	Mixed
400	Toscos (2006), USA	We are developing a preventative health cell phone application that helps motivate teenage girls to exercise by exploiting their social desire to stay connected with their peers. Our study investigated how collecting, sharing and comparing personal fitness information impacts activity level and health awareness.	Mobile features: PDA App and SMS<br>App name: *Chick Clique*<br>Operative system: Not specified	PA	Adolescents (females only)	Quantitative
399	Toscos (2008), USA	We present the findings from a three-week field study of a mobile phone application designed to create a support group for physical activity within an existing social network of adolescent girls. Our findings suggest design considerations for technologies that encourage physical activity for this user group.	Mobile features: App<br>App name: not provided<br>Operative system: Symbian<br>Tested on: Nokia N73	PA	Adolescents (females only)	Mixed

Study ID	Author (Year), Country	Objectives of the paper	Technologies used	Relevant Outcomes	Target population	Methodology/Study design
412	van Dantzig (2013), Netherlands	In this paper, we present the results of our quest to create effective persuasive mobile applications aimed at reducing sedentary behavior. First, we developed SitCoach, a mobile application to nudge office workers from their seats. SitCoach monitors physical activity and sedentary behavior and provides timely persuasive messages suggesting active breaks.	Mobile features: App and SMS<br>App name: *SitCoach*<br>Operative system: iOS	PA	Sedentary adults	Mixed (related to RCT)
419	Verwey (2014), Netherlands	We tested the performance, acceptance and user satisfaction of a tool to stimulate physical activity. The tool consisted of an accelerometer, a smartphone app and a server/web application.	Mobile features: App (using accelerometer)<br>App name: *It's LiFe!*<br>Operative system: Android<br>Tested on: Samsung Galaxy Ace	PA	Overweight or obese patients with COPD or T2D<br>(mostly males)	Mixed
456	Zuckerman (2014), Israel	We set out to evaluate the effectiveness of virtual rewards and social comparison-two game elements prevalent in persuasive systems. We developed a research prototype, called "StepByStep," aimed to promote routine walking. We created different versions of StepByStep, implemented as an application on Android-based mobile devices, and compared their effectiveness in two field studies.	Mobile features: App (using accelerometer)<br>App name: *StepByStep*<br>Operative system: Android<br>Tested on: Samsung Galaxy S	PA	University students and staff<br>(mostly females)	Mixed
Web 2.0 technologies (3 studies, 14%)<br>***Study design: Mixed (1/3, 33%, linked to subsequent RCT); Quantitative (2/3, 67%, all linked to subsequent RCT)***						
134	Foster (2010), UK	This paper presents the design and in-the-wild evaluation of StepMatron, a Facebook application designed to provide social and competitive context for daily pedometer readings in order to motivate physical activity in the working environment.	Web 2.0 features: Facebook app	PA	Nurses<br>(mostly females)	Quantitative (related to RCT)
200	Kamal (2013), Canada	We investigate how online social networks (OSN) can change health behaviour by blending theories from health behaviour and participation in OSNs.	Web 2.0 features: Custom social networking site	Diet	Adults (mostly females)	Mixed (related to RCT)

Study ID	Author (Year), Country	Objectives of the paper	Technologies used	Relevant Outcomes	Target population	Methodology/Study design
204	Kernot (2014), Australia	This mixed method study aimed to pilot and assess the usability of the Mums Step It Up Facebook app, a new team-based physical activity intervention for mothers with young children.	Web 2.0 features: Facebook app integration	Weight-related and behavioural (PA)	Post-partum women	Quantitative (related to RCT)<br><br>Trial name: *Mums Step it Up* (ACTRN12613000069752)
Mobile and Web 2.0 technologies (3 studies, 13%)<br>***Study design: Mixed (3/3, 100%, of which 1 linked to subsequent RCT)***						
128	Fjeldsoe (2012), Australia	To describe the iterative development process and final version of 'MobileMums': a physical activity intervention for women with young children (<5 years) delivered primarily via mobile telephone (mHealth) short messaging service (SMS). This paper described the process of developing the MobileMums physical activity intervention and the final version currently undergoing efficacy testing.	Mobile features: SMS<br><br>Web 2.0 features: Facebook group	PA	Adults (females only)	Mixed (related to RCT)<br><br>Trial name: *MobileMums* (ACTRN12611000481976)
210	Khalil (2013), UAE	We developed a mobile application called STEP UP that monitors physical activity and provides data that can be easily shared within a social network. We then conducted an exploratory, theoretical study based on the theory of reasoned action (TRA) followed by an experimental trial and user study. The purpose of the studies was to explore the effect of persuasive technology on physical activity behavior and to investigate its effectiveness in motivating users to use the technology to be more physically active.	Mobile features: App<br>App name: *Step Up*<br>Operative system: Java<br><br>Web 2.0 features: Custom social networking site	PA	University students and staff<br>(females only)	Mixed<br><br>Trial name: *Step UP*
449	Young (2010), USA	The purpose of this study is to provide real-time information sharing in order to modify the behaviours of girls and ultimately lead to improved health habits. Our study investigated how collecting, sharing and comparing personal fitness information impacts activity level and health awareness.	Mobile features: App and SMS<br>App name: not provided<br>Operative system: Not specified<br><br>Web 2.0 features: Twitter-like	PA	Adolescents (females only)	Mixed

1b) Technologies for behavior change (reviews)

Study ID	Author (Year), Country	Objectives	Technology/Intervention ("focus", relevant keywords)	Relevant Outcomes	Target population	Methodology/Study design
1.1) Design and development (10 studies)						
Mobile technologies (7 studies, 70%)						
42	Bert (2014), Italy	This paper aims to explore the availability of smartphone applications in the field of health promotion and to point out advantages and disadvantages of their use.	Mobile technologies ("smartphone apps")	Various health indicators, including those for weight-related outcomes (BMI, weight) PA, diet	General pop.	Systematic "scoping" review with qualitative synthesis
68	Carter (2014), UK	This report aims to discuss what is known about the current evidence regarding these handheld electronic devices aimed at facilitating dietary behaviour change for weight loss in overweight and obese adults and the key issues for consideration in this emerging field.	Mobile technologies ("Handheld Electronic Technology", incl. micro-computers, PDAs, text pagers, text-messaging, smartphone apps)	Weight loss, diet	Overweight/obese adults	Non-systematic review with qualitative synthesis
72	Castelnuovo (2014), Italy	mHealth applications which can be implemented in weight loss protocols and obesity rehabilitation are discussed, taking into account future research directions in this promising area.	Mobile technologies ("mHealth apps") and the *TECNOB* project	Weight management	Obese	Non-systematic review with qualitative synthesis of research project (Case report)
127	Fiordelli (2013), Switzerland	To provide a comprehensive view of the field of mHealth research to date and to understand whether and how the new generation of smartphones has triggered research, since their introduction 5 years ago.	Mobile technologies ("mHealth", incl. mobile health, m-health, mobile phone, cell phone, smartphone, iPhone, Blackberry, Android)	Various health indicators, including those for weight-related outcomes (BMI, weight) PA, diet	General pop.	Systematic "scoping" review with qualitative synthesis

Study ID	Author (Year), Country	Objectives	Technology/Intervention ("focus", relevant keywords)	Relevant Outcomes	Target population	Methodology/Study design
223	Klasnja (2012), USA	Our first goal in this paper is to map out the current state of this rapidly growing body of work. Rather than focus on a particular condition or health objective (e.g., encouraging weight loss), we present a taxonomy of the strategies and types of interventions that have been implemented with mobile phones. Because our goal is to provide a review of the mobile phone design space, rather than a systematic review of efficacy evaluations, we present only illustrative examples of systems that use each type of intervention that we identified.	Mobile technologies (incl. mobile phones, cell phones, SMS, and text message)	Various health indicators, including those for weight-related outcomes (BMI, weight) PA, diet	General pop.	Systematic "scoping" review with qualitative synthesis
335	Riley (2011), USA	The study aims to determine how health behavior theories are applied to mobile interventions. This is a review of the theoretical basis and interactivity of mobile health behavior interventions. In contrast to meta-analytic reviews of effect size, the purpose of this review is to assess how theory has been employed in the development of mobile health behavior interventions and how the interactive and adaptive potential of these interventions may require new dynamic systems theories of health behavior.	Mobile technologies (defined as: "computer devices that are intended to be always on and carried on the person throughout the day")	Various health indicators, including those for weight-related outcomes (BMI, weight) PA, diet	General pop.	Systematic "scoping" review with qualitative synthesis

Study ID	Author (Year), Country	Objectives	Technology/Intervention ("focus", relevant keywords)	Relevant Outcomes	Target population	Methodology/Study design
357	Shaw (2014), USA	Treatment fidelity is critical because the valid interpretation and translation of intervention studies depend on treatment fidelity assessments. We describe strategies used to assess treatment fidelity in mobile health (mHealth) interventions aimed at sustaining healthy behaviours in weight loss. We reviewed treatment fidelity recommendations for mHealth-based behavioural interventions and described how these recommendations were applied in three recent weight loss studies. We illustrate how treatment fidelity can be supported during study design, training of providers, treatment delivery, receipt of treatment, and enactment of treatment skills.	Mobile technologies ("mHealth interventions") as employed in the following interventions: *Sustaining Weight Loss through Text Messaging: mSustain*; *Weighing Everyday to Improve and Gain Health: WEIGH; Shape Plan*	Treatment fidelity; Healthy behaviours in initiating and maintaining weight loss	Obese adult patients	Non-systematic review with qualitative synthesis of research project (Case report)
Web 2.0 technologies (2 studies, 20%)						
* 3 [Also cited in Section 3]	Adams (2007), Netherlands	In this article, I will discuss the emergent findings from a new project that looks at blogging interfaces as potential tools for disease prevention and health promotion. The purpose of this paper has been to take a first look at health-related blog development in the Netherlands and to raise questions about new trends in health information management that are emerging with the further development of point-and-click tools.	Blogs (*Valtaf.nl*)	PA and diet	General pop.	Non-systematic review with qualitative synthesis of research project (Case report)
168	Hamm (2013), Canada	To map the state of the existing literature evaluating the use of social media in patient and caregiver populations.	Web 2.0 technologies (defined as Kaplan and Haenlein's classification scheme: "collaborative projects, blogs or micro blogs, content communities, social networking sites and virtual worlds")	Various health indicators, including those for weight-related outcomes (BMI, weight) PA, diet	Patients and caregivers	Systematic "scoping" review with qualitative synthesis
Various technologies, including mobile, Web 2.0, Internet, computers, etc. (1 study, 10%)						

Study ID	Author (Year), Country	Objectives	Technology/Intervention ("focus", relevant keywords)	Relevant Outcomes	Target population	Methodology/Study design
395	Thomas (2014), USA	The purpose of this review is to discuss studies in which digital technology has been used for behavioural weight control, report on advances in consumer technology that are widely adopted but insufficiently tested, and explore potential future directions for both.	Various technologies ("Digital technology, incl. web, mobile phone, smartphone tools, apps, text-messaging, virtual reality, gaming, consumer activity monitors e.g. *FitBit*, *Nike+ Fuelband*, etc.)	Weight control	General pop. and diabetic patients	Non-systematic review with qualitative synthesis
1.2) Evaluations (39 studies)						
Mobile technologies (20 studies, 51%)						
5	Aguilar-Martinez (2014), Spain	We conducted a systematic review of the literature on the use of mobile phones for weight loss. The objective of the present study was to review the use of mobile phones and associated apps as weight-loss tools.	Mobile technologies (incl. cellular phones, smartphones)	Weight loss	Overweight or obese adults	Systematic review with qualitative synthesis
31	Bacigalupo (2013), UK	The aim of this systematic review was to study in more detail whether mobile technology is an appropriate medium for facilitating weight loss in over- weight and obese adults, but also to identify, compare and contrast the features of the studies and interventions to inform future RCT study design.	Mobile technologies (incl. mobile phones, PDAs, hand-held computers, pocket computers, Palm Pilots)	Weight-loss (incl. weight changes, BMI, waist circumference)	Overweight or obese adults	Systematic review with qualitative synthesis
* 51 [Also cited in Section 2b]	Bort-Roig (2014), Australia	This study systematically reviewed evidence on smartphones and their viability for measuring and influencing physical activity.	Mobile technologies ("smartphones", incl. mobile phones, cell-phones, smartphones; excluding text messaging)	PA	General pop.	Systematic review with qualitative synthesis

Study ID	Author (Year), Country	Objectives	Technology/Intervention ("focus", relevant keywords)	Relevant Outcomes	Target population	Methodology/Study design
47	Blackman (2013), USA	The purpose of this systematic review was to use the RE-AIM (reach, effectiveness, adoption, implementation, and maintenance) framework to determine the extent to which mHealth intervention research for promoting PA reports on factors that inform generalizability across settings and populations and to provide recommendations for investigators planning to conduct this type of research.	Mobile technologies ("mHealth", incl. mobile phones, cell phone, PDAs, SMS, or text messaging)	PA; RE-AIM components	General pop.	Systematic review with qualitative synthesis
57	Buchholz (2013), USA	Review the existing scientific literature on adult physical activity text messaging interventions.	Mobile technologies (text messaging)	PA	Adults	Systematic review with qualitative synthesis
58	Buhi (2013), USA	To perform a systematic review of the literature concerning behavioural mobile health (mHealth) and summarize points related to heath topic, use of theory, audience, purpose, design, intervention components, and principal results that can inform future health education applications.	Mobile technologies (incl. cell-phones, text messaging, SMS, mobile phone, multi- media messaging service, MMS, video, and mobile video)	Various health indicators, including those for weight-related outcomes (BMI, weight) PA, diet	General pop.	Systematic review with qualitative synthesis
87	Cole-Lewis (2010), USA	The primary objective of this systematic review is to assess the effectiveness of behavior change interventions for disease management and prevention delivered primarily through text messaging.	Mobile technologies (text messaging)	Weight-related outcomes (BMI, weight) PA, diet	General pop.	Systematic review with qualitative synthesis

Study ID	Author (Year), Country	Objectives	Technology/Intervention ("focus", relevant keywords)	Relevant Outcomes	Target population	Methodology/Study design
* 107<br><br>[Also cited in Section 3]	Derbyshire (2013), UK	In this paper, we set out to evaluate whether smartphone technology use is effective in terms of significantly improving aspects of women's health, including reductions in body weight and improved health awareness for chronic diseases, including diabetes, heart disease, breast cancer, and osteoporosis. The uses of health apps to improve diet quality, support mental, and maternal health are also reviewed.	Mobile technologies ("smartphone technology", incl. cellular phone, mobile phone, smartphones, apps)	Various health indicators, including those for Weight-related outcomes (BMI, weight) PA, diet	Women	Systematic review with qualitative synthesis
126	Fanning (2012), USA	To conduct a meta-analysis of research utilizing mobile devices to influence physical activity behavior. The aims of this review were to: (1) examine the efficacy of mobile devices in the physical activity setting, (2) explore and discuss implementation of device features across studies, and (3) make recommendations for future intervention development.	Mobile technologies (incl. mobile phones, cell phones, PDAs, SMS, text messaging)	PA	General pop.	Systematic review with meta-analysis
132	Fjeldsoe (2009), Australia	This review analyses the application of SMS for delivering health behavior change interventions to establish what can be learned from research conducted to date and make recommendations for future research	Mobile technologies (text messaging)	Various health indicators, including those for Weight-related outcomes (BMI, weight) PA, diet	General pop.	Systematic review with qualitative synthesis
136	Free (2013), UK	This systematic review aimed to quantify the effectiveness of mobile technology-based interventions delivered to health care consumers for health behaviour change and management of diseases.	Mobile technologies (incl. mobile phone, cell phone, PDA, SMS, or text messaging)	Various health behaviours, including diet and PA	Health care consumers	Systematic review with meta-analysis
215	Khokhar (2014), Canada	The purpose of the present study was to perform a systematic review and meta-analysis of RCTs reporting the use of mobile electronic devices in weight loss efforts among overweight and obese adult population.	Mobile technologies ("mobile electronic devices", incl. mobile phone, Internet, computers handheld, wireless technology, text- messaging, email, smartphone)	Body weight	Overweight or obese adults	Systematic review with meta-analysis

Study ID	Author (Year), Country	Objectives	Technology/Intervention ("focus", relevant keywords)	Relevant Outcomes	Target population	Methodology/Study design
259	Lyzwinski (2014), UK	The aims of this review will be to determine whether mobile devices induce weight loss and improvements in diet and physical activity levels when compared with standard controls without a weight loss intervention or controls allocated to non-mobile device weight loss interventions.	Mobile technologies (incl. mobile phone, text messaging, smartphones, PDAs)	Weight- related outcomes (weight change, BMI, waist circumference, body fat percentage), PA, diet (changes in fruit and vegetable intake)	Adults	Systematic review with meta-analysis
* 308<br><br>[Also cited in Section 2b]	O'Reilly (2013), USA	A comprehensive systematic review of how mobile technology has been used for measuring PA and promoting PA behavior change.	Mobile technologies (incl. mobile phones, SMS, Internet, web email as keywords)	PA	General pop.	Systematic review with qualitative synthesis
324	Preston (2011), USA	This article will focus on recent research looking at the effectiveness of text messaging as a health intervention eliciting a positive behavioural response among adolescents and young adults in the management of chronic disease and unhealthy behaviours.	Mobile technologies (text messaging)	Various health behaviours, including diet and PA	Adolescents and young adults, managing chronic diseases and unhealthy behaviours	Systematic review with qualitative synthesis
359	Shaw (2012), USA	The purpose of this review was to answer the following question: What is the relationship between the use of SMS as an intervention medium and weight loss?	Mobile technologies (text messaging)	Weight- related outcomes (e.g., weight change, BMI, waist circumference, body fat percentage), PA and diet	General pop.	Systematic review with qualitative synthesis
368	Siopis (2014), Australia	This review investigates the efficacy of weight management programmes incorporating text messaging.	Mobile (text messaging)	Weight- related outcomes (weight change, BMI, waist circumference, body fat percentage)	General pop.	Systematic review with meta-analysis
383	Stephens (2013), USA	This systematic review was conducted to determine user satisfaction and effectiveness of smartphone applications and text messaging interventions to promote weight reduction and physical activity.	Mobile technologies ("smartphones and text messaging")	Various health indicators, including weight-related outcomes (e.g., weight change, BMI, waist circumference, body fat percentage) and PA	General pop.	Systematic review with qualitative synthesis

Study ID	Author (Year), Country	Objectives	Technology/Intervention ("focus", relevant keywords)	Relevant Outcomes	Target population	Methodology/Study design
426	Wei (2011), USA	The present study analyses the feasibility and efficacy of text-messaging-delivered interventions in clinical outcomes and healthy behaviour modifications.	Mobile technologies (text messaging)	Various health indicators, including weight-related outcomes (e.g., weight change, BMI, waist circumference, body fat percentage)	General pop.	Systematic review with qualitative synthesis
431	Williams (2012), USA	To determine if text messaging can be used as a communication tool to promote the adherence to daily physical activity guidelines in adults, a literature review was conducted.	Mobile technologies (text messaging)	PA	Adults	Systematic review with qualitative synthesis
Web 2.0 technologies (6 studies, 15%)						
25	Ashrafian (2014), UK	The objective of this study was to review the role of interventions that used social networking services in the management of obese and overweight patients. We used a comprehensive meta-analysis of published randomized controlled studies to assess the effect of the services in achieving beneficial reductions in BMI	Web 2.0 technologies (social networking services)	Body mass index (BMI)	Obese and overweight patients	Systematic review with meta-analysis
76	Chang (2013), USA	The objective of this study was to systematically describe the use and impact of social media in online weight management interventions.	Web 2.0 technologies (defined as Kaplan and Haenlein's classification scheme: "collaborative projects, blogs or micro blogs, content communities, social networking sites and virtual worlds")	Various health indicators, including weight-related outcomes (e.g., weight change, BMI, waist circumference, body fat percentage), PA and diet	General pop.	Systematic review with qualitative synthesis
133	Foster (2013), UK	To compare the effectiveness of remote and web 2.0 interventions for PA promotion in community dwelling adults (Aged 16 years and above) with a control group exposed to placebo or no or minimal intervention.	Web 2.0 and remote technologies (defined as using Internet, smart phones or more traditional methods e.g., telephone, mail- outs)	PA	Adults	Systematic review with meta-analysis
261	Maher (2014), Australia	The intent of the study was to systematically review the current level of evidence regarding the effectiveness of online social network health behavior interventions.	Web 2.0 technologies (social networking sites)	Various health indicators, including weight-related outcomes (e.g., weight change, BMI, waist circumference, body fat percentage), PA and diet	General pop.	Systematic review with meta-analysis

Study ID	Author (Year), Country	Objectives	Technology/Intervention ("focus", relevant keywords)	Relevant Outcomes	Target population	Methodology/Study design
334	Richards (2013), UK	To compare the effectiveness of face-to-face versus remote and web 2.0 interventions for PA promotion in community dwelling adults (Aged 16 years and above).	Web 2.0 and remote technologies (compared to face-to-face)	PA	Adults	Systematic review with meta-analysis
432	Williams (2014), Canada	To conduct a systematic review of randomised controlled trials (RCTs) examining the use of social media to promote healthy diet and exercise in the general population.	Web 2.0 technologies (defined as Kaplan and Haenlein's classification scheme: "collaborative projects, blogs or micro blogs, content communities, social networking sites and virtual worlds")	PA and diet, weight loss, waist circumference	General pop.	Systematic review with meta-analysis
Various technologies, including mobile, Web 2.0, Internet, computers, etc. (13 studies, 33%)						
10	Allen (2014), USA	The literature was systematically reviewed by examining clinical trials of technology-assisted interventions for weight loss or weight maintenance among overweight and obese adults.	Various technologies (incl. telephone, iPhone, iPad, smartphone, smart phone, electronic mail, email, e mail, e-mail, Internet, world wide web social media, personal electronic device, PED, PDAs)	Weight-related outcomes (weight loss, weight loss maintained)	Overweight and obese adults	Systematic review with qualitative synthesis
16	Antwi (2013), USA	The objective of this review is to synthesise the best available evidence on the effectiveness of web-based programs on the reduction of childhood obesity in school age children.	Web-based technologies (incl., but not limited to the Internet, social networking media, Internet mobile applications, and email)	Weight-related outcomes (e.g., weight change, BMI, waist circumference, body fat percentage)	School-aged children	Systematic review with qualitative synthesis
40	Bennett (2014), USA	This systematic review evaluated the efficacy of eHealth weight management interventions among overweight and obese racial/ethnic minority adults.	Various technologies ("eHealth", incl. computer, web, SMS, mobile phone, apps, email or related technologies, social media)	Weight-related outcomes (e.g., weight change, BMI, waist circumference, body fat percentage)	Overweight or obese, adults of racial/ethnic minorities	Systematic review with qualitative synthesis
78	Chen (2014), USA	The two specific objectives of this review were to evaluate the existing literature reported on the effectiveness of technology-based interventions in preventing obesity in adolescents and to explore components of these interventions that are associated with significant BMI outcomes.	Various technologies (incl. computers, social media, Internet, mobile phones)	BMI or BMI z-score (both self-report and measurement collected using the World Health Organization classification) and one of the health behaviours (diet and PA)	Adolescents	Systematic review with qualitative synthesis

Study ID	Author (Year), Country	Objectives	Technology/Intervention ("focus", relevant keywords)	Relevant Outcomes	Target population	Methodology/Study design
94	Coons (2012), USA	This article systematically reviews the recent literature examining technology-supported interventions for weight loss and maintenance among overweight and obese adults.	Various technologies (incl. Internet, PDAs)	Weight-related outcomes (e.g., weight change, BMI, waist circumference, body fat percentage)	Overweight or obese adults	Systematic review with qualitative synthesis
98	Cushing (2010), USA	The current study quantitatively evaluated the impact of eHealth interventions on paediatric health promoting and maintaining behaviours believed to impact the development or worsening of a physical disease and their associated outcomes.	Various technologies ("eHealth", incl. Internet, eHealth, multimedia, cell phone, PDA, virtual reality, pager, and CD-ROM)	Weight- related outcomes (e.g., weight change, BMI, waist circumference, body fat percentage), PA and diet	Children and adolescents	Systematic review with meta-analysis
181	Heron (2010), USA	The goal of the present review is to synthesize and critique mobile technology-based ecological momentary interventions (EMI) aimed at improving health behaviours and psychological and physical symptoms.	Various technologies (incl. palmtop computers, pocket computers, handheld computers, PDAs, cellular phones, mobile phones, text messages, SMS)	Various health indicators, including weight-related outcomes (e.g., weight change, BMI, waist circumference, body fat percentage), PA and diet	General pop.	Systematic review with qualitative synthesis
199	Joseph (2014), USA	The purpose of this article is to provide a comprehensive up-to-date review of Internet-based physical activity intervention research targeting adult populations.	Web-based interventions (incl. Internet, web, email, but mentioned: mobile/cellular phone technology)	PA	Adults	Systematic review with qualitative synthesis
214	Khaylis (2010), USA	The purpose of this article was to qualitatively review studies that used technology- based interventions for weight loss and to identify specific components of these interventions that are effective in facilitating weight loss.	Various technologies (incl. cell phones, Internet, handheld devices)	Weight-related outcomes (e.g., weight change, BMI, waist circumference, body fat percentage)	General pop.	Systematic review with qualitative synthesis
235	Lau (2011), China	The present review aimed to systematically evaluate the efficacy and methodological quality of ICT-based PA interventions for children and adolescents based on evidence from randomized controlled trials.	Various technologies (incl. Internet, email, mobile phone, SMS)	PA	Children and adolescents	Systematic review with qualitative synthesis

Study ID	Author (Year), Country	Objectives	Technology/Intervention (“focus”, relevant keywords)	Relevant Outcomes	Target population	Methodology/Study design
276	Mehta (2011), USA	The purpose of the study was to systematically analyse interventions that used the Internet or cell phones for promotion of physical activity among adults 21 years and older.	Various technologies (“Internet and cell phones”)	PA, Weight-related outcomes (body fat percentage, waist circumference), cardiovascular risk score	Adults	Systematic review with qualitative synthesis
306	Norman (2007), USA	To review eHealth intervention studies for adults and children that targeted behavior change for physical activity, healthy eating, or both behaviours.	Various technologies (“eHealth”, incl. web, computer, e-mail, multimedia, Internet, PDAs, cell phones)	Various health indicators, including weight-related outcomes (e.g., weight change, BMI, waist circumference, body fat percentage), PA and diet	Children and adults	Systematic review with qualitative synthesis
392	Tang (2014), UK	A systematic review of reviews was conducted to examine the effectiveness of self-directed interventions and to identify intervention content associated with effectiveness.	Various technologies employed in “Self-directed interventions” (incl. Internet, eHealth, home-based, text message)	Various health indicators, including weight-related outcomes (e.g., weight change, BMI, waist circumference, body fat percentage), PA and diet	Adults	Systematic review of reviews with qualitative synthesis

2a) Technologies for measuring behavior (primary research articles)

Study ID	Author (Year), Country	Objectives of the paper	Technologies used	Relevant Outcomes	Target population	Methodology/ Study design
2.1) Design and development (11 studies)						
Mobile technologies (11 studies, 100%)<br>***Study design: Descriptive (11/11, 100%)***						
6	Ahmad (2014), USA	In this paper, we present a user interface for a mobile telephone food record that relies on taking images, using the built-in camera, as the primary method of recording. We describe the design and implementation of this user interface while stressing the solutions	Mobile features: App (using camera)<br>App name: not provided<br>Operative system: iOS	Diet	General pop.	Descriptive
45	Bieber (2009), Germany	In this paper we describe a novel concept of using a mobile phone without any additional devices for physical activity recognition. This enables a permanent, non- obtrusive activity monitoring for everyday usage.	Mobile features: App (using accelerometer)<br>App name: *DiaTrace*<br>Operative system: Java<br>Tested on: Sony Ericsson w910i or w760i	PA	Patients and caregivers	Descriptive
83	Choudhury (2008), USA	Over the past four years, we've been building an automatic activity recognition system using on-body sensors. The Mobile Sensing Platform (MSP) tackles several of these design and deployment challenges. Moreover, we've carried out several real-world deployments and user studies, using the results to improve the hardware, software design, and activity recognition algorithms. The lessons learned have broad relevance to context-aware ubiquitous computing applications.	Mobile features: App (using accelerometer and GPS)<br>App name: *Mobile Sensing Platform, UbiFit Garden*<br>Operative system: Not specified	PA	General pop.	Descriptive
155	Goh Kim (2012), Malaysia	This research focuses on developing an Android-based mobile phone prototype to calculate and determine the duration of physical exercise, time to exercise and the types of exercise needed daily by the user.	Mobile features: App (using accelerometer)<br>App name: not provided<br>Operative system: Android	PA	Adults	Descriptive

Study ID	Author (Year), Country	Objectives of the paper	Technologies used	Relevant Outcomes	Target population	Methodology/ Study design
187	Hughes (2010), USA	We developed an electronic food diary on a mobile phone that includes an energy balance visualization and computes and displays the difference between energy intake from food entries and energy expenditure from a multiple-sensor device that provides objective estimates of energy expenditure in real time.	Mobile features: App (using accelerometer, GPS, and camera)<br>App name: not provided<br>Operative system: Python, Windows Mobile<br>Tested on: Nokia S60; HTC	PA and diet	General pop.	Descriptive<br><br>Trial name: *BALANCE*
192	Intille (2011), USA	This paper describes the motivation for, and overarching design of, an open-source hardware and software system to enable population-scale, longitudinal measurement of physical activity and sedentary behavior using common mobile phones.	Mobile features: App (using accelerometer)<br>App name: *Wockets*<br>Operative system: Windows Mobile	PA	General pop.	Descriptive
207	Ketabdar (2010), Germany	In this paper, we propose a system and methodology for using mobile phones for monitoring physical activities of a user, and its applications in assisting elderly or people with need for special care and monitoring.	Mobile features: App (using accelerometer)<br>App name: *ActivityMonitor*<br>Operative system: iOS<br>Tested on: iPhone 3G	PA	Elderly or people with need for special care and monitoring	Descriptive
** 277<br><br>[Also cited in Section 1a]	Mendi (2013), Turkey	In this paper, we introduce a real-time food intake monitoring system for mobile devices. The proposed system gets acceleration data from the sensor placed on the wrist of the user during a meal. The data is then sent to the mobile device via Bluetooth.	Mobile features: App (using accelerometer and Bluetooth)<br>App name: not provided<br>Operative system: Android	Diet	General pop.	Descriptive
420	Villalobos (2011), Canada	In this article we will propose a smart system that takes advantage of smartphones to build a platform for the monitoring of the amount of calorie intake for obesity patients. The patient uses the built-in camera of the smartphone to take a picture of any food that he/she wants to eat. The system will then process the images to detect the type of food and portion size, and uses the information to estimate the amount of calories.	Mobile features: App (using camera)<br>App name: *Nutrient Intake Monitoring System (NIMS)*<br>Operative system: iOS	Diet	Obese adult patients	Descriptive

Study ID	Author (Year), Country	Objectives of the paper	Technologies used	Relevant Outcomes	Target population	Methodology/ Study design
427	Weiss (2010), USA	[In this paper we present] FIVR (Food Intake Visual and voice Recognizer) is designed to use new digital photographing technology to reduce measurement error associated with a food record. The intent is to create a tool that would both increase accuracy of intake records and reduce the recording burden for respondents. Using a mobile phone with a camera	Mobile features: App (using camera and microphone)<br>App name: *Food Intake Visual and voice Recognizer (FIVR)*<br>Operative system: Not specified	Diet	General pop.	Descriptive
454	Zhu (2008), USA	In this paper, we propose a novel food record method using a mobile device that will provide an accurate account of daily food and nutrient intake among adolescents.	Mobile features: PDA App (using camera and GPS)<br>App name: not provided<br>Operative system: Not specified	Diet	General pop.	Descriptive
2.2) Feasibility (3 studies)						
Mobile technologies (3 studies, 100%)<br>***Study design: Mixed (1/3, 33%); Quantitative (2/3, 66%, of which 1 linked to subsequent RCT)***						
100	Daugherty (2012), USA	To evaluate a defined set of skills among adolescents and adults when using the mobile telephone food record to capture images and to compare the perceptions and preferences between adults and adolescents regarding their use of the mobile telephone food record.	Mobile features: App (using camera)<br>App name: not provided<br>Operative system: Windows Mobile<br>Tested on: HTC p4351	Diet	Adolescents and adults (mostly females)	Mixed
178	Heinonen (2012), Finland	The aim of the present study was to evaluate the usability and feasibility of a mobile phone program in recording of symptoms during a physical exercise trial.	Mobile features: App and SMS<br>App name: not provided<br>Operative system: Symbian	PA	Adults (females only)	Quantitative (related to RCT)
339	Rofey (2010), USA	This study examined the feasibility of using Ecological Momentary Assessment (EMA) to examine important domains relevant to inter-regulatory health processes in overweight adolescent females in their natural environments. The aims of this pilot study were to (i) examine the feasibility of utilizing EMA in overweight children participating in an empirically validated cognitive–behavioural weight-management intervention, and (ii) examine the relationship between EMA and weight (primary) and behavioural outcomes (secondarily; mood and sleep).	Mobile features: App<br>App name: *BodyMedia*<br>Operative system: Not specified	Weight-related and behavioural (PA)	Overweight children and adolescents (females only)	Quantitative

Study ID	Author (Year), Country	Objectives of the paper	Technologies used	Relevant Outcomes	Target population	Methodology/ Study design
2.3) Evaluations (30 studies)						
Mobile technologies (30 studies, 100%)<br>***Study design: Mixed (1/30, 3%, linked to subsequent RCT); Quantitative (29/30, 97%, of which 5 linked to subsequent RCT)***						
4	Aguiar (2014), Portugal	This paper presents a smartphone-based method for classification of inactive postures and physical activities including the calculation of energy expenditure. The implemented solution considers two different positions for the smartphone, the user's pocket or belt.	Mobile features: App (using accelerometer)<br>App name: not provided<br>Operative system: Android	PA	Adults (mostly females)	Quantitative
14	Anguita (2013), Italy	The exploitation of smartphones for Human Activity Recognition (HAR) has been an active research area in which the development of fast and efficient Machine Learning approaches is crucial for preserving battery life and reducing computational requirements. In this work, we present a HAR system, which incorporates smartphone-embedded inertial sensors and uses Support Vector Machines (SVM) for the classification of Activities of Daily Living (ADL).	Mobile features: App (using accelerometer)<br>App name: not provided<br>Operative system: Android	PA	General pop.	Quantitative
15	Antos (2014), USA	The recorded accelerations are not only affected by our activities but also by the phone's location. Here we develop a method to solve this kind of problem, based on the intuition that activities change rarely, and phone locations change even less often. A hidden Markov model (HMM) tracks changes across both activities and locations, enabled by a static support vector machine (SVM) classifier that probabilistically identifies activity-location pairs.	Mobile features: App (using accelerometer)<br>App name: not provided<br>Operative system: Android<br>Tested on: T-Mobile G1	PA	Healthy adults	Quantitative
22	Arif (2014), Saudi Arabia	In this study, acceleration sensor present in the smartphone is used to monitor the physical activity of the user. Physical activities including Walking, Jogging, Sitting, Standing, Walking upstairs and Walking downstairs are classified.	Mobile features: App (using accelerometer)<br>App name: not provided<br>Operative system: Android	PA	General pop.	Quantitative

Study ID	Author (Year), Country	Objectives of the paper	Technologies used	Relevant Outcomes	Target population	Methodology/ Study design
27	Atienza (2006), USA	This preliminary investigation examined whether computerized behavioural strategies (reinforcement, feedback, and tracking) could increase rates of adherence to the health assessments among middle-aged and older adults. In addition, we examined the acceptability of electronic diaries to ecologically assess everyday health in this group and explored the temporal correspondence between computerized physical activity assessments with a standardized measure of physical activity.	Mobile features: PDA App<br>App name: not provided<br>Operative system: Windows Mobile<br>Tested on: Casio Cassiopeia E-125 Pocket PC	PA	Middle-aged and older adults<br>(mostly females)	Quantitative (related to RCT)
38	Beasley (2005), USA	Study objectives were to assess the accuracy of a food record delivered on a personal digital assistant (PDA) and to examine sources of error from the PDA-based food record.	Mobile features: PDA App<br>App name: *DietMatePro*<br>Operative system: Not specified	Diet	Overweight or obese adults (mostly females)	Quantitative
** 37<br><br>[Also cited in Section 1a]	Beasley (2008), USA	The purpose of this 4-week randomized, controlled trial was to evaluate the effectiveness of DietMatePro. Two primary aims were to 1) compare concordance of reported energy and nutrient intakes obtained from DietMatePro and paper food diaries with 24-hr recall; and 2) compare adherence to a strict dietary regimen among users randomized to use DietMatePro versus a paper-based food record.	Mobile features: PDA App<br>App name: *DietMatePro*<br>Operative system: Palm<br>Tested on: PalmZire 21	Diet	Overweight or obese adults (mostly females)	Quantitative (related to RCT)
41	Bergman (2012), USA	This study examined the validity of a selected free pedometer application (iPedometer; IP) for the iPhone that could be used to assess physical activity.	Mobile features: App (using accelerometer)<br>App name: *iPedometer*<br>Operative system: iOS<br>Tested on: iPhone 3G	PA	Adults	Quantitative
44	Bexelius (2011), Sweden	Our objective was to compare within- and between-subject variations in physical activity level by means of cell phones with corresponding estimates using an accelerometer. In addition, we compared the agreement of daily PAL values obtained using the cell phone questionnaire with corresponding data obtained using an accelerometer.	Mobile features: App<br>App name: not provided<br>Operative system: Java	PA	Adults (females only)	Quantitative

Study ID	Author (Year), Country	Objectives of the paper	Technologies used	Relevant Outcomes	Target population	Methodology/ Study design
67	Carter (2013), UK	The present study aimed to validate the diet measures recorded on My Meal Mate against a reference measure of 24 h dietary recalls.	Mobile features: App (using camera)<br>App name: *My Meal Mate*<br>Operative system: Android<br>Tested on: HTC Desire	Diet	Adults (mostly females)	Quantitative (related to RCT)<br><br>Trial name: *My Meal Mate* (NCT01744535)
124	Emken (2012), USA	The purpose of this study was to evaluate the in-laboratory recognition accuracy of KNOWME. KNOWME Networks is a wireless body area network with 2 triaxial accelerometers, a heart rate monitor, and mobile phone that acts as the data collection hub. One function of KNOWME Networks is to detect physical activity (PA) in overweight Hispanic youth.	Mobile features: App<br>App name: *KNOWME*<br>Operative system: Python<br>Tested on: Nokia N95	PA	Overweight adolescents of ethnic minority (males only)	Quantitative<br><br>Trial name: *KNOWME*
163	Guidoux (2014), France	This paper introduces a function dedicated to the estimation of total energy expenditure of daily activities based on data from accelerometers integrated into smartphones. The use of mass-market sensors such as accelerometers offers a promising solution for the general public due to the growing smartphone market over the last decade.	Mobile features: App (using accelerometer)<br>App name: not provided<br>Operative system: Android<br>Tested on: Samsung Galaxy xCover; LG Nexus 4	PA	Adults	Quantitative (related to RCT)<br><br>Trial name: *eMouve* (NCT01995253; NCT01995162)
195	Jia (2012), USA	The objective of this study is to investigate two novel approaches to provide the missing information, enabling food volume estimation from a single image.	Mobile features: App (using camera)<br>App name: not provided<br>Operative system: Not specified	Diet	General pop.	Quantitative
216	Kikunaga (2007), Japan	This study was carried out to examine first, the validity of a new dietary assessment method, a handheld personal digital assistant with camera and mobile phone card, in comparison with a weighed diet record.	Mobile features: PDA App (using camera and phone card)<br>App name: *Wellnavi*<br>Operative system: Not specified	Diet	Adults (mostly females)	Quantitative
232	Lacson (2006), USA	The goals of this paper are two-fold: (1) We propose a framework for acquiring the structure of foods; and (2) We present and evaluate a four-step algorithm that enables automatic extraction of dietary information from these records, which in turn allows automated mapping to the Diet History Questionnaire dietary/nutrient database	Mobile features: App (using camera and microphone)<br>App name: not provided<br>Operative system: Symbian<br>Tested on: Nokia 6600	Diet	General pop.	Quantitative

Study ID	Author (Year), Country	Objectives of the paper	Technologies used	Relevant Outcomes	Target population	Methodology/ Study design
236	Lauritzen (2013), Spain	This study was conducted to determine the accuracy and usefulness of two current commercially available activity trackers in rollator dependent elderly with reduced mobility, compared with elderly with normal mobility and healthy adults.	Mobile features: App<br>App name: *Noom, FitBit Ultra*<br>Operative system: Android<br>Tested on: Samsung GT-I9300	PA	Elderly with reduced and normal mobility, and healthy adults (mostly females)	Quantitative
237	Lee (2012), USA	The objectives of this study were to (1) assess the error of automatically determined portion weights compared to known portion weights of foods and (2) to compare the error between automation and human	Mobile features: App (using camera)<br>App name: not provided<br>Operative system: Windows Mobile<br>Tested on: HTC p4351	PA	Children and adolescents (mostly males)	Quantitative
269	Martin (2009), USA	The aim of the present study was to report the first reliability and validity tests of the remote food photography method (RFPM), which consists of camera-enabled cell phones with data transfer capability.	Mobile features: PDA App (using camera)<br>App name: *Remote Food Photography Method (RFPM)*<br>Operative system: Palm<br>Tested on: PalmZire 72; Motorola i860	Diet	Adults (mostly females)	Quantitative (related to RCT)
** 273<br><br>[Also cited in Section 1a]	Mattila (2010), Finland	In the present study, we used data from an earlier study, and examined the validity of self-observed weight measures as well as behavioural patterns in those successful and unsuccessful in weight loss.	Mobile features: App<br>App name: *Wellness Diary*<br>Operative system: Symbian	Weight-related and behavioural (PA, diet)	Adults (mostly males)	Quantitative
274	McClung (2009), USA	The purpose of this study was to compare energy expenditure measured using DLW with self-reported energy intake using a PDA or written food record (written record) in weight-stable individuals over 7 days.	Mobile features: PDA App<br>App name: *BalanceLog*<br>Operative system: Not specified	Diet	Adults	Mixed (related to RCT)
322	Pfaeffli (2013), New Zealand	The aims of this study were to determine the convergent validity of a mobile phone physical activity questionnaire against accelerometry in people with cardiovascular disease (CVD), and to compare how the app questionnaire performed compared with the commonly used self-recall International Physical Activity Questionnaire (IPAQ).	Mobile features: App<br>App name: *MobilePAL*<br>Operative system: Android	PA	Overweight or obese adults with CVD (mostly males)	Quantitative

Study ID	Author (Year), Country	Objectives of the paper	Technologies used	Relevant Outcomes	Target population	Methodology/ Study design
370	Six (2011), USA	The development of a mobile telephone food record (mpFR) in which image analysis and volume estimation data can be indexed with the Food and Nutrient Database for Dietary Studies (FNDDS) has the potential to improve the accuracy of dietary assessment. The objective of this analysis was to compare the measured energy and protein content of foods to the published values in the FNDDS.	Mobile features: App (using camera)<br>App name: *mpFR* (mobile telephone food record)<br>Operative system: Not specified	PA	Children and adolescents (mostly males)	Quantitative
374	Smith (2014), USA	This paper addresses the need for diet assessment methods that capture the rapidly changing beverage consumption patterns in China. The objective of this study was to develop a 3-day smartphone-assisted 24-hour recall to improve the quantification of beverage intake and validate the extent to which the written record and smartphone-assisted recalls adequately estimated total fluid intake, using 24-hour urine samples.	Mobile features: App<br>App name: not provided<br>Operative system: Not specified	Weight-related and behavioural (PA, diet)	Adults of Chinese ethnicity (mostly males)	Quantitative (related to RCT)
391	Sun (2011), China	This app recognizes people’s physical activities when they place the mobile phone in the pockets near the pelvic region.	Mobile features: App (using accelerometer)<br>App name: not provided<br>Operative system: Symbian<br>Tested on: Nokia N97	PA	Adults	Quantitative
417	Vathsangam (2013), USA	In this paper, we present a practical solution for estimating an individual’s energy expenditure using mobile phones in real-life ambulatory settings.	Mobile features: App (using accelerometer)<br>App name: not provided<br>Operative system: Android<br>Tested on: Samsung Galaxy Nexus S; Samsung Galaxy Nexus 4	PA	Adults (mostly males)	Quantitative
439	Wu (2012), USA	To examine the validity of the iPod Touch (Apple, Inc.) and particularly to understand the value of using gyroscopes for classifying types of physical activity, with the goal of creating a measurement and feedback system that easily integrates into individuals’ daily living.	Mobile features: App (using accelerometer)<br>App name: not provided<br>Operative system: iOS<br>Tested on: iPod Touch	PA	Adults (mostly females)	Quantitative

Study ID	Author (Year), Country	Objectives of the paper	Technologies used	Relevant Outcomes	Target population	Methodology/ Study design
441	Xia (2011), Australia	The purpose of this study is to translate the rule-based classification algorithm and modify for healthy subjects using the accelerometer on a smart phone. This will provide an application that automatically classifies one's physical activity, either for wellness of healthy people or clinical assessment of patients in hospitals and at home.	Mobile features: App (using accelerometer)<br>App name: not provided<br>Operative system: iOS<br>Tested on: iPhone	PA	Adult patients (mostly males)	Quantitative
443	Xue (2014), China	In this paper we propose a new feature for activity recognition from tri-axial acceleration signals. To improve the classification performance, two fusion methods, minimal distance optimization (MDO) and variance contribution ranking (VCR) are proposed.	Mobile features: App (using accelerometer)<br>App name: not provided<br>Operative system: Android<br>Tested on: HTC; Samsung	PA	General pop.	Quantitative
445	Yon (2006), USA	The goal of this study was to investigate whether the use of a personal digital assistant (PDA) for dietary self-monitoring would reduce underreporting prevalence and improve the validity of self-reported energy intake.	Mobile features: PDA App<br>App name: *Calorie King's Diet Diary*<br>Operative system: Palm<br>Tested on: PalmZire 21	Diet	Adults (mostly females)	Quantitative
450	Yusuf (2013), Australia	The main aim of this study was to gain new knowledge on using off-the-shelf wearable sensors for conducting activity recognition by a computational approach based on learning from patters in the data. Annotated data were first collected with a fixed unobtrusive sensor as part of specific everyday basic activities to achieve the objectives: - contribute to the understanding of how artificial intelligence (machine learning) methods can be applied to tri-axial accelerometer data signals; - determine a useful automated methodology for classifying the data into a range of different human activities of interest.	Mobile features: App (using accelerometer)<br>App name: not provided<br>Operative system: iOS<br>Tested on: iPhone	PA	Adults	Quantitative
2.4) Design and Feasibility (10 studies)						
Mobile technologies (9 studies, 90%)<br>***Study design: Mixed (5/9, 56% of which 1 related to subsequent RCT); Quantitative (4/9, 44%)***						

Study ID	Author (Year), Country	Objectives of the paper	Technologies used	Relevant Outcomes	Target population	Methodology/ Study design
21	Arab (2010), USA	To explore the frequency of imaging needed to capture all foods eaten, we examined the number of images of individual foods consumed in a pilot study of automated imaging using camera phones set to an image-capture frequency of one snapshot every 10 seconds.	Mobile features: App (using camera)<br>App name: *Image-Diet Day*<br>Operative system: Symbian<br>Tested on: Nokia N80	Diet	Adults	Mixed
86	Chung (2009), China	The objective of this research was to design and develop an interactive dietary recording instrument with dietary education and to evaluate its usability.	Mobile features: App (using camera)<br>App name: *eDiet*<br>Operative system: Not specified	Diet	Adults (mostly females)	Quantitative
85	Chung-Tse (2014), Taiwan	This paper proposed a context-aware prompting system to maintain or increase physical activity for personal healthcare management. The proposed mechanism utilizes a smart phone to collect activity context and transmit to remote server for context-aware.	Mobile features: App (using accelerometer)<br>App name: not provided<br>Operative system: Android<br>Tested on: HTC Desire A8181	PA	Adults	Quantitative
147	Gao (2009), USA	This paper presents a novel health-aware smart phone system, which utilizes the embedded accelerometer to monitor daily physical activities and the built-in camera to analyse food items.	Mobile features: App (using GPS)<br>App name: *HealthAware*<br>Operative system: Windows Mobile<br>Tested on: HTC Touch Diamond	PA and diet	General pop.	Quantitative
** 158<br><br>[Also cited in Section 1a]	Greeff (2013), New Zealand	In this research we developed a smartphone application, which assists users with learning and monitoring exercises. A key feature of the application is a novel algorithm for analysing accelerometer data and automatically counting repetitive exercises.	Mobile features: App (using accelerometer)<br>App name: *My Personal Trainer*<br>Operative system: iOS<br>Tested on: iPhone	PA	Adolescents and adults	Quantitative
285	Möller (2012), Germany	We present a personal trainer for ubiquitous monitoring and assessment of physical activity using standard fitness equipment. The system records and analyses exercises using the sensors of a personal smartphone attached to the gym equipment.	Mobile features: App (using accelerometer)<br>App name: *GymSkill* (VMI Fit)<br>Operative system: Android	PA	Adults (mostly females)	Mixed

Study ID	Author (Year), Country	Objectives of the paper	Technologies used	Relevant Outcomes	Target population	Methodology/ Study design
371	Six (2010), USA	Mobile telephones with an integrated camera can provide a unique mechanism for collecting dietary information that reduces burden on record-keepers. Objectives for this study were to test whether participant's proficiency with the mobile telephone food record improved after training and repeated use and to measure changes in perceptions regarding use of the mobile telephone food record after training and repeated use.	Mobile features: App (using camera)<br>App name: not provided<br>Operative system: Windows Mobile<br>Tested on: HTC p4351	Diet	Children and adolescents (mostly females)	Mixed
** 401<br><br>[Also cited in Section 1a]	Tsai (2006), USA	In this paper, we describe the Patient-Centred Assessment and Counseling Mobile Energy Balance (PmEB) cell phone application that allows users to self-monitor caloric balance in real time. In this paper, we first discuss related work and the design and implementation of PmEB. Then, we describe the four-phase development methodology. The findings of the first three phases, first detailed in an earlier work-in-progress paper, are summarized. Next, we report findings from our feasibility study.	Mobile features: App<br>App name: *PmEB*<br>Operative system: Java<br>Tested on: Nokia 6600, Motorola V300, Motorola RAZR V3xx	PA and diet	Overweight or obese adult patients (mostly females)	Mixed (related to RCT)
433	Wohlers (2009), USA	This paper describes tests of the usability of an electronic diary in the form of a smart phone to monitor food intake and PA in college students.	Mobile features: App (using GPS)<br>App name: not provided<br>Operative system: Windows Mobile<br>Tested on: Motorola Q9h	PA and diet	Adults	Mixed
Mobile and Web 2.0 technologies (1 study, 10%)<br>Study design: Mixed (1/1, 100%)						
338	Rodrigues (2013), Portugal	The objective of this work was the proposal, design, construction and validation of a mobile health system for dietetic monitoring and assessment. This application may be personalized to keep a daily personal health record of an individual's food intake and daily exercise and to share this with a social networking [component].	Mobile features: App (using accelerometer and camera) and SMS<br>App name: *SapoFitness*<br>Operative system: Android, iOS<br><br>Web 2.0 features: Sharing on Facebook, Twitter	PA and diet	Adults	Mixed
2.5) Design and Evaluations (24 studies)						
Mobile technologies (23 studies, 96%)<br>Study design: Mixed (7/23, 30%); Quantitative (16/23, 70%);						

Study ID	Author (Year), Country	Objectives of the paper	Technologies used	Relevant Outcomes	Target population	Methodology/ Study design
12	Altini (2014), Netherlands	In this paper, we propose a data fusion approach to the problem of physical activity recognition and walking speed estimation using smartphones. Our architecture combines different sensors to take into account practical issues arising in realistic settings, such as variability in phone location and orientation.	Mobile features: App (using accelerometer and GPS)<br>App name: not provided<br>Operative system: Android<br>Tested on: Google Nexus 4	PA	Adults (mostly males)	Quantitative
20	Arab (2011), USA	This study examines the use of a novel, cost-effective image-assisted recall method that combines automatic image capture (to reduce participant reactivity) with a web-based 24-h dietary recall. The goal was to test the feasibility of this approach to enhance dietary assessment.	Mobile features: App (using camera)<br>App name: *Image-Diet Day*<br>Operative system: Symbian<br>Tested on: Nokia N80	Diet	Adults (mostly females)	Mixed
79	Chen (2010), USA	We propose leveraging mobile phones to make this process faster, easier and automatic. In this paper, we demonstrate the feasibility of the first stage: identifying dishes from videos to assist in calorie estimation.	Mobile features: App (using camera)<br>App name: not provided<br>Operative system: Not specified	Diet	General pop.	Quantitative
114	Donaire-Gonzalez (2013), Spain	We sought to validate estimates of physical activity and determine the usability for large population-based studies of the smartphone-based software.	Mobile features: App<br>App name: *CalFit*<br>Operative system: Android<br>Tested on: Google G1	PA	Adults (mostly females)	Mixed
121	Edgar (2012), USA	In the paper we demonstrate feasibility of real-time recognition of multiple household and athletic activities on a cell phone using the data collected by a wearable sensor system consisting of a sensor and a wrist accelerometer.	Mobile features: App (using accelerometer)<br>App name: *SmartShoe*<br>Operative system: Windows Mobile<br>Tested on: Omnia I8000	PA	General pop.<br>(Adults (males only)	Mixed
172	He (2013), China	This study utilized three built-in kinematic sensors in a smartphone to recognize PA and found out which features derived from the three sensors were significant to different PA.	Mobile features: App (using accelerometer)<br>App name: not provided<br>Operative system: Android<br>Tested on: Samsung I9023, Google Nexus S	PA	General pop.	Quantitative
209	Khalil (2009), UAE	In this paper, we present the design, implementation, and evaluation of a step counter application.	Mobile features: App (using accelerometer)<br>App name: *StepUp*<br>Operative system: Symbian<br>Tested on: Nokia N95	PA	General pop.<br>(Age not specified, males and females)	Quantitative

Study ID	Author (Year), Country	Objectives of the paper	Technologies used	Relevant Outcomes	Target population	Methodology/ Study design
212	Khanna (2010), USA	In this paper, we describe the Technology Assisted Dietary Assessment (TADA) project at Purdue University. Dietary intake, what someone eats during the course of a day, provides valuable insights for mounting intervention programs for prevention of many chronic diseases such as obesity and cancer. An overview of our methods used in the TADA project is presented.	Mobile features: App (using camera)<br>App name: not provided<br>Operative system: Nokia Maemo<br>Tested on: Nokia N810 Internet Tablet	Diet	General pop. (Adolescents, gender not specified)	Quantitative<br><br>Trial name: *TADA*
228	Kong (2012), USA	This paper presents a mobile phone based system, to help assess food intakes with few human interventions.	Mobile features: App (using camera)<br>App name: *DietCam*<br>Operative system: iOS<br>Tested on: iPhone 3GS	Diet	General pop.	Quantitative
239	Lee (2011), Korea	This study is part of the ongoing development of treatment methods for metabolic syndrome (MS) project, which involves monitoring daily physical activity. In this study, we have focused on detecting walking activity from subjects which includes many other physical activities such as standing, sitting, lying, walking, running, and falling.	Mobile features: App (using accelerometer)<br>App name: not provided<br>Operative system: iOS<br>Tested on: iPod	PA	Adults (mostly males)	Mixed
262	Maitland (2006), UK	We describe a prototype application that tracks the daily exercise activities of people carrying phones, using fluctuation in signal strength to estimate a user's movement.	Mobile features: App (using accelerometer and GPS)<br>App name: *Shakra*<br>Operative system: Windows Mobile	PA	Adults	Mixed
270	Martín (2013), Spain	This work focuses on exploring the use of smartphones to perform activity recognition without interfering in the user's lifestyle. Thus, we study how to build an activity recognition system to be continuously executed in a mobile device in background mode.	Mobile features: App (using accelerometer)<br>App name: not provided<br>Operative system: Android<br>Tested on: Google Nexus S	PA	Adults (mostly males)	Quantitative
302	Nolan (2014), Canada	Our purpose was to establish proof-of-concept that a smartphone could record acceleration during physical activity, and those data could be modelled to predict activity type (walking or running), speed, and energy expenditure (METs). An application to record and e-mail accelerations was developed for the Apple iPhone/iPod Touch.	Mobile features: App<br>App name: not provided<br>Operative system: iOS<br>Tested on: iPhone; iPod Touch	PA	Adults (mostly females)	Mixed

Study ID	Author (Year), Country	Objectives of the paper	Technologies used	Relevant Outcomes	Target population	Methodology/ Study design
321	Pernek (2013), Slovenia	The contributions of this paper are summarized as follows:<br>• We design a robust dynamic time warping-based algorithm suitable for smartphones to detect exercise repetitions in a continuous acceleration stream.<br>• We present an experimental study to evaluate the algorithm's accuracy in terms of correctly detected repetitions and temporal accuracy of detection of repetition start and end times. Therefore, we conducted experiments observing ten users in two different environments while performing nine different exercises. We describe the outcomes and summarize the results.	Mobile features: App (using accelerometer)<br>App name: not provided<br>Operative system: Android	PA	Adolescents and adults (mostly males)	Quantitative
328	Puri (2009), USA	We present a system that improves accuracy of food intake assessment using computer vision techniques. Our solution is to use a mobile phone to capture images of foods, recognize food types, estimate their respective volumes and finally return quantitative nutrition information.	Mobile features: App (using camera)<br>App name: *Veggie Vision*<br>Operative system: Not specified	Diet	General pop.	Quantitative
331	Rahman (2012), Australia	In this paper we will present a new method for generating texture features from food images and demonstrate that this new feature provides greater food classification accuracy for a mobile phone based dietary assessment system.	Mobile features: App (using camera)<br>App name: not provided<br>Operative system: iOS<br>Tested on: iPhone	Diet	General pop.	Quantitative:<br><br>Trial name: *TADA*
330	Rahman (2012), Australia	In this paper we propose a food volume estimation approach that requires only a pair of stereo images to be captured.	Mobile features: App (using camera)<br><br>App name: not provided<br><br>Operative system: iOS<br>Tested on: iPhone 4S	Diet	General pop.	Quantitative<br><br>Trial name: Technology Assisted Dietary Assessment (TADA)

Study ID	Author (Year), Country	Objectives of the paper	Technologies used	Relevant Outcomes	Target population	Methodology/ Study design
394	Thatte (2012), USA	This article presents the KNOWME platform, a complete, end-to-end, body area sensing system that integrates off-the-shelf biometric sensors with a Nokia N95 mobile phone to continuously monitor the metabolic signals of a subject. Design robustness is achieved by creating a three-tiered sensor data collection architecture.	Mobile features: App (using accelerometer)<br><br>App name: not provided<br><br>Operative system: Symbian<br>Tested on: Nokia N95	PA	Adolescents and adults (mostly females)	Quantitative<br><br>Trial name: KNOWME
416	Vankipuram (2012), USA	In this work, we present a mobile app developed on the iOS platform that monitors activity levels using accelerometry. The data captured by the sensor is utilized to provide real-time motivational feedback to enable reinforcement of positive behaviours in older adults.	Mobile features: App (using accelerometer)<br><br>App name: *ReadySteady*<br><br>Operative system: iOS<br>Tested on: iPod Touch	PA	Older adults	Quantitative
** 410<br><br>[Also cited in Section 1a]	Valentin (2013), USA	In the following paper, we compare two methods for engaging individuals in exercise based on passive versus active-encouragement. The passive method utilizes a wearable device that records exercise activities throughout the day whereas the active-encouragement approach utilizes a smartphone device to send encouraging reminders to the user during the day.	Mobile features: App<br>App name: *Fit Up*<br>Operative system: Android<br>Tested on: LG Optimus	PA	Adolescents and adults (mostly males)	Quantitative
442	Xu (2014), USA	This paper presents a novel end-to-end system solution to some of these challenges. The system is built on the prescription-based context-driven activity classification methodology. Finally, we use a series of experimental field evaluations to confirm the expected benefits of the proposed system in terms of classification accuracy, rate, and sensor operating life.	Mobile features: App<br><br>App name: not provided<br><br>Operative system: Android<br>Tested on: Google Nexus	PA	Adults	Quantitative
452	Zhong (2010), China	In this paper, we design a system to measure the patient's activity by estimating his walking habits. The system, ready to be integrated in a mobile health application, consists of an inertial sensor with a tri-axial orthogonal accelerometer attached to patient's foot, while the sensor is connected to a smart phone for data processing.	Mobile features: App (using accelerometer)<br><br>App name: not provided<br><br>Operative system: Java	PA	Adults	Quantitative

Study ID	Author (Year), Country	Objectives of the paper	Technologies used	Relevant Outcomes	Target population	Methodology/ Study design
453	Zhu (2010), USA	In this paper, we describe a novel mobile telephone food record that will provide an accurate account of daily food and nutrient intake. Our approach includes the use of image analysis tools for identification and quantification of food that is consumed at a meal.	Mobile features: App (using camera and GPS)<br>App name: not provided<br>Operative system: iOS, Nokia Maemo<br>Tested on: iPhone; Nokia N810 Internet Tablet	Diet	General pop.	Quantitative
Mobile and Web 2.0 technologies (1 study, 4%)<br>***Study design: Mixed (1/1, 100%)***						
455	Zongjian (2013), China	Motivated by the competition and achievement system in many video games, we propose Runking, a mobile social network based persuasion system for running exercise.	Mobile features: App<br>App name: *Runking*<br>Operative system: Android<br><br>Web 2.0 features: Custom social networking site	PA	General pop.	Quantitative
2.6) Feasibility and Evaluations (17 studies)						
Mobile technologies (17 studies, 100%)<br>***Study design: Mixed (2/17, 12%); Quantitative (15/17, 88%)***						
43	Bexelius (2010), Sweden	This study evaluates repeated measures of physical activity level (PAL) and the feasibility of using a Java-based questionnaire downloaded onto cell phones for collection of such data. The data obtained were compared with reference estimates based on the doubly labelled water method and indirect calorimetry.	Mobile features: App<br>App name: not provided<br>Operative system: Java	PA	Adults (females only)	Quantitative
52	Boushey (2009), USA	This paper describes the pilot study that was used to determine adolescents' preferences for reporting dietary intake and the early development of using mobile devices for dietary assessment.	Mobile features: PDA App<br>App name: not provided<br>Operative system: Not specified	Diet	Overweight or obese children and adolescents (mostly males)	Mixed
119	Dunton (2011), USA	The present study tested the feasibility, acceptability, and validity of an electronic Ecological Momentary Assessment (EMA) protocol using electronic surveys administered on the display screen of mobile phones to assess children's physical activity and sedentary behaviours.	Mobile features: App<br>App name: *MyExperience*<br>Operative system: Windows Mobile<br>Tested on: HTC Shadow	PA	Sedentary children and adolescents	Mixed<br><br>Trial name: *Healthy PLACES*

Study ID	Author (Year), Country	Objectives of the paper	Technologies used	Relevant Outcomes	Target population	Methodology/ Study design
120	Dunton (2012), USA	Research tested the feasibility and validity of an Ecological Momentary Assessment (EMA) self-report protocol using electronic surveys on mobile phones to assess adults' physical activity and sedentary behaviours.	Mobile features: App<br>App name: *MyExperience*<br>Operative system: Windows Mobile<br>Tested on: HTC Shadow	Weight-related and behavioural (PA, diet)	Sedentary adults (mostly females)	Quantitative<br><br>Trial name: *Healthy PLACES*
135	Fowles (2008), USA	The purpose of this study is to determine perceptions of accuracy and ease of use of PDA-based dietary records in a sample of low-income pregnant women and the feasibility of using PDA-based dietary record software in this population.	Mobile features: PDA App<br>App name: *BalanceLog*<br>Operative system: Not specified	Diet	Adults (females only)	Quantitative
141	Fukuo (2009), Japan	The aim of the study was to assess the accuracy and feasibility of use of a new PDA-based food diary, including food photographs.	Mobile features: PDA App (using camera)<br>App name: not provided<br>Operative system: OpenZaurus<br>Tested on: Sharp Zaurus SL-C3000	Diet	Adults (mostly females)	Quantitative
143	Fukuoka (2011), USA	The purposes of this study were 1) to determine compliance with a pedometer and mobile phone-based physical activity diary, and 2) to assess concordance between self-reported daily steps recorded and transmitted by a mobile phone and pedometer-measured daily steps in sedentary women.	Mobile features: App and SMS<br>App name: not provided<br>Operative system: Not specified<br>Tested on: Motorola RAZR V3xx	PA	Sedentary adults (females only)	Quantitative
164	Guiry (2014), Ireland	In this paper, the authors describe a method of accurately detecting human activity using a smartphone accelerometer paired with a dedicated chest sensor. The design, implementation, testing and validation of a custom mobility classifier are also presented.	Mobile features: App (using accelerometer)<br>App name: not provided<br>Operative system: Android<br>Tested on: Samsung Galaxy S	PA	Adults (mostly females)	Quantitative
203	Kelly (2012), Ireland	In this paper, we investigate the feasibility of using a modern smartphone, with limited placement constraints, as the sensing modality for an activity recognition system.	Mobile features: App (using accelerometer)<br>App name: not provided<br>Operative system: Android<br>Tested on: Google Nexus S	PA	General pop.	Quantitative
230	Kósa (2014), Hungary	The paper describes the first, preclinical evaluation of a dietary logging application developed at the University of Pannonia, Hungary. The mobile user interface is briefly introduced. The three evaluation phases examined the completeness and contents of the dietary database and the time expenditure of the mobile-based diet logging procedure.	Mobile features: App (using camera)<br>App name: *Lavinia*<br>Operative system: Android	Diet	General pop.	Quantitative

Study ID	Author (Year), Country	Objectives of the paper	Technologies used	Relevant Outcomes	Target population	Methodology/ Study design
267	Martin (2012), USA	Herein, the results from two studies are reported that examined the reliability and validity (accuracy) of the RFPM at estimating energy and nutrient intake.	Mobile features: App (using camera)<br>App name: *Remote Food Photography Method (RFPM)*<br>Operative system: Not specified	Diet	Overweight or obese adults (mostly females)	Quantitative
327	Ptomey (2013), USA	The objective of this study was to examine the feasibility and outcomes of combining photos with 24-hour dietary recalls for the assessment of energy and macronutrient intakes in adults with intellectual and developmental disabilities.	Mobile features: App (using camera)<br>App name: not provided<br>Operative system: iOS<br>Tested on: iPad	Diet	Overweight or obese adults (mostly males)	Quantitative
348	Schiel (2010), Germany	We conducted a pilot trial of electronic technology integrated into the treatment of children and adolescents who are overweight or obese. The present trial was designed to answer the following questions. First, can modern electronic technology be integrated into the therapy of children and adolescents who are overweight or obese? Second, is it possible to identify the differences between the subjects' self-reported physical activity and eating habits and the actual data recorded by the technical devices?	Mobile features: App (using accelerometer and camera)<br>App name: *DiaTrace, MoSeBO*<br>Operative system: Symbian<br>Tested on: Sony Ericsson	PA and diet	Overweight or obese children and adolescents (mostly males)	Quantitative
349	Schiel (2011), Germany	To assess physical activity in patients with Type 1 Diabetes an innovative telemedical support system has been developed. In 2008 a scientific project was started with the aim of the development of a system to measure physical activity. The aim of the feasibility trial was to prove its use in a clinical setting.	Mobile features: App (using accelerometer and camera)<br>App name: *DiaTrace*<br>Operative system: Symbian<br>Tested on: Sony Ericsson	PA	Overweight or obese children and adolescents with T1D	Quantitative
347	Schiel (2012), Germany	The present pilot trial aimed to answer the following two questions: 1) Can modern electronic healthcare technology be integrated into current therapy of children and adolescents with overweight or obesity? 2) Is there any discrepancy between self-report (physical activity and eating habits) and objectively recorded data?	Mobile features: App (using accelerometer and camera)<br>App name: *DiaTrace, MoSeBO*<br>Operative system: Symbian<br>Tested on: Sony Ericsson	PA and diet	Overweight or obese children and adolescents (mostly females)	Quantitative

Study ID	Author (Year), Country	Objectives of the paper	Technologies used	Relevant Outcomes	Target population	Methodology/ Study design
384	Sternfeld (2012), USA	Physical activity diaries reduce the recall error inherent in self-reported PA but are burdensome. The purpose of this study was to compare a cell phone-based diary with a paper diary and examine the reliability and validity of the cell phone diary.	Mobile features: App<br>App name: *BeWell*<br>Operative system: Not specified	PA	Adults	Quantitative
421	Wang (2006), Japan	With the aim of developing a new dietary instrument for assessing an individual's usual intakes, we evaluated a hand-held personal digital assistant with camera and mobile telephone card.	Mobile features: PDA App (using camera)<br>App name: *Wellnavi*<br>Operative system: Not specified	Diet	Adults (females only)	Quantitative
2.7) Design, Feasibility, and Evaluations (7 studies)						
Mobile technologies (7 studies, 100%)<br>***Study design: Mixed (7/7, 100%, of which 2 linked to subsequent RCT)***						
112	Doherty (2012), Canada	To present the design and pilot test results of a continuous multi-sensor monitoring system of real-world physiological conditions and daily life (activities, travel, exercise, and food consumption), culminating in a Web-based graphical decision-support interface.	Mobile features: App (using accelerometer and GPS)<br>App name: not provided<br>Operative system: BlackBerry	PA and diet	Overweight or obese adults with T2D	Mixed
113	Doherty (2014), Canada	The objective of this paper is to demonstrate how passive (quantitative) tracking of human activity using GPS/accelerometers can be enhanced with interactive in-situ (qualitative) experience sampling of subjects' psychological/emotional wellbeing using smartphone-based technologies.	Mobile features: App (using accelerometer and GPS)<br>App name: not provided<br>Operative system: BlackBerry<br>Tested on: Storm2 9550	PA	Adults (mostly males)	Mixed
171	He (2014), USA	In this paper, we present a smartphone application, which runs in the background of users' smartphones and monitors their daily physical activity continuously. Unlike traditional pedometers that only passively count steps and estimate burnt calories, On11 also detects sedentary behaviours (sitting, lying down).	Mobile features: App (using accelerometer)<br>App name: *On11*<br>Operative system: Android	PA	Sedentary adults	Mixed
202	Kawano (2013), Japan	We propose a mobile food recognition system the purposes of which are estimating calorie and nutritious of foods and recording a user's eating habits.	Mobile features: App (using camera)<br>App name: not provided<br>Operative system: Android<br>Tested on: Samsung Galaxy Note II	Diet	General pop. (Adults, gender not specified)	Mixed

Study ID	Author (Year), Country	Objectives of the paper	Technologies used	Relevant Outcomes	Target population	Methodology/ Study design
252	Long (2013), USA	This study tested the effectiveness of using cell phones with digital pictures to prompt memory and mypyramidtracker.gov to estimate self-reported fruit and vegetable intake.	Mobile features: App (using camera)<br>App name: not provided<br>Operative system: Not specified	Diet	Adults (mostly females)	Mixed (related to RCT)
340	Rollo (2011), Australia	We evaluated a mobile phone application for recording dietary intake.	Mobile features: PDA App (using camera)<br>App name: *Nutricam*<br>Operative system: Symbian<br>Tested on: Sony Ericsson K800i	Diet	Overweight or obese older adult patients with T2D<br>(mostly males)	Mixed
378	Spook (2013), Netherlands	To examine the feasibility, usability, and ecological validity of an application (app) with regard to DI and PA among Dutch vocational education students.	Mobile features: App<br>App name: *mEMA*<br>Operative system: Android, Blackberry, iOS	PA and diet	Vocational education students	Mixed (related to RCT)

2b) Technologies for measuring behavior (reviews)

Study ID	Author (Year), Country	Objectives of the paper	Technologies used	Relevant Outcomes	Target population	Methodology/ Study design
2.1) Design and development (3 studies)						
Mobile technologies						
268	Martin (2014), USA	The present review describes recently validated methods of food intake estimation that utilise digital images and outlines how these methods have been adapted to estimate food intake in a number of environments, including free-living conditions, Head Start settings and children's homes.	Mobile technologies ("Smartphones", used to capture photos of foods using an app: *Food Photography Application*)	Diet	Children and adults	Non-systematic review with qualitative synthesis
346	Schap (2014), USA	This review summarises the system design and the evidence-based development of image-based methods for dietary assessment among children.	Mobile technologies ("Smartphones", used to capture photos of foods, used in the trial: *Technology Assisted Dietary Assessment, TADA*)	Diet	Children and adolescents	Non-systematic review with qualitative synthesis
388	Stumbo (2013), USA	This paper describes six projects sponsored by the United States National Institutes of Health that use digital methods to improve food records and two mobile phone applications using crowdsourcing.	Mobile technologies ("Digital methods and mobile technologies") as employed in six studies	Diet	General pop.	Non-systematic review with qualitative synthesis
2.2) Feasibility (1 study)						
Mobile technologies						
** 356<br><br>[Also cited in "Evaluatio ns"]	Sharp (2014), Australia	This review aims to evaluate the validity, feasibility, and acceptability of dietary assessment methods that have been deployed on mobile phone platforms.	Mobile technologies (incl. smartphones, apps, mobile phones, cell-phones)	Diet	General pop.	Systematic review with qualitative synthesis
2.3) Evaluations (8 studies)						
Mobile technologies						
* 51<br><br>[Also cited in Section 1b]	Bort-Roig (2014), Australia	This study systematically reviewed evidence on smartphones and their viability for measuring and influencing physical activity.	Mobile technologies ("Smartphones", incl. mobile phones, cell-phones, smartphones; excluding text messaging)	PA	General pop.	Systematic review with qualitative synthesis

Study ID	Author (Year), Country	Objectives of the paper	Technologies used	Relevant Outcomes	Target population	Methodology/ Study design
242	Lieffers (2012), Canada	In this review, we compile and discuss research on dietary intake recording applications for mobile devices in healthy populations and those trying to lose weight. The goal was to understand how use of such applications compares with use of conventional methods, to provide information about appropriate use of this technology in dietetic practice (including barriers to and challenges with use), and to identify future research directions.	Mobile technologies (incl. smartphone, mobile phone, mobile electronic device, personal digital assistant, computers-handheld)	Diet	Healthy and those trying to lose weight	Systematic review with qualitative synthesis
** 308<br><br>[Also cited in Section 1b]	O'Reilly (2013), USA	A comprehensive systematic review of how mobile technology has been used for measuring PA and promoting PA behavior change.	Mobile technologies (incl. mobile phones, SMS, Internet, web email as keywords)	PA	General pop.	Systematic review with qualitative synthesis
** 343<br><br>[Also cited in Section 3]	Rusin (2013), Slovakia	The objective of this paper was to summarize existing approaches to self-management of food intake recording and to analyse the functionalities and input methods.	Mobile technologies ("Smartphone apps" used for self-management of food intake (not video or audio analysis)	Diet	General pop.	Systematic review with qualitative synthesis
** 356<br><br>[Also cited in "Feasibility"]	Sharp (2014), Australia	This review aims to evaluate the validity, feasibility, and acceptability of dietary assessment methods that have been deployed on mobile phone platforms.	Mobile technologies (incl. smartphones, apps, mobile phones, cell-phones)	Diet	General pop.	Systematic review with qualitative synthesis
Various technologies, including mobile, web, computers						
191	Illner (2012), France	To report on the inventory of available innovative technologies for dietary assessment and to critically evaluate their strengths and weaknesses as compared with the conventional methodologies (i.e. Food Frequency Questionnaires, food records, 24-hour dietary recalls) used in epidemiological studies.	Various technologies (incl. Internet, web, digital, PDAs, computer, electronic technology and technology	Diet	General pop.	Systematic review with qualitative synthesis
253	Long (2010), USA	The purpose of this article is to present a review of the evidence on the effectiveness of technology-based methods for dietary assessment, which included fruit and vegetable consumption.	Various technologies (incl. computers, web, technologies in general)	Diet	General pop.	Systematic review with qualitative synthesis

Study ID	Author (Year), Country	Objectives of the paper	Technologies used	Relevant Outcomes	Target population	Methodology/ Study design
298	Ngo (2009), Spain	The purpose of this literature review was to describe and evaluate the applications of ICT in dietary intake assessment. The aim was to explore a broad range of studies that assessed the use of new as well as renewed technologies to obtain dietary data.	Various technologies (incl. ICT, technology, PDAs, computers, Internet, radio frequency, digital photography)	Diet	General pop.	Systematic review with qualitative synthesis

3) Overviews of apps and social media content

Study ID	Author (Year), Country	Objectives of the paper	Technologies used	Relevant Outcomes	Target population	Methodology/ Study design
Mobile technologies (21 studies)						
8	Akker (2014), Netherlands	In this article we report a survey of tailoring techniques currently employed in state of the art real time physical activity coaching systems. We present a survey of state of the art activity coaching systems as well as a conceptual framework.	Coaching systems and mobile apps available from searches in PubMed	PA	General pop.	Content Analysis
29	Azar (2013), USA	The goal of the study was to evaluate diet/nutrition and anthropometric tracking apps based on incorporation of features consistent with theories of behavior change.	Mobile apps, available from iTunes App Store	PA and diet, anthropometric tracking	General pop.	Content Analysis
53	Breton (2011), USA	The aim of this study was to review and summarize the content of available weight control apps.	Mobile apps, available from iTunes App Store	Weight control	General pop.	Content Analysis
89	Conroy (2014), USA	The aim of this study was to characterize the behavior change techniques represented in online descriptions of top-ranked apps for physical activity.	Mobile apps, available from iTunes App Store and Google Play	PA	General pop.	Content Analysis
96	Cowan (2013), USA	To quantify the presence of health behavior theory constructs in iPhone apps targeting physical activity.	Mobile apps, available from iTunes App Store	PA	General pop.	Content Analysis

Study ID	Author (Year), Country	Objectives of the paper	Technologies used	Relevant Outcomes	Target population	Methodology/ Study design
* 107<br><br>[Also cited in Section 1b]	Derbyshire (2013), UK	In this paper, we set out to evaluate whether smartphone technology use is effective in terms of significantly improving aspects of women's health, including reductions in body weight and improved health awareness for chronic diseases, including diabetes, heart disease, breast cancer, and osteoporosis. The uses of health apps to improve diet quality, support mental, and maternal health are also reviewed.	Mobile apps, available from iTunes App Store	Various health indicators, including those for weight-related outcomes (BMI, weight) PA, diet	Women	Systematic Review with qualitative synthesis; Content Analysis
111	Direito (2014), New Zealand	The purpose of this study was to investigate the presence of BCTs in physical activity and dietary apps and determine how reliably the taxonomy checklist can be used to identify BCTs in smartphone apps.	Mobile apps, available from iTunes App Store	PA and diet	General pop.	Content Analysis
148	Garcia-Gomez (2014), Spain	The main purpose of this article is to analyse different m-health apps for a broad spectrum of consumers by means of three different experiences. This goal was defined following the strategic documents generated by the main prospective observatories of Information and Communications Technology for health.	Mobile apps, available from iTunes App Store, Google Play, Blackberry, Symbian)	Diet	General pop.	Content Analysis
161	Grimes (2010), USA	We performed a content analysis of the information shared in a locally and culturally focused health application.	Mobile app: *EatWell*	Various health topics, including weight, PA and diet	General pop.	Content Analysis of interview materials
180	Hermawati (2014), UK	This paper reviews reported studies that concern the design and development of mobile phone applications to prevent obesity, and analyses them from a user-centred design perspective.	Mobile apps, available from searches in databases (ANTE, ACM DL, Ei Compendex, IEEE Wiley eBooks Library, IEEE/IET Electronic Library, INSPEC Archive and Zetoc)	PA and diet	General pop.	Content Analysis
258	Lyons (2014), USA	The purpose of this study was to describe the behavior change techniques implemented in commercially available electronic activity monitors.	Activity monitors and mobile apps, available from listings: CNET reviews for wearable technology; "Health and Fitness" category (Apple Store); "activity monitors" on Amazon	PA	General pop.	Content Analysis

Study ID	Author (Year), Country	Objectives of the paper	Technologies used	Relevant Outcomes	Target population	Methodology/ Study design
280	Middelweerd (2014), Netherlands	The present study aims to review apps developed for iOS and Android that promote physical activity among adults through individually tailored feedback and advice.	Mobile apps, available from iTunes App Store and Google Play	PA	Adults	Content Analysis
281	Millington (2014), UK	This paper presents an in-depth study of prominent health and fitness-themed smartphone apps.	Mobile apps, available from listings in the Canadian Broadcasting Corporation (CBC) report entitled "Top 10 apps for health, fitness and nutrition"	PA	General pop.	Content Analysis
311	Pagoto (2013), USA	The primary aims of the study were to determine the degree to which commercial weight- loss mobile apps include the behavioural strategies included in evidence-based weight-loss interventions, and to identify features that enhance behavioural strategies via technology.	Mobile apps, available from iTunes App Store and Google Play	Weight-related	General pop.	Content Analysis
* 343<br><br>[Also cited in Section 2b]	Rusin (2013), Slovakia	The objective of this paper was to summarise existing approaches to self-management of food intake recording and to analyse the functionalities and input methods. […] We searched for relevant apps and classified them into three types: research apps developed for research purposes, free apps and "freemium" apps available in two versions: free ("light" version) and paid (with additional functionality). In the paper, we discuss self-management in general and the drawbacks of paper-based food diaries, and we present our findings.	Mobile apps, available from iTunes App Store and Google Play	Diet	General pop.	Systematic Review with qualitative synthesis; Content Analysis
350	Schoffman (2013), USA	The present analysis examines the content of mobile apps for the prevention and treatment of paediatric obesity (Children/teens < 18 years) through weight loss, HE, and PA to determine if strategies and behavioural targets of the 2007 Expert Committee for Pediatric Obesity Prevention (ECPOP) are promoted	Mobile apps, available from iTunes App Store and Google Search	Weight-related and PA and diet	Children and adolescents	Content Analysis
364	Silva (2014), USA	This paper reports on the results of a heuristic evaluation of Nike+ and RunKeeper, two of the most popular health and fitness mobile apps found in both Google Play and the iTunes stores for Android and iOS platforms respectively.	Mobile apps, available from iTunes App Store and Google Play (*Nike+* and *RunKeeper*)	PA	General pop.	Content Analysis

Study ID	Author (Year), Country	Objectives of the paper	Technologies used	Relevant Outcomes	Target population	Methodology/ Study design
423	Watkins (2014), USA	Despite the potential of tablets to provide new methods for increasing older adults' FV intake, the usability issues facing older adults using tablet applications (apps) dealing with FV remain unclear. To address this gap in the literature, this study addressed the following research question: What are common usability problems for older adult users of existing iPad apps identified though expert review?	Mobile/iPad apps, available from iTunes App Store and identified though expert review	Diet	Older adults	Mixed
424	Wearing (2014), USA	The present study examines the content of children's exercise and nutrition smartphone apps.	Mobile apps, available from iTunes App Store	PA and diet	Children	Content Analysis
428	West (2012), USA	The purpose of this study was to conduct a content analysis of app developers' written descriptions of health and fitness apps, which interface with the iPhone. Additionally, this study sought to analyse the written description for each health and fitness app to appraise its potential for influencing behavior change.	Mobile apps, available from iTunes App Store	PA	General pop.	Content Analysis
429	West (2013), USA	The purpose of this study was to examine actual content of diet-related apps available in Apple's App Store. Specifically, this study aimed to explore the extent to which constructs of health behavior theory are present in diet-related apps. We hypothesized that constructs of health behavior theory were not widely implemented in the design of most diet-related apps.	Mobile apps, available from iTunes App Store	Diet	General pop.	Content Analysis
Web 2.0 technologies (16 studies)						
* 3<br><br>[Also cited in Section 1b]	Adams (2007), Netherlands	In this article, I will discuss the emergent findings from a new project that looks at blogging interfaces as potential tools for disease prevention and health promotion. The purpose of this paper has been to take a first look at health- related blog development in the Netherlands and to raise questions about new trends in health information management that are emerging with the further development of point-and-click tools.	Blogs (*Valtaf.nl*)	PA and diet	General pop.	Case report

Study ID	Author (Year), Country	Objectives of the paper	Technologies used	Relevant Outcomes	Target population	Methodology/ Study design
19	Appleton (2014), Australia	To explore parents' use of asynchronous online discussion boards for child health information seeking, advice and social support.	Discussion boards	Weight loss, social support	Adults, parents	Content Analysis
33	Ballantine (2011), New Zealand	This study aims to explore how users of a commercial social networking site who are focussed on weight loss give and/or receive social support to/from other users. The authors use quantitative data from 145 members of the Weight Watchers Facebook page to explore how social support is both given and received, and the communication style by which this is achieved.	Social networking site (*Weight Watchers* on Facebook)	Weight loss, social support, communicati on style	General pop.	Quantitative; Content Analysis
48	Boepple (2014), USA	"Healthy" living blogs are a recent addition to internet media that offer advice on improving physical and mental health. Often, these sites include information on eating, exercise, and self-image. This study was a content analysis designed to evaluate the information included on these sites.	"Healthy living" blogs	PA and diet	General pop.	Content Analysis
170	Harris (2014), USA	Little is known about the use of social media as a tool for health communication. We used a mixed-methods design to examine communication about childhood obesity on Twitter.	Twitter	Obesity related topics	General pop.	Content Analysis
257	Lynch (2010), Canada	This paper describes the virtual socialization, behaviours, and attitudes being promoted in one community of food bloggers.	Food blogs	Healthy eating (diet)	General pop.	Content analysis
283	Modave (2014), USA	We systematically identified and evaluated the quality and comprehensiveness of online information related to weight loss that users were likely to access. We evaluated the content quality, accessibility of the information, and author credentials for Web sites in 2012 that were identified from weight loss specific queries that we generated. We scored the content with respect to available evidence-based guidelines for weight loss.	Websites and blogs	Weight loss	General pop.	Content Analysis

Study ID	Author (Year), Country	Objectives of the paper	Technologies used	Relevant Outcomes	Target population	Methodology/ Study design
294	Nakhasi (2014), USA	The aim was to evaluate the functionality, features, and usability of existing online social networks which seek to increase physical activity and fitness among users by connecting them to physical activity partners, not just online, but also face-to-face.	Websites with social media components, available from listings Google; TechCrunch; Y Combinator	PA	General pop.	Content Analysis
309	Pagoto (2014), USA	The present mixed-methods study aims to describe adults who use the online social network Twitter to discuss a current weight loss attempt, how their Twitter friends compare with their Facebook friends, their family and in-person friends in terms of positive and negative social influence, whether positive and negative social influence from these relationships predicts weight lost in the most recent attempt, and the benefits and drawbacks of discussing weight loss in an online social network.	Twitter and Facebook	Positive and negative social influence; benefits and drawbacks related to weight loss, PA and diet	Adults	Mixed
317	Paul (2014), USA	We describe a topic modelling framework for discovering health topics in Twitter, a social media website. This is an exploratory approach with the goal of understanding what health topics are commonly discussed in social media. This paper describes in detail a statistical topic model created for this purpose, the Ailment Topic Aspect Model (ATAM), as well as our system for filtering general Twitter data based on health keywords and supervised classification.	Twitter	Various health topics, including weight, PA and diet	General pop.	Quantitative
344	Sanford (2010), USA	This paper explores the use of weight loss blogs by the morbidly obese and argues that blogging provides invaluable computer-mediated social support for them in four ways: (a) it allows for empathy; (b) it ensures accountability to others; (c) it provides venting and advice seeking; and (d) it shares validation of the weight loss experience.	Blogs (*Fatfighterblogs.com*)	Social support for weight loss	Morbidly obese bloggers	Qualitative Survey and Content Analysis

Study ID	Author (Year), Country	Objectives of the paper	Technologies used	Relevant Outcomes	Target population	Methodology/ Study design
345	Savolainen (2010), Finland	This paper investigates the strategies that people employ to solicit and provide informational and emotional support in the blogosphere. The studies of online support groups discussed above indicate that researchers have converged in four different types of support that people may solicit and provide: informational, esteem, emotional, and social network support.	"Weight loss and dieting" blogs available from *Bloglista.fi*	Dietary issues discussed in blogs; strategies for soliciting support; strategies for providing support.	General pop.	Content Analysis
367	Simunaniemi (2011), Sweden	This study aims to describe the nature of non-commercial Swedish blogs with fruits and vegetables (F&V)-related content and to identify different blogger types.	Fruit and vegetable-related blogs available from Google searches	Fruit and vegetable consumption and other dietary issues discussed	General pop.	Content analysis
398	Toscos (2011), USA	This article describes the top barriers that emerged from a qualitative analysis of message board traffic from a three-month healthy lifestyle intervention that promoted physical activity and healthy eating.	Online community message boards of a healthy lifestyle intervention (*GetFit!*)	Barriers to PA and healthy eating	General pop.	Content Analysis
415	Vandelanotte (2014), Australia	The purpose of this study was to evaluate whether freely accessible websites delivering physical activity interventions use evidence-based behavior change techniques and provide social media applications.	Websites with social media components, available from Google, Bing, and Yahoo searches, and Google Trends	PA	General pop.	Content Analysis

Reference list of included studies (in alphabetical order)

1. Acharya SDE, O. U.; Sereika, S. M.; Styn, M. A.; Burke, L. E. Using a personal digital assistant for self-monitoring influences diet quality in comparison to a standard paper record among overweight/obese adults. J Am Diet Assoc. 2011;111(4):583-8. PMID: Cn-00778386. doi:10.1016/j.jada.2011.01.009.
2. Adams MAS, J. F.; Norman, G. J.; Hovell, M. F.; Hekler, E. B.; Perata, E. An adaptive physical activity intervention for overweight adults: A randomized controlled trial. PLoS ONE. 2013;8(12). PMID: Cn-00979395. doi:10.1371/journal.pone.0082901.
3. Adams SA. Using blogging tools to help individuals record their experiences: an exploration and review of two commercial web applications in the Netherlands. Studies in Health Technology and Informatics. 2007;130:193-203. PMID: 17917193.
4. Aguiar BS, J.; Rocha, T.; Carneiro, S.; Sousa, I.; Ieee,, editor. Monitoring Physical Activity and Energy Expenditure with Smartphones. 2014 Ieee-Embs International Conference on Biomedical and Health Informatics (Bhi); 2014.
5. Aguilar-Martinez AS-S, J. M.; Mancebo-Moreno, G.; Medina, F. X.; Carreras-Collado, R.; Saigi-Rubio, F. Use of mobile phones as a tool for weight loss: a systematic review. Journal of Telemedicine and Telecare. 2014 Sep;20(6):339-49. doi:10.1177/1357633x14537777.
6. Ahmad ZK, N.; Kerr, D. A.; Boushey, C. J.; Delp, E. J., editor. A Mobile Phone User Interface for Image-Based Dietary Assessment. 2014 Conference on Mobile Devices and Multimedia - Enabling Technologies, Algorithms, and Applications; 2014 February 18, 2014; San Francisco, CA, USA.
7. Ahtinen AI, Minna; Ramiah, Shruti; Blom, Jan. Advise, acknowledge, grow and engage: Design principles for a mobile wellness application to support physical activity. . International Journal of Mobile and Blended Learning. 2013;5(4):20-55.
8. Akker H, Jones VM, Hermens HJ. Tailoring real-time physical activity coaching systems: A literature survey and model. User Modeling and User-Adapted Interaction. 2014;24:351-92. PMID: 2014-26747-001. doi:10.1007/s11257-014-9146-y.
9. Allen JKS, J.; Dennison Himmelfarb, C. R.; Stewart, K. J.; Hauck, S. Randomized controlled pilot study testing use of smartphone technology for obesity treatment. Journal of Obesity. 2013. PMID: CN-00977935. doi:10.1155/2013/151597.
10. Allen JKS, Janna; Patel, Angel. Technology-Assisted Weight Management Interventions: Systematic Review of Clinical Trials. Telemedicine and e-Health. 2014 2014/12/01;20(12):1103-20. doi:10.1089/tmj.2014.0030.
11. Almomani AA, Eman; Alkoffash, Mahmud ; AL-Alawneh, Ahmad A Framework of Childhood Obesity Intervention Using Persuasive Mobile Game Technology. Australian Journal of Basic and Applied Sciences. 2014;8(7):499-509.
12. Altini MV, R.; Van Hoof, C.; van Dort, M.; Amft, O.; Ieee, editor. Self-Calibration of Walking Speed Estimations Using Smartphone Sensors. 2014 IEEE International Conference on Pervasive Computing and Communications Workshops (Percom Workshops); 2014 March 24-28, 2014; Budapest, Hungary.
13. Anderson I, Maitland J, Sherwood S, Barkhuus L, Chalmers M, Hall M, et al. Shakra: Tracking and Sharing Daily Activity Levels with Unaugmented Mobile Phones. Mobile Netw Appl. 2007 2007/06/01/;12(2-3):185-99. doi:10.1007/s11036-007-0011-7.
14. Anguita DG, A.; Oneto, L.; Parra, X.; Reyes-Ortiz, J. L., editor. Training Computationally Efficient Smartphone-Based Human Activity Recognition Models. 23rd International Conference on Artificial Neural Networks; 2013 September 10-13, 2013; Sofia, Bulgaria.
15. Antos SAA, M. V.; Kording, K. P. Hand, belt, pocket or bag: Practical activity tracking with mobile phones. Journal of Neuroscience Methods. 2014 Jul;231:22-30. doi:10.1016/j.jneumeth.2013.09.015.
16. Antwi FAF, N.; Garcon, M. C.; Lopez, L.; Rubiano, R.; Slyer, J. T. Effectiveness of web-based programs on the reduction of childhood obesity in school-aged children: a systematic review. JBI Database of Systematic Reviews and Implementation Reports. 2013;11(6):1-44. PMID: DARE-12013057780. doi:10.11124/jbisrir-2013-459.
17. Anwar MH, Edward;Skujins, John;Huynh, Kitty;Doss, Cristopher, editor. Kalico: A Smartphone Application for Health-smart Menu Selection Within a Budget. International Conference Smart Health, ICSH 2013; 2013 August 3-4, 2013; Beijing, China. Berlin, Heidelberg: Springer-Verlag.
18. Appel HBH, B.; Cole, A.; James, R.; Ai, A. L. Starting the conversation-a childhood obesity knowledge project using an app. British Journal of Medicine and Medical Research. 2014;4(7):1526-38. PMID: 20143021481.
19. Appleton JF, Cathrine; Brown, Nicola. Friend or foe? An exploratory study of Australian parents' use of asynchronous discussion boards in childhood obesity. Collegian. 2014;21(2):151-8.
20. Arab LE, D.; Kim, D. H.; Burke, J.; Goldman, J. Feasibility testing of an automated image-capture method

to aid dietary recall. Eur J Clin Nutr. 2011 Oct;65(10):1156-62. PMID: 21587282. doi:10.1038/ejcn.2011.75.
21. Arab LW, A. Automated camera-phone experience with the frequency of imaging necessary to capture diet. J Am Diet Assoc. 2010 Aug;110(8):1238-41. PMID: 20656101. doi:10.1016/j.jada.2010.05.010.
22. Arif MB, M.; Kattan, A.; Ahamed, S. I. Better Physical Activity Classification using Smartphone Acceleration Sensor. J Med Syst. 2014 Sep;38(9):10. doi:10.1007/s10916-014-0095-0.
23. Årsand EO, O. A.; Varmedal, R.; Mortensen, W.; Hartvigsen, G., editor. A system for monitoring physical activity data among people with type 2 diabetes. 21st International Congress of the European-Federation-for-Medical-Informatic (MIE2008); 2008 May 27, 2008; Gothenburg, Sweden: IOS Press.
24. Arteaga SMK, S., editor. Designing an Application to Motivate Teenagers' Physical Activity. 2012 IEEE International Conference on Communications (ICC); 2012 June 10-15, 2012; Ottawa, Canada: Ieee.
25. Ashrafian HT, Tania; Harling, Leanne; Kerr, Karen; Athanasiou, Thanos; Darzi, Ara. Social Networking Strategies That Aim To Reduce Obesity Have Achieved Significant Although Modest Results. Health Affairs. 2014;33(9):1641-7. PMID: 2012728909. Language: English. Entry Date: 20140926. Revision Date: 20141003. Publication Type: journal article. doi:10.1377/hlthaff.2014.0370.
26. Atienza AAK, A. C.; Oliveira, B. M.; Ahn, D. K.; Gardner, C. D. Using hand-held computer technologies to improve dietary intake. American Journal of Preventive Medicine. 2008;34(6):514-8. PMID: Cn-00639572. doi:10.1016/j.amepre.2008.01.034.
27. Atienza AAO, B.; Fogg, B. J.; King, A. C. Using electronic diaries to examine physical activity and other health behaviors of adults age 50+. Journal of Aging and Physical Activity. 2006;14(2):192-202. PMID: 2009345948. Language: English. Entry Date: 20070112. Revision Date: 20091218. Publication Type: journal article.
28. Ayubi SUP, B., editor. PersonA: Persuasive social network for physical Activity. 34th Annual International Conference of the IEEE Engineering in Medicine and Biology Society (EMBS); 2012 August 28-September 1, 2012; San Diego, CA, USA.
29. Azar KMJ, Lesser LI, Laing BY, Stephens J, Aurora MS, Burke LE, et al. Mobile applications for weight management: Theory-based content analysis. . American Journal of Preventive Medicine. 2013;45(5):583-9. doi:10.1016/j.amepre.2013.07.005.
30. Ba SW, Lei. Digital health communities: The effect of their motivation mechanisms. Decision Support Systems. 2013;55(4):941-7. doi:10.1016/j.dss.2013.01.003.
31. Bacigalupo R, Cudd P, Littlewood C, Bissell P, Hawley MS, Buckley Woods H. Interventions employing mobile technology for overweight and obesity: an early systematic review of randomized controlled trials. Obesity Reviews. 2013;14(4):279-91. doi:10.1111/obr.12006.
32. Baghaei NK, Stephen; Freyne, Jill; Brindal, Emily; Berkovsky, Shlomo; Smith, Greg. Engaging families in lifestyle changes through social networking. International Journal of Human Computer Interaction. 2011;27(10):971-90. doi:10.1080/10447318.2011.555315.
33. Ballantine PWS, Rachel J. Help me, I'm fat! Social support in online weight loss networks. . Journal of Consumer Behaviour. 2011;10(6):332-7. doi:10.1002/cb.374.
34. Bartley JF, Jonathon; Pendse, Prachi; Xin, Da; Brown, Garrett; Hagseth, Paul; Agrawal, Ashish; Goldberg, Daniel W.; Hammond, Tracy, editor. World of Workout: A Contextual Mobile RPG to Encourage Long Term Fitness. 21st ACM SIGSPATIAL International Conference on Advances in Geographic Information Systems (ACM SIGSPATIAL GIS 2013); 2013 November 5-8, 2013 Orlando, FL, USA. New York, NY, USA: ACM.
35. Batch BCT, C.; Bagwell, J.; Corsino, L.; Intille, S.; Lin, P. H.; Lazenka, T.; Bennett, G.; Bosworth, H. B.; Voils, C.; Grambow, S.; Sutton, A.; Bordogna, R.; Pangborn, M.; Schwager, J.; Pilewski, K.; Caccia, C.; Burroughs, J.; Svetkey, L. P. Weight loss intervention for young adults using mobile technology: Design and rationale of a randomized controlled trial - Cell Phone Intervention for You (CITY). Contemp Clin Trials. 2014;37(2):333-41. PMID: CN-00985243. doi:10.1016/j.cct.2014.01.003.
36. Bauer S, de Niet J, Timman R, Kordy H. Enhancement of care through self-monitoring and tailored feedback via text messaging and their use in the treatment of childhood overweight. Patient Education and Counseling. 2010 2010/06//;79(3):315-9. doi:10.1016/j.pec.2010.03.014.
37. Beasley J, Riley WT, Jean-Mary J. Accuracy of a PDA-based dietary assessment program. Nutrition. 2005 2005/06//;21(6):672-7. doi:10.1016/j.nut.2004.11.006.
38. Beasley JMR, W. T.; Davis, A.; Singh, J. Evaluation of a PDA-based dietary assessment and intervention program: a randomized controlled trial. Journal of the American College of Nutrition. 2008;27(2):280-6. PMID: Cn-00650365.
39. Bech-Larsen TG, A. Promoting healthy eating to children: a text message (SMS) feedback approach. International Journal of Consumer Studies. 2013;37(3):250-6. PMID: 20133292642.
40. Bennett GGS, D. M.; Stoute, C.; Lanpher, M.; Lane, I.; Askew, S.; Foley, P. B.; Baskin, M. L. Electronic health (eHealth) interventions for weight management among racial/ethnic minority adults: a systematic

review. Obesity Reviews. 2014;15:146-58. doi:10.1111/obr.12218.
41. Bergman RJS, J. W.; Hall, M. E.; Bergman, S. M. Is there a valid app for that? Validity of a free pedometer iPhone application. Journal of Physical Activity and Health. 2012 Jul;9(5):670-6. PMID: 21946023.
42. Bert FG, M.; Gualano, M. R.; Siliquini, R. Smartphones and Health Promotion: A Review of the Evidence. J Med Syst. 2014 Jan;38(1):11. doi:10.1007/s10916-013-9995-7.
43. Bexelius CL, M.; Sandin, S.; Trolle Lagerros, Y.; Forsum, E.; Litton, J. E. Measures of physical activity using cell phones: validation using criterion methods. J Med Internet Res. 2010;12(1):e2. PMID: 20118036. doi:10.2196/jmir.1298.
44. Bexelius CS, S.; Trolle Lagerros, Y.; Litton, J. E.; Lof, M. Estimation of physical activity levels using cell phone questionnaires: a comparison with accelerometry for evaluation of between-subject and within-subject variations. J Med Internet Res. 2011;13(3):e70. PMID: 21946080. doi:10.2196/jmir.1686.
45. Bieber GV, J.; Urban, B., editor. Activity Recognition for Everyday Life on Mobile Phones. 5th International Conference on Universal Access in Human-Computer Interaction held at the HCI International 2009; 2009 July 19-24, 2009; San Diego, CA, USA: Springer-Verlag Berlin.
46. Bielik P, Tomlein M, Krátky P, Mitrík Š, Barla M, Bieliková M, editors. Move2Play: An Innovative Approach to Encouraging People to Be More Physically Active. 2nd ACM International Health Informatics Symposium, IHI 2012; 2012 January 28-30, 2012; Miami, FL, USA. New York, NY, USA: ACM.
47. Blackman KCZ, J.; Berrey, L. M.; Alexander, R.; Fanning, J.; Hill, J. L.; Estabrooks, P. A. Assessing the internal and external validity of mobile health physical activity promotion interventions: a systematic literature review using the RE-AIM framework. J Med Internet Res. 2013;15(10):e224. PMID: 24095951. doi:10.2196/jmir.2745.
48. Boepple LT, Joel Kevin. A content analysis of healthy living blogs: Evidence of content thematically consistent with dysfunctional eating attitudes and behaviors. International Journal of Eating Disorders. 2014;47(4):362-7. doi:10.1002/eat.22244.
49. Bonacina SP, Y. A.; Marsilio, S.; Montin, E.; Passarelli, F.; Masseroli, M.; Pinciroli, F. A web-based tool for cooperating behaviors in eating and physical activity control. Annual Review of CyberTherapy and Telemedicine. 2009;7(1):155-9. PMID: 2010307221.
50. Bond DST, J. G.; Raynor, H. A.; Moon, J.; Sieling, J.; Trautvetter, J.; Leblond, T.; Wing, R. R. B-MOBILE - A Smartphone-Based Intervention to Reduce Sedentary Time in Overweight/Obese Individuals: A Within-Subjects Experimental Trial. PLoS ONE. 2014 Jun;9(6):8. doi:10.1371/journal.pone.0100821.
51. Bort-Roig JG, Nicholas; Puig-Ribera, Anna; Contreras, Ruth; Trost, Stewart. Measuring and Influencing Physical Activity with Smartphone Technology: A Systematic Review. Sports Medicine. 2014;44(5):671-86. PMID: 2012562379. Language: English. Entry Date: 20140509. Revision Date: 20140530. Publication Type: journal article. doi:10.1007/s40279-014-0142-5.
52. Boushey CJK, D. A.; Wright, J.; Lutes, K. D.; Ebert, D. S.; Delp, E. J. Use of technology in children's dietary assessment. (Special Issue: Dietary assessment at the ends of life's spectrum). Eur J Clin Nutr. 2009;63(Supplement 1):S50-S7. PMID: 20093101975. doi:10.1038/ejcn.2008.65.
53. Breton ER, Fuemmeler BF, Abroms LC. Weight loss-there is an app for that! But does it adhere to evidence-informed practices? Translational Behavioral Medicine. 2011 December;1(4):523-9. PMID: 2012370595. doi:10.1007/s13142-011-0076-5.
54. Brindal E, Freyne J, Saunders I, Berkovsky S, Smith G, Noakes M. Features predicting weight loss in overweight or obese participants in a web-based intervention: randomized trial. J Med Internet Res. 2012 2012;14(6). doi:10.2196/jmir.2156.
55. Brindal EH, Gilly; Freyne, Jill; Coombe, Mac; Berkovsky, Shlomo; Noakes, Manny. Design and pilot results of a mobile phone weight-loss application for women starting a meal replacement programme. . Journal of Telemedicine and Telecare. 2013;19(3):166-74. doi:10.1177/1357633X13479702.
56. Brown ONOC, Lauren E.; Savaiano, Dennis. Mobile MyPlate: A Pilot Study Using Text Messaging to Provide Nutrition Education and Promote Better Dietary Choices in College Students. Journal of American College Health. 2014;62(5):320-7. PMID: 2012589934. Language: English. Entry Date: 20140606. Publication Type: journal article. doi:10.1080/07448481.2014.899233.
57. Buchholz SWW, J.; Ingram, D.; Fogg, L. Physical Activity Text Messaging Interventions in Adults: A Systematic Review. Worldviews on Evidence-Based Nursing. 2013;10(3):163-73. PMID: DARE-12013051959.
58. Buhi ERT, T. E.; Martinasek, M. P.; Oberne, A. B.; Fuhrmann, H. J.; McDermott, R. J. Mobile phone-based behavioural interventions for health: A systematic review. Health Education Journal. 2013 September;72(5):564-83. PMID: 2013550140. doi:10.1177/0017896912452071.
59. Buis LRH, Lindsey; Turske, Scott A.; Des Jardins, Terrisca R.; Yarandi, Hossein; Bondurant, Patricia. Use of a text message program to raise type 2 diabetes risk awareness and promote health behavior change (part II): Assessment of participants' perceptions on efficacy. . J Med Internet Res. 2013;15(12):85-93.
60. Burke LEC, M. B.; Sereika, S. M.; Elci, O. U.; Styn, M. A.; Acharya, S. D.; Sevick, M. A.; Ewing, L. J.;

Glanz, K. The effect of electronic self-monitoring on weight loss and dietary intake: a randomized behavioral weight loss trial. Obesity (Silver Spring). 2011;19(2):338-44. PMID: CN-00780749. doi:10.1038/oby.2010.208.

61. Burke LES, M. A.; Glanz, K.; Ewing, L. J.; Elci, O. U.; Conroy, M. B.; Sereika, S. M.; Acharya, S. D.; Music, E.; Keating, A. L.; Sevick, M. A. SMART trial: A randomized clinical trial of self-monitoring in behavioral weight management-design and baseline findings. Contemp Clin Trials. 2009;30(6):540-51. PMID: CN-00733355. doi:10.1016/j.cct.2009.07.003.
62. Burke LES, M. A.; Sereika, S. M.; Conroy, M. B.; Ye, L.; Glanz, K.; Sevick, M. A.; Ewing, L. J. Using mHealth technology to enhance self-monitoring for weight loss: a randomized trial. American Journal of Preventive Medicine. 2012;43(1):20-6. PMID: CN-00840310. doi:10.1016/j.amepre.2012.03.016.
63. Buttussi F, Chittaro L. Smarter Phones for Healthier Lifestyles: An Adaptive Fitness Game. IEEE Pervasive Computing. 2010 2010/10//;9(4):51-7. doi:10.1109/MPRV.2010.52.
64. Caperchione CMK, G. S.; Savage, T. N.; Rosenkranz, R. R.; Maeder, A. J.; Vandelanotte, C.; Duncan, M. J.; Van Itallie, A.; Tague, R.; Mummery, W. K. WALK 2.0: Examining the effectiveness of Web 2.0 features to increase physical activity in a 'real world' setting: an ecological trial. BMJ Open. 2014;4(10):7. doi:10.1136/bmjopen-2014-006374.
65. Carter MCB, V. J.; Cade, J. E. Development of 'My Meal Mate' - A smartphone intervention for weight loss. Nutrition Bulletin. 2013;38(1):80-4. PMID: 2011921448. Language: English. Entry Date: 20130215. Revision Date: 20140314. Publication Type: journal article. doi:10.1111/nbu.12016.
66. Carter MCB, V. J.; Nykjaer, C.; Cade, J. E. 'My Meal Mate' (MMM): validation of the diet measures captured on a smartphone application to facilitate weight loss. Br J Nutr. 2013 Feb 14;109(3):539-46. PMID: 22717334. doi:10.1017/S0007114512001353.
67. Carter MCB, V. J.; Nykjaer, C.; Cade, J. E. Adherence to a smartphone application for weight loss compared to website and paper diary: pilot randomized controlled trial. J Med Internet Res. 2013;15(4):e32. PMID: CN-00915767. doi:10.2196/jmir.2283.
68. Carter MCB, V. J.; Cade, J. E. Handheld Electronic Technology for Weight Loss in Overweight/Obese Adults. Current Obesity Reports. 2014 2014/09/01;3(3):307-15. doi:10.1007/s13679-014-0112-0.
69. Casey MH, P. S.; Murphy, A. W.; Glynn, L.; Glynn, F.; Laighin, G. O.; Heaney, D. Patients' experiences of using a smartphone application to increase physical activity: the SMART MOVE qualitative study in primary care. British Journal of General Practice. 2014 Aug;64(625):E500-E8. doi:10.3399/bjgp14X680989.
70. Castelnuovo GM, G. M.; Cuzziol, P.; Cesa, G. L.; Tuzzi, C.; Villa, V.; Liuzzi, A.; Petroni, M. L.; Molinari, E. TECNOB: study design of a randomized controlled trial of a multidisciplinary telecare intervention for obese patients with type-2 diabetes. BMC Public Health. 2010;10:204. PMID: CN-00895728. doi:10.1186/1471-2458-10-204.
71. Castelnuovo GM, G. M.; Cuzziol, P.; Cesa, G. L.; Corti, S.; Tuzzi, C.; Villa, V.; Liuzzi, A.; Petroni, M. L.; Molinari, E. TECNOB study: Ad interim results of a randomized controlled trial of a multidisciplinary telecare intervention for obese patients with type-2 diabetes. Clinical Practice and Epidemiology in Mental Health. 2011;7:44-50. PMID: CN-00896453. doi:10.2174/1745017901107010044.
72. Castelnuovo GM, Gian Mauro; Pietrabissa, Giada; Corti, Stefania; Giusti, Emanuele Maria; Molinari, Enrico; Simpson, Susan. Obesity and outpatient rehabilitation using mobile technologies: The potential mHealth approach. Frontiers in Psychology. 2014;5.
73. Cavallo DNB, Jane D.; Tate, Deborah F.; DeVellis, Robert F.; Zimmer, Catherine; Ammerman, Alice S. The role of companionship, esteem, and informational support in explaining physical activity among young women in an online social network intervention. Journal of Behavioral Medicine. 2013;37:955-66. PMID: 2013-35029-001. doi:10.1007/s10865-013-9534-5.
74. Cavallo DNT, Deborah F.; Ries, Amy V.; Brown, Jane D.; DeVellis, Robert F.; Ammerman, Alice S. A social media-based physical activity intervention: A randomized controlled trial. . American Journal of Preventive Medicine. 2012;43(5):527-32. doi:10.1016/j.amepre.2012.07.019.
75. Cavallo DNT, Deborah F.; Ward, Dianne S.; DeVellis, Robert F.; Thayer, Linden M.; Ammerman, Alice S. Social support for physical activity—role of Facebook with and without structured intervention. Translational Behavioral Medicine. 2014 2014/12/01;4(4):346-54. doi:10.1007/s13142-014-0269-9.
76. Chang TC, V.; Zhang, C.; Woolford, S. J. The role of social media in online weight management: systematic review. J Med Internet Res. 2013;15(11):e262. PMID: 24287455. doi:10.2196/jmir.2852.
77. Chee HPH, A. S.; Barakatun Nisak, M. Y.; Mohd Nasir, M. T. A randomised controlled trial of a facebook-based physical activity intervention for government employees with metabolic syndrome. Malaysian Journal of Nutrition. 2014;20(2):165-81.
78. Chen J-L, Wilkosz ME. Efficacy of technology-based interventions for obesity prevention in adolescents: a systematic review. Adolescent Health, Medicine and Therapeutics. 2014 08/07;5:159-70. PMID: PMC4132224. doi:10.2147/AHMT.S39969.

79. Chen N, Lee YY, Rabb M, Schatz B, editors. Toward Dietary Assessment via Mobile Phone Video Cameras. 2010 American Medical Informatics Association (AMIA) Annual Symposium 2010 November 13-17, 2010; Washington, DC, USA.
80. Cheung PPY, Chow BC, Parfitt G. Using Environmental Stimuli in Physical Activity Intervention for School Teachers: A Pilot Study. Global Journal of Health Education and Promotion. 2008 2008/01/01/;11(1).
81. Cheung PPYC, Shihui; Wong, Emmy Man Yee. Using Mobile Phone Messages in Pedometer-Based Intervention for Working Adults in Hong Kong. Asian Journal of Exercise and Sports Science. 2012;9(2):76-85. PMID: 94819596.
82. Chomutare TX, A. N.; Iyengar, M. S.; Ieee, editor. Social Network Analysis to Delineate Interaction Patterns that Predict Weight Loss Performance. 2014 IEEE 27th International Symposium on Computer-Based Medical Systems (CBMS); 2014 May 27-29, 2014; New York, NY, USA.
83. Choudhury T, Consolvo S, Harrison B, Hightower J, Lamarca A, Legrand L, et al. The Mobile Sensing Platform: An Embedded Activity Recognition System. IEEE Pervasive Computing. 2008 2008/04//;7(2):32-41. doi:10.1109/MPRV.2008.39.
84. Chuah MS, Steve, editor. Fitness Tour: A Mobile Application for Combating Obesity. 12th ACM International Symposium on Mobile Ad Hoc Networking and Computing, MobiHoc 2011; 2011 May 16-19, 2011; Paris, France. New York, NY, USA: ACM.
85. Chung LMYC, Y. W. Y.; Wong, T. K. W. Usability test of an interactive dietary recording. International Electronic Journal of Health Education. 2009;12:123-34. PMID: 2010386895. Language: English. Entry Date: 20091002. Revision Date: 20110513. Publication Type: journal article.
86. Chung-Tse LH, S. J.; Chia-Tai, Chan, editor. Context-Aware Prompting System for Improving Physical Activity. 2014 IEEE International Symposium on Computer, Consumer and Control (IS3C); 2014 June 10-12, 2014; Taichung, Taiwan: Ieee.
87. Cole-Lewis H, Kershaw T. Text messaging as a tool for behavior change in disease prevention and management. Epidemiologic Reviews. 2010 2010/04/01/;32(1):56-69. doi:10.1093/epirev/mxq004.
88. Collins CE, Dewar DL, Schumacher TL, Finn T, Morgan PJ, Lubans DR. 12 Month changes in dietary intake of adolescent girls attending schools in low-income communities following the NEAT Girls cluster randomized controlled trial. Appetite. 2014 2014/02/01/;73:147-55. doi:10.1016/j.appet.2013.11.003.
89. Conroy DE, Yang CH, Maher JP. Behavior change techniques in top-ranked mobile apps for physical activity. American Journal of Preventive Medicine. 2014;46(6):649-52. PMID: 20143180857.
90. Conroy MB, Yang K, Elci OU, Gabriel KP, Styn MA, Wang J, et al. Physical activity self-monitoring and weight loss: 6-month results of the SMART trial. Medicine and Science in Sports and Exercise. 2011 2011/08//;43(8):1568-74. doi:10.1249/MSS.0b013e31820b9395.
91. Consolvo S, Everitt K, Smith I, Landay JA, editors. Design Requirements for Technologies That Encourage Physical Activity. Conference on Human Factors in Computing Systems, CHI 2006; 2006 April 22-27, 2006; Montreal, Canada. New York, NY, USA: ACM.
92. Consolvo S, Klasnja P, McDonald DW, Avrahami D, Froehlich J, LeGrand L, et al., editors. Flowers or a Robot Army?: Encouraging Awareness & Activity with Personal, Mobile Displays. 10th International Conference on Ubiquitous Computing, UbiComp 2008; 2008 September 21-24, 2008; Seoul, South Korea. New York, NY, USA: ACM.
93. Consolvo S, McDonald DW, Toscos T, Chen MY, Froehlich J, Harrison B, et al., editors. Activity Sensing in the Wild: A Field Trial of Ubifit Garden. 10th International Conference on Ubiquitous Computing, UbiComp 2008; 2008 September 21-24, 2008; Seoul, South Korea. New York, NY, USA: ACM.
94. Coons MJD, A.; Buscemi, J.; Duncan, J. M.; Pellegrini, C. A.; Steglitz, J.; Pictor, A.; Spring, B. Technology Interventions to Curb Obesity: A Systematic Review of the Current Literature. Current Cardiovascular Risk Reports. 2012;6(2):120-34. PMID: 2013159053. doi:10.1007/s12170-012-0222-8.
95. Cornejo RH, D.; Favela, J.; Tentori, M.; Ochoa, S., editor. Persuading older adults to socialize and exercise through ambient games. 2012 6th International Conference on Pervasive Computing Technologies for Healthcare (PervasiveHealth); 2012 May 21-24, 2012; San Diego, CA, USA.
96. Cowan LT, Van Wagenen SA, Brown BA, Hedin RJ, Seino-Stephan Y, Hall PC, et al. Apps of steel: Are exercise apps providing consumers with realistic expectations?: A content analysis of exercise apps for presence of behavior change theory. . Health Education and Behavior. 2013;40(2):133-9. doi:10.1177/1090198112452126.
97. Cushing CC, Jensen CD, Steele RG. An Evaluation of a Personal Electronic Device to Enhance Self-Monitoring Adherence in a Pediatric Weight Management Program using a Multiple Baseline Design. J Pediatr Psychol. 2011 2011/04/01/;36(3):301-7. doi:10.1093/jpepsy/jsq074.
98. Cushing CC, Steele RG. A Meta-Analytic Review of eHealth Interventions for Pediatric Health Promoting and Maintaining Behaviors. J Pediatr Psychol. 2010 2010/10/01/;35(9):937-49. doi:10.1093/jpepsy/jsq023.
99. Damkliang KS, J.; Jingjit, J., editor. A Preliminary Design of Recommended Nutrition and Energy

Expenditure Application for Smartphone. 2nd International Conference on Mechanical Design and Power Engineering (ICMDPE 2013) 2014 November 29-30, 2013; Beijing, China. Stafa-Zurich, Switzerland Trans Tech Publications Ltd.
100. Daugherty BLS, T. E.; Ettienne-Gittens, R.; Zhu, F. M.; Bosch, M.; Delp, E. J.; Ebert, D. S.; Kerr, D. A.; Boushey, C. J. Novel technologies for assessing dietary intake: evaluating the usability of a mobile telephone food record among adults and adolescents. J Med Internet Res. 2012;14(2):e58. PMID: 22504018. doi:10.2196/jmir.1967.
101. David PB, J.; Pennell, M. L.; Katz, M. L.; DeGraffinreid, C. R.; Paskett, E. D. A walking intervention for postmenopausal women using mobile phones and Interactive Voice Response. Journal of Telemedicine and Telecare. 2012;18(1):20-5. PMID: CN-00836544. doi:10.1258/jtt.2011.110311.
102. David PP, Michael L.; Foraker, Randi E.; Katz, Mira L.; Buckworth, Janet; Paskett, Electra D. How Are Previous Physical Activity and Self-Efficacy Related to Future Physical Activity and Self-Efficacy? Health Education and Behavior. 2014;41(6):573-6. PMID: 99406346.
103. de Niet JT, R.; Bauer, S.; van den Akker, E.; Buijks, H.; de Klerk, C.; Kordy, H.; Passchier, J. The effect of a short message service maintenance treatment on body mass index and psychological well-being in overweight and obese children: A randomized controlled trial. Pediatric Obesity. 2012;7(3):205-19. doi:10.1111/j.2047-6310.2012.00048.x.
104. de Niet JT, R.; Bauer, S.; Akker, E.; Klerk, C.; Kordy, H.; Passchier, J. Short message service reduces dropout in childhood obesity treatment: a randomized controlled trial. Health Psychology. 2012;31(6):797-805. PMID: Cn-00911730. doi:10.1037/a0027498.
105. Demment MMG, M.L.; Olson, C.M. How an online intervention to prevent excessive gestational weight gain is used and by whom: a randomized controlled process evaluation. J Med Internet Res. 2014;16(8):e194.
106. Dennison L, Morrison L, Conway G, Yardley L. Opportunities and Challenges for Smartphone Applications in Supporting Health Behavior Change: Qualitative Study. J Med Internet Res. 2013 2013/04/18/;15(4). doi:10.2196/jmir.2583.
107. Derbyshire E, Dancey D. Smartphone medical applications for women's health: What is the evidence-base and feedback? International Journal of Telemedicine and Applications. 2013;2013(782074). PMID: 2014109207. doi:10.1155/2013/782074.
108. Dewar DL, Morgan PJ, Plotnikoff RC, Okely AD, Collins CE, Batterham M, et al. The Nutrition and Enjoyable Activity for Teen Girls Study: A Cluster Randomized Controlled Trial. American Journal of Preventive Medicine. 2013 2013/09//;45(3):313-7. doi:10.1016/j.amepre.2013.04.014.
109. Ding DA, S.; Hiremath, S.; Parmanto, B., editor. Physical activity monitoring and sharing platform for manual wheelchair users. 34th Annual International Conference of the IEEE Engineering in Medicine and Biology Society (EMBS); 2012 August 28-September 1, 2012; San Diego, CA, USA.
110. Ding HK, M.; Duncan, M.; Ireland, D.; Noakes, M.; Hooker, C., editor. A mobile phone enabled health promotion program for middle-aged males. 35th Annual International Conference of the IEEE Engineering in Medicine and Biology Society (EMBS); 2013 July 3-7, 2013; Osaka, Japan.
111. Direito A, Dale LP, Shields E, Dobson R, Whittaker R, Maddison R. Do physical activity and dietary smartphone applications incorporate evidence-based behaviour change techniques? BMC Public Health. 2014;14(646). PMID: 20143235454.
112. Doherty SL, CJ; Canally, C. Tracking human activity and well-being in natural environments using wearable sensors and experience sampling. Social Science and Medicine. 2014;106:83-92.
113. Doherty STO, P. A multi-sensor monitoring system of human physiology and daily activities. Telemedicine and e-Health. 2012 Apr;18(3):185-92. PMID: 22480300. doi:10.1089/tmj.2011.0138.
114. Donaire-Gonzalez DdN, A.; Seto, E.; Mendez, M.; Nieuwenhuijsen, M. J.; Jerrett, M. Comparison of physical activity measures using mobile phone-based CalFit and Actigraph. J Med Internet Res. 2013;15(6):e111. PMID: 23896156. doi:10.2196/jmir.2470.
115. Donaldson ELF, S.; Morris, M. A text message based weight management intervention for overweight adults. Journal of Human Nutrition and Dietetics. 2014;27(s2):90-7. PMID: 20143129124.
116. Duncan JMJ, E. A.; Kozak, A. T.; Roehrig, M.; Russell, S. W.; McFadden, H. G.; Demott, A.; Pictor, A.; Hedeker, D.; Spring, B. PDA+: A Personal Digital Assistant for Obesity Treatment - an RCT testing the use of technology to enhance weight loss treatment for veterans. BMC Public Health. 2011;11:223. PMID: CN-00788239. doi:10.1186/1471-2458-11-223.
117. Duncan MJV, C.; Rosenkranz, R. R.; Caperchione, C. M.; Ding, H.; Ellison, M.; George, E. S.; Hooker, C.; Karunanithi, M.; Kolt, G. S.; Maeder, A.; Noakes, M.; Tague, R.; Taylor, P.; Viljoen, P.; Mummery, W. K. Effectiveness of a website and mobile phone based physical activity and nutrition intervention for middle-aged males: trial protocol and baseline findings of the ManUp Study. BMC Public Health. 2012;12:656. PMID: CN-00841973. doi:10.1186/1471-2458-12-656.
118. Duncan MV, Corneel; Kolt, Gregory S.; Rosenkranz, Richard R.; Caperchione, Cristina M.; George, Emma

S.; Ding, Hang; Hooker, Cindy; Karunanithi, Mohan; Maeder, Anthony J.; Noakes, Manny; Tague, Rhys; Taylor, Pennie; Viljoen, Pierre; Mummery, W. Kerry. Effectiveness of a Web- and mobile phone-based intervention to promote physical activity and healthy eating in middle-aged males: Randomized controlled trial of the ManUp study. J Med Internet Res. 2014;16(6):40-60.
119. Dunton GFL, Yue; Intille, Stephen S.; Spruijt-Metz, Donna; Pentz, Maryann. Investigating children's physical activity and sedentary behavior using ecological momentary assessment with mobile phones. . Obesity (Silver Spring). 2011;19(6):1205-12. doi:10.1038/oby.2010.302.
120. Dunton GFL, Yue; Kawabata, Keito; Intille, Stephen. Momentary assessment of adults' physical activity and sedentary behavior: Feasibility and validity. . Frontiers in Psychology. 2012;3:260. doi:10.3389/fpsyg.2012.00260.
121. Edgar SRF, G. D.; Sazonov, E. S., editor. Recognition of household and athletic activities using SmartShoe. 34th Annual International Conference of the IEEE Engineering in Medicine and Biology Society (EMBS); 2012 August 28-September 1, 2012; San Diego, CA, USA.
122. Edwards HMM, Sharon; Zhao, Tingting; Humphries, Lynne. Design requirements for persuasive technologies to motivate physical activity in adolescents: a field study. Behaviour & Information Technology. 2013 2014/09/02;33(9):968-86. doi:10.1080/0144929X.2013.841755.
123. Ehlers DK, Huberty JL. Middle-Aged Women's Preferred Theory-Based Features in Mobile Physical Activity Applications. J Phys Act Health. 2014;11(7):1379-85. PMID: 100359205.
124. Emken BAL, M.; Thatte, G.; Lee, S.; Annavaram, M.; Mitra, U.; Narayanan, S.; Spruijt-Metz, D. Recognition of physical activities in overweight Hispanic youth using KNOWME Networks. Journal of Physical Activity and Health. 2012 Mar;9(3):432-41. PMID: 21934162.
125. Faghanipour SH, E.; Nikpour, S.; Shariatpanahi, S. A. S.; Hosseini, A. F. Mobile phone short message service (SMS) for weight management in Iranian overweight and obese women: A pilot study. International Journal of Telemedicine and Applications. 2013 (785654). PMID: 2013647914. doi:10.1155/2013/785654.
126. Fanning JM, S. P.; McAuley, E. Increasing physical activity with mobile devices: a meta-analysis. J Med Internet Res. 2012;14(6):e161. PMID: 23171838. doi:10.2196/jmir.2171.
127. Fiordelli MD, N.; Schulz, P. J. Mapping mHealth Research: A Decade of Evolution. J Med Internet Res. 2013 May;15(5):14. doi:10.2196/jmir.2430.
128. Fjeldsoe BP, P.; Bauman, A.; Goode, A.; Maher, G.; Eakin, E. 'Get Healthy, Stay Healthy': protocol for evaluation of a lifestyle intervention delivered by text-message following the Get Healthy Information and Coaching ServiceReg. BMC Public Health. 2014;14(112). PMID: 20143069118.
129. Fjeldsoe BS, Marshall AL, Miller YD. Behavior change interventions delivered by mobile telephone short-message service. American Journal of Preventive Medicine. 2009 2009//02/;36(2):165-73. doi:10.1016/j.amepre.2008.09.040.
130. Fjeldsoe BSM, Yvette D.; Marshall, Alison L. Mobilemums: A randomized controlled trial of an SMS-based physical activity intervention. Annals of Behavioral Medicine. 2010;39(2):101-11. doi:10.1007/s12160-010-9170-z.
131. Fjeldsoe BSM, Y. D.; O'Brien, J. L.; Marshall, A. L. Iterative development of MobileMums: a physical activity intervention for women with young children. International Journal of Behavioral Nutrition and Physical Activity. 2012;9:151. PMID: 23256730. doi:10.1186/1479-5868-9-151.
132. Fjeldsoe BSM, Yvette D.; Marshall, Alison L. Social cognitive mediators of the effect of the MobileMums intervention on physical activity. Health Psychology. 2013;32(7):729-38. doi:10.1037/a0027548.
133. Foster CR, J.; Thorogood, M.; Hillsdon, M. Remote and web 2.0 interventions for promoting physical activity. Cochrane Database of Systematic Reviews. 2013 (9). PMID: 2012376553. Language: English. Entry Date: 20131122. Publication Type: journal article.
134. Foster DL, Conor; Kirman, Ben; Lawson, Shaun; James, Gary, editor. Motivating physical activity at work: Using persuasive social media for competitive step counting. 14th MindTrek: Entertainment and Media in the Ubiquitous Era; 2010 October 6-8, 2010 Tampere, Finland: ACM Press.
135. Fowles ERG, Breine. The Feasibility of Personal Digital Assistants (PDAs) to Collect Dietary Intake Data in Low-Income Pregnant Women. Journal of Nutrition Education and Behavior. 2008 01/01/;40(6-):374-7. PMID: EJ816696.
136. Free C, Phillips G, Galli L, Watson L, Felix L, Edwards P, et al. The Effectiveness of Mobile-Health Technology-Based Health Behaviour Change or Disease Management Interventions for Health Care Consumers: A Systematic Review. PLoS Medicine. 2013 2013/01/15/;10(1). doi:10.1371/journal.pmed.1001362.
137. Freyne J, Berkovsky S, Kimani S, Baghaei N, Brindal E, editors. Improving health information access through social networking. 2010 IEEE 23rd International Symposium on Computer-Based Medical Systems (CBMS); 2010 October 10-15, 2010; Perth, WA, Australia.
138. Freyne J, Bhandari D, Berkovsky S, Borlyse L, Campbell C, Chau S, editors. Mobile Mentor: Weight Management Platform. International Conference on Intelligent User Interfaces, IUI 2010; 2010 February 7-

10, 2010; Hong Kong, China. New York, NY, USA: ACM.
139. Freyne JB, Emily; Hendrie, Gilly; Berkovsky, Shlomo; Coombe, Mac, editor. Mobile Applications to Support Dietary Change: Highlighting the Importance of Evaluation Context. CHI EA 2012: ACM SIGCHI Conference on Human Factors in Computing Systems; 2012 May 5-10, 2012; Austin, TX, USA. New York, NY, USA: ACM.
140. Fujiki Y, Kazakos K, Puri C, Buddharaju P, Pavlidis I, Levine J. NEAT-o-Games: Blending Physical Activity and Fun in the Daily Routine. ACM Computers in Entertainment. 2008 2008/07//;6(2):21:1-:2. doi:10.1145/1371216.1371224.
141. Fukuo W, Yoshiuchi K, Ohashi K, Togashi H, Sekine R, Kikuchi H, et al. Development of a Hand-Held Personal Digital Assistant–Based Food Diary with Food Photographs for Japanese Subjects. J Am Diet Assoc. 2009 2009/07//;109(7):1232-6. doi:10.1016/j.jada.2009.04.013.
142. Fukuoka YK, E.; Bonnet, K.; Lindgren, T. Real-time social support through a mobile virtual community to improve healthy behavior in overweight and sedentary adults: a focus group analysis. J Med Internet Res. 2011;13(3):e49. PMID: 21752785. doi:10.2196/jmir.1770.
143. Fukuoka YK, E.; Dracup, K.; Jong, S. S. New insights into compliance with a mobile phone diary and pedometer use in sedentary women. Journal of Physical Activity and Health. 2011 Mar;8(3):398-403. PMID: 21487139.
144. Fukuoka YK, J.; Suarez, L.; Vittinghoff, E.; Haskell, W.; Noorishad, T.; Pham, K. The mPED randomized controlled clinical trial: applying mobile persuasive technologies to increase physical activity in sedentary women protocol. BMC Public Health. 2011;11:933. PMID: CN-00860443. doi:10.1186/1471-2458-11-933.
145. Fukuoka YL, T.; Jong, S. Qualitative exploration of the acceptability of a mobile phone and pedometer-based physical activity program in a diverse sample of sedentary women. Public Health Nurs. 2012 May-Jun;29(3):232-40. PMID: 22512424. doi:10.1111/j.1525-1446.2011.00997.x.
146. Fukuoka YV, E.; Jong, S. S.; Haskell, W. Innovation to motivation—pilot study of a mobile phone intervention to increase physical activity among sedentary women. Preventive Medicine. 2010;51(3/4):287-9. PMID: 2010781459. Language: English. Entry Date: 20110128. Revision Date: 20120727. Publication Type: journal article. doi:10.1016/j.ypmed.2010.06.006.
147. Gao CMK, F. Y.; Tan, J. D., editor. HealthAware: Tackling Obesity with Health Aware Smart Phone Systems. 2009 IEEE International Conference on Robotics and Biomimetics (ROBIO); 2009 December 19-23, 2009; Guilin, China: Ieee.
148. Garcia-Gomez JMdlT-D, Isabel; Vicente, Javier; Robles, Montserrat; Lopez-Coronado, Miguel; Rodrigues, Joel J. Analysis of mobile health applications for a broad spectrum of consumers: a user experience approach. Health Inform J. 2014;20(1):74-84.
149. Gasser R, Brodbeck D, Degen M, Luthiger J, Wyss R, Reichlin S, editors. Persuasiveness of a Mobile Lifestyle Coaching Application Using Social Facilitation. 1st International Conference on Persuasive Technology for Human Well-Being, PERSUASIVE 2006; 2006 May 18-19, 2006; Eindhoven, The Netherlands: Springer Berlin Heidelberg.
150. Gay VL, P. Personalised mobile health and fitness apps: lessons learned from myFitnessCompanion. Studies in Health Technology and Informatics. 2012;177:248-53. PMID: 22942063.
151. Gerber BSS, M. R.; Thompson, A. L.; Sharp, L. K.; Fitzgibbon, M. L. Mobile phone text messaging to promote healthy behaviors and weight loss maintenance: a feasibility study. Health Inform J. 2009 Mar;15(1):17-25. PMID: 19218309. doi:10.1177/1460458208099865.
152. Glanz KM, S.; Moylan, J.; Evensen, D.; Curb, J. D. Improving dietary self-monitoring and adherence with hand-held computers: A pilot study. American Journal of Health Promotion. 2006 Jan-Feb;20(3):165-70. PMID:16422134.
153. Glynn LGH, P. S.; Casey, M.; Glynn, F.; Alvarez-Iglesias, A.; Newell, J.; Ólaighin, G.; Heaney, D.; Murphy, A. W. SMART MOVE - a smartphone-based intervention to promote physical activity in primary care: study protocol for a randomized controlled trial. Trials. 2013;14:157. PMID: Cn-00863670. doi:10.1186/1745-6215-14-157.
154. Glynn LGH, P. S.; Casey, M.; Glynn, F.; Alvarez-Iglesias, A.; Newell, J.; Olaighin, G.; Heaney, D.; O'Donnell, M.; Murphy, A. W. Effectiveness of a smartphone application to promote physical activity in primary care: the SMART MOVE randomised controlled trial. British Journal of General Practice. 2014 Jul;64(624):E384-E91. doi:10.3399/bjgp14X680461.
155. Goh Kim NB, M. S. B. A., editor. Android-based exercise application. 2012 IEEE International Conference on Computer and Information Science (ICCIS); 2012 June 12-14, 2012; Kuala Lumpur, Malaysia.
156. Gorton DD, R.; Maddison, R.; Mhurchu, C. N.; Jull, A. Consumer views on the potential use of mobile phones for the delivery of weight-loss interventions. Journal of Human Nutrition and Dietetics. 2011;24(6):616-9. doi:10.1111/j.1365-277X.2011.01163.x.
157. Graham MLU, Keriann H.; Niederdeppe, Jeff; Gay, Geri K.; Olson, Christine M. The Theory, Development, and Implementation of an e-Intervention to Prevent Excessive Gestational Weight Gain: e-

Moms Roc. Telemedicine and e-Health. 2014 2014/12/01;20(12):1135-42. doi:10.1089/tmj.2013.0354.
158. Greeff CRY, Joe; MacDonald, Bruce; Wünsche, Burkhard C., editor. My Personal Trainer: An Iphone Application for Exercise Monitoring and Analysis. AUIC '13 - Australasian User Interface Conference; 2013 January 29-February 1, 2013; Adelaide, SA, Australia. Darlinghurst, Australia, Australia: Australian Computer Society, Inc.
159. Greene JS, R.; Piniewski, B.; Kil, D.; Hahn, J. S. The impact of an online social network with wireless monitoring devices on physical activity and weight loss. Journal of Primary Care and Community Health. 2013;4(3):189-94. PMID: 20133234648. doi:10.1177/2150131912469546.
160. Grimes A, Bednar M, Bolter JD, Grinter RE, editors. EatWell: sharing nutrition-related memories in a low-income community. ACM Conference on Computer Supported Cooperative Work, CSCW 2008; 2008 November 8—12, 2008; San Diego, CA, USA: ACM.
161. Grimes A, Landry BM, Grinter RE, editors. Characteristics of shared health reflections in a local community. ACM Conference on Computer Supported Cooperative Work, CSCW 2010; 2010 February 6-10, 2010; Savannah, GA, USA: ACM.
162. Groenewegen MS, D.; Deichmann, D.; van Halteren, A., editor. Connecting with Active People Matters: The Influence of an Online Community on Physical Activity Behavior. 4th International Conference on Social Informatics (SocInfo); 2012 December 5-7, 2012; Lausanne, Switzerland: Springer-Verlag Berlin.
163. Guidoux RD, Martine; Fleury, Gérard; Lacomme, Philippe; Lamaudière, Nicolas; Manenq, Pierre-Henri; Paris, Ludivine; Ren, Libo; Rousset, Sylvie. A smartphone-driven methodology for estimating physical activities and energy expenditure in free living conditions. J Biomed Inform. 2014 12//;52(0):271-8. doi:10.1016/j.jbi.2014.07.009.
164. Guiry JJvdV, Pepijn; Nelson, John; Warmerdam, Lisanne; Riper, Heleen. Activity recognition with smartphone support. Medical Engineering & Physics. 2014 6//;36(6):670-5. doi:10.1016/j.medengphy.2014.02.009.
165. Haapala IB, N. C.; Biggs, S.; Surakka, L.; Manninen, P. Weight loss by mobile phone: a 1-year effectiveness study. Public Health Nutr. 2009;12(12):2382-91. PMID: CN-00733304. doi:10.1017/S1368980009005230.
166. Hagberg LAB, H. K.; Bertz, F.; Winkvist, A. Cost-utility analysis of a randomized controlled weight loss trial among lactating overweight/obese women. BMC Public Health. 2014;14(38). PMID: 20143037361.
167. Hales SBD, Charis; Turner-McGrievy, Gabrielle M. Varying social media post types differentially impacts engagement in a behavioral weight loss intervention. Translational Behavioral Medicine. 2014 2014/12/01;4(4):355-62. doi:10.1007/s13142-014-0274-z.
168. Hamm MPC, A.; Shulhan, J.; Milne, A.; Scott, S. D.; Given, L. M.; Hartling, L. Social media use among patients and caregivers: A scoping review. BMJ Open. 2013;3(5). PMID: 2013346526. doi:10.1136/bmjopen-2013-002819.
169. Harries TE, P.; Stride, C.; Rettie, R.; Walton, S., editor. Walking in the Wild - Using an Always-On Smartphone Application to Increase Physical Activity. 14th IFIP TC 13 International Conference; 2013 September 2-6, 2013; Cape Town, South Africa.
170. Harris JKM-R, S.; Tabak, R. G.; Ruhr, L. R.; Maier, R. C. Communication About Childhood Obesity on Twitter. American Journal of Public Health. 2014 Jul;104(7):E62-E9. doi:10.2105/ajph.2013.301860.
171. He QA, Emmanuel, editor. On11: An Activity Recommendation Application to Mitigate Sedentary Lifestyle. 12th International Conference on Mobile Systems, Applications, and Services, MobiSys 2014; 2014 June 16-19, 2014; Bretton Woods, NH, USA. New York, NY, USA: ACM.
172. He Y, Li Y. Physical Activity Recognition Utilizing the Built-In Kinematic Sensors of a Smartphone. International Journal of Distributed Sensor Networks. 2013 2013/04/10/;2013. doi:10.1155/2013/481580.
173. Hearn LM, Margaret; Fletcher, Anna. Online healthy lifestyle support in the perinatal period: what do women want and do they use it? Australian Journal of Primary Health. 2013;19(4):313-8. PMID: 2012409940. Language: English. Entry Date: 20140103. Revision Date: 20140110. Publication Type: journal article. doi:10.1071/py13039.
174. Hearn LM, M; Lester, L. Reaching perinatal women online: the Healthy You, Healthy Baby website and app. Journal of Obesity. 2014;2014:573928.
175. Hebden L, Cook A, van der Ploeg HP, Allman-Farinelli M. Development of smartphone applications for nutrition and physical activity behavior change. JMIR Res Protoc. 2012 2012;1(2). doi:10.2196/resprot.2205.
176. Hebden LB, K.; McGeechan, K.; Denney-Wilson, E.; Harris, M.; Bauman, A.; Allman-Farinelli, M. 'TXT2BFiT' a mobile phone-based healthy lifestyle program for preventing unhealthy weight gain in young adults: study protocol for a randomized controlled trial. Trials. 2013;14:75. PMID: CN-00964815. doi:10.1186/1745-6215-14-75.
177. Hebden LC, A.; Ploeg, H. P.; King, L.; Bauman, A.; Allman-Farinelli, M. A mobile health intervention for weight management among young adults: a pilot randomised controlled trial. Journal of Human Nutrition

and Dietetics. 2014;27(4):322-32. PMID: 97163186.
178. Heinonen RL, R.; Lindfors, P.; Nygård, C. H. Usability and feasibility of mobile phone diaries in an experimental physical exercise study. Telemedicine and e-Health. 2012;18(2):115-9. PMID: Cn-00835390. doi:10.1089/tmj.2011.0087.
179. Helander EK, K.; Korhonen, I.; Wansink, B. Factors Related to Sustained Use of a Free Mobile App for Dietary Self-Monitoring With Photography and Peer Feedback: Retrospective Cohort Study. J Med Internet Res. 2014 Apr;16(4):52-64. doi:10.2196/jmir.3084.
180. Hermawati S, Lawson G. Managing obesity through mobile phone applications: a state-of-the-art review from a user-centred design perspective. Personal and Ubiquitous Computing. 2014 Dec;18(8):2003-23. doi:10.1007/s00779-014-0757-4.
181. Heron KES, Joshua M. Ecological momentary interventions: Incorporating mobile technology into psychosocial and health behaviour treatments. . British Journal of Health Psychology. 2010;15(1):1-39. doi:10.1348/135910709X466063.
182. Herring SJC, J. F.; Bennett, G. G.; Davey, A.; Foster, G. D. Using Technology to Promote Postpartum Weight Loss in Urban, Low-Income Mothers: A Pilot Randomized Controlled Trial. Journal of Nutrition Education and Behavior. 2014 Nov-Dec;46(6):610-5. doi:10.1016/j.jneb.2014.06.002.
183. Hingle MN, M.; Medeiros, M.; Grace, S. Texting for health: the use of participatory methods to develop healthy lifestyle messages for teens. Journal of Nutrition Education and Behavior. 2013 Jan-Feb;45(1):12-9. PMID: 23103255. doi:10.1016/j.jneb.2012.05.001.
184. Hingle MY, D.; Fowler, J.; Kobourov, S.; Schneider, M. L.; Falk, D.; Burd, R. Collection and visualization of dietary behavior and reasons for eating using Twitter. J Med Internet Res. 2013;15(6):e125. PMID: 23796439. doi:10.2196/jmir.2613.
185. Hofmann WA, Marieke; Vohs, Kathleen D.; Baumeister, Roy F. Dieting and the self-control of eating in everyday environments: An experience sampling study. British Journal of Health Psychology. 2014;19(3):523-39. doi:10.1111/bjhp.12053.
186. Huang JSD, L.; Terrones, L.; Schubert, L.; Roberts, W.; Finklestein, J.; Swartz, M. C.; Norman, G. J.; Patrick, K. Fit4Life: A weight loss intervention for children who have survived childhood leukemia. Pediatric Blood and Cancer. 2014;61(5):894-900. PMID: Cn-00981451. doi:10.1002/pbc.24937.
187. Hughes DCA, A.; Denning, T.; Hurvitz, P.; Lester, J.; Beresford, S.; Borriello, G.; Bruemmer, B.; Moudon, A. V.; Duncan, G. E. BALANCE (Bioengineering Approaches for Lifestyle Activity and Nutrition Continuous Engagement): developing new technology for monitoring energy balance in real time. Journal of Diabetes Science and Technology. 2010 Mar;4(2):429-34. PMID: 20307404.
188. Hui-Huang HM-H, Chang; Yen, N. Y., editor. A health management application with QR-Code input and rule inference. 2012 International Conference on Information Security and Intelligence Control (ISIC); 2012 August 14-16, 2012; Yunlin, Taiwan.
189. Hurling RC, M.; Boni, M. D.; Fairley, B. W.; Hurst, T.; Murray, P.; Richardson, A.; Sodhi, J. S. Using internet and mobile phone technology to deliver an automated physical activity program: randomized controlled trial. J Med Internet Res. 2007;9(2):e7. PMID: Cn-00588579. doi:10.2196/jmir.9.2.e7.
190. Hwang KOE, J. M.; Sciamanna, C. N.; Bernstam, E. V.; Thomas, E. J. Structural social support predicts functional social support in an online weight loss programme. Health Expectations. 2014 Jun;17(3):345-52. doi:10.1111/j.1369-7625.2011.00759.x.
191. Illner AKF, H.; Boeing, H.; Huybrechts, I.; Crispim, S. P.; Slimani, N. Review and evaluation of innovative technologies for measuring diet in nutritional epidemiology. Int J Epidemiol. 2012 Aug;41(4):1187-203. PMID: 22933652. doi:10.1093/ije/dys105.
192. Intille SSA, F.; Mota, S.; Kuris, B.; Botana, P.; Haskell, W. L., editor. Design of a wearable physical activity monitoring system using mobile phones and accelerometers. 33rd Annual International Conference of the IEEE Engineering in Medicine and Biology Society (EMBS); 2011 August 30-September 3, 2011; Boston, MA, USA.
193. Järvinen PJr, T. H.; Lähteenmäki, L.; Södergård, C., editor. HyperFit: Hybrid media in personal nutrition and exercise management. 2008 2nd International Conference on Pervasive Computing Technologies for Healthcare (PervasiveHealth) 2008 January 30-February 1, 2008; Tampere, Finland.
194. Jen W-Y. The adoption of mobile weight management services in a virtual community: The perspective of college students. . Telemedicine and e-Health. 2010;16(4):490-7. doi:10.1089/tmj.2009.0126.
195. Jia WY, Y.; Fernstrom, J. D.; Yao, N.; Sclabassi, R. J.; Fernstrom, M. H.; Sun, M. Imaged based estimation of food volume using circular referents in dietary assessment. Journal of Food Engineering. 2012;109(1):76-86. PMID: 20113384298. doi:10.1016/j.jfoodeng.2011.09.031.
196. Johnston CAR, S.; Miller-Kovach, K.; Moreno, J. P.; Foreyt, J. P. A randomized controlled trial of a community-based behavioral counseling program. American Journal of Medicine. 2013;126(12):1143.e19-24. PMID: CN-00962489. doi:10.1016/j.amjmed.2013.04.025.
197. Joo N-SK, Bom-Taeck. Mobile phone short message service messaging for behaviour modification in a

community-based weight control programme in Korea. . Journal of Telemedicine and Telecare. 2007;13(8):416-20. doi:10.1258/135763307783064331.

198. Joo NSP, Y. W.; Park, K. H.; Kim, C. W.; Kim, B. T. Cost-effectiveness of a community-based obesity control programme. Journal of Telemedicine and Telecare. 2010;16(2):63-7. PMID: 20008053. doi:10.1258/jtt.2009.090407.
199. Joseph RPD, N. H.; Benitez, T. J.; Pekmezi, D. W. Internet-based physical activity interventions. American Journal of Lifestyle Medicine. 2014;8(1):42-67. PMID: 20143101247.
200. Kamal NF, S.; McGrenere, J.; Nance, K.; Marsden, G., editor. Helping Me Helping You: Designing to Influence Health Behaviour through Social Connections. 14th IFIP TC 13 International Conference 2013 September 2-6, 2013; Cape Town, South Africa.
201. Kamal NF, Sidney; Fergusson, Michael. Online social networks for health behaviour change: Designing to increase socialization. Computers in Human Behavior. 2014. PMID: 2014-15829-001. doi:10.1016/j.chb.2014.03.068.
202. Kawano YY, K., editor. Real-time Mobile Food Recognition System. 26th IEEE Conference on Computer Vision and Pattern Recognition (CVPR); 2013 June 25-27, 2013; Portand, OR, USA.
203. Kelly DC, B., editor. An investigation into non-invasive physical activity recognition using smartphones. 34th Annual International Conference of the IEEE Engineering in Medicine and Biology Society (EMBS); 2012 August 28-September 1, 2012; San Diego, CA, USA.
204. Kernot JO, T.; Lewis, L. K.; Maher, C. Effectiveness of a facebook-delivered physical activity intervention for post-partum women: a randomized controlled trial protocol. BMC Public Health. 2013;13:518. PMID: Cn-00915965. doi:10.1186/1471-2458-13-518.
205. Kernot JO, T.; Lewis, L. K.; Maher, C. Usability Testing and Piloting of the Mums Step It Up Program - A Team-Based Social Networking Physical Activity Intervention for Women with Young Children. PLoS ONE. 2014 Oct;9(10):8. doi:10.1371/journal.pone.0108842.
206. Kerr DAP, C. M.; Howat, P.; Delp, E. J.; Pickering, M.; Kerr, K. R.; Dhaliwal, S. S.; Pratt, I. S.; Wright, J.; Boushey, C. J. Connecting Health and Technology (CHAT): protocol of a randomized controlled trial to improve nutrition behaviours using mobile devices and tailored text messaging in young adults. BMC Public Health. 2012;12:477. PMID: CN-00864834. doi:10.1186/1471-2458-12-477.
207. Ketabdar HL, M., editor. System and Methodology for Using Mobile Phones in Live Remote Monitoring of Physical Activities. 2010 IEEE International Symposium on Technology and Society (ISTAS); 2010 June 7-9, 2010; Wollongong, NSW, Australia.
208. Keung CL, Alexa; Lu, Shirley; O'Keefe, Megan, editor. BunnyBolt: A Mobile Fitness App for Youth. IDC '13 - Interaction Design and Children; 2013 June 24-27, 2013; New York, NY, USA. New York, NY, USA: ACM.
209. Khalil A, Glal S, editors. StepUp: A step counter mobile application to promote healthy lifestyle. 2009 International Conference on the Current Trends in Information Technology (CTIT); 2009 December 15-16, 2009; Dubai, UAE.
210. Khalil AA, Salam. Harnessing social dynamics through persuasive technology to promote healthier lifestyle. Computers in Human Behavior. 2013;29(6):2674-81. doi:10.1016/j.chb.2013.07.008.
211. Khan AMS, M. H., editor. Promoting a Healthier Life-Style Using Activity-Aware Smart Phones. 2012 IEEE 4th International Conference on Intelligent and Advanced Systems (ICIAS); 2012 June 12-14, 2012; Kuala Lumpur, Malaysia: Ieee.
212. Khanna N, Boushey CJ, Kerr D, Okos M, Ebert DS, Delp EJ, editors. An Overview of the Technology Assisted Dietary Assessment Project at Purdue University. 2010 IEEE International Symposium on Multimedia (ISM); 2010 December 13-15, 2010; Taichung, Taiwan.
213. Kharrazi HV, L., editor. Increasing Physical Activity by Implementing a Behavioral Change Intervention Using Pervasive Personal Health Record System: An Exploratory Study. 6th International Conference on Universal Access in Human-Computer Interaction (UAHCI); 14th International Conference on Human-Computer Interaction (HCI); 2011 July 9-14, 2011; Orlando, FL, USA: Springer-Verlag Berlin.
214. Khaylis A, Yiaslas T, Bergstrom J, Gore-Felton C. A Review of Efficacious Technology-Based Weight-Loss Interventions: Five Key Components. Telemedicine and e-Health. 2010 2010/11//;16(9):931-8. doi:10.1089/tmj.2010.0065.
215. Khokhar BJ, Jessica; Ronksley, Paul E.; Armstrong, Marni J.; Caird, Jeff; Rabi, Doreen. Effectiveness of mobile electronic devices in weight loss among overweight and obese populations: a systematic review and meta-analysis. BMC Obesity. 2014 2014/10/14/;1(1). doi:10.1186/s40608-014-0022-4.
216. Kikunaga S, Tin T, Ishibashi G, Wang D-H, Kira S. The Application of a Handheld Personal Digital Assistant with Camera and Mobile Phone Card (Wellnavi) to the General Population in a Dietary Survey. Journal of Nutritional Science and Vitaminology. 2007 2007;53(2):109-16. doi:10.3177/jnsv.53.109.
217. Kim BHG, K. Text messaging to motivate walking in older African Americans: a randomized controlled trial. American Journal of Preventive Medicine. 2013;44(1):71-5. PMID: CN-00864325.

doi:10.1016/j.amepre.2012.09.050.
218. Kim JYL, Kee Hyuk; Kim, So Hye; Kim, Kyung Hee; Kim, Jeong Hyun; Han, Jong Soo; Bang, Soo Seok; Shin, Jong Hyuk; Kim, Seong Hye; Hwang, Eun Joo; Bae, Woo Kyung. Needs analysis and development of a tailored mobile message program linked with electronic health records for weight reduction. Int J Med Inf. 2013 11//;82(11):1123-32. doi:10.1016/j.ijmedinf.2013.08.004.
219. King ACA, D. K.; Oliveira, B. M.; Atienza, A. A.; Castro, C. M.; Gardner, C. D. Promoting physical activity through hand-held computer technology. American Journal of Preventive Medicine. 2008;34(2):138-42. PMID: CN-00629397. doi:10.1016/j.amepre.2007.09.025.
220. King ACH, E. B.; Grieco, L. A.; Winter, S. J.; Sheats, J. L.; Buman, M. P.; Banerjee, B.; Robinson, T. N.; Cirimele, J. Harnessing different motivational frames via mobile phones to promote daily physical activity and reduce sedentary behavior in aging adults. PLoS ONE. 2013;8(4):e62613. PMID: 23638127. doi:10.1371/journal.pone.0062613.
221. Kirwan MD, M. J.; Vandelanotte, C.; Mummery, W. K. Using smartphone technology to monitor physical activity in the 10,000 Steps program: a matched case-control trial. J Med Internet Res. 2012;14(2):e55. PMID: 22522112. doi:10.2196/jmir.1950.
222. Kirwan MD, Mitch J.; Vandelanotte, Corneel; Mummery, W. Kerry. Design, development, and formative evaluation of a smartphone application for recording and monitoring physical activity levels: The 10,000 steps "iStepLog". Health Education and Behavior. 2013;40(2):140-51. doi:10.1177/1090198112449460.
223. Klasnja PP, W. Healthcare in the pocket: mapping the space of mobile-phone health interventions. J Biomed Inform. 2012 Feb;45(1):184-98. PMID: 21925288. doi:10.1016/j.jbi.2011.08.017.
224. Knight E, Stuckey MI, Petrella RJ. Health Promotion Through Primary Care: Enhancing Self-Management With Activity Prescription and mHealth. The Physician and Sportsmedicine. 2014 Sep;42(3):90-9. doi:10.3810/psm.2014.09.2080.
225. Knight EP, R. J. Prescribing physical activity for healthy aging: longitudinal follow-up and mixed method analysis of a primary care intervention. The Physician and Sportsmedicine. 2014 Nov;42(4):30-8. PMID: 25419886. doi:10.3810/psm.2014.11.2089.
226. Ko LKT-M, Gabrielle M.; Campbell, Marci K. Information processing versus social cognitive mediators of weight loss in a podcast-delivered health intervention. . Health Education and Behavior. 2014;41(2):197-206. doi:10.1177/1090198113504413.
227. Kolt GSR, R. R.; Savage, T. N.; Maeder, A. J.; Vandelanotte, C.; Duncan, M. J.; Caperchione, C. M.; Tague, R.; Hooker, C.; Mummery, W. K. WALK 2.0 - using Web 2.0 applications to promote health-related physical activity: a randomised controlled trial protocol. BMC Public Health. 2013;13:436. PMID: CN-00964044. doi:10.1186/1471-2458-13-436.
228. Kong FYT, J. D. DietCam: Automatic dietary assessment with mobile camera phones. Pervasive and Mobile Computing. 2012 Feb;8(1):147-63. doi:10.1016/j.pmcj.2011.07.003.
229. Kornman KPS, V. A.; Chou, A. C.; Binh, Nguyen; Lee, A.; O'Connor, J.; Steinbeck, K. S.; Hill, A. J.; Kohn, M. R.; Smita, Shah; Baur, L. A. Electronic therapeutic contact for adolescent weight management: the Loozit study. Telemedicine and e-Health. 2010;16(6):678-85. PMID: 20103273703. doi:10.1089/tmj.2009.0180.
230. Kósa IV, István; Pintér, Balázs; Nemes, Márta; Kámánné, Krisztina; Kohut, László, editor. Preclinical tests of an Android Based Dietary Logging Application. EFMI Special Topic Conference 2014 April 27-29, 2014; Budapest, Hungary. Budapest, Hungary; 2014.
231. Kurdi HAA, A.; Al-Muaibed, A.; Alotaibi, B.; Alhaweal, R.; Alotaibi, T., editor. (my)PDA: A Mobile Healthcare Application for Personal Diet Assisting. International Conference on Advanced Computer Science Applications and Technologies (ACSAT); 2012 November 26-28, 2012; Kuala Lumpur, Malaysia: Ieee.
232. Lacson R, Long W, editors. Natural Language Processing of Spoken Diet Records (SDRs). 2006 American Medical Informatics Association (AMIA) Annual Symposium; 2006 November 11-15, 2006; Washington, DC, USA.
233. Laing BYM, Carol M.; Tseng, Chi-Hong; Leng, Mei; Vaisberg, Ekaterina; Mahida, Megha; Bholat, Michelle; Glazier, Eve; Morisky, Donald E.; Bell, Douglas S. Effectiveness of a Smartphone Application for Weight Loss Compared With Usual Care in Overweight Primary Care PatientsA Randomized, Controlled TrialSmartphone Application for Weight Loss in Overweight Primary Care Patients. Annals of Internal Medicine. 2014;161(10_Supplement):S5-S12. doi:10.7326/M13-3005.
234. Lane NDL, M.; Mohammod, M.; Yang, X. C.; Lu, H.; Cardone, G.; Ali, S.; Doryab, A.; Berke, E.; Campbell, A. T.; Choudhury, T. BeWell: Sensing Sleep, Physical Activities and Social Interactions to Promote Wellbeing. Mobile Netw Appl. 2014 Jun;19(3):345-59. doi:10.1007/s11036-013-0484-5.
235. Lau PWL, E. Y.; Wong del, P.; Ransdell, L. A systematic review of information and communication technology-based interventions for promoting physical activity behavior change in children and adolescents. J Med Internet Res. 2011;13(3):e48. PMID: 21749967. doi:10.2196/jmir.1533.

236. Lauritzen JM, A.; Sevillano, J. L.; Civit, A., editor. The Usefulness of Activity Trackers in Elderly with Reduced Mobility: A Case Study. 14th World Congress on Medical and Health Informatics, Medinfo 2013; 2013 August 20-23, 2013; Copenhagen, Denmark.
237. Lee CDC, J.; Schap, T. E.; Kerr, D. A.; Delp, E. J.; Ebert, D. S.; Boushey, C. J. Comparison of known food weights with image-based portion-size automated estimation and adolescents' self-reported portion size. Journal of Diabetes Science and Technology. 2012 Mar;6(2):428-34. PMID: 22538157.
238. Lee CYL, H.; Jeon, K. M.; Hong, Y. M.; Park, S. H. Self-management program for obesity control among middle-aged women in Korea: a pilot study. Jpn J Nurs Sci. 2011 Jun;8(1):66-75. PMID: 21615699. doi:10.1111/j.1742-7924.2010.00160.x.
239. Lee MH, Kim J, Jee SH, Yoo SK. Integrated Solution for Physical Activity Monitoring Based on Mobile Phone and PC. Healthc Inform Res. 2011 2011/03//;17(1):76-86. doi:10.4258/hir.2011.17.1.76.
240. Lee WC, Y. M.; Kim, S.; Ho, S. H.; Choi, I. Evaluation of a mobile phone-based diet game for weight control. Journal of Telemedicine and Telecare. 2010;16(5):270-5. PMID: 20558620. doi:10.1258/jtt.2010.090913.
241. Leijdekkers P, Gay V. Mobile apps for chronic disease management: lessons learned from myFitnessCompanion. Health and Technology. 2013;3:111-8. PMID: 22942063. doi:10.1007/s12553-013-0044-9.
242. Lieffers JRH, R. M. Dietary assessment and self-monitoring with nutrition applications for mobile devices. Canadian Journal of Dietetic Practice and Research. 2012;73(3):e253-60. PMID: 22958633.
243. Liew Kwei PJP, Poh; Law Kok, Meng; Husain, W.; Adnan, M. H. M., editor. A framework of a childhood obesity intervention using persuasive web-mobile technology. Computer & Information Science (ICCIS), 2012 International Conference on; 2012 12-14 June 2012.
244. Lim JZ, Andong; Ko, Jeonggil; Terzis, Andreas; Szanton, Sarah; Gitlin, Laura. A closed-loop approach for improving the wellness of low-income elders at home using game consoles. IEEE Communications Magazine. 2012;50(1):44-51. PMID: 70576356. doi:10.1109/MCOM.2012.6122531.
245. Limam Mansar SJ, S.; Behih, N.; Shahzad, M.; Anggraini, A. Adapting a database of text messages to a mobile-based weight loss program: The case of the middle east. International Journal of Telemedicine and Applications. 2014 (658149). PMID: 2014080925. doi:10.1155/2014/658149.
246. Lin ML, Nicholas D.; Mohammod, Mashfiqui; Yang, Xiaochao; Lu, Hong; Cardone, Giuseppe; Ali, Shahid; Doryab, Afsaneh; Berke, Ethan; Campbell, Andrew T.; Choudhury, Tanzeem, editor. BeWell+: multi-dimensional wellbeing monitoring with community-guided user feedback and energy optimization. Wireless Health 2012, WH '12; 2012 October 23-25, 2012; San Diego, CA, USA. New York, NY, USA: ACM.
247. Lin PHW, YanFang; Levine, E.; Askew, S.; Lin, ShenTing; Chang, CuiQing; Sun, JiaNi; Foley, P.; Wang, HaiJun; Li, Xu; Bennett, G. G. A text messaging-assisted randomized lifestyle weight loss clinical trial among overweight adults in Beijing. (Special Issue: Eat, sleep, exercise.). Obesity (Silver Spring). 2014;22(5):E29-E37. PMID: 20143169275. doi:10.1002/oby.20686.
248. Lin RJZ, X. Leveraging social media for preventive care-A gamification system and insights. Studies in Health Technology and Informatics. 2012;180:838-42. PMID: 22874310.
249. Lin YZJ, J.; de Vries, B.; Timmermans, H., editor. Motivate: Context Aware Mobile Application for Activity Recommendation. 2nd International Joint Conference on Ambient Intelligence; 2011 November 16-18, 2011; Amsterdam, Netherlands: Springer-Verlag Berlin.
250. Lister CW, Joshua H.; Richards, Rickelle; Crookston, Benjamin; Cougar Hall, Parley; Redelfs, Alisha H. Technology for health: A qualitative study on barriers to using the iPad for diet change. Health. 2013;5(4):761-8. PMID: 2012192612. Language: English. Entry Date: 20140411. Revision Date: 20140425. Publication Type: journal article. doi:10.4236/health.2013.54101.
251. Lombard CD, Amanda; Jolley, Damien; Ball, Kylie; Teede, Helena. A low intensity, community based lifestyle programme to prevent weight gain in women with young children: Cluster randomised controlled trial. . BMJ: British Medical Journal. 2010;341(7764).
252. Long JDB, C.; Rogers, T. J.; Littlefield, L. A.; Estep, G.; Shriver, B. J.; Roman-Shriver, C. R.; Culpepper, D.; Kuenzi, G.; Song, H. Effectiveness of cell phones and mypyramidtracker.gov to estimate fruit and vegetable intake. Appl Nurs Res. 2013 Feb;26(1):17-23. PMID: 23182338. doi:10.1016/j.apnr.2012.08.002.
253. Long JDL, Laurel A.; Estep, Gary; Martin, Hope; Rogers, Toby J.; Boswell, Carol; Shriver, Brent J.; Roman-Shriver, Carmen R. Evidence review of technology and dietary assessment. Worldviews on Evidence-Based Nursing. 2010;7(4):191-204. PMID: 2010881408. Language: English. Entry Date: 20110325. Revision Date: 20120309. Publication Type: journal article. doi:10.1111/j.1741-6787.2009.00173.x.
254. Lubans DR, Morgan PJ, Dewar D, Collins CE, Plotnikoff RC, Okely AD, et al. The Nutrition and Enjoyable Activity for Teen Girls (NEAT girls) randomized controlled trial for adolescent girls from

disadvantaged secondary schools: rationale, study protocol, and baseline results. BMC Public Health. 2010 2010/10/28/;10(1):652. PMID: 21029467. doi:10.1186/1471-2458-10-652.
255. Lubans DR, Morgan PJ, Okely AD, Dewar D, Collins CE, Batterham M, et al. Preventing Obesity Among Adolescent Girls: One-Year Outcomes of the Nutrition and Enjoyable Activity for Teen Girls (NEAT Girls) Cluster Randomized Controlled Trial. Archives of Pediatrics and Adolescent Medicine. 2012 2012/09/01/;166(9):821-7. doi:10.1001/archpediatrics.2012.41.
256. Lubans DR, Smith JJ, Skinner G, Morgan PJ. Development and Implementation of a Smartphone Application to Promote Physical Activity and Reduce Screen-time in Adolescent Boys. Frontiers in Public Health. 2014 2014;2. doi:10.3389/fpubh.2014.00042.
257. Lynch M. Healthy habits or damaging diets: an exploratory study of a food blogging community. Ecology of Food and Nutrition. 2010 Jul-Aug;49(4):316-35. PMID: 21888474. doi:10.1080/03670244.2010.491054.
258. Lyons EJ, Lewis ZH, Mayrsohn BG, Rowland JL. Behavior Change Techniques Implemented in Electronic Lifestyle Activity Monitors: A Systematic Content Analysis. J Med Internet Res. 2014 Aug;16(8):15. doi:10.2196/jmir.3469.
259. Lyzwinski LN. A systematic review and meta-analysis of mobile devices and weight loss with an Intervention Content Analysis. Journal of Personalized Medicine. 2014 September;4(3):311-85. PMID: 2014464269. doi:10.3390/jpm4030311.
260. Ma X, Chen G, Xiao J, editors. Analysis of an Online Health Social Network. 1st ACM International Health Informatics Symposium, IHI 2010; 2010 November 11-12, 2010; Arlington, VA, USA. New York, NY, USA: ACM.
261. Maher CAL, Lucy K.; Ferrar, Katia; Marshall, Simon; De Bourdeaudhuij, Ilse; Vandelanotte, Corneel. Are health behavior change interventions that use online social networks effective? A systematic review. . J Med Internet Res. 2014;16(2):238-50. doi:10.2196/jmir.2952.
262. Maitland JS, S.; Barkhuus, L.; Anderson, I.; Hall, M.; Brown, B.; Chalmers, M.; Muller, H., editor. Increasing the Awareness of Daily Activity Levels with Pervasive Computing. Pervasive Health Conference and Workshops, 2006; 2006 November 29-December 1, 2006; Innsbruck, Austria.
263. Mansar SLJ, S.; Shahzad, M.; Anggraini, A.; Behih, N.; AlZeyara, A., editor. A Usability Testing Experiment For A Localized Weight Loss Mobile Application. 4th Conference on ENTERprise Information Systems (CENTERIS) - Aligning Technology, Organizations and People / International Conference on Health and Social Care Information Systems and Technologies (HCIST) 2012 October 3-5, 2012; Algarve, Portugal: Elsevier Science Bv.
264. Marcus A. Health machine. . Information Design Journal. 2011;19(1):69-89. doi:10.1075/idj.19.1.09mar.
265. Marins DRdODJ, M.; Xexeo, G. B.; de A M Chaves, B.; D'Ipolitto, C., editor. SmartRabbit: A Mobile Exergame Using Geolocation. 2011 Brazilian Symposium on Games and Digital Entertainment (SBGAMES); 2011 November 7-9, 2011; Salvador, Brazil.
266. Marshall ALM, Y. D.; Graves, N.; Barnett, A. G.; Fjeldsoe, B. S. Moving MobileMums forward: protocol for a larger randomized controlled trial of an improved physical activity program for women with young children. BMC Public Health. 2013;13(593). PMID: 20133239863.
267. Martin CK, Han H, Coulon SM, Allen HR, Champagne CM, Anton SD. A novel method to remotely measure food intake of free-living individuals in real time: the remote food photography method. Br J Nutr. 2009 2009/02//;101(03):446-56. doi:10.1017/S0007114508027438.
268. Martin CKC, J. B.; Han, H.; Allen, H. R.; Rood, J. C.; Champagne, C. M.; Gunturk, B. K.; Bray, G. A. Validity of the Remote Food Photography Method (RFPM) for estimating energy and nutrient intake in near real-time. Obesity (Silver Spring). 2012 Apr;20(4):891-9. PMID: 22134199. doi:10.1038/oby.2011.344.
269. Martin CKN, T.; Gunturk, B.; Correa, J. B.; Allen, H. R.; Champagne, C. Measuring food intake with digital photography. (Special Issue: Developing technological solutions for dietary assessment in children and young people.). Journal of Human Nutrition and Dietetics. 2014;27(s1):72-81. PMID: 20143057918.
270. Martín HB, Ana M.; Iglesias, Josué; Casar, José R. Activity Logging Using Lightweight Classification Techniques in Mobile Devices. Personal and Ubiquitous Computing. 2013 2013/04//;17(4):675-95. doi:10.1007/s00779-012-0515-4.
271. Mattila EK, I.; Salminen, J. H.; Ahtinen, A.; Koskinen, E.; Sarela, A.; Parkka, J.; Lappalainen, R. Empowering citizens for well-being and chronic disease management with wellness diary. IEEE Trans Inf Technol Biomed. 2010 Mar;14(2):456-63. PMID: 20007055. doi:10.1109/TITB.2009.2037751.
272. Mattila EL, Raimo; Parkka, Juha; Salminen, Jukka; Korhonen, llkka. Use of a mobile phone diary for observing weight management and related behaviours. . Journal of Telemedicine and Telecare. 2010;16(5):260-4. doi:10.1258/jtt.2009.091103.
273. Mattila EP, J.; Hermersdorf, M.; Kaasinen, J.; Vainio, J.; Samposalo, K.; Merilahti, J.; Kolari, J.; Kulju, M.; Lappalainen, R.; Korhonen, I. Mobile diary for wellness management--results on usage and usability in two user studies. IEEE Trans Inf Technol Biomed. 2008 Jul;12(4):501-12. PMID: 18632330.

doi:10.1109/TITB.2007.908237.
274. McClung HL, Sigrist LD, Smith TJ, Karl JP, Rood JC, Young AJ, et al. Monitoring Energy Intake: A Hand-Held Personal Digital Assistant Provides Accuracy Comparable to Written Records. J Am Diet Assoc. 2009 2009/07//;109(7):1241-5. doi:10.1016/j.jada.2009.04.015.
275. McMahon SV, M.; Fleury, J. Mobile computer application for promoting physical activity. J Gerontol Nurs. 2013 Apr;39(4):15-20. PMID: 23463915. doi:10.3928/00989134-20130226-01.
276. Mehta PS, Manoj. Internet and cell phone based physical activity interventions in adults. Archives of Exercise in Health and Disease. 2011;2(2):108-13. PMID: 61167168.
277. Mendi EO, O.; Pekesen, E.; Bayrak, C., editor. Food Intake Monitoring System for Mobile Devices. 5th IEEE International Workshop on Advances in Sensors and Interfaces (IWASI); 2013 June 13-14, 2013; Bary, Italy: Ieee.
278. Mendis KF, T.; Paxton, K.; Hyland, K.; Yelverton, J.; McLean, R.; Canalese, J.; Brown, A.; Steinbeck, K. Large and forgotten in rural Australia: assessment, attitudes and possible approaches to losing weight in young adult males. BMC Public Health. 2014;14(243). PMID: 20143112882.
279. Merchant GW, Nadir; Patrick, Kevin; Fowler, James H.; Norman, Greg J.; Gupta, Anjali; Servetas, Christina; Calfas, Karen; Raste, Ketaki; Pina, Laura; Donohue, Mike; Griswold, William G.; Marshall, Simon. Click "like" to change your behavior: A mixed methods study of college students' exposure to and engagement with Facebook content designed for weight loss. J Med Internet Res. 2014;16(6):182-201.
280. Middelweerd A, Mollee JS, van der Wal CN, Brug J, te Velde SJ. Apps to promote physical activity among adults: a review and content analysis. International Journal of Behavioral Nutrition and Physical Activity. 2014 Aug;11:9. doi:10.1186/s12966-014-0097-9.
281. Millington B. Smartphone Apps and the Mobile Privatization of Health and Fitness. Critical Studies in Media Communication. 2014;31(5):479-93. PMID: 99777296. doi:10.1080/15295036.2014.973429.
282. Miners AH, J.; Felix, L.; Murray, E.; Michie, S.; Edwards, P. An economic evaluation of adaptive e-learning devices to promote weight loss via dietary change for people with obesity. BMC Health Serv Res. 2012;12:190. PMID: 22769737. doi:10.1186/1472-6963-12-190.
283. Modave F, Shokar NK, Penaranda E, Nguyen N. Analysis of the accuracy of weight loss information search engine results on the internet. American journal of public health. 2014;104(10):1971-8.
284. Mojica CMP-M, Deborah; Yin, Zenong; Akopian, David; Esparza, Laura A. Assessing media access and use among Latina adolescents to inform development of a physical activity promotion intervention incorporating text messaging. Health Promotion Practice. 2014;15(4):548-55.
285. Möller AR, L.; Diewald, S.; Scherr, J.; Kranz, M.; Hammerla, N.; Olivier, P.; Plotz, T., editor. GymSkill: A Personal Trainer for Physical Exercises. 20th IEEE International Conference on Pervasive Computing and Communications (PerCom); 2012 March 19-23, 2012; Lugano, Switzerland.
286. Monteiro SMJ, J.; Howat, P.; Burns, S.; Jones, C.; Dhaliwal, S. S.; McManus, A.; Hills, A. P.; Anderson, A. S. The protocol of a randomized controlled trial for playgroup mothers: Reminder on Food, Relaxation, Exercise, and Support for Health (REFRESH) Program. BMC Public Health. 2011;11:648. PMID: CN-00806541. doi:10.1186/1471-2458-11-648.
287. Morak JS, Karin; Goerzer, Evelin; Kastner, Peter; Toplak, Hermann; Ludvik, Bernhard; Schreier, Guenter. A pilot study of mobile phone-based therapy for obese patients. . Journal of Telemedicine and Telecare. 2008;14(3):147-9. doi:10.1258/jtt.2008.003015.
288. Mukhtar HB, D., editor. Using Adaptive Feedback for Promoting Awareness about Physical Activeness in Adults. 2013 IEEE 10th International Conference on Ubiquitous Intelligence and Computing; 10th International Conference on Autonomic and Trusted Computing (UIC/ATC); 2013 December 18-21, 2013; Vietri sul Mare, Italy.
289. Mulas FP, P.; Manca, M.; Boratto, L.; Carta, S., editor. Linking Human-Computer Interaction with the Social Web: A web application to improve motivation in the exercising activity of users. 2013 IEEE 4th International Conference on Cognitive Infocommunications (CogInfoCom); 2013 December 2-5, 2013; Budapest, Hungary.
290. Mulas FP, P.; Manca, M.; Boratto, L.; Carta, S., editor. Using new communication technologies and social media interaction to improve the motivation of users to exercise. 2013 2nd International Conference on Future Generation Communication Technology (FGCT); 2013 Nobember 12-14, 2013; London, UK.
291. Munson SAC, S., editor. Exploring goal-setting, rewards, self-monitoring, and sharing to motivate physical activity. 2012 6th International Conference on Pervasive Computing Technologies for Healthcare (PervasiveHealth); 2012 May 21-24, 2012; San Diego, CA, USA.
292. Munson SAL, D.; Newman, M. W.; Resnick, P., editor. Happier Together: Integrating a Wellness Application into a Social Network Site. 5th International Conference on Persuasive Technology 2010 June 7-10, 2010; Copenhagen, Denmark: Springer-Verlag Berlin.
293. Mutsuddi AUC, K., editor. Text messages for encouraging physical activity Are they effective after the novelty effect wears off? 2012 6th International Conference on Pervasive Computing Technologies for

Healthcare (PervasiveHealth); 2012 May 21-24, 2012; San Diego, CA, USA.
294. Nakhasi A, Shen AX, Passarella RJ, Appel LJ, Anderson CAM. Online social networks that connect users to physical activity partners: A review and descriptive analysis. J Med Internet Res. 2014;16(6):61-74.
295. Napolitano MAH, S.; Bennett, G. G.; Ives, A. K.; Foster, G. D. Using Facebook and text messaging to deliver a weight loss program to college students. Obesity (Silver Spring). 2013;21(1):25-31. PMID: CN-00907651. doi:10.1002/oby.20232.
296. Newton KH, Wiltshire EJ, Elley CR. Pedometers and text messaging to increase physical activity: randomized controlled trial of adolescents with type 1 diabetes. Diabetes Care. 2009 2009/05//;32(5):813-5. doi:10.2337/dc08-1974.
297. Newton RLM, A. M.; Allen, H. R.; Machtmes, R.; Han, H.; Johnson, W. D.; Schuna, J. M.; Broyles, S. T.; Tudor-Locke, C.; Church, T. S. Parent-Targeted Mobile Phone Intervention to Increase Physical Activity in Sedentary Children: Randomized Pilot Trial. J Med Internet Res. 2014 Nov;16(11):15. doi:10.2196/mhealth.3420.
298. Ngo JE, A.; Molag, M.; Roesle, J.; García-Segovia, P.; Serra-Majem, L. A review of the use of information and communication technologies for dietary assessment. Br J Nutr. 2009;101:S102-12. PMID: 2010380356. Language: English. Entry Date: 20091009. Revision Date: 20091218. Publication Type: journal article.
299. Nguyen B, Shrewsbury VA, O'Connor J, Steinbeck KS, Lee A, Hill AJ, et al. Twelve-month outcomes of the loozit randomized controlled trial: a community-based healthy lifestyle program for overweight and obese adolescents. Archives of Pediatrics and Adolescent Medicine. 2012 2012/02//;166(2):170-7. doi:10.1001/archpediatrics.2011.841.
300. Nguyen BS, V. A.; O'Connor, J.; Steinbeck, K. S.; Hill, A. J.; Shah, S.; Kohn, M. R.; Torvaldsen, S.; Baur, L. A. Two-year outcomes of an adjunctive telephone coaching and electronic contact intervention for adolescent weight-loss maintenance: the Loozit randomized controlled trial. International Journal of Obesity. 2013;37(3):468-72. PMID: CN-00969437. doi:10.1038/ijo.2012.74.
301. Ni Mhurchu CW, Robyn; McRobbie, Hayden; Ball, Kylie; Crawford, David; Michie, Jo; Jiang, Yannan; Maddison, Ralph; Waterlander, Wilma; Myers, Katie. Feasibility, acceptability and potential effectiveness of a mobile health (mHealth) weight management programme for New Zealand adults. BMC Obesity. 2014;1(1):10. PMID: doi:10.1186/2052-9538-1-10.
302. Nolan MM, J. R.; Doyle-Baker, P. K. Validity of the Apple iPhone (R)/iPod Touch (R) as an Accelerometer-Based Physical Activity Monitor: A Proof-of-Concept Study. Journal of Physical Activity and Health. 2014 May;11(4):759-69. doi:10.1123/jpah.2011-0336.
303. Nollen NLH, Tresza; Carlson, Susan; Rapoff, Michael; Goggin, Kathy; Mayfield, Carlene; Ellerbeck, Edward. Development and Functionality of a Handheld Computer Program to Improve Fruit and Vegetable Intake among Low-Income Youth. Health Educ Res. 2013 04/01/;28(2):249-64. PMID: EJ999542.
304. Nollen NLM, Matthew S.; Carlson, Susan E.; Rapoff, Michael A.; Goggin, Kathy J.; Ellerbeck, Edward F. Mobile technology for obesity prevention: A randomized pilot study in racial- and ethnic-minority girls. American Journal of Preventive Medicine. 2014;46(4):404-8. doi:10.1016/j.amepre.2013.12.011.
305. Norman GJ, Zabinski MF, Adams MA, Rosenberg DE, Yaroch AL, Atienza AA. A Review of eHealth Interventions for Physical Activity and Dietary Behavior Change. American Journal of Preventive Medicine. 2007 2007/10//;33(4):336-45.e16. doi:10.1016/j.amepre.2007.05.007.
306. Norman GJK, J. K.; Adams, M. A.; Patrick, K.; Marshall, S. J. Fruit and vegetable intake and eating behaviors mediate the effect of a randomized text-message based weight loss program. Preventive Medicine. 2013;56(1):3-7. PMID: Cn-00921144. doi:10.1016/j.ypmed.2012.10.012.
307. O'Malley GC, M.; Burls, A.; Murphy, S.; Murphy, N.; Perry, I. J. A smartphone intervention for adolescent obesity: study protocol for a randomised controlled non-inferiority trial. Trials. 2014 Jan;15:6. doi:10.1186/1745-6215-15-43.
308. O'Reilly GAS-M, D. Current mHealth technologies for physical activity assessment and promotion. American Journal of Preventive Medicine. 2013;45(4):501-7. PMID: Dare-12013056298.
309. Pagoto S, Schneider K, Jojic M, DeBiasse M, Mann D. Evidence-based strategies in weight-loss mobile apps. American Journal of Preventive Medicine. 2013;45(5):576-82. doi:10.1016/j.amepre.2013.04.025.
310. Pagoto SLS, Kristin L.; Oleski, Jessica; Smith, Brian; Bauman, Michael. The adoption and spread of a core-strengthening exercise through an online social network. . Journal of Physical Activity and Health. 2014;11(3):648-53.
311. Pagoto SS, K. L.; Evans, M.; Waring, M. E.; Appelhans, B.; Busch, A. M.; Whited, M. C.; Thind, H.; Ziedonis, M. Tweeting it off: characteristics of adults who tweet about a weight loss attempt. Journal of the American Medical Informatics Association. 2014 Nov;21(6):1032-7. doi:10.1136/amiajnl-2014-002652.
312. Park MJK, H. S.; Kim, K. S. Cellular phone and Internet-based individual intervention on blood pressure and obesity in obese patients with hypertension. Int J Med Inf. 2009;78(10):704-10. PMID: CN-00722995. doi:10.1016/j.ijmedinf.2009.06.004.

313. Park MJK, H. S. Evaluation of mobile phone and Internet intervention on waist circumference and blood pressure in post-menopausal women with abdominal obesity. Int J Med Inf. 2012;81(6):388-94. PMID: CN-00863696. doi:10.1016/j.ijmedinf.2011.12.011.
314. Patrick KM, S. J.; Davila, E. P.; Kolodziejczyk, J. K.; Fowler, J. H.; Calfas, K. J.; Huang, J. S.; Rock, C. L.; Griswold, W. G.; Gupta, A.; Merchant, G.; Norman, G. J.; Raab, F.; Donohue, M. C.; Fogg, B. J.; Robinson, T. N. Design and implementation of a randomized controlled social and mobile weight loss trial for young adults (project SMART). Contemp Clin Trials. 2014 Jan;37(1):10-8. doi:10.1016/j.cct.2013.11.001.
315. Patrick KN, G. J.; Davila, E. P.; Calfas, K. J.; Raab, F.; Gottschalk, M.; Sallis, J. F.; Godbole, S.; Covin, J. R. Outcomes of a 12-month technology-based intervention to promote weight loss in adolescents at risk for type 2 diabetes. Journal of Diabetes Science and Technology. 2013;7(3):759-70. PMID: CN-00959765.
316. Patrick KR, F.; Adams, M. A.; Dillon, L.; Zabinski, M.; Rock, C. L.; Griswold, W. G.; Norman, G. J. A text message-based intervention for weight loss: randomized controlled trial. J Med Internet Res. 2009;11(1):e1. PMID: CN-00680898. doi:10.2196/jmir.1100.
317. Paul MJD, M. Discovering Health Topics in Social Media Using Topic Models. PLoS ONE. 2014 Aug;9(8):11. doi:10.1371/journal.pone.0103408.
318. Pellegrini CAD, J. M.; Moller, A. C.; Buscemi, J.; Sularz, A.; DeMott, A.; Pictor, A.; Pagoto, S.; Siddique, J.; Spring, B. A smartphone-supported weight loss program: design of the ENGAGED randomized controlled trial. BMC Public Health. 2012;12:1041. PMID: CN-00841617. doi:10.1186/1471-2458-12-1041.
319. Pellegrini CAH, Sara A.; Collins, Linda M.; Spring, Bonnie. Optimization of remotely delivered intensive lifestyle treatment for obesity using the Multiphase Optimization Strategy: Opt-IN study protocol. Contemp Clin Trials. 2014 7//;38(2):251-9. doi:10.1016/j.cct.2014.05.007.
320. Perez-Cruzado DC-V, A. I. Improving adherence physical activity with a smartphone application based on adults with intellectual disabilities (APPCOID). BMC Public Health. 2013;13(1173). PMID: 20143022296.
321. Pernek IH, K. A.; Kokol, P. Exercise repetition detection for resistance training based on smartphones. Personal and Ubiquitous Computing. 2013 Apr;17(4):771-82. doi:10.1007/s00779-012-0626-y.
322. Pfaeffli LM, Ralph; Jiang, Yannan; Dalleck, Lance; Löf, Marie. Measuring Physical Activity in a Cardiac Rehabilitation Population Using a Smartphone-Based Questionnaire. J Med Internet Res. 2013 2013/03/22;15(3):e61. doi:10.2196/jmir.2419.
323. Po'e E, ; Heerman, William J.; Mistry, Rishi S.; Barkin, Shari L. Growing Right Onto Wellness (GROW): a family-centered, community-based obesity prevention randomized controlled trial for preschool child-parent pairs. Contemp Clin Trials. 2013;36(2):436-49.
324. Preston KEW, T. A.; O'Sullivan, A. L. Prompting healthy behavior via text messaging in adolescents and young adults. American Journal of Lifestyle Medicine. 2011;5(3):247-52. PMID: 20113192290. doi:10.1177/1559827610392325.
325. Prestwich A, Perugini M, Hurling R. Can implementation intentions and text messages promote brisk walking? A randomized trial. Health Psychology. 2010 2010;29(1):40-9. doi:10.1037/a0016993.
326. Prestwich AP, M.; Hurling, R. Can the effects of implementation intentions on exercise be enhanced using text messages? Psychology and Health. 2009;24(6):677-87. PMID: 20093209083. doi:10.1080/08870440802040715.
327. Ptomey LTH, Stephen D.; Lee, Jaehoon; Sullivan, Debra K.; Rondon, Mary F.; Donnelly, Joseph E. Photo-Assisted Recall Increases Estimates of Energy and Macronutrient Intake in Adults with Intellectual and Developmental Disabilities. Journal of the Academy of Nutrition and Dietetics. 2013;113(12):1704-9. PMID: 2012370410. Language: English. Entry Date: 20140606. Revision Date: 20140627. Publication Type: journal article. doi:10.1016/j.jand.2013.07.029.
328. Puri M, Zhu Z, Yu Q, Divakaran A, Sawhney H, editors. Recognition and volume estimation of food intake using a mobile device. 2009 Workshop on Applications of Computer Vision (WACV); 2009 December 7-8, 2009; Snowbird, UT, USA.
329. Rabin CB, B. Desired features of smartphone applications promoting physical activity. Telemedicine and e-Health. 2011 Dec;17(10):801-3. PMID: 22010977. doi:10.1089/tmj.2011.0055.
330. Rahman MHL, Q.; Pickering, M.; Frater, M.; Kerr, D.; Bouchey, C.; Delp, E., editor. Food Volume Estimation in a Mobile Phone Based Dietary Assessment System. 8th International Conference on Signal Image Technology and Internet Based Systems (SITIS) 2012 November 25-29, 2012; Sorrento, Italy.
331. Rahman MHP, M. R.; Kerr, D.; Boushey, C. J.; Delp, E. J., editor. A New Texture Feature for Improved Food Recognition Accuracy in a Mobile Phone Based Dietary Assessment System. 2012 IEEE International Conference on Multimedia and Expo Workshops (ICMEW); 2012 July 9-13, 2012; Melbourne, VIC, Australia.
332. Read E. Feasibility of the Diabetes and Technology for Increased Activity (DaTA) Study: a pilot intervention in high-risk rural adults. Journal of Physical Activity and Health. 2014 Jan;11(1):118-26.

PMID: 23249608. doi:10.1123/jpah.2011-0381.
333. Recio-Rodriguez JIM-C, C.; Gonzalez-Viejo, N.; Gomez-Arranz, A.; Arietaleanizbeascoa, M. S.; Schmolling-Guinovart, Y.; Maderuelo-Fernandez, J. A.; Perez-Arechaederra, D.; Rodriguez-Sanchez, E.; Gomez-Marcos, M. A.; Garcia-Ortiz, L. Effectiveness of a smartphone application for improving healthy lifestyles, a randomized clinical trial (EVIDENT II): study protocol. BMC Public Health. 2014;14(254). PMID: 20143112910.
334. Richards JT, M.; Hillsdon, M.; Foster, C. Face-to-face versus remote and web 2.0 interventions for promoting physical activity. Cochrane Database of Systematic Reviews. 2013 (9). PMID: 2012376552. Language: English. Entry Date: 20131122. Revision Date: 20140606. Publication Type: journal article.
335. Riley WT, Rivera DE, Atienza AA, Nilsen W, Allison SM, Mermelstein R. Health behavior models in the age of mobile interventions: are our theories up to the task? Translational Behavioral Medicine. 2011 2011/03//;1(1):53-71. doi:10.1007/s13142-011-0021-7.
336. Robbins LBP, K. A.; Vermeesch, A.; Resnicow, K.; You, Z.; An, L.; Wesolek, S. M. "Girls on the Move" intervention protocol for increasing physical activity among low-active underserved urban girls: a group randomized trial. BMC Public Health. 2013;13:474. PMID: CN-00920617. doi:10.1186/1471-2458-13-474.
337. Robinson EH, S.; Daley, A. J.; Jolly, K.; Lycett, D.; Lewis, A.; Aveyard, P. Development and feasibility testing of a smart phone based attentive eating intervention. BMC Public Health. 2013;13(639). PMID: 20133255006.
338. Rodrigues JJPCL, Ivo M. C.; Silva, Bruno M. C.; Torre, Isabel de La. A new mobile ubiquitous computing application to control obesity: SapoFit. . Informatics for Health and Social Care. 2013;38(1):37-53. doi:10.3109/17538157.2012.674586.
339. Rofey DLH, E. E.; Phillips, J.; Vogt, K.; Silk, J. S.; Dahl, R. E. Utilizing Ecological Momentary Assessment in pediatric obesity to quantify behavior, emotion, and sleep. Obesity (Silver Spring). 2010 Jun;18(6):1270-2. PMID: 20019675. doi:10.1038/oby.2009.483.
340. Rollo MEA, S.; Lyons-Wall, P.; Russell, A. Trial of a mobile phone method for recording dietary intake in adults with type 2 diabetes: evaluation and implications for future applications. Journal of Telemedicine and Telecare. 2011;17(6):318-23. PMID: 21844173. doi:10.1258/jtt.2011.100906.
341. Rompotis CJG, J. Robert; Byrne, Susan M. Benefits of habit-based informational interventions: a randomised controlled trial of fruit and vegetable consumption. Australian and New Zealand Journal of Public Health. 2014;38(3):247-52. doi:10.1111/1753-6405.12232.
342. Rossi MCP, C.; Consorti, C.; Almonti, T.; Foglini, P.; Giostra, N.; Nanni, P.; Talevi, S.; Bartolomei, D.; Vespasiani, G. An interactive diary for diet management (DAI): a new telemedicine system able to promote body weight reduction, nutritional education, and consumption of fresh local produce. Diabetes Technology and Therapeutics. 2010 Aug;12(8):641-7. PMID: 20615106. doi:10.1089/dia.2010.0025.
343. Rusin M, Årsand E, Hartvigsen G. Functionalities and input methods for recording food intake: a systematic review. Int J Med Inf. 2013 Aug;82(8):653-64. PMID: 23415822. doi:10.1016/j.ijmedinf.2013.01.007.
344. Sanford AA. 'I Can Air My Feelings Instead of Eating Them': Blogging as Social Support for the Morbidly Obese. Communication Studies. 2010;61(5):567-84. PMID: 55053427. doi:10.1080/10510974.2010.514676.
345. Savolainen R. Dietary blogs as sites of informational and emotional support. Information Research. 2010 Dec;15(4):12.
346. Schap TEZ, F.; Delp, E. J.; Boushey, C. J. Merging dietary assessment with the adolescent lifestyle. Journal of Human Nutrition and Dietetics. 2014;27(s1):82-8. PMID: 20143057919.
347. Schiel RK, A.; Bieber, G.; Kramer, G.; Seebach, H.; Hoffmeyer, A. Identification of determinants for weight reduction in overweight and obese children and adolescents. Journal of Telemedicine and Telecare. 2010;16(7):368-73. PMID: 20679405. doi:10.1258/jtt.2010.091005.
348. Schiel RK, A.; Bieber, G. Electronic health technology for the assessment of physical activity and eating habits in children and adolescents with overweight and obesity IDA. Appetite. 2012 Apr;58(2):432-7. PMID: 22155072. doi:10.1016/j.appet.2011.11.021.
349. Schiel RT, A.; Kaps, A.; Bieber, G. An innovative telemedical support system to measure physical activity in children and adolescents with type 1 diabetes mellitus. Experimental and Clinical Endocrinology and Diabetes. 2011 Oct;119(9):565-8. PMID: 21472657. doi:10.1055/s-0031-1273747.
350. Schoffman DE, Turner-McGrievy G, Jones SJ, Wilcox S. Mobile apps for pediatric obesity prevention and treatment, healthy eating, and physical activity promotion: Just fun and games? Translational Behavioral Medicine. 2013 September;3(3):320-5. PMID: 2013576826. doi:10.1007/s13142-013-0206-3.
351. Schwerdtfeger ARS, Catalina; Warken, Matthias. Using text messages to bridge the intention-behavior gap? A pilot study on the use of text message reminders to increase objectively assessed physical activity in daily life. . Frontiers in Psychology. 2012;3:270. doi:10.3389/fpsyg.2012.00270.

352. Sevick MAS, R. A.; Zickmund, S.; Wang, Y.; Korytkowski, M.; Burke, L. E. Factors associated with probability of personal digital assistant-based dietary self-monitoring in those with type 2 diabetes. Journal of Behavioral Medicine. 2010;33(4):315-25. PMID: Cn-00762271. doi:10.1007/s10865-010-9257-9.
353. Shapiro JRB, S.; Hamer, R. M.; Kordy, H.; Ward, D.; Bulik, C. M. Use of text messaging for monitoring sugar-sweetened beverages, physical activity, and screen time in children: a pilot study. Journal of Nutrition Education and Behavior. 2008;40(6):385-91. PMID: Cn-00738762.
354. Shapiro JRK, T.; Doran, N.; Thompson, S.; Sallis, J. F.; Calfas, K.; Patrick, K. Text4Diet: a randomized controlled study using text messaging for weight loss behaviors. Preventive Medicine. 2012;55(5):412-7. PMID: Cn-00967898. doi:10.1016/j.ypmed.2012.08.011.
355. Sharifi MD, Eileen M.; Horan, Christine M.; Price, Sarah; Marshall, Richard; Hacker, Karen; Finkelstein, Jonathan A.; Taveras, Elsie M. Leveraging text messaging and mobile technology to support pediatric obesity-related behavior change: A qualitative study using parent focus groups and interviews. J Med Internet Res. 2013;15(12):60-70. PMID: 2014-01844-005.
356. Sharp DBA-F, M. Feasibility and validity of mobile phones to assess dietary intake. Nutrition. 2014 Nov-Dec;30(11-12):1257-66. doi:10.1016/j.nut.2014.02.020.
357. Shaw RB, H. Short message service (SMS) text messaging as an intervention medium for weight loss: a literature review. Health Inform J. 2012;18(4):235-50. PMID: DARE-12013006020. doi:10.1177/1460458212442422.
358. Shaw RJ, Bosworth HB, Hess JC, Silva SG, Lipkus IM, Davis LL, et al. Development of a Theoretically Driven mHealth Text Messaging Application for Sustaining Recent Weight Loss. JMIR mhealth and uhealth. 2013 2013/05/07/;1(1). doi:10.2196/mhealth.2343.
359. Shaw RJB, H. B.; Silva, S. S.; Lipkus, I. M.; Davis, L. L.; Sha, R. S.; Johnson, C. M. Mobile health messages help sustain recent weight loss. American Journal of Medicine. 2013;126(11):1002-9. PMID: CN-00962613. doi:10.1016/j.amjmed.2013.07.001.
360. Shaw RJS, D. M.; Zullig, L. L.; Bosworth, H. B.; Johnson, C. M.; Davis, L. L. mHealth interventions for weight loss: a guide for achieving treatment fidelity. Journal of the American Medical Informatics Association. 2014 Nov;21(6):959-63. doi:10.1136/amiajnl-2013-002610.
361. Shay LE, Seibert D, Watts D, Sbrocco T, Pagliara C. Adherence and weight loss outcomes associated with food-exercise diary preference in a military weight management program. Eating Behaviors. 2009 2009/12//;10(4):220-7. doi:10.1016/j.eatbeh.2009.07.004.
362. Shrewsbury VAOC, J.; Steinbeck, K. S.; Stevenson, K.; Lee, A.; Hill, A. J.; Kohn, M. R.; Shah, S.; Torvaldsen, S.; Baur, L. A. A randomised controlled trial of a community-based healthy lifestyle program for overweight and obese adolescents: the Loozit study protocol. BMC Public Health. 2009;9:119. PMID: CN-00720924. doi:10.1186/1471-2458-9-119.
363. Silva BML, I. M.; Rodrigues, J. J. P. C.; Ray, P., editor. SapoFitness: A mobile health application for dietary evaluation. 2011 IEEE 13th International Conference on e-Health Networking Applications and Services (Healthcom); 2011 June 13-15, 2011; Columbia, MO, USA.
364. Silva PA, Holden K, Nii A, editors. Smartphones, Smart Seniors, But Not-So-Smart Apps: A Heuristic Evaluation of Fitness Apps. 8th International Conference, Augmented Cognition, AC 2014, Held as Part of HCI International 2014; 2014 June 22-27, 2014; Heraklion, Crete, Greece,.
365. Silveira PvdL, Rolf; van het Reve, Eva; Daniel, Florian; Casati, Fabio; de Bruin, Eling D. Tablet-based strength-balance training to motivate and improve adherence to exercise in independently living older people: A phase II preclinical exploratory trial. J Med Internet Res. 2013;15(8):114-30. PMID: 2013-41345-009.
366. Silveira PvhR, E.; Daniel, F.; Casati, F.; de Bruin, E. D. Motivating and assisting physical exercise in independently living older adults: a pilot study. Int J Med Inf. 2013 May;82(5):325-34. PMID: 23273420. doi:10.1016/j.ijmedinf.2012.11.015.
367. Simunaniemi A-MS, Helena; Andersson, Agneta; Nydahl, Margaretha. Laypeople blog about fruit and vegetables for self-expression and dietary influence. . Health Communication. 2011;26(7):621-30. doi:10.1080/10410236.2011.561520.
368. Siopis GC, T.; Allman-Farinelli, M. A systematic review and meta-analysis of interventions for weight management using text messaging (Provisional abstract). Journal of Human Nutrition and Dietetics. 2014 (3):epub. PMID: DARE-12014012179. doi:10.1111/jhn.12207.
369. Sirriyeh RL, R.; Ward, J. Physical activity and adolescents: an exploratory randomized controlled trial investigating the influence of affective and instrumental text messages. British Journal of health Psychology. 2010;15(Pt 4):825-40. PMID: CN-00772053. doi:10.1348/135910710X486889.
370. Six BLS, T. E.; Zhu, F. M.; Mariappan, A.; Bosch, M.; Delp, E. J.; Ebert, D. S.; Kerr, D. A.; Boushey, C. J. Evidence-based development of a mobile telephone food record. J Am Diet Assoc. 2010 Jan;110(1):74-9. PMID: 20102830. doi:10.1016/j.jada.2009.10.010.
371. Six BLS, T. E.; Kerr, D. A.; Boushey, C. J. Evaluation of the Food and Nutrient Database for Dietary

Studies for use with a mobile telephone food record. Journal of Food Composition and Analysis. 2011;24(8):1160-7. PMID: 20113408375. doi:10.1016/j.jfca.2011.06.006.
372. Smith JJ, Morgan PJ, Plotnikoff RC, Dally KA, Salmon J, Okely AD, et al. Smart-phone obesity prevention trial for adolescent boys in low-income communities: the ATLAS RCT. Pediatrics. 2014 2014/09//;134(3):e723-31. doi:10.1542/peds.2014-1012.
373. Smith JJM, P. J.; Plotnikoff, R. C.; Dally, K. A.; Salmon, J.; Okely, A. D.; Finn, T. L.; Babic, M. J.; Skinner, G.; Lubans, D. R. Rationale and study protocol for the 'Active Teen Leaders Avoiding Screen-time' (ATLAS) group randomized controlled trial: An obesity prevention intervention for adolescent boys from schools in low-income communities. Contemp Clin Trials. 2014;37(1):106-19. PMID: CN-00961814. doi:10.1016/j.cct.2013.11.008.
374. Smith LPH, J.; Seto, E.; Du, S.; Zang, J.; Zou, S.; Popkin, B. M.; Mendez, M. A. Development and validity of a 3-day smartphone assisted 24-hour recall to assess beverage consumption in a Chinese population: a randomized cross-over study. Asia Pacific journal of clinical nutrition. 2014;23(4):678-90. PMID: 25516327. doi:10.6133/apjcn.2014.23.4.10.
375. Sniehotta FFP, J.; Hobbs, N.; Araújo-Soares, V. Testing self-regulation interventions to increase walking using factorial randomized N-of-1 trials. Health Psychology. 2012;31(6):733-7. PMID: CN-00970469. doi:10.1037/a0027337.
376. Soureti AM, P.; Cobain, M.; Chinapaw, M.; Mechelen, W.; Hurling, R. Exploratory study of web-based planning and mobile text reminders in an overweight population. J Med Internet Res. 2011;13(4):e118. PMID: CN-00860418. doi:10.2196/jmir.1773.
377. Spillers FA, S., editor. Does Social User Experience Improve Motivation for Runners? A Diary Study Comparing Mobile Health Applications. 1st International Conference on Design, User Experience, and Usability, DUXU 2011; 14th International Conference on Human-Computer Interaction (HCI); 2014 July 9-14, 2011; Orlando, FL, USA.
378. Spook JEP, T.; Kok, G.; Van Empelen, P. Monitoring dietary intake and physical activity electronically: feasibility, usability, and ecological validity of a mobile-based Ecological Momentary Assessment tool. J Med Internet Res. 2013;15(9):e214. PMID: 24067298. doi:10.2196/jmir.2617.
379. Spring BD, J. M.; Janke, E. A.; Kozak, A. T.; McFadden, H. G.; DeMott, A.; Pictor, A.; Epstein, L. H.; Siddique, J.; Pellegrini, C. A.; Buscemi, J.; Hedeker, D. Integrating technology into standard weight loss treatment: a randomized controlled trial. JAMA Internal Medicine. 2013;173(2):105-11. PMID: CN-00848278. doi:10.1001/jamainternmed.2013.1221.
380. Spring BS, K.; McFadden, H. G.; Vaughn, J.; Kozak, A. T.; Smith, M.; Moller, A. C.; Epstein, L.; Russell, S. W.; DeMott, A.; Hedeker, D. Make Better Choices (MBC): study design of a randomized controlled trial testing optimal technology-supported change in multiple diet and physical activity risk behaviors. BMC Public Health. 2010;10:586. PMID: Cn-00772508. doi:10.1186/1471-2458-10-586.
381. Spring BS, K.; McFadden, H. G.; Vaughn, J.; Kozak, A. T.; Smith, M.; Moller, A. C.; Epstein, L. H.; Demott, A.; Hedeker, D.; Siddique, J.; Lloyd-Jones, D. M. Multiple behavior changes in diet and activity: a randomized controlled trial using mobile technology. Archives of internal Medicine. 2012;172(10):789-96. PMID: CN-00840335. doi:10.1001/archinternmed.2012.1044.
382. Steinberg DML, E. L.; Askew, S.; Foley, P.; Bennett, G. G. Daily Text Messaging for Weight Control Among Racial and Ethnic Minority Women: Randomized Controlled Pilot Study. J Med Internet Res. 2013 Nov;15(11):11. doi:10.2196/jmir.2844.
383. Stephens JA, J. Mobile phone interventions to increase physical activity and reduce weight: a systematic review (Provisional abstract). Journal of Cardiovascular Nursing. 2013;28(4):320-9. PMID: Dare-12013046090.
384. Sternfeld BJ, S. F.; Picchi, T.; Chasan-Taber, L.; Ainsworth, B.; Quesenberry, C. P., Jr. Evaluation of a cell phone-based physical activity diary. Medicine and Science in Sports and Exercise. 2012 Mar;44(3):487-95. PMID: 21857369. doi:10.1249/MSS.0b013e3182325f45.
385. Stroulia EF, S.; Bazelli, B.; Gibbs, D.; Lederer, R.; Faulkner, R.; Ferguson-Roberts, J.; Mullen, B., editor. Smart-phone Application Design for Lasting Behavioral Changes. 2013 IEEE 26th International Symposium on Computer-Based Medical Systems (CBMS); 2013 June 20-22, 2013; Porto, Portugal.
386. Struempler BJP, S. M.; Mastropietro, L. M.; Arsiwalla, D.; Bubb, R. R. Changes in fruit and vegetable consumption of third-grade students in body quest: food of the warrior, a 17-class childhood obesity prevention program. Journal of Nutrition Education and Behavior. 2014;46(4):286-92. PMID: 20143228029. doi:10.1016/j.jneb.2014.03.001.
387. Stuckey MR-M, E.; Read, E.; Munoz, C.; Shoemaker, K.; Kleinstiver, P.; Petrella, R. Diabetes and Technology for Increased Activity (DaTA) study: results of a remote monitoring intervention for prevention of metabolic syndrome. Journal of Diabetes Science and Technology. 2011 Jul;5(4):928-35. PMID: 21880236.
388. Stumbo PJ, editor. New technology in dietary assessment: a review of digital methods in improving food

record accuracy. Conference on ‘Translating nutrition: integrating research, practice and policy’ 2013 July 16-19, 2012; Belfast, Northern Ireland.

389. Sugano M, Yamakazi C, editors. Behavioral Analysis of SNS Users with Regard to Diet. IADIS International Conferences - Web Based Communities and Social Media, Social Media 2011, Internet Applications and Research 2011, Part of the IADIS Multi Conference on Computer Science and Information Systems 2011, MCCSIS; 2011 July 20-26, 2011; Rome, Italy. Rome.

390. Suggs SB, Holly; Bardus, Marco; Lloyd, Scott. Effects of text messaging in addition to emails on physical activity among university and college employees in the UK. Journal of Health Services Research & Policy. 2013 April 1, 2013;18(1 suppl):56-64. doi:10.1177/1355819613478001.

391. Sun LZ, D. Q.; Li, N., editor. Physical Activity Monitoring with Mobile Phones. 9th International Conference on Smart Homes and Health Telematics (ICOST 2011) 2011 June 20-22, 2011; Montreal, Canada: Springer-Verlag Berlin.

392. Tang JA, Charles; Greaves, Colin; Yates, Tom. Self-directed interventions to promote weight loss: A systematic review of reviews. . J Med Internet Res. 2014;16(2):79-93. doi:10.2196/jmir.2857.

393. Taveras EMM, J.; O'Brien, A.; Haines, J.; Sherry, B.; Bottino, C. J.; Troncoso, K.; Schmidt, M. E.; Koziol, R. Healthy Habits, Happy Homes: methods and baseline data of a randomized controlled trial to improve household routines for obesity prevention. Preventive Medicine. 2012;55(5):418-26. PMID: Cn-00967798. doi:10.1016/j.ypmed.2012.08.016.

394. Thatte GL, M.; Lee, S.; Emken, A.; Narayanan, S.; Mitra, U.; Spruijt-Metz, D.; Annavaram, M. KNOWME: An Energy-Efficient Multimodal Body Area Network for Physical Activity Monitoring. Acm Transactions on Embedded Computing Systems. 2012 Aug;11:24. doi:10.1145/2331147.2331158.

395. Thomas JGB, Dale S. Review of innovations in digital health technology to promote weight control. Current Diabetes Reports. 2014;14(5):485.

396. Thomas JGW, Rena R. Health-E-Call, a Smartphone-Assisted Behavioral Obesity Treatment: Pilot Study. JMIR mHealth and uHealth. 2013;1(1):e3. PMID: PMC4114436. doi:10.2196/mhealth.2164.

397. Thorsteinsen KV, J.; Svendsen, G. B. Increasing physical activity efficiently: An experimental pilot study of a website and mobile phone intervention. International Journal of Telemedicine and Applications. 2014 (746232). PMID: 2014400708. doi:10.1155/2014/746232.

398. Toscos T, Faber A, An S, Gandhi MP, editors. Chick Clique: Persuasive Technology to Motivate Teenage Girls to Exercise. Conference on Human Factors in Computing Systems, CHI 2006; 2006 Apri 22-27, 2006; Montreal, Canada. New York, NY, USA: ACM.

399. Toscos T, Faber A, Connelly K, Upoma AM, editors. Encouraging physical activity in teens Can technology help reduce barriers to physical activity in adolescent girls? 2008 2nd International Conference on Pervasive Computing Technologies for Healthcare (PervasiveHealth); 2008 January 30-February 1, 2008; Tampere, Finland.

400. Toscos TC, S.; McDonald, D. W. Barriers to Physical Activity: A Study of Self-Revelation in an Online Community. J Med Syst. 2011 Oct;35(5):1225-42. doi:10.1007/s10916-011-9721-2.

401. Tsai CCL, Gunny; Raab, Fred; Norman, Gregory J.; Sohn, Timothy; Griswold, William G.; Patrick, Kevin, editor. Usability and Feasibility of PmEB: A Mobile Phone Application for Monitoring Real Time Caloric Balance. Pervasive Health Conference and Workshops, 2006; 2006 November 29-December 1, 2006; Innsbruck, Austria.

402. Turk MW, Elci OU, Wang J, Sereika SM, Ewing LJ, Acharya SD, et al. Self-monitoring as a mediator of weight loss in the SMART randomized clinical trial. International Journal of Behavioral Medicine. 2013 2013/12//;20(4):556-61. doi:10.1007/s12529-012-9259-9.

403. Turner-McGrievy GK, Sri; Campbell, Marci K. Delivering health information via podcast or Web: Media effects on psychosocial and physiological responses. . Health Communication. 2013;28(2):101-9. doi:10.1080/10410236.2011.651709.

404. Turner-McGrievy GMB, M. W.; Moore, J. B.; Kaczynski, A. T.; Barr-Anderson, D. J.; Tate, D. F. Comparison of traditional versus mobile app self-monitoring of physical activity and dietary intake among overweight adults participating in an mHealth weight loss program. Journal of the American Medical Informatics Association. 2013;20(3):513-8. PMID: CN-00877255. doi:10.1136/amiajnl-2012-001510.

405. Turner-McGrievy GMC, M. K.; Tate, D. F.; Truesdale, K. P.; Bowling, J. M.; Crosby, L. Pounds Off Digitally study: a randomized podcasting weight-loss intervention. American Journal of Preventive Medicine. 2009;37(4):263-9. PMID: Cn-00722028. doi:10.1016/j.amepre.2009.06.010.

406. Turner-McGrievy GMD, C. R.; Wilcox, S. Does the type of weight loss diet affect who participates in a behavioral weight loss intervention? A comparison of participants for a plant-based diet versus a standard diet trial. Appetite. 2014 Feb;73:156-62. doi:10.1016/j.appet.2013.11.008.

407. Turner-McGrievy GMT, D. F. Weight loss social support in 140 characters or less: Use of an online social network in a remotely delivered weight loss intervention. Translational Behavioral Medicine. 2013;3(3):287-94. PMID: Cn-00916478. doi:10.1007/s13142-012-0183-y.

408. Turner-McGrievy GMT, D. F. Are we sure that Mobile Health is really mobile? An examination of mobile device use during two remotely-delivered weight loss interventions. Int J Med Inf. 2014;83(5):313-9. PMID: Cn-00984690. doi:10.1016/j.ijmedinf.2014.01.002.
409. Turner-McGrievy GT, D. Tweets, Apps, and Pods: Results of the 6-month Mobile Pounds Off Digitally (Mobile POD) randomized weight-loss intervention among adults. J Med Internet Res. 2011;13(4):e120. PMID: Cn-00860407. doi:10.2196/jmir.1841.
410. Valentin GH, A. M., editor. Dealing with childhood obesity: Passive versus active activity monitoring approaches for engaging individuals in exercise. Biosignals and Biorobotics Conference (BRC), 2013 ISSNIP; 2013 February 18-20, 2013; Rio de Janeiro, Brazil.
411. Valle CG, Tate DF, Mayer DK, Allicock M, Cai J. A randomized trial of a Facebook-based physical activity intervention for young adult cancer survivors. J Cancer Surviv. 2013 2013/09//;7(3):355-68. doi:10.1007/s11764-013-0279-5.
412. van Dantzig SG, G.; van Halteren, A. T. Toward a persuasive mobile application to reduce sedentary behavior. Personal and Ubiquitous Computing. 2013 Aug;17(6):1237-46. doi:10.1007/s00779-012-0588-0.
413. van der Weegen SV, R.; Tange, H. J.; Spreeuwenberg, M. D.; de Witte, L. P. Usability testing of a monitoring and feedback tool to stimulate physical activity. Patient Preference and Adherence. 2014 17 Mar;8:311-22. PMID: 2014205697. doi:10.2147/PPA.S57961.
414. Vandelanotte C, Kirwan M, Rebar A, Alley S, Short C, Fallon L, et al. Examining the use of evidence-based and social media supported tools in freely accessible physical activity intervention websites. International Journal of Behavioral Nutrition and Physical Activity. 2014;11(1):1-20. PMID: 2012704039. Language: English. Entry Date: 20140919. Revision Date: 20140919. Publication Type: journal article. doi:10.1186/s12966-014-0105-0.
415. Vandelanotte CC, C. M.; Ellison, M.; George, E. S.; Maeder, A.; Kolt, G. S.; Duncan, M. J.; Mohanraj, Karunanithi; Noakes, M.; Hooker, C.; Viljoen, P.; Mummery, W. K. What kinds of website and mobile phone-delivered physical activity and nutrition interventions do middle-aged men want? (Special Issue: Advancing shared conversation to enhance health care enrollment in the United States-a timely opportunity and challenge for health literacy.). Journal of Health Communication: International Perspectives. 2013;18(9):1070-83. PMID: 20133325516. doi:10.1080/10810730.2013.768731.
416. Vankipuram MM, S.; Fleury, J., editor. ReadySteady: app for accelerometer-based activity monitoring and wellness-motivation feedback system for older adults. 2012 American Medical Informatics Association (AMIA) Annual Symposium; 2012 November 3-7, 2012; Chicago, IL, USA: AMIA; PMC3540520.
417. Vathsangam HZ, M.; Tarashansky, A.; Sawchuk, A. A.; Sukhatme, G. S., editor. Towards Practical Energy Expenditure Estimation With Mobile Phones. 47th Asilomar Conference on Signals, Systems and Computers; 2013 November 3-6, 2013; Pacific Grove, CA, USA. NEW YORK: Ieee; 2013.
418. Verwey RvdW, S.; Tange, H.; Spreeuwenberg, M.; van der Weijden, T.; de Witte, L. Get moving: the practice nurse is watching you! A case study of the user-centred design process and testing of a web-based coaching system to stimulate the physical activity of chronically ill patients in primary care. Inform Prim Care. 2012;20(4):289-98. PMID: 23890341.
419. Verwey RvdW, Sanne; Spreeuwenberg, Marieke; Tange, Huibert; van der Weijden, Trudy; de Witte, Luc. A pilot study of a tool to stimulate physical activity in patients with COPD or type 2 diabetes in primary care. . Journal of Telemedicine and Telecare. 2014;20(1):29-34. doi:10.1177/1357633X13519057.
420. Villalobos GA, Rana; Hariri, Behnoosh; Shirmohammadi, Shervin, editor. A Personal Assistive System for Nutrient Intake Monitoring. Ubi-MUI '11: MM International Multimedia Conference; 2011 November 28-December 1, 2011; Scottsdale, AR, USA. New York, NY, USA: ACM.
421. Wang DHK, M.; Kira, S. Development of a new instrument for evaluating individuals' dietary intakes. J Am Diet Assoc. 2006 Oct;106(10):1588-93. PMID: 17000191.
422. Wang J, Sereika S, Chasens, Ewing, Matthews, Burke L. Effect of adherence to self-monitoring of diet and physical activity on weight loss in a technology-supported behavioral intervention. Patient Preference and Adherence. 2012 2012/03//. doi:10.2147/PPA.S28889.
423. Watkins I, Kules B, Yuan X, Xie B. Heuristic Evaluation of Healthy Eating Apps for Older Adults. Journal of Consumer Health on the Internet. 2014;18(2):105-27. PMID: 2012618232. Language: English. Entry Date: 20140704. Revision Date: 20140711. Publication Type: journal article. doi:10.1080/15398285.2014.902267.
424. Wearing J, Nollen N, Befort C, Davis AM, Agemy CK. iPhone app adherence to expert-recommended guidelines for pediatric obesity prevention. Child. 2014;10(2):132-44.
425. Wedderkopp NJ, E.; Franz, C.; Klakk, H.; Heidemann, M.; Christiansen, C.; Moller, N. C.; Leboeuf-Yde, C. Study protocol. The Childhood Health, Activity, and Motor Performance School Study Denmark (The CHAMPS-study DK). BMC Pediatr. 2012;12:128. PMID: 22906115. doi:10.1186/1471-2431-12-128.
426. Wei J, Hollin I, Kachnowski S. A review of the use of mobile phone text messaging in clinical and healthy behaviour interventions. Journal of Telemedicine and Telecare. 2011 2011;17(1):41-8.

doi:10.1258/jtt.2010.100322.
427. Weiss R, Stumbo PJ, Divakaran A. Automatic Food Documentation and Volume Computation using Digital Imaging and Electronic Transmission. J Am Diet Assoc. 2010 2010/01//;110(1). doi:10.1016/j.jada.2009.10.011.
428. West JH, Hall PC, Arredondo V, Berrett B, Guerra B, Farrell J. Health Behavior Theories in Diet Apps. Journal of Consumer Health on the Internet. 2013 2013 Jan-Mar;17(1):10-24. PMID: 2011941754. Language: English. Entry Date: 20130301. Revision Date: 20140704. Publication Type: journal article. doi:10.1080/15398285.2013.756343.
429. West JH, Hall PC, Hanson CL, Barnes MD, Giraud-Carrier C, Barrett J. There's an app for that: content analysis of paid health and fitness apps. J Med Internet Res. 2012;14(3):e72. PMID: 22584372. doi:10.2196/jmir.1977.
430. Wharton CMJ, Carol S.; Cunningham, Barbara K.; Sterner, Danielle. Dietary Self-Monitoring, But Not Dietary Quality, Improves With Use of Smartphone App Technology in an 8-Week Weight Loss Trial. Journal of Nutrition Education and Behavior. 2014;46(5):440-4. PMID: 98259108.
431. Williams AD. Use of a text messaging program to promote adherence to daily physical activity guidelines: a review of the literature. Bariatric Nursing and Surgical Patient Care. 2012;7(1):13-6. PMID: 2011488735. Language: English. Entry Date: 20120525. Revision Date: 20120525. Publication Type: journal article. doi:10.1089/bar.2012.9999.
432. Williams GH, M. P.; Shulhan, J.; Vandermeer, B.; Hartling, L. Social media interventions for diet and exercise behaviours: a systematic review and meta-analysis of randomised controlled trials (Provisional abstract). BMJ Open. 2014;4(2):e003926. PMID: DARE-12014015422.
433. Wohlers EMS, J. R.; Barden, C. M.; Moon, J. K., editor. Smart phones are useful for food intake and physical activity surveys. 31st Annual International Conference of the IEEE Engineering in Medicine and Biology Society (EMBS); 2009 September 3-6, 2009; Minneapolis, MN, USA.
434. Womble LG, Wadden TA, McGuckin BG, Sargent SL, Rothman RA, Krauthamer-Ewing ES. A Randomized Controlled Trial of a Commercial Internet Weight Loss Program. Obesity Research. 2004 2004/06/01/;12(6):1011-8. doi:10.1038/oby.2004.124.
435. Woolford SJB, K. L. C.; Derry, H. A.; Jepson, C. M.; Clark, S. J.; Strecher, V. J.; Resnicow, K. OMG do not say LOL: obese adolescents' perspectives on the content of text messages to enhance weight loss efforts. Obesity (Silver Spring). 2011;19(12):2382-7. PMID: 20113389524. doi:10.1038/oby.2011.266.
436. Woolford SJC, S. J.; Strecher, V. J.; Resnicow, K. Tailored mobile phone text messages as an adjunct to obesity treatment for adolescents. Journal of Telemedicine and Telecare. 2010;16(8):458-61. PMID: 20959393. doi:10.1258/jtt.2010.100207.
437. Woolford SJEM, A. D.; Sami, A.; Blake, N. Let's face it: patient and parent perspectives on incorporating a Facebook group into a multidisciplinary weight management program. Child. 2013 Aug;9(4):305-10. PMID: 23869854. doi:10.1089/chi.2013.0047.
438. Woolford SJK, S.; Barr, K. L. C.; Clark, S. J.; Strecher, V. J.; Resnicow, K. A picture may be worth a thousand texts: obese adolescents' perspectives on a modified photovoice activity to aid weight loss. Child. 2012;8(3):230-6. PMID: 20123232924.
439. Wu WD, S.; Ramirez, E. E.; Peterson, C.; Norman, G. J. Classification accuracies of physical activities using smartphone motion sensors. J Med Internet Res. 2012;14(5):e130. PMID: 23041431. doi:10.2196/jmir.2208.
440. Wylie CGC, P., editor. Persuasive Mobile Health Applications. 1st International Conference on Electronic Healthcare; 2009 September 8-9, 2008; London, UK: Springer.
441. Xia YC, V.; Garcia, E.; Ding, H.; Karunaithi, M. Development of an automated physical activity classification application for mobile phones. Studies in Health Technology and Informatics. 2011;168:188-94. PMID: 21893928.
442. Xu JYH, I. Chang; Chieh, Chien; Kaiser, W. J.; Pottie, G. J. Context-driven, Prescription-Based Personal Activity Classification: Methodology, Architecture, and End-to-End Implementation. Biomedical and Health Informatics, IEEE Journal of. 2014;18(3):1015-25. doi:10.1109/JBHI.2013.2282812.
443. Xue Y, Hu YQ, Jin LW. Activity Recognition Based on an Accelerometer in a Smartphone Using an FFT-Based New Feature and Fusion Methods. Ieice Transactions on Information and Systems. 2014 Aug;E97D(8):2182-6. doi:10.1587/transinf.E97.D.2182.
444. Yen WJL, N. M. MyPyramid-omega-3 fatty acid nutrition education intervention may improve food groups and omega-3 fatty acid consumption in university middle-aged women. Nutrition Research. 2013;33(2):103-8. PMID: Cn-00965462. doi:10.1016/j.nutres.2012.11.015.
445. Yon BA, Johnson RK, Harvey-Berino J, Gold BC. The Use of a Personal Digital Assistant for Dietary Self-Monitoring Does Not Improve the Validity of Self-Reports of Energy Intake. J Am Diet Assoc. 2006 2006/08//;106(8):1256-9. doi:10.1016/j.jada.2006.05.004.
446. Yon BA, Johnson RK, Harvey-Berino J, Gold BC, Howard AB. Personal Digital Assistants are

Comparable to Traditional Diaries for Dietary Self-Monitoring During a Weight Loss Program. Journal of Behavioral Medicine. 2007 2007/04/01/;30(2):165-75. doi:10.1007/s10865-006-9092-1.
447. Youm SP, S. H. Development and Evaluation of a Mobile Application for Personal Lifestyle Check-Up and Improvement. Telemedicine and e-Health. 2014 Nov;20(11):1057-63. doi:10.1089/tmj.2013.0335.
448. Young MM, editor. Twitter Me: Using Micro-blogging to Motivate Teenagers to Exercise. 5th International Conference on Design Science Research in Informatin Systems and Technology; 2010 June 4-5, 2010; St. Gallen, Switzerland: Springer-Verlag Berlin.
449. Young-Seob J, Ho-Jin C, Yong-Jin K, Kyu-Chang K, Ju-Yeon L, Hye-Hyon K, et al., editors. Semi-automated lifestyle manager for obesity. Big Data and Smart Computing (BIGCOMP), 2014 International Conference on; 2014 15-17 Jan. 2014.
450. Yusuf FM, A.; Basilakis, J. Hierarchical classifier approach to physical activity recognition via wearable smartphone tri-axial accelerometer. Studies in Health Technology and Informatics. 2013;188:174-80. PMID: 23823307.
451. Yuzhong LJ, J.; de Vries, B.; Timmermans, H., editor. Motivate: Towards context-aware recommendation mobile system for healthy living. 2011 5th International Conference on Pervasive Computing Technologies for Healthcare (PervasiveHealth); 2011 May 23-26, 2011; Dublin, Ireland.
452. Zhong S, Wang L, Bernardos AM, Song M, editors. An accurate and adaptive pedometer integrated in mobile health application. IET International Conference on Wireless Sensor Network, 2010 IET-WSN; 2010 November 15-17, 2010; Beijing, China.
453. Zhu F, Bosch M, Woo I, Kim S, Boushey CJ, Ebert DS, et al. The Use of Mobile Devices in Aiding Dietary Assessment and Evaluation. IEEE J Sel Top Signal Process. 2010 2010/08//;4(4):756-66. doi:10.1109/JSTSP.2010.2051471.
454. Zhu F, Mariappan A, Boushey CJ, Kerr D, Lutes KD, Ebert DS, et al., editors. Technology-Assisted Dietary Assessment. IS&T/SPIE Conference on Computational Imaging; 2008 January 28-29, 2008; San Jose, CA, USA. San Jose.
455. Zongjian HY, Luo; Guanqing, Liang, editor. Runking: A mobile social persuasion system for running exercise. 2013 Computing, Communications and IT Applications Conference (ComComAp); 2013 April 1-4, 2013; Hong Kong, China.
456. Zuckerman OG-O, A. Deconstructing gamification: evaluating the effectiveness of continuous measurement, virtual rewards, and social comparison for promoting physical activity. Personal and Ubiquitous Computing. 2014 Oct;18(7):1705-19. doi:10.1007/s00779-014-0783-2.
457. Zwinderman MJS, Azadeh; Ma, Xinyu; Bajracharya, Prina; Sandberg, Hans; Kaptein, Maurits Clemens, editor. Phone Row: A Smartphone Game Designed to Persuade People to Engage in Moderate-intensity Physical Activity. 7th international conference on Persuasive Technology: design for health and safety PERSUASIVE 2012; 2012 June 6-8, 2012; Linköping, Sweden. Berlin, Heidelberg: Springer-Verlag.
