# Supplementary material for: Mobile Phone and Web 2.0 Technologies for Weight Management: A Systematic Scoping Review
Source: J Med Internet Res. 2015 Nov 16;17(11):e259. doi: 10.2196/jmir.5129 (PMC4704945; doi:10.2196/jmir.5129)
Supplement: Multimedia Appendix 4 [file jmir_v17i11e259_app4.pdf]

Mobile Phone and Web 2.0 technologies for Weight Management: a Systematic Scoping Review

Multimedia Appendix 4 – Excluded refrences with reasons

ID	Study	Reason for exclusion
1	Anonymous (2005)	Reference type: not full-text article
2	Abebe (2013)	Type of study: Not original research
3	Abraham (2014)	Reference type: not full-text article
4	Anderson (2014)	Technology: not focus on mobile devices and/or Web 2.0/social media
5	Anton (2012)	Technology: not focus on mobile devices and/or Web 2.0/social media
6	Arbogast (2013)	Reference type: thesis/dissertation (full-text mostly not available)
7	Arora (2012)	Scope: not focus on weight management/obesity prevention
8	Årsand (2012)	Scope: not focus on weight management/obesity prevention
9	Årsand (2008)	Scope: not focus on weight management/obesity prevention
10	Årsand (2010)	Scope: not focus on weight management/obesity prevention
11	Arvidsson (2007)	Technology: not focus on mobile devices and/or Web 2.0/social media
12	Bakken (2014)	Scope: not focus on weight management/obesity prevention
13	Baranowski (2012)	Type of study: Not original research
14	Barragan (2014)	Type of study: Not original research
15	Baulch (2008)	Type of study: Not original research
16	Berke (2011)	Technology: not focus on mobile devices and/or Web 2.0/social media
17	Biddle (2014)	Technology: not focus on mobile devices and/or Web 2.0/social media
18	Binks (2011)	Reference type: not full-text article
19	Blanso (2011)	Scope: not focus on weight management/obesity prevention
20	Bond (2013)	Technology: not focus on mobile devices and/or Web 2.0/social media
21	Breland (2013)	Scope: not focus on weight management/obesity prevention
22	Brindal (2013)	Duplicate
23	Brooke (2013)	Scope: not focus on weight management/obesity prevention
24	Brox (2011)	Technology: not focus on mobile devices and/or Web 2.0/social media
25	Burner (2013)	Scope: not focus on weight management/obesity prevention
26	Butler (2010)	Type of study: Not original research
27	Carnie (2013)	Technology: not focus on mobile devices and/or Web 2.0/social media
28	Carrard (2011)	Technology: not focus on mobile devices and/or Web 2.0/social media
29	Carter (2013)	Reference type: thesis/dissertation (full-text mostly not available)
30	Castelnuovo (2014)	Type of study: Not original research

ID	Study	Reason for exclusion
31	Castelnuovo (2011)	Technology: not focus on mobile devices and/or Web 2.0/social media
32	Cavallo (2012)	Reference type: thesis/dissertation (full-text mostly not available)
33	Centis (2013)	Technology: not focus on mobile devices and/or Web 2.0/social media
34	Choi (2013)	Reference type: not full-text article
35	Chomutare (2011)	Scope: not focus on weight management/obesity prevention
36	Chuah (2012)	Reference type: not full-text article
37	Clark (2006)	Scope: not focus on weight management/obesity prevention
38	Clary (2010)	Technology: not focus on mobile devices and/or Web 2.0/social media
39	Cleland (2013)	Scope: not focus on weight management/obesity prevention
40	Corsino (2013)	Scope: not focus on weight management/obesity prevention
41	Costello (2011)	Type of study: Not original research
42	Cotter (2014)	Scope: not focus on weight management/obesity prevention
43	Cowan (2011)	Reference type: thesis/dissertation (full-text mostly not available)
44	Cummiskey (2011)	Type of study: Not original research
45	de Jongh (2012)	Scope: not focus on weight management/obesity prevention
46	Delpier (2013)	Scope: not focus on weight management/obesity prevention
47	Denney-Wilson (2013)	Reference type: not full-text article
48	Dennison (2014)	Technology: not focus on mobile devices and/or Web 2.0/social media
49	Dobkin (2011)	Type of study: Not original research
50	Dobratz (2006)	Reference type: thesis/dissertation (full-text mostly not available)
51	Donaldson (2011)	Reference type: not full-text article
52	Donnelly (2007)	Technology: not focus on mobile devices and/or Web 2.0/social media
53	Dunton (2012)	Scope: not focus on weight management/obesity prevention
54	Dunton (2009)	Reference type: not full-text article
55	Dunton (2012)	Scope: not focus on weight management/obesity prevention
56	Dunton (2011)	Scope: not focus on weight management/obesity prevention
57	Durant (2014)	Scope: not focus on weight management/obesity prevention
58	El-Gayar (2013)	Scope: not focus on weight management/obesity prevention
59	Farmer (2005)	Scope: not focus on weight management/obesity prevention
60	Faruqi (2013)	Reference type: not full-text article
61	Ferrer-Roca (2004)	Scope: not focus on weight management/obesity prevention
62	Firoozi (2013)	Reference type: not full-text article
63	Fjeldsoe (2009)	Scope: not focus on weight management/obesity prevention
64	Fjeldsoe (2012)	Duplicate
65	Flitcroft (2006)	Reference type: not full-text article
66	Frimming (2011)	Scope: not focus on weight management/obesity prevention
67	Frood (2010)	Reference type: not full-text article
68	Funk (2011)	Technology: not focus on mobile devices and/or Web 2.0/social media
69	Garnett (2014)	Type of study: Not original research
70	Gill (2012)	Type of study: Not original research
71	Göbel (2012)	Reference type: not full-text article

ID	Study	Reason for exclusion
72	Goodyear-Smith (2013)	Technology: not focus on mobile devices and/or Web 2.0/social media
73	Greenlaw (2011)	Type of study: Not original research
74	Guillory (2014)	Reference type: thesis/dissertation (full-text mostly not available)
75	Hackman (2014)	Type of study: Not original research
76	Hagobian (2013)	Type of study: Not original research
77	Hijazi (2011)	Reference type: thesis/dissertation (full-text mostly not available)
78	Hilbert (2009)	Scope: not focus on weight management/obesity prevention
79	Holtz (2014)	Technology: not focus on mobile devices and/or Web 2.0/social media
80	Holzinger (2010)	Type of study: Not original research
81	Hongu (2014)	Type of study: Not original research
82	Hongu (2011)	Type of study: Not original research
83	Howard (2011)	Reference type: thesis/dissertation (full-text mostly not available)
84	Hughes (2010)	Reference type: thesis/dissertation (full-text mostly not available)
85	Hussein (2011)	Scope: not focus on weight management/obesity prevention
86	Hutcheson (2013)	Reference type: thesis/dissertation (full-text mostly not available)
87	Incel (2013)	Type of study: Not original research
88	Ino (2012)	Reference type: not full-text article
89	Jerome (2014)	Scope: not focus on weight management/obesity prevention
90	Jogova (2013)	Technology: not focus on mobile devices and/or Web 2.0/social media
91	Jones (2013)	Reference type: thesis/dissertation (full-text mostly not available)
92	Jung (2012)	Scope: not focus on weight management/obesity prevention
93	Kane (2010)	Technology: not focus on mobile devices and/or Web 2.0/social media
94	Kang (2014)	Technology: not focus on mobile devices and/or Web 2.0/social media
95	Kaplan (2013)	Type of study: Not original research
96	Kapp (2011)	Reference type: thesis/dissertation (full-text mostly not available)
97	Kaufman (2010)	Type of study: Not original research
98	Kaufman (2011)	Type of study: Not original research
99	Kaufman (2012)	Type of study: Not original research
100	Keeney (2012)	Reference type: not full-text article
101	Kelly (2013)	Reference type: not full-text article
102	Ketabdar (2010)	Reference type: not full-text article
103	Kikunaga (2007)	Duplicate
104	Kim (2013)	Technology: not focus on mobile devices and/or Web 2.0/social media
105	Kim (2008)	Scope: not focus on weight management/obesity prevention
106	King (2014)	Technology: not focus on mobile devices and/or Web 2.0/social media
107	King (2011)	Reference type: not full-text article
108	Knight (2013)	Reference type: not full-text article
109	Kouris (2010)	Scope: not focus on weight management/obesity prevention
110	Koyle (2013)	Reference type: thesis/dissertation (full-text mostly not available)
111	Larwin (2008)	Technology: not focus on mobile devices and/or Web 2.0/social

ID	Study	Reason for exclusion
		media
112	Leavy (2013)	Type of study: Not original research
113	Ledwidge (2013)	Scope: not focus on weight management/obesity prevention
114	Lee (2006)	Reference type: thesis/dissertation (full-text mostly not available)
115	Lee (2007)	Scope: not focus on weight management/obesity prevention
116	Lewis (2014)	Reference type: not full-text article
117	Li (2008)	Reference type: not full-text article
118	Liao (2014)	Technology: not focus on mobile devices and/or Web 2.0/social media
119	Lin (2012)	Technology: not focus on mobile devices and/or Web 2.0/social media
120	Love (2013)	Reference type: not full-text article
121	Lu (2009)	Reference type: thesis/dissertation (full-text mostly not available)
122	Maheshwari (2008)	Reference type: thesis/dissertation (full-text mostly not available)
123	Mark'sson (2006)	Reference type: thesis/dissertation (full-text mostly not available)
124	Mateo (2014)	Reference type: not full-text article
125	McAlexander (2011)	Scope: not focus on weight management/obesity prevention
126	McCallum (2010)	Reference type: thesis/dissertation (full-text mostly not available)
127	McDoniel (2010)	Technology: not focus on mobile devices and/or Web 2.0/social media
128	McGraa (2010)	Reference type: thesis/dissertation (full-text mostly not available)
129	McTigue (2011)	Reference type: not full-text article
130	Mears (2010)	Type of study: Not original research
131	Morgan (2012)	Reference type: not full-text article
132	Morgan (2010)	Technology: not focus on mobile devices and/or Web 2.0/social media
133	Morgan (2011)	Technology: not focus on mobile devices and/or Web 2.0/social media
134	Mori (2011)	Scope: not focus on weight management/obesity prevention
135	Mosa (2012)	Scope: not focus on weight management/obesity prevention
136	Murtagh (2010)	Scope: not focus on weight management/obesity prevention
137	Nes (2012)	Scope: not focus on weight management/obesity prevention
138	Neve (2012)	Reference type: not full-text article
139	Newell (2012)	Scope: not focus on weight management/obesity prevention
140	Nguyen (2011)	Reference type: thesis/dissertation (full-text mostly not available)
141	Nguyen (2014)	Scope: not focus on weight management/obesity prevention
142	Norris-Ellis (2012)	Reference type: thesis/dissertation (full-text mostly not available)
143	O'Brien (2013)	Reference type: thesis/dissertation (full-text mostly not available)
144	Olmen (2013)	Scope: not focus on weight management/obesity prevention
145	Oloritun (2013)	Scope: not focus on weight management/obesity prevention
146	Ormseth (2011)	Reference type: thesis/dissertation (full-text mostly not available)
147	Orsama (2013)	Scope: not focus on weight management/obesity prevention
148	Owens (2009)	Scope: not focus on weight management/obesity prevention
149	Po'e (2013)	Technology: not focus on mobile devices and/or Web 2.0/social media
150	Postrach (2012)	Reference type: not full-text article
151	Powers (2008)	Scope: not focus on weight management/obesity prevention

ID	Study	Reason for exclusion
152	Richardson (2010)	Technology: not focus on mobile devices and/or Web 2.0/social media
153	Riva (2011)	Scope: not focus on weight management/obesity prevention
154	Robertson (2007)	Scope: not focus on weight management/obesity prevention
155	Rollins (2014)	Reference type: thesis/dissertation (full-text mostly not available)
156	Rossi (2013)	Scope: not focus on weight management/obesity prevention
157	Rotheram-Borus (2012)	Scope: not focus on weight management/obesity prevention
158	Royall (2011)	Reference type: not full-text article
159	Rutanen (2014)	Scope: not focus on weight management/obesity prevention
160	Ruxton (2014)	Type of study: Not original research
161	Schap (2011)	Technology: not focus on mobile devices and/or Web 2.0/social media
162	Seely (2013)	Reference type: thesis/dissertation (full-text mostly not available)
163	Sevick (2012)	Scope: not focus on weight management/obesity prevention
164	Sevick (2008)	Scope: not focus on weight management/obesity prevention
165	Shaw (2012)	Reference type: thesis/dissertation (full-text mostly not available)
166	Shay (2008)	Type of study: Not original research
167	Shim (2011)	Reference type: not full-text article
168	Skrøvseth (2012)	Scope: not focus on weight management/obesity prevention
169	Skrøvseth (2012)	Scope: not focus on weight management/obesity prevention
170	Sneed (2012)	Reference type: thesis/dissertation (full-text mostly not available)
171	Snively (2011)	Reference type: thesis/dissertation (full-text mostly not available)
172	Spring (2013)	Type of study: Not original research
173	Steinberg (2013)	Technology: not focus on mobile devices and/or Web 2.0/social media
174	Stellefson (2013)	Scope: not focus on weight management/obesity prevention
175	Stephens (2014)	Reference type: not full-text article
176	Stephens (2011)	Type of study: Not original research
177	Stevens (2012)	Type of study: Not original research
178	Stuckey (2011)	Scope: not focus on weight management/obesity prevention
179	Stuckey (2011)	Reference type: not full-text article
180	Stuckey (2011)	Scope: not focus on weight management/obesity prevention
181	Sun (2014)	Technology: not focus on mobile devices and/or Web 2.0/social media
182	Talbot (2011)	Scope: not focus on weight management/obesity prevention
183	Tamura (2014)	Technology: not focus on mobile devices and/or Web 2.0/social media
184	Tani (2010)	Scope: not focus on weight management/obesity prevention
185	Tate (2008)	Type of study: Not original research
186	Tate (2013)	Type of study: Not original research
187	Tettey (2010)	Reference type: thesis/dissertation (full-text mostly not available)
188	Thomas (2014)	Type of study: Not original research
189	Thomas (2011)	Type of study: Not original research
190	Thompson-Felty (2014)	Reference type: thesis/dissertation (full-text mostly not available)
191	Trevorrow (2012)	Technology: not focus on mobile devices and/or Web 2.0/social

ID	Study	Reason for exclusion
		media
192	Tu (2011)	Reference type: thesis/dissertation (full-text mostly not available)
193	Turner-McGrievy (2010)	Reference type: thesis/dissertation (full-text mostly not available)
194	Uden (2008)	Type of study: Not original research
195	Vandelanotte (2012)	Technology: not focus on mobile devices and/or Web 2.0/social media
196	Vandelanotte (2011)	Technology: not focus on mobile devices and/or Web 2.0/social media
197	Verhoeven (2010)	Scope: not focus on weight management/obesity prevention
198	Vermeulen (2013)	Scope: not focus on weight management/obesity prevention
199	Vodopivec-Jamsek (2012)	Scope: not focus on weight management/obesity prevention
200	Vuong (2012)	Scope: not focus on weight management/obesity prevention
201	Wang (2010)	Reference type: thesis/dissertation (full-text mostly not available)
202	Weiler (2013)	Type of study: Not original research
203	Weineland (2012)	Technology: not focus on mobile devices and/or Web 2.0/social media
204	Wier (2009)	Technology: not focus on mobile devices and/or Web 2.0/social media
205	Winett (2011)	Technology: not focus on mobile devices and/or Web 2.0/social media
206	Woolford (2009)	Reference type: not full-text article
207	Yu (2012)	Scope: not focus on weight management/obesity prevention
208	Yumak (2013)	Technology: not focus on mobile devices and/or Web 2.0/social media
209	Zenk (2014)	Scope: not focus on weight management/obesity prevention
210	Zheng (2010)	Scope: not focus on weight management/obesity prevention
211	Zulman (2013)	Technology: not focus on mobile devices and/or Web 2.0/social media

References for Appendix 4

1. Boots weight loss programme rolled out. Pharmaceutical Journal. 2005 08 Oct;275(7370):431. PMID: 2005473567.
2. Abebe NA, Capozza KL, Des Jardins TR, Kulick DA, Rein AL, Schachter AA, et al. Considerations for Community-Based mHealth Initiatives: Insights From Three Beacon Communities. Journal of Medical Internet Research. 2013 Oct;15(10):96-110. PMID: WOS:000326501500006. doi: 10.2196/jmir.2803.
3. Abraham AA, Chow WC, So HK, Woo J, Chan SM, Nelson EAS. Feasibility of Using Cell Phone Reminders to Motivate Behaviour Change in Obese Adolescents in Hong Kong. Hong Kong Journal of Paediatrics. 2014;19 (3):197. PMID: 75007113.
4. Anderson EJ, Sylvia LG, Lynch M, Sonnenberg L, Lee H, Nathan DM. Comparison of Energy Assessment Methods in Overweight Individuals. Journal of the Academy of Nutrition & Dietetics. 2014;114(2):273-8. PMID: 2012426497. doi: 10.1016/j.jand.2013.07.008.
5. Anton SD, LeBlanc E, Allen HR, Karabetian C, Sacks F, Bray G, et al. Use of a computerized tracking system to monitor and provide feedback on dietary goals for calorie-restricted diets: the POUNDS LOST study. Journal of Diabetes Science and Technology. 2012;6(5):1216-25. PMID: CN-00853688.
6. Arbogast S. 140 characters to skinny : social support provided by commercial weight-loss programs via twitter [Thesis (M.A.)]. USA: Clemson University, 2013; 2013.

7. Arora S, Peters AL, Agy C, Menchine M. A mobile health intervention for inner city patients with poorly controlled diabetes: proof-of-concept of the TExT-MED program. Diabetes Technol Ther. 2012 Jun;14(6):492-6. PMID: 22524591. doi: http://dx.doi.org/10.1089/dia.2011.0252.
8. Arsand E, Frøisland DH, Skrøvseth SO, Chomutare T, Tatara N, Hartvigsen G, et al. Mobile health applications to assist patients with diabetes: lessons learned and design implications. Journal of Diabetes Science and Technology. 2012 Sep;6(5):1197-206. PMID: 23063047.
9. Årsand E, Tatara N, Ostengen G, Hartvigsen G. Mobile phone-based self-management tools for type 2 diabetes: the few touch application. Journal of Diabetes Science and Technology. 2010 Mar;4(2):328-36. PMID: 20307393.
10. Arsand E, Tufano JT, Ralston JD, Hjortdahl P. Designing mobile dietary management support technologies for people with diabetes. J Telemed Telecare. 2008;14(7):329-32. PMID: 18852310. doi: http://dx.doi.org/10.1258/jtt.2008.007001.
11. Arvidsson D, Slinde F, Larsson S, Hulthen L. Energy cost of physical activities in children: validation of SenseWear Armband. Medicine and Science in Sports and Exercise. 2007 Nov;39(11):2076-84. PMID: 17986918.
12. Bakken SH, Jia; Chen, Elizabeth S.; Jeeyae, Choi; John, Rita Marie; Nam-Ju, Lee; Mendonca, Eneida; Roberts, William Dan; Velez, Olivia; Currie, Leanne M. The Effect of a Mobile Health Decision Support System on Diagnosis and Management of Obesity, Tobacco Use, and Depression in Adults and Children. Journal for Nurse Practitioners. 2014;10(10):774-80. PMID: 2012809185. doi: 10.1016/j.nurpra.2014.07.017.
13. Baranowski T, Frankel L. Let's get technical! Gaming and technology for weight control and health promotion in children. Child. 2012 Feb;8(1):34-7. PMID: 22799477. doi: http://dx.doi.org/10.1089/chi.2011.0103.
14. Barragan NC, Noller AJ, Robles B, Gase LN, Leighs MS, Bogert S, et al. The “Sugar Pack” Health Marketing Campaign in Los Angeles County, 2011-2012. Health Promotion Practice. 2014;15(2):208-16. PMID: 2012475967. doi: 10.1177/1524839913507280.
15. Baulch J, Chester A, Brennan L. Treatment alternatives for overweight and obesity: The role of online interventions. Behaviour Change. 2008;25(1):1-14. PMID: 2008335956. doi: http://dx.doi.org/10.1375/bech.25.1.1.
16. Berke EM, Choudhury T, Ali S, Rabbi M. Objective measurement of sociability and activity: Mobile sensing in the community. Annals of Family Medicine. 2011;9(4):344-50. PMID: 2011-16969-007.
17. Biddle SJHP, I.; Pearson, N. Interventions designed to reduce sedentary behaviours in young people: a review of reviews. BJSM online. 2014;48(3):182-6. PMID: 20143071334. doi: http://dx.doi.org/10.1136/bjsports-2013-093078.
18. Binks M, Van Mierlo TT. The relationships of the psychological influence of food and perceived barriers to lifestyle change to body mass index and to utilization of online weight loss tools. Canadian Journal of Diabetes. 2011 May;35 (2):209. PMID: 70446333.
19. Blanson Henkemans OA, Rogers WA, Dumay AMC. Personal characteristics and the law of attrition in randomized controlled trials of eHealth services for self-care. Gerontechnology. 2011;10(3):157-68. PMID: 2011473149.
20. Bond DST, Graham J.; Ryder, Beth A.; Vithiananthan, Sivamainthan; Pohl, Dieter; Wing, Rena. Ecological Momentary Assessment of the Relationship between Intention and Physical Activity Behavior in Bariatric Surgery Patients. International Journal of Behavioral Medicine. 2013 03//;20(1):82-7. PMID: 85631305. doi: 10.1007/s12529-011-9214-1.
21. Breland JY, Yeh VM, Yu J. Adherence to evidence-based guidelines among diabetes self-management apps. Translational Behavioral Medicine. 2013 September;3(3):277-86. PMID: 2013576821. doi: http://dx.doi.org/10.1007/s13142-013-0205-4.
22. Brindal EH, Gilly; Freyne, Jill; Coombe, Mac; Berkovsky, Shlomo; Noakes, Manny. Design and pilot results of a mobile phone weight-loss application for women starting a meal replacement programme. . J Telemed Telecare. 2013;19(3):166-74. doi: http://dx.doi.org/10.1177/1357633X13479702.
23. Brooke MJ, Thompson BM. Food and Drug Administration regulation of diabetes-related mHealth technologies. Journal of Diabetes Science and Technology. 2013;7(2):296-301. PMID: 23566984.
24. Brox E, Luque LF, Evertsen GJ, Hernandez JEG, editors. Exergames for elderly: Social exergames to persuade seniors to increase physical activity. 5th International Conference on Pervasive Computing Technologies for Healthcare (PervasiveHealth), 2011; 2011 23-26 May 2011; Dublin, Ireland.

25. Burner E, Menchine M, Taylor E, Arora S. Gender differences in diabetes self-management: a mixed-methods analysis of a mobile health intervention for inner-city Latino patients. Journal of Diabetes Science and Technology. 2013;7(1):111-8. PMID: 23439166.
26. Butler R. The technology of obesity: prevention and treatment. In: Tsichlia G, Johnstone A, editors. Fat matters: from sociology to science. Cumbria, UK: M&K Update Ltd 2010. p. 39-49.
27. Carnie A, Lin J, Aicher B, Leon B, Courville AB, Sebring NG, et al. Randomized trial of nutrition education added to internet-based information and exercise at the work place for weight loss in a racially diverse population of overweight women. Nutrition and Diabetes. 2013 December;3(DECEMBER):1-7. PMID: 2014046345. doi: http://dx.doi.org/10.1038/nutd.2013.39.
28. Carrard I, Crépin C, Rouget P, Lam T, Van Der Linden M, Golay A. Acceptance and efficacy of a guided Internet self-help treatment program for obese patients with binge eating disorder. Clinical Practice and Epidemiology in Mental Health. 2011;7:8-18. PMID: 2012223606. doi: http://dx.doi.org/10.2174/1745017901107010008.
29. Carter MC. Development, validation and use of a smartphone application for weight loss [Thesis (Ph.D.)]. Leeds, UK: University of Leeds (School of Food Science and Nutrition), 2013; 2013.
30. Castelnuovo G, Manzoni GM, Pietrabissa G, Corti S, Giusti EM, Molinari E, et al. Obesity and outpatient rehabilitation using mobile technologies: the potential mHealth approach. Frontiers in Psychology. 2014 Jun;5:7. PMID: WOS:000337847300001. doi: 10.3389/fpsyg.2014.00559.
31. Castelnuovo G, Manzoni GM, Villa V, Cesa GL, Pietrabissa G, Molinari E. The STRATOB study: design of a randomized controlled clinical trial of Cognitive Behavioral Therapy and Brief Strategic Therapy with telecare in patients with obesity and binge-eating disorder referred to residential nutritional rehabilitation. Trials. 2011;12:114. PMID: CN-00800149. doi: 10.1186/1745-6215-12-114.
32. Cavallo DN. Using online social network technology to increase social support for physical activity: The internet support for healthy associations promoting exercise (INSHAPE) study [Dissertation Abstract]. Chapel Hill, USA: University of North Carolina, Chapel Hill; 2012.
33. Centis E, Marzocchi R, Suppini A, Dalle Grave R, Villanova N, Hickman IJ, et al. The role of lifestyle change in the prevention and treatment of NAFLD. Curr Pharm Des. 2013;19(29):5270-9. PMID: 23394095.
34. Choi J, Fukuoka Y, Lee KA. Development of a Mobile Phone-Based Physical Activity Program in Pregnant Women. Communicating Nursing Research. 2013;46:386-. PMID: 2012376743.
35. Chomutare T, Fernandez-Luque L, Arsand E, Hartvigsen G. Features of mobile diabetes applications: review of the literature and analysis of current applications compared against evidence-based guidelines. Journal of Medical Internet Research. 2011;13(3):e65. PMID: 21979293. doi: http://dx.doi.org/10.2196/jmir.1874.
36. Chuah M, Jakes G, Qin Z, editors. WiFiTreasureHunt: A Mobile Social Application for Staying Active Physically. 14th International Conference on Ubiquitous Computing (Ubicomp 2012) 2012 2012; Pittsburgh, PA, USA. New York, NY, USA: ACM.
37. Clark A, Kovarik S, Voigt M, Hayes J. Using the MyPyramid.gov website as a tool for diabetes self-management education. Diabetes Spectrum. 2006 Spring;19(2):122-6. PMID: 2006606008. doi: http://dx.doi.org/10.2337/diaspect.19.2.122.
38. Clary JM, editor. "Mississippi in Motion" and "Families Food and Fitness Community of Practice" virtual education site: Improving the health of Americans. 2nd International Conference on Education and New Learning Technologies (EDULEARN); 2010; Barcelona, Spain: Iated-Int Assoc Technology Education a& Development.
39. Cleland VB, K. What might work? Exploring the perceived feasibility of strategies to promote physical activity among women living in socioeconomically disadvantaged neighbourhoods. Health Educ Res. 2013 Apr;28(2):205-19. PMID: 22987863. doi: http://dx.doi.org/10.1093/her/cys097.
40. Corsino LL, P. H.; Batch, B. C.; Intille, S.; Grambow, S. C.; Bosworth, H. B.; Bennett, G. G.; Tyson, C.; Svetkey, L. P.; Voils, C. I. Recruiting young adults into a weight loss trial: report of protocol development and recruitment results. Contemporary Clinical Trials. 2013 Jul;35(2):1-7. PMID: 23591327. doi: http://dx.doi.org/10.1016/j.cct.2013.04.002.
41. Costello RB, Loria CM, Lau J, Sacks FM, Yetley EA. Update on Nutrition Research Methodologies. Nutrition Today. 2011;46(3):116-22. PMID: 2011185749. doi: 10.1097/NT.0b013e318212d4b9.
42. Cotter AP, Durant N, Agne AA, Cherrington AL. Internet interventions to support lifestyle modification for diabetes management: A systematic review of the evidence. Journal of Diabetes and

Its Complications. 2014 Mar-Apr;28(2):243-51. PMID: WOS:000333542900025. doi: 10.1016/j.jdiacomp.2013.07.003.
43. Cowan DK. Evaluation of nutritional quality through a counselor administered weight loss program utilizing a smart phone app [Thesis]. Arizona, USA: Arizona State University; 2011.
44. Cummiskey M. There's an App for that: Smartphone Use in Health and Physical Education. Journal of Physical Education, Recreation & Dance (JOPERD). 2011 10/01/;82(8):24-9. PMID: EJ965312.
45. de Jongh T, Gurol-Urganci I, Vodopivec-Jamsek V, Car J, Atun R. Mobile phone messaging for facilitating self-management of long-term illnesses. Cochrane Database of Systematic Reviews. 2012 (12). PMID: 2011917381.
46. Delpier TG, Sheri; Wedin, Bitsy M. Decreasing sugar-sweetened beverage consumption in the rural adolescent population. Journal of Pediatric Health Care. 2013;27(6):470-8. doi: http://dx.doi.org/10.1016/j.pedhc.2012.07.002.
47. Denney-Wilson E, Campbell KJ, Laws R, Russell G, Lynch J, Ball K, et al. Assisting parents to prevent obesity in their young children: The COMPaRE-PHC early childhood research program. Obesity Research and Clinical Practice. 2013 December;7:e98. PMID: 71423669. doi: http://dx.doi.org/10.1016/j.orcp.2013.12.678.
48. Dennison L, Morrison L, Lloyd S, Phillips D, Stuart B, Williams S, et al. Does brief telephone support improve engagement with a Web-based weight management intervention? Journal of Medical Internet Research. 2014;16(3):130-44. doi: http://dx.doi.org/10.2196/jmir.3199.
49. Dobkin BH, Dorsch A. The promise of mHealth: Daily activity monitoring and outcome assessments by wearable sensors. Neurorehabilitation and Neural Repair. 2011;25(9):788-98. doi: http://dx.doi.org/10.1177/1545968311425908.
50. Dobratz JR. Validation of prediction equations and hand-held indirect calorimetry for resting energy expenditure assessment in extremely obese women [Thesis]. USA: University of Minnesota; 2006.
51. Donaldson EL, Fallows S. A text message-based weight management intervention for overweight adults. Journal of Human Nutrition & Dietetics. 2011;24(4):385-6. PMID: 2011218610. doi: 10.1111/j.1365-277X.2011.01177.
52. Donnelly JE, Smith BK, Dunn L, Mayo MM, Jacobsen DJ, Stewart EE, et al. Comparison of a phone vs clinic approach to achieve 10% weight loss. International journal of obesity (2005). 2007;31(8):1270-6. PMID: CN-00617758. doi: 10.1038/sj.ijo.0803568.
53. Dunton GF, Intille S, Beaudin J, Pentz MA. Pilot Test of a Real-Time Data Capture Protocol to Assess Children's Exposure to and Experience of Physical Activity Contexts Using Mobile Phones. Obesity. 2009 Nov;17:S150-S1. PMID: WOS:000271237800369.
54. Dunton GF, Intille S, Wolch J, Pentz MA. Investigating the impact of a smart growth community on the contexts of children's physical activity using Ecological Momentary Assessment. Health Place. 2012 Jan;18(1):76-84. PMID: 22243909. doi: http://dx.doi.org/10.1016/j.healthplace.2011.07.007.
55. Dunton GF, Kawabata K, Intille S, Wolch J, Pentz, MA. Assessing the social and physical contexts of children's leisure-time physical activity: An ecological momentary assessment study. . American Journal of Health Promotion. 2012;26(3):135-42. doi: http://dx.doi.org/10.4278/ajhp.100211-QUAN-43.
56. Dunton GF, Liao Y, Intille S, Wolch J., Pentz MA. Physical and social contextual influences on children's leisure-time physical activity: an ecological momentary assessment study. J Phys Act Health. 2011 Jan;8 Suppl 1:S103-8. PMID: 21350250.
57. Durant NHJ, Rodney P.; Cherrington, Andrea; Cuffee, Yendelela; Knight, BernNadette; Lewis, Dwight, Jr.; Allison, Jeroan J. Recommendations for a culturally relevant Internet-based tool to promote physical activity among overweight young African American women, Alabama, 2010-2011. Preventing Chronic Disease: Public Health Research, Practice, and Policy. 2014;11:130169. doi: http://dx.doi.org/10.5888/pcd11.130169.
58. El-Gayar O, Timsina P, Nawar N, Eid W. Mobile applications for diabetes self-management: status and potential. Journal of Diabetes Science and Technology. 2013;7(1):247-62. PMID: 23439183.
59. Farmer A, Gibson O, Hayton P, Bryden K, Dudley C, Neil A, et al. A real-time, mobile phone-based telemedicine system to support young adults with type 1 diabetes. Inform Prim Care. 2005;13(3):171-7. PMID: 16259856.
60. Faruqi N, Joshi C, Dennis S, Lloyd J, Taggart J, Spooner C, et al. What health literacy interventions are effective in the primary healthcare settings in weight loss management - A systematic review.

Obesity Research and Clinical Practice. 2013 December;7:e99. PMID: 71423670. doi: http://dx.doi.org/10.1016/j.orcp.2013.12.679.
61. Ferrer-Roca O, Cardenas A, Diaz-Cardama A, Pulido P. Mobile phone text messaging in the management of diabetes. J Telemed Telecare. 2004;10(5):282-5. PMID: 15494086.
62. Firoozi M, Gheed Rahmat A. Virtual social network for management of obesity in children and adolescents. Iranian Journal of Pediatrics. 2013 October;23:S9-S10. PMID: 71257046.
63. Fjeldsoe BS, Marshall AL, Miller YD. Behavior Change Interventions Delivered by Mobile Telephone Short-Message Service. American Journal of Preventive Medicine. 2009 Feb;36(2):165-73. PMID: WOS:000262679600012. doi: 10.1016/j.amepre.2008.09.040.
64. Fjeldsoe BSM, Y. D.; O'Brien, J. L.; Marshall, A. L. Iterative development of MobileMums: a physical activity intervention for women with young children. International Journal of Behavioral Nutrition & Physical Activity. 2012;9:151. PMID: 23256730. doi: http://dx.doi.org/10.1186/1479-5868-9-151.
65. Flitcroft I. Calorie transfer: A new approach to the global nutrition crisis. MedGenMed Medscape General Medicine. 2006;8(1). PMID: 2006102435.
66. Frimming RE, Polsgrove MJ, Bower GG. Evaluation of a Health and Fitness Social Media Experience. American Journal of Health Education. 2011;42(4):222-7. PMID: 2011226110.
67. Frood A. Technology: A flavour of the future. Nature. 2010 12/24/12/23/2010 Supplement;468(7327):S21-S2. PMID: 56656268. doi: 10.1038/468S21a.
68. Funk KL, Stevens VJ, Bauck A, Brantley PJ, Hornbrook M, Jerome GJ, et al. Development and implementation of a tailored self-assessment tool in an internet-based weight loss maintenance program. Clinical Practice and Epidemiology in Mental Health. 2011;7:67-73. PMID: 2012223599. doi: http://dx.doi.org/10.2174/1745017901107010067.
69. Garnett BR, Buelow R, Franko DL, Becker C, Rodgers RF, Austin SB. The Importance of Campaign Saliency as a Predictor of Attitude and Behavior Change: A Pilot Evaluation of Social Marketing Campaign Fat Talk Free Week. Health Communication. 2014;29(10):984-95. PMID: 96222983. doi: 10.1080/10410236.2013.827613.
70. Gill TP, Boylan S. Public Health Messages: Why Are They Ineffective and What Can Be Done? Current Obesity Reports. 2012 March;1(1):50-8. PMID: 2012706748. doi: http://dx.doi.org/10.1007/s13679-011-0003-6.
71. Göbel S, editor. Cloud-based Games for Health: Serious Games and Social Media As Multimedia Technologies for Healthcare. CMBAS-EH '12 - ACM Multimedia; 2012 2012; Nara, Japan. New York, NY, USA: ACM.
72. Goodyear-Smith F, Warren J, Elley CR. The eCHAT program to facilitate healthy changes in New Zealand primary care. J Am Board Fam Med. 2013 Mar-Apr;26(2):177-82. PMID: 23471931. doi: http://dx.doi.org/10.3122/jabfm.2013.02.120221.
73. Greenlaw R, editor. Wellness, Social Networking, and Algorithms. International Computer Science and Engineering Conference (ICSEC 2010) 2011 Mar; Chiang Mai, Thailand.
74. Guillory JE. Social support, psychosocial resources and eating: Using social media to encourage healthy eating [Dissertation Abstract]. USA: Cornell University; 2014.
75. Hackman CL, Knowlden AP. Theory of reasoned action and theory of planned behavior-based dietary interventions in adolescents and young adults: A systematic review. Adolescent Health, Medicine and Therapeutics. 2014 06 Jun;5:101-14. PMID: 2014427713. doi: http://dx.doi.org/10.2147/AHMT.S56207.
76. Hagobian TA, Phelan S. Lifestyle interventions to reduce obesity and diabetes. American Journal of Lifestyle Medicine. 2013;7(2):84-98. PMID: 20133133109. doi: http://dx.doi.org/10.1177/1559827612449600.
77. Hijazi RR. Exploring the use of smart phones to influence healthy behaviors [Dissertation Abstract]. Minneapolis, Minnesota, USA: Walden University; 2011.
78. Hilbert A, Rief W, Tuschen-Caffier B, de Zwaan M, Czaja J. Loss of control eating and psychological maintenance in children: an ecological momentary assessment study. Behav Res Ther. 2009 Jan;47(1):26-33. PMID: 19010458. doi: http://dx.doi.org/10.1016/j.brat.2008.10.003.
79. Holtz B, Krein SL, Bentley DR, Hughes ME, Giardino ND, Richardson CR. Comparison of Veteran experiences of low-cost, home-based diet and exercise interventions. Journal of Rehabilitation Research and Development. 2014;51(1):149-60. PMID: WOS:000334952600013. doi: 10.1682/jrrd.2013.04.0088.

80. Holzinger A, Dorner S, Fodinger M, Valdez AC, Ziefle M, editors. Chances of Increasing Youth Health Awareness through Mobile Wellness Applications. 6th Symposium of the Workgroup Human-Computer Interaction and Usability Engineering 2010; Klagenfurt, Austria: Springer-Verlag Berlin.
81. Hongu N, Going SB, Orr BJ, Merchant NC, Hingle MD, Roe DJ, et al. Mobile Technologies for Promoting Health and Physical Activity. ACSM's Health and Fitness Journal. 2014;18(4):8-15. PMID: 2012626695. doi: 10.1249/FIT.0000000000000050.
82. Hongu N, Hingle MD, Merchant NC, Orr BJ, Going SB, Mosqueda MI, et al. Dietary Assessment Tools Using Mobile Technology. Topics in Clinical Nutrition. 2011;26(4):300-11. PMID: 2011383736. doi: 10.1097/TIN.0b013e3182379525.
83. Howard AK. Feedforward: A mobile design strategy that supports emotive learning for preventive health practices and enduring lifestyle change [Dissertation Abstract]. Raleigh, North Carolina, USA: North Carolina State University; 2011.
84. Hughes CD. The BALANCE study (Bioengineering Approaches to Lifestyle Activity and Nutrition Continuous Engagement) : using the design-feedback iterative cycle to improve methods for measuring energy intake via electronic food diary [Thesis]. USA: University of Washington; 2010.
85. Hussein WI, Hasan K, Jaradat AA. Effectiveness of mobile phone short message service on diabetes mellitus management; the SMS-DM study. Diabetes Res Clin Pract. 2011 Oct;94(1):e24-6. PMID: 21840079. doi: http://dx.doi.org/10.1016/j.diabres.2011.07.025.
86. Hutcheson TD. Using mobile technology to impact fruit and vegetable consumption in low-income youth [Dissertation Abstract]. USA: University of Kansas; 2013.

87. Incel OD, Kose M, Ersoy C. A Review and Taxonomy of Activity Recognition on Mobile Phones. BioNanoScience. 2013 June;3(2):145-71. PMID: 2013327784. doi: http://dx.doi.org/10.1007/s12668-013-0088-3.
88. Ino H, Taira K, Sakamoto Y. The impact of the application "iNST" development for nutrition support for patients in Japan. Clinical Nutrition, Supplement. 2012 September;7 (1):116. PMID: 70903675. doi: http://dx.doi.org/10.1016/S1744-1161%2812%2970281-8.
89. Jerome GJ, Dalcin A, Coughlin JW, Fitzpatrick S, Wang N-Y, Durkin N, et al. Longitudinal accuracy of Web-based self-reported weights: Results from the Hopkins POWER trial. Journal of Medical Internet Research. 2014;16(7):85-92.
90. Jogova M, Song JES, Campbell AC, Warbuton D, Warshawski T, Chanoine JP. Process Evaluation of the Living Green, Healthy and Thrifty (LiGHT) Web-Based Child Obesity Management Program: Combining Health Promotion with Ecology and Economy. Canadian Journal of Diabetes. 2013 April;37(2):72-81. PMID: 2013271650. doi: http://dx.doi.org/10.1016/j.jcjd.2013.03.359.
91. Jones EM. Electronic apps for food and appetite monitoring: Acceptability and reactive effects in women with eating and weight concerns [Dissertation Abstract]. Atlanta, Georgia, USA: Emory University; 2013.
92. Jung H, Lee B, Lee JE, Kwon YH, Song H. Efficacy of a programme for workers with metabolic syndrome based on an e-health system in the workplace: a pilot study. J Telemed Telecare. 2012 Sep;18(6):339-43. PMID: 22912490. doi: http://dx.doi.org/10.1258/jtt.2012.120318.
93. Kane NA, Simmons MC, John D, Thompson DL, Basset DR. Validity of the Nike + device during walking and running [corrected] [published erratum appears in INT J SPORTS MED 2010 Feb;31(2):105]. International Journal of Sports Medicine. 2010;31(2):101-5. PMID: 2010573403. doi: 10.1055/s-0029-1242810.
94. Kang JS, Kang HS, Jeong Y. A web-based health promotion program for patients with metabolic syndrome. Asian Nursing Research. 2014 March;8(1):82-9. PMID: 2014226135. doi: http://dx.doi.org/10.1016/j.anr.2014.03.002.
95. Kaplan RM, Stone AA. Bringing the Laboratory and Clinic to the Community: Mobile Technologies for Health Promotion and Disease Prevention. Annual Review of Psychology. 2013;64(1):471-98. PMID: 2011804553. doi: 10.1146/annurev-psych-113011-143736.
96. Kapp CTL. The use of a personal fitness device in conjunction with online social networking as an effective delivery system for occupational therapy community practice to enhance an individual's personal occupation and self-efficacy [Thesis]. USA: East Carolina University; 2011.
97. Kaufman N. Internet and information technology use in treatment of diabetes. International Journal of Clinical Practice (Supplement). 2010;64:41-6. PMID: 2010742089. doi: 10.1111/j.1742-1241.2009.02277.x.

98. Kaufman N. Information technology in the service of diabetes prevention and treatment. Int J Clin Pract Suppl. 2011 Feb;Supplement.(170):47-54. PMID: 21323812. doi: http://dx.doi.org/10.1111/j.1742-1241.2010.02578.x.
99. Kaufman N. Using Health Information Technology to Prevent and Treat Diabetes. International Journal of Clinical Practice (Supplement). 2012;66:40-8. PMID: 2011458962. doi: 10.1111/j.1742-1241.2011.02853.x.
100. Keeney M, Yeh M, Landman R, Leung M, Navder K. Exploring the Use of an iPhone App: A Novel Approach to Dietary Assessment. Journal of the Academy of Nutrition & Dietetics. 2012;112:A22-A. PMID: 2011717357. doi: 10.1016/j.jand.2012.06.075.
101. Kelly T. A resource guide for web-based physical activity logs. Bariatric Surgical Patient Care. 2013 01 Jun;8(2):83-4. PMID: 2013396307. doi: http://dx.doi.org/10.1089/bari.2013.9986.
102. Ketabdar H, Lyra M, editors. ActivityMonitor: Assisted Life Using Mobile Phones. Proceedings of the 14th ACM International Conference on Intelligent User Interfaces; 2010; Hong Kong, China: Assoc Computing Machinery.
103. Kikunaga S, Tin T, Ishibashi G, Wang D-H, Kira S. The Application of a Handheld Personal Digital Assistant with Camera and Mobile Phone Card (Wellnavi) to the General Population in a Dietary Survey. Journal of Nutritional Science and Vitaminology. 2007 2007;53(2):109-16. doi: 10.3177/jnsv.53.109.
104. Kim HYP, H. A.; Min, Y. H.; Jeon, E. Development of an obesity management ontology based on the nursing process for the mobile-device domain. Journal of Medical Internet Research. 2013;15(6):e130. PMID: 23811542. doi: http://dx.doi.org/10.2196/jmir.2512.
105. Kim SIK, H. S. Effectiveness of mobile and internet intervention in patients with obese type 2 diabetes. International Journal of Medical Informatics. 2008;77(6):399-404. PMID: CN-00637001. doi: 10.1016/j.ijmedinf.2007.07.006.
106. King AC, Hekler EB, Castro CM, Buman MP, Marcus BH, Napolitano MA, et al. Exercise advice by humans versus computers: Maintenance effects at 18 months. Health Psychology. 2014;33(2):192-6. PMID: Cn-00983932. doi: http://dx.doi.org/10.1037/a0030646.
107. King NA, Loewenich F, Hills AP, Wood RE, Byrne NM. An energy balance APP: A self-management tool for monitoring food intake, appetite and body weight. Obesity Research and Clinical Practice. 2011 October;5:S57-S8. PMID: 70547526. doi: http://dx.doi.org/10.1016/j.orcp.2011.08.013.
108. Knight E, Stuckey MI, Petrella RJ. Physical activity prescription and remote self-monitoring technologies: Can we reduce cardiovascular disease risk? Canadian Journal of Diabetes. 2013 October;37:S48. PMID: 71210571. doi: http://dx.doi.org/10.1016/j.jcjd.2013.08.142.
109. Kouris I, Mougiakakou S, Scarnato L, Iliopoulou D, Diem P, Vazeou A, et al. Mobile phone technologies and advanced data analysis towards the enhancement of diabetes self-management. Int. 2010;5(4):386-402. PMID: 21041177.
110. Koyle AE. The value of infusing self-efficacy theory with smartphone technology to sustain walking for exercise in a worksite population [Dissertation Abstract]. USA: University of Utah; 2013.
111. Larwin KH, Larwin DA. Decreasing excessive media usage while increasing physical activity: A single-subject research study. Behavior Modification. 2008;32(6):938-56. doi: http://dx.doi.org/10.1177/0145445508319668.
112. Leavy JE, Rosenberg M, Barnes R, Bauman A, Bull FC. Would you Find Thirty online? Website use in a Western Australian physical activity campaign. Health Promot J Aust. 2013 Aug;24(2):118-25. PMID: 24168738. doi: http://dx.doi.org/10.1071/HE12916.
113. Ledwidge MTOH, R.; Lalor, L.; Travers, B.; Edwards, N.; Kelly, D.; Voon, V.; McDonald, K. M. Can individualized weight monitoring using the HeartPhone algorithm improve sensitivity for clinical deterioration of heart failure? Eur J Heart Fail. 2013 Apr;15(4):447-55. PMID: 23204211. doi: http://dx.doi.org/10.1093/eurjhf/hfs186.
114. Lee N-J. Development and evaluation of a prototype personal digital assistant-decision support system for the management of obesity. USA: Columbia University; 2006.
115. Lee N-J, Bakken S. Development and evaluation of a prototype personal digital assistant-decision support system for the management of obesity. International Journal of Medical Informatics. 2007 2007;76S:S281-S92. doi: 10.1016/j.ijmedinf.2007.05.009.
116. Lewis K. Weight Watchers for the Facebook Era--How Does It Compare to the Do-It-Yourself Approach? Journal of Clinical Outcomes Management. 2014;21(3):102-5. PMID: 2012515630.

117. Li SY, Zhu WM, Park SH, Lin J. Monitoring Glucose and Physical Activity Using Mobile Phone: A Proposed System. Medicine and Science in Sports and Exercise. 2008 May;40(5):S427-S8. PMID: WOS:000208070903400. doi: 10.1249/01.mss.0000322821.83961.1d.
118. Liao YI, Stephen; Wolch, Jennifer; Pentz, Mary Ann; Dunton, Genevieve Fridlund. Understanding the physical and social contexts of children's non-school sedentary behavior: An ecological momentary assessment study. J Phys Act Health. 2014;11(3):588-613.
119. Lin LP, Wan Dali PE. The impact of nutrition education interventions on the dietary habits of college students in developed nations: A brief review. Malaysian Journal of Medical Sciences. 2012 January-March;19(1):4-14. PMID: 2012045302.
120. Love P, Romanus A, Whelan J, Smith E, Bolton K, Pettman T, et al. Knowledge translation and exchange: Enhancing communication for best practice in obesity prevention. Obesity Research and Clinical Practice. 2013 December;7:e91. PMID: 71423656. doi: http://dx.doi.org/10.1016/j.orcp.2013.12.665.
121. Lu AS. An experimental test of the effectiveness of customized narrative and non-narrative health blogs [Dissertation Abstract]. Chapel Hill, USA: University of North Carolina at Chapel Hill; 2009.
122. Maheshwari M. A model for computer-tailored motivational message generation for a healthy lifestyle management information system [Dissertation Abstract]. Claremont, California, USA: University of Claremont; 2008.
123. Mark˙sson GG. Internet- and mobile phone programs as tools to change behaviour and improve weight control in the treatment for obesity, overweight and type 2 diabetes : a review of studies [M.Sc. thesis]. Denmark: Royal Veterinary and Agricultural University; 2006.
124. Mateo KF, Jay M. Access to a behavioral weight loss website with or without group sessions increased weight loss in statewide campaign. Journal of Clinical Outcomes Management. 2014;21(8):345-8.
125. McAlexander KM, Sahnoune I, Alastuey L, Bode S, Lee RE. Using virtual and real, immersive technologies to enhance learning in obesity studies. Journal of CyberTherapy and Rehabilitation (JCR). 2011;4(4):455-60. PMID: 2012573471.
126. McCallum KG. Facebook me the utility of social networking sites in increasing social support for exercise programs [Report]: University of Texas at Austin; 2010.
127. McDoniel SO, Wolskee P, Shen J. Treating obesity with a novel hand-held device, computer software program, and Internet technology in primary care: the SMART motivational trial. Patient education and counseling. 2010;79(2):185-91. PMID: CN-00748848. doi: 10.1016/j.pec.2009.07.034.
128. McGraa KL. The effects of persuasive motivational text messaging on adherence to diet and exercise programs across different personality traits [Dissertation Abstract]. Santa Barbara, California, USA: Fielding Graduate University; 2010.
129. McTigue KM, Hess R. What role can the internet play in preventing diabetes? Diabetes Management. 2011 January;1(1):9-12. PMID: 2012164976. doi: http://dx.doi.org/10.2217/dmt.10.3.
130. Mears D. Technology in Physical Education Article #6 in a 6-Part Series: Physical Activity Monitoring: Gadgets and Uses. Strategies: A Journal for Physical and Sport Educators. 2010 2010;23(3):28-31. PMID: EJ900979. doi: 10.1080/08924562.2010.10590874.
131. Morgan P, Callister R, Collins C, Plotnikoff R, Young M, Berry N, et al. The SHED-IT Community Trial: A randomised controlled trial of Internet- and paper-based weight loss programs tailored for overweight and obese men. Obesity Research and Clinical Practice. 2012 October;6:30-1. PMID: 70899146. doi: http://dx.doi.org/10.1016/j.orcp.2012.08.062.
132. Morgan PJ, Collins CE, Plotnikoff RC, McElduff P, Burrows T, Warren JM, et al. The SHED-IT community trial study protocol: a randomised controlled trial of weight loss programs for overweight and obese men. BMC public health. 2010;10:701. PMID: Cn-00779443. doi: 10.1186/1471-2458-10-701.
133. Morgan PJ, Warren JM, Lubans DR, Collins CE, Callister R. Engaging men in weight loss: Experiences of men who participated in the male only SHED-IT pilot study. Obesity Research and Clinical Practice. 2011 July-September;5(3):e239-e48. PMID: 2011449768. doi: http://dx.doi.org/10.1016/j.orcp.2011.03.002.
134. Mori DL, Silberbogen AK, Collins AE, Ulloa EW, Brown KL, Niles BL. Promoting physical activity in individuals with diabetes: Telehealth approaches. Diabetes Spectrum. 2011 Summer;24(3):127-35. PMID: 2012021608. doi: http://dx.doi.org/10.2337/diaspect.24.3.127.

135. Mosa AS, Yoo I, Sheets L. A systematic review of healthcare applications for smartphones. BMC Med Inf Decis Mak. 2012;12:67. PMID: 22781312. doi: http://dx.doi.org/10.1186/1472-6947-12-67.
136. Murtagh EM, Murphy MH, Boone-Heinonen J. Walking: the first steps in cardiovascular disease prevention. Current Opinion in Cardiology. 2010;25(5):490-6. PMID: 2010754621. doi: 10.1097/HCO.0b013e32833ce972.
137. Nes AA, van Dulmen S, Eide E, Finset A, Kristjansdottir OB, Steen IS, et al. The development and feasibility of a web-based intervention with diaries and situational feedback via smartphone to support self-management in patients with diabetes type 2. Diabetes Res Clin Pract. 2012 Sep;97(3):385-93. PMID: 22578890. doi: http://dx.doi.org/10.1016/j.diabres.2012.04.019.
138. Neve M, Morgan P, Callister R, Collins C. Evaluating weight loss, website use, and attrition in commercial web-based weight loss programs. Obesity Research and Clinical Practice. 2012 October;6:60. PMID: 70899206. doi: http://dx.doi.org/10.1016/j.orcp.2012.08.123.
139. Newell D, McHiro RB. Increasing compliance toward home exercise in chiropractic patients using SMS texting: A pilot study. Clinical Chiropractic. 2012;15(3/4):107-11. PMID: 2012608980. doi: 10.1016/j.clch.2012.10.042.
140. Nguyen AD-V. The Influence of Message Framing on Engagement with a Mobile Application for Motivating Exercise [Thesis]. USA: Massachusetts Institute of Technology; 2011.
141. Nguyen TN, Su S, Celler B, Nguyen H. Advanced portable remote monitoring system for the regulation of treadmill running exercises. Artificial Intelligence in Medicine. 2014 Jun;61(2):119-26. PMID: WOS:000338822200006. doi: 10.1016/j.artmed.2014.05.002.
142. Norris-Ellis TT. Association between availability of an on-site workplace wellness program, physical activity, and nutrition [Dissertation Abstract; PhD]. Minnesota, USA: Walden University; 2012.
143. O'Brien T. Mobile Health Technology Interventions to Improve the Health Status of Older Rural Women. USA: Medical University of South Carolina; 2013.
144. Olmen Jv, Ku GM, Pelt Mv, Kalobu JC, Hen H, Darras C, et al. The effectiveness of text messages support for diabetes self-management: protocol of the TEXT4DSM study in the democratic Republic of Congo, Cambodia and the Philippines. BMC Public Health. 2013;13(423). PMID: 20133176244.
145. Oloritun ROO, Taha B. M. J.; Moturu, Sai; Madan, Anmol; Pentland, Alex Sandy; Khayal, Inas. Change in BMI accurately predicted by social exposure to acquaintances. PLoS One. 2013;8(11):e79238.
146. Ormseth SR. Process and outcome evaluation of a social-networking website for health promotion [Thesis]. California, USA: Loma Linda University; 2011.
147. Orsama AL, Lahteenmaki J, Harno K, Kulju M, Wintergerst E, Schachner H, et al. Active assistance technology reduces glycosylated hemoglobin and weight in individuals with type 2 diabetes: Results of a theory-based randomized trial. Diabetes Technol Ther. 2013;15(8):662-9. PMID: CN-00918633. doi: 10.1089/dia.2013.0056.
148. Owens SL, L.; McDonough, S.; Green, K.; Loftin, M. Feasibility of a home-delivered Internet obesity prevention program for fourth-grade students. Pediatr Exerc Sci. 2009 Aug;21(3):279-90. PMID: 19827452.
149. Po'e EK, Heerman WJ, Mistry RS, Barkin SL. Growing Right Onto Wellness (GROW): a family-centered, community-based obesity prevention randomized controlled trial for preschool child-parent pairs. Contemporary Clinical Trials. 2013;36(2):436-49. PMID: 20143032310. doi: http://dx.doi.org/10.1016/j.cct.2013.08.013.
150. Postrach E, Aspalter R, Elbelt U, Koller M, Schulzke JD, Valentini L. Efficacy of an internet-based weight loss program: A proof-of-principle trial. Clinical Nutrition, Supplement. 2012 September;7 (1):217. PMID: 70903932. doi: http://dx.doi.org/10.1016/S1744-1161%2812%2970538-0.
151. Powers MA, March SB, Evert A. Use of Internet technology to support nutrition and diabetes self-management care. Diabetes Spectrum. 2008 Spring;21(2):91-9. PMID: 2008274529. doi: http://dx.doi.org/10.2337/diaspect.21.2.91.
152. Richardson CR, Buis LR, Janney AW, Goodrich DE, Sen A, Hess ML, et al. An Online Community Improves Adherence in an Internet-Mediated Walking Program. Part 1: Results of a Randomized Controlled Trial. Journal of Medical Internet Research. 2010 Oct-Dec;12(4):138-53. PMID: WOS:000285637900012. doi: 10.2196/jmir.1338.

153. Riva G, Wiederhold BK, Mantovani F, Gaggioli A. Interreality: The experiential use of technology in the treatment of obesity. Clinical Practice and Epidemiology in Mental Health. 2011;7:51-61. PMID: 2012223601. doi: http://dx.doi.org/10.2174/1745017901107010051.
154. Robertson C, Kattelmann K, Ren C. Control of type 2 diabetes mellitus using interactive internet-based support on a Northern Plains Indian reservation: A pilot study. Topics in Clinical Nutrition. 2007 April/June;22(2):185-93. PMID: 2007236207. doi: http://dx.doi.org/10.1097/01.TIN.0000270137.00099.91.
155. Rollins BM. Weight-related beliefs, behaviors, and social networks of obese, young adult African-American women: Implications for healthy weight interventions [Dissertation Abstract]. USA: Texas A&M University; 2014.
156. Rossi MC, Nicolucci A, Lucisano G, Pellegrini F, Di Bartolo P, Miselli V, et al. Impact of the "Diabetes Interactive Diary" telemedicine system on metabolic control, risk of hypoglycemia, and quality of life: a randomized clinical trial in type 1 diabetes. Diabetes Technol Ther. 2013 Aug;15(8):670-9. PMID: 23844569. doi: http://dx.doi.org/10.1089/dia.2013.0021.
157. Rotheram-Borus MJ, Tomlinson M, Gwegwe M, Comulada WS, Kaufman N, Keim M. Diabetes buddies: Peer support through a mobile phone buddy system. The Diabetes Educator. 2012;38(3):357-65. doi: http://dx.doi.org/10.1177/0145721712444617.
158. Royall D, Brauer P, Ackah E, Dwyer JJM, Edwards AM, Goy R, et al. Eliciting provider and patient perspectives to develop an interdisciplinary obesity management planning framework in primary care. Canadian Journal of Diabetes. 2011 May;35 (2):172. PMID: 70446208.
159. Rutanen R, Nygard C-H, Moilanen J, Mikkola T, Raitanen J, Tomas E, et al. Effect of physical exercise on work ability and daily strain in symptomatic menopausal women: A randomized controlled trial. Work: Journal of Prevention, Assessment and Rehabilitation. 2014;47(2):281-6.
160. Ruxton C, Derbyshire E. Strategies to encourage healthy eating among children and young adults. Primary Health Care. 2014;24(5):33-41. PMID: 2012591987.
161. Schap TE, Six BL, Delp EJ, Ebert DS, Kerr DA, Boushey CJ. Adolescents in the United States can identify familiar foods at the time of consumption and when prompted with an image 14 h postprandial, but poorly estimate portions. Public Health Nutr. 2011 Jul;14(7):1184-91. PMID: 21324224. doi: http://dx.doi.org/10.1017/S1368980010003794.
162. Seely E. Facebook as social supprt for a one-time weight loss intervention among college students [(M.S.); Southern Illinois University Carbondale,; 2013.]2013.
163. Sevick MA, Korytkowski M, Stone RA, Piraino B, Ren D, Sereika S, et al. Biophysiologic Outcomes of the Enhancing Adherence in Type 2 Diabetes (ENHANCE) Trial. Journal of the Academy of Nutrition & Dietetics. 2012;112(8):1147-57. PMID: 2011617808. doi: 10.1016/j.jand.2012.05.008.
164. Sevick MA, Zickmund S, Korytkowski M, Piraino B, Sereika S, Mihalko S, et al. Design, feasibility, and acceptability of an intervention using personal digital assistant-based self-monitoring in managing type 2 diabetes. Contemporary clinical trials. 2008;29(3):396-409. PMID: CN-00637171. doi: 10.1016/j.cct.2007.09.004.
165. Shaw RJ. A Mobile Health Intervention to Sustain Recent Weight loss: Duke University; 2012.
166. Shay L. Self-monitoring and weight management. On-Line Journal of Nursing Informatics. 2008;12(1):10p. PMID: 2009863811.
167. Shim HS, Kim HS. Cellular phone and internet-based individual intervention on fasting plasma glucose, blood pressure, and waist circumference for people with metabolic syndrome [abstract]. Diabetes Technol Ther. 2011;13(2):173-293. PMID: Cn-00869649. doi: 10.1089/dia.2010.1219.
168. Skrøvseth SO, Arsand E, Godtliebsen F, Hartvigsen G. Mobile phone-based pattern recognition and data analysis for patients with type 1 diabetes. Diabetes Technol Ther. 2012 Dec;14(12):1098-104. PMID: 23035775. doi: http://dx.doi.org/10.1089/dia.2012.0160.
169. Skrøvseth SO, Arsand E, Godtliebsen F, Joakimsen RM. Model driven mobile care for patients with type 1 diabetes. Stud Health Technol Inform. 2012;180:1045-9. PMID: 22874353.
170. Sneed MM. Blogging in the Fatosphere: A Qualitative Study of Perceptions of Personal Risks and Benefits for Women who Blog about Weight, Weight Loss, and Dieting Issues [Thesis / Dissertation ETD]. USA: East Tennessee State University; 2012.
171. Snively HL. The BALANCE study (Bioengineering Approaches for Lifestyle Activity and Nutrition Continuous Engagement) : developing and testing a novel device for measuring energy balance in real-time [Thesis]. Washington, USA: University of Washington; 2011.

172. Spring B, Gotsis M, Paiva A, Spruijt-Metz D. Healthy apps: mobile devices for continuous monitoring and intervention. IEEE Pulse. 2013 Nov-Dec;4(6):34-40. PMID: 24233190. doi: http://dx.doi.org/10.1109/MPUL.2013.2279620.
173. Steinberg DM, Tate DF, Bennett GG, Ennett S, Samuel-Hodge C, Ward DS. The efficacy of a daily self-weighing weight loss intervention using smart scales and e-mail. Obesity (Silver Spring, Md). 2013;21(9):1789-97. PMID: Cn-00913984. doi: 10.1002/oby.20396.
174. Stellefson M, Chaney B, Barry AE, Chavarria E, Tennant B, Walsh-Childers K, et al. Web 2.0 chronic disease self-management for older adults: a systematic review. Journal of Medical Internet Research. 2013;15(2):e35. PMID: 23410671. doi: http://dx.doi.org/10.2196/jmir.2439.
175. Stephens J, Allen J. First Place Winner: Randomized Controlled Pilot Study Testing Use of Smartphone Technology for Obesity Treatment. Journal of Cardiovascular Nursing. 2014;29(5):386-. PMID: 2012778884.
176. Stephens J, Allen JK, Himmelfarb CRD. "Smart" coaching to promote physical activity, diet change, and cardiovascular health. . Journal of Cardiovascular Nursing. 2011;26(4):282-4.
177. Stevens CJ, Bryan AD. Rebranding exercise: There's an app for that. . American Journal of Health Promotion. 2012;27(2):69-70. doi: http://dx.doi.org/10.4278/ajhp.120711-CIT-338.
178. Stuckey M, Fulkerson R, Read E, Russell-Minda E, Munoz C, Kleinstiver P, et al. Remote monitoring technologies for the prevention of metabolic syndrome: the Diabetes and Technology for Increased Activity (DaTA) study. Journal of Diabetes Science and Technology. 2011 Jul;5(4):936-44. PMID: 21880237.
179. Stuckey M, Russell-Minda E, Read E, Munoz C, Shoemaker K, Kleinstiver P, et al. Diabetes and Technology for Increased Activity (DaTA) study: results of a remote monitoring intervention for prevention of metabolic syndrome. Journal of Diabetes Science and Technology. 2011 Jul;5(4):928-35. PMID: 21880236.
180. Stuckey MI, Shapiro S, Sabourin KJ, Munoz C, Petrella RJ. Effects of a 12-week remote health monitoring intervention on metabolic syndrome risk factors. Canadian Journal of Diabetes. 2011 May;35 (2):159. PMID: 70446166.
181. Sun M, Burke LE, Mao Z-H, Chen Y, Chen H-C, Bai Y, et al., editors. eButton: A Wearable Computer for Health Monitoring and Personal Assistance. DAC '14 - Design Automation Conference; 2014 2014; San Francisco, California, USA. New York, NY, USA: ACM.
182. Talbot TB. Virtual reality and interactive gaming technology for obese and diabetic children: is military medical technology applicable? Journal of Diabetes Science and Technology. 2011 Mar;5(2):234-8. PMID: 21527087.
183. Tamura T, Maeno S, Hattori T, Kimura Y, Yoshida M, Minato K. Assessment of participant compliance with a Web-based home healthcare system for promoting specific health checkups. Biocybernetics and Biomedical Engineering. 2014;34(1):63-9. PMID: CN-00982078. doi: http://dx.doi.org/10.1016/j.bbe.2013.12.001.
184. Tani S, Marukami T, Matsuda A, Shindo A, Takemoto K, Inada H. Development of a health management support system for patients with diabetes mellitus at home. J Med Syst. 2010 Jun;34(3):223-8. PMID: 20503606.
185. Tate DF. Application of innovative technologies in the prevention and treatment of overweight in children and adolescents. In: Jelalian E, Steele RG, editors. Handbook of childhood and adolescent obesity. New York, NY, US: Springer Science + Business Media; 2008. p. 378-404.
186. Tate EB, Spruijt-Metz D, O'Reilly G, Jordan-Marsh M, Gotsis M, Pentz MA, et al. mHealth approaches to child obesity prevention: Successes, unique challenges, and next directions. Translational Behavioral Medicine. 2013 December;3(4):406-15. PMID: 2013733461. doi: http://dx.doi.org/10.1007/s13142-013-0222-3.
187. Tettey N-S. An online evaluation of a new web-based source of information on eating healthy and being active designed for African American women: Exploring relationships among personal-level variables and website ratings [Dissertation Abstract]. USA: Columbia University; 2010.
188. Thomas JG, Bond DS. Review of innovations in digital health technology to promote weight control. Current Diabetes Reports. 2014;14(5). PMID: 20143167184. doi: http://dx.doi.org/10.1007/s11892-014-0485-1.
189. Thomas JG, Bond DS, Sarwer DB, Wing RR. Technology for behavioral assessment and intervention in bariatric surgery. Surg. 2011 Jul-Aug;7(4):548-57. PMID: 21514246. doi: http://dx.doi.org/10.1016/j.soard.2011.02.009.

190. Thompson-Felty C. iPhone applications and improvement in weight and health parameters a randomized controlled trial [Thesis]. USA: Arizona State University; 2014.
191. Trevorrow P. Technology running the world: The nike+ipod kit and levels of physical activity. / La technologie qui fait avancer le monde : le kit « Nike + iPod » et les niveaux d'activité physique. Society & Leisure / Loisir & Société. 2012 Spring2012;35(1):131-54. PMID: 83438713.
192. Tu H. Development of an Activity Pattern Recognition System with Smartphone-Applying to Improve the Physical Activity of Overweight People [Thesis]. China: National Cheng Kung University Institute of Medical Informatics; 2011.
193. Turner-McGrievy GM. Pounds off digitally (pod): An examination of the use of podcasting to promote weight loss [Dissertation Abstract]. Chapel Hill, USA: University of North Carolina Chapel Hill; 2010.
194. Uden L, Helo P. Designing mobile interfaces using activity theory. International Journal of Mobile Communications. 2008;6(5):616-32. PMID: 34372589. doi: 10.1504/IJMC.2008.019325.
195. Vandelanotte C, Duncan MJ, Plotnikoff RC, Mummery WK. Do participants' preferences for mode of delivery (text, video, or both) influence the effectiveness of a Web-based physical activity intervention? Journal of medical Internet research. 2012;14(1):e37. PMID: CN-00814723. doi: 10.2196/jmir.1998.
196. Vandelanotte C, Mummery WK. Qualitative and quantitative research into the development and feasibility of a video-tailored physical activity intervention. . The International Journal of Behavioral Nutrition and Physical Activity. 2011;8:70. doi: http://dx.doi.org/10.1186/1479-5868-8-70.
197. Verhoeven F, Tanja-Dijkstra K, Nijland N, Eysenbach G, van Gemert-Pijnen L. Asynchronous and synchronous teleconsultation for diabetes care: a systematic literature review. Journal of Diabetes Science and Technology. 2010 May;4(3):666-84. PMID: 20513335.
198. Vermeulen J, Neyens JCL, Spreeuwenberg MD, van Rossum E, Sipers W, Habets H, et al. User-centered development and testing of a monitoring system that provides feedback regarding physical functioning to elderly people. Patient Preference and Adherence. 2013 January;7:843-54. PMID: 2013559118. doi: http://dx.doi.org/10.2147/PPA.S45897.

199. Vodopivec-Jamsek V, de Jongh T, Gurol-Urganci I, Atun R, Car J. Mobile phone messaging for preventive health care. Cochrane Database of Systematic Reviews. 2012 (12). PMID: 2011917380.
200. Vuong AM, Huber JC, Jr., Bolin JN, Ory MG, Moudouni DM, Helduser J, et al. Factors affecting acceptability and usability of technological approaches to diabetes self-management: a case study. Diabetes Technol Ther. 2012 Dec;14(12):1178-82. PMID: 23013155. doi: http://dx.doi.org/10.1089/dia.2012.0139.
201. Wang J. Social problem solving and adherence to self-monitoring in association with changes in weight and cardiometabolic risk factors in a behavioral weight loss trial: University of Pittsburgh; 2010.
202. Weiler R, Neyndorff C. BJSM social media contributes to health policy rethink: a physical activity success story in Hertfordshire. BJSM online. 2013 Jun;47(9):593-4. PMID: 23444393. doi: http://dx.doi.org/10.1136/bjsports-2012-091945.
203. Weineland S, Arvidsson D, Kakoulidis TP, Dahl J. Acceptance and commitment therapy for bariatric surgery patients, a pilot RCT. Obesity Research and Clinical Practice. 2012 January-March;6(1):e21-e30. PMID: 2012048331. doi: http://dx.doi.org/10.1016/j.orcp.2011.04.004.
204. Wier MF, Ariëns GA, Dekkers JC, Hendriksen IJ, Smid T, Mechelen W. Phone and e-mail counselling are effective for weight management in an overweight working population: a randomized controlled trial. BMC public health. 2009;9:6. PMID: CN-00697655. doi: 10.1186/1471-2458-9-6.
205. Winett RA, Anderson ES, Wojcik JR, Winett SG, Moore S, Blake C. Guide to health: A randomized controlled trial of the effects of a completely web-based intervention on physical activity, fruit and vegetable consumption, and body weight. Translational Behavioral Medicine. 2011 March;1(1):165-74. PMID: 2012370442. doi: http://dx.doi.org/10.1007/s13142-010-0006-y.
206. Woolford SJ, Clark SJ. Tailored mobile phone text messages as an adjunct to obesity treatment for adolescents. Journal of Adolescent Health. 2009 Feb;44(2):S2-S3. PMID: WOS:000263270100006.

207. Yu CH, Parsons J, Mamdani M, Lebovic G, Shah BR, Bhattacharyya O, et al. Designing and evaluating a web-based self-management site for patients with type 2 diabetes--systematic website

development and study protocol. BMC Med Inf Decis Mak. 2012;12:57. PMID: 22726578. doi: http://dx.doi.org/10.1186/1472-6947-12-57.
208. Yumak Z, Pu P. Survey of Sensor-Based Personal Wellness Management Systems. BioNanoScience. 2013 September;3(3):254-69. PMID: 2013709202. doi: http://dx.doi.org/10.1007/s12668-013-0099-0.
209. Zenk SN, Horoi I, McDonald A, Corte C, Riley B, Odoms-Young AM. Ecological momentary assessment of environmental and personal factors and snack food intake in African American women. Appetite. 2014;83:333-41. PMID: 2014-45333-044. doi: 10.1016/j.appet.2014.09.008.
210. Zheng H, Nugent C, McCullagh P, Huang Y, Zhang S, Burns W, et al. Smart self management: assistive technology to support people with chronic disease. J Telemed Telecare. 2010;16(4):224-7. PMID: 20511581. doi: http://dx.doi.org/10.1258/jtt.2010.004017.
211. Zulman DM, Damschroder LJ, Smith RG, Resnick PJ, Sen A, Krupka EL, et al. Implementation and evaluation of an incentivized Internet-mediated walking program for obese adults. Translational Behavioral Medicine. 2013 December;3(4):357-69. PMID: 2013733456. doi: http://dx.doi.org/10.1007/s13142-013-0211-6.
